# Photochemical Radical Bicyclization of 1,5-Enynes: Divergent Synthesis of Fluorenes and Azepinones

**DOI:** 10.1021/acs.orglett.3c04246

**Published:** 2024-01-17

**Authors:** Babasaheb Sopan Gore, Chun-Cheng Chen, Ping-Yu Lin, Jeh-Jeng Wang

**Affiliations:** † Department of Medicinal and Applied Chemistry, Kaohsiung Medical University, No. 100, Shih-Chuan First Rd, Sanmin District, Kaohsiung City 807, Taiwan; ‡ Department of Medical Research, Kaohsiung Medical University Hospital, No. 100, Tzyou First Rd, Sanmin District, Kaohsiung City 807, Taiwan

## Abstract

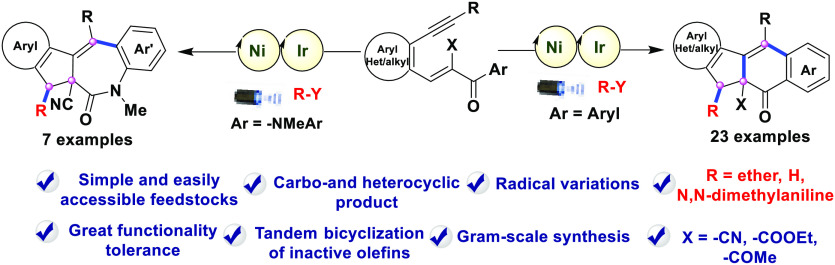

A dual nickel- and iridium-photocatalyzed radical cascade bicyclization reaction for the synthesis of highly complex molecular structures in an atom- and step-economic manner has been described. A series of radical precursors are utilized for the divergent synthesis of diversely substituted fluorenes and indenoazepinones bearing quaternary carbons by using cascade cyclization reactions of 1,5-enynes. This reaction is characterized by its mild conditions, broad substrate scope, excellent selectivity, and satisfactory yield including facile scale-up synthesis.

The radical cascade reaction is a fundamental method for organic synthesis because it allows direct access to the synthesis of biologically important targets.^1^ Additionally, such an approach enables the production of highly functionalized polycyclic architectures from readily accessible feedstocks in a concise, efficient, and straightforward manner.^2^ Among these, 1,5-enynes are attractive building blocks in cascade cyclization owing to their varying levels of reactivity for one-pot heterocycle and carbocycle synthesis.^3^ The key advantage of this process is its ability to produce structural complexity under mild conditions with high atom economy.^4^ Several cycloisomerization reactions have been observed in 1,5-enynes,^5a^ such as metal-catalyzed cycloisomerization, which includes nucleophilic and electrophilic additions.^5b^ However, the radical bicyclization of 1,5-enynes remains unexplored under visible-light-mediated photoredox catalytic conditions. Owing to their low reactivity and high steric factors, the use of the internal alkenes of enynes as radical acceptors poses considerable challenges and remains largely unsolved. Jiang et al. developed a protocol for the tandem 5-*exo*-*dig*/6-*endo*-*trig* carbonylation of 1,5-enynes with aryl sulfonyls in the presence of TBAI/BPO/PivOH and copper (Scheme 1a).^6a^ Similarly, in 2013, Alabugin et al. described the regioselective synthesis of substituted indenes with Bu_3_Sn−H as a radical source under conventional conditions (Scheme 1b).^6b^ Recently, in 2020 Wu and co-workers reported copper-catalyzed radical bicyclization of 1,6-enynes with aldehydes for the synthesis of benzo[*a*]fluorenone skeletons under heating conditions (Scheme 1c).^6c^ Despite recent advances, the development of robust, reliable multiple-ring polycyclic molecules containing quaternary carbon centers^7^ and the use of ether, α-amino alkyl, or tin hydride radicals in the tandem bicyclization of 1,5-enynes remain largely unexplored. Meanwhile, polycyclic aromatic hydrocarbons and azepine derivatives are valuable resources for material science, drug discovery, pharmaceuticals, and other bioactive compounds (Figure 1).^8^ Results from previous studies^9a^ and our interest in enyne-assisted cyclization^9b−9d^ led to the investigation of a previously unexplored application of radical cascade cyclization induced by photoredox catalysis. Further, the realization of our envisaged protocol will provide new opportunities for the synthesis of valuable and sterically demanding quaternary carbon centers. This article describes the development of an efficient, practical, and modular platform for building highly diastereoselective and regiodivergent 5-*exo*-*dig* bicyclizations with high atom and step economy in a single operation to construct the substituted fluorene and azepinone scaffolds under visible-light induced photoredox catalysis (Scheme 1d). Initially, we examined the intermolecular oxidative α-functionalization of an ether such as 1,4-dioxane with conjugated enynes of internal olefins. The first experiment involved 1,4-dioxane as the substrate and solvent coupled with (*E*)-2-benzoyl-3-(2-phenylethynyl)phenyl)acrylonitrile (**1a**) as the electron-deficient internal olefin under blue LED irradiation with 1 mol % of Ir(III) sensitizer (Table 1). The reaction did not yield the desired product in the absence of the catalyst or oxidant (entries 1 and 2). Further screening of various oxidants, such as K_2_S_2_O_8_, H_2_O_2_, DTBP, and TBHP (entries 3–6), in the presence of NiCl_2_ (10 mol %), 1,10-phenanthroline (20 mol %), [Ir{dFCF_3_ppy}_2_(bpy)]PF_6_ (1.0 mol %), and DIPEA (10 mol %) under blue LED irradiation indicated the effectiveness of TBHP as an oxidant, affording an 88% yield of benzo[*b*]fluorene-10a-carbonitrile **2a** (entry 6). However, the reaction produced the desired product in a yield of only 62% after a prolonged reaction time (10 h) (entry 7). Several commercially available photocatalysts (TBADT and Ru(bpy)_3_(PF_6_)_2_) were then examined (entries 8 and 9), but the performance remained unaltered. Increasing or decreasing NiCl_2_ as well as TBHP was found to be ineffective (entries 10–13). Several other catalysts commonly used for catalytic transformations were also screened, including CuCl_2_, FeCl_2_, NiBr_2_, and Ni(acac)_2_. Each of these catalysts performed poorly, and their use considerably inhibited the generation of **2a** (entries 14–17). Finally, replacing Hünig’s base with K_3_PO_4_, Na_2_CO_3_, and TEA did not form the desired product but rather caused the decomposition of the starting material (entries 18–20). The diastereomeric ratio of these products was determined using ^1^H NMR and found to be between 1:1 and 1.5:1; both were easily isolated as major and minor products using standard silica gel chromatography. As a consequence of the versatility of radical cascade cyclization, we investigated intermolecular radical cascades of 1,5-enyne substrates with a variety of radical precursors (Scheme 2). When the α–β-conjugated 1,5-enynes were treated under optimized conditions (Table 1, entry 6) with dioxane as a radical initiator, a separable diastereomeric mixture (1.5:1) of compounds **2a** was obtained at a yield of 88% (Scheme 2). Although the reaction was shown to be robust, the designed photocatalytic process has not been investigated for fluorene synthesis. The scope of the substrate was later evaluated by extensively varying and probing different substituents at various positions of the aromatic ring, such as methoxy, methyl, fluoro, and chloro groups. In all cases, the reactions occurred smoothly, which indicated comparable reactivity and provided good yields with moderate to excellent diastereoselectivity (**2b**–**2k**) (Scheme 2). Cyclohexene-incorporated enyne (**1l**) was also tested, and the corresponding product (**2l**) was obtained in high yields (Scheme 2). Next, we also successfully demonstrated that the reaction of THF (**1m**), ester (**1n**), and ketone (**1o**) incorporated fluorene synthesis in good yields (Scheme 2).

**Figure 1:**
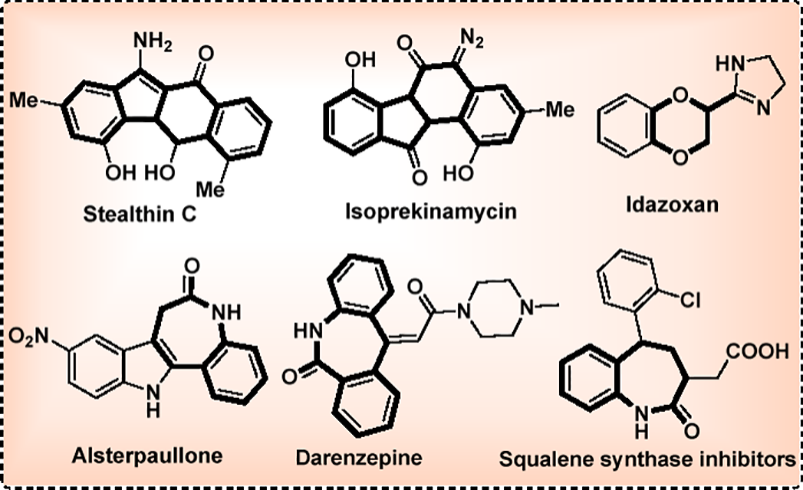
Representative bioactive scaffolds.

**Scheme 1:**
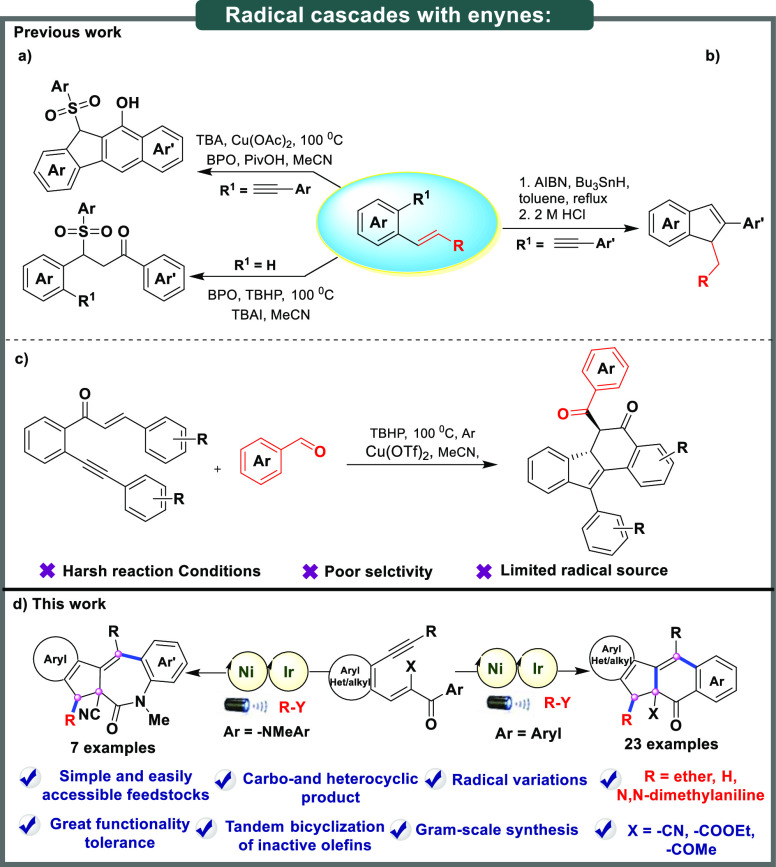
Different Reactivity Patterns for Radical Cascades of Enynes

**Scheme 2:**
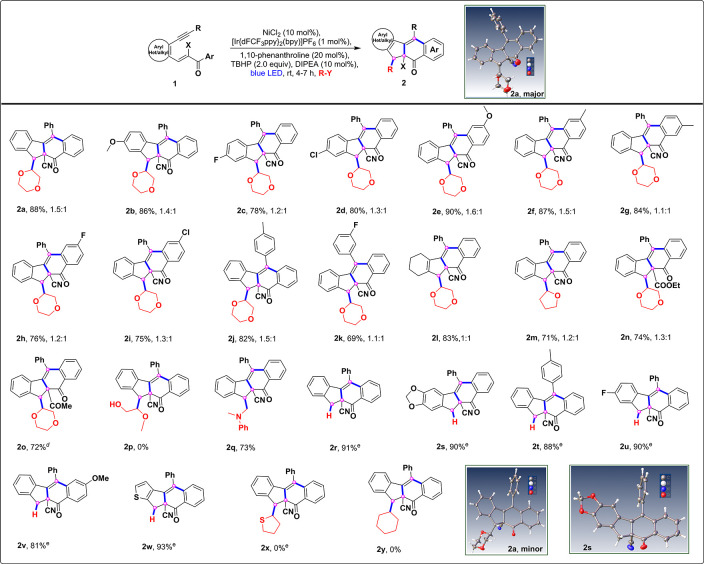
Substrate Scope for Radical Bicyclization– Standard conditions: **1** (0.2 mmol), NiCl_2_ (10 mol %), 1,10-phenanthroline (20 mol%), DIPEA (10 mol %), TBHP (70% aq, 2.0 equiv), R–Y = 1,4-dioxane/THF/2-methoxyethanol/cyclohexane (3.0 mL), [Ir{dFCF_3_ppy}_2_(bpy)]PF_6_ (1.0 mol%), stirred for 4–7 h and irradiated with blue LEDs (λ_max_ = 462 nm, latent temperature ∼25–30 °C). Isolated yields. Diastereomeric ratio determined by ^1^H NMR analysis. A mixture of isomers was isolated. 1,2-DCE (3.0 mL) was used as a solvent instead of 1,4-dioxane for *N*, *N*-dimethylaniline (2.0 equiv), Bu_3_Sn–H (2.0 equiv), and tetrahydrothiophene (2.0 equiv).

**Table 1:**
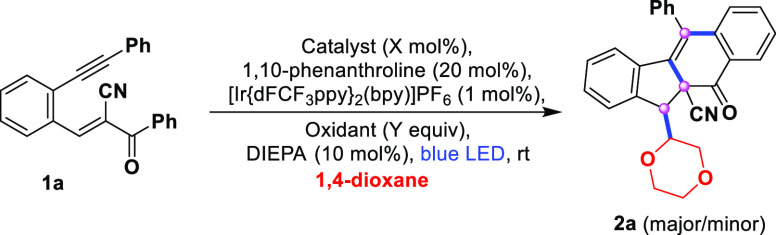
Optimization of Reaction Conditions for Radical Cyclization^a^

Entry	Catalyst (mol %)	Oxidant (*Y* equiv)	Yield (%)^b^	dr^c^
1			0	
2	NiCl_2_ (10)		0	
3	NiCl_2_ (10)	K_2_S_2_O_8_ (2.0)	trace	
4	NiCl_2_ (10)	H_2_O_2_ (2.0)	trace	
5	NiCl_2_ (10)	DTBP (2.0)	50	1.1:1
**6**	**NiCl**_**2**_**(10)**	**TBHP (2.0)**	**88**	**1.5:1**
7^d^	NiCl_2_ (10)	TBHP (2.0)	62	1.3:1
8^e^	NiCl_2_ (10)	TBHP (2.0)	trace	
9^f^	NiCl_2_ (10)	TBHP (2.0)	15	1:1
10	NiCl_2_ (20)	TBHP (2.0)	58	1.3:1
11	NiCl_2_ (5)	TBHP (2.0)	69	1.4:1
12	NiCl_2_ (10)	TBHP (3.0)	71	1.3:1
13	NiCl_2_ (10)	TBHP (1.0)	60	1.3:1
14	CuCl_2_ (10)	TBHP (2.0)	53	1.2:1
15	FeCl_2_ (10)	TBHP (3.0)	10	1:1
16	NiBr_2_ (10)	TBHP (2.0)	46	1.1:1
17	Ni(acac)_2_ (10)	TBHP (3.0)	48	1.1:1
18^g^	NiCl_2_ (10)	TBHP (2.0)	0	
19^h^	NiCl_2_ (10)	TBHP (2.0)	0	
20^i^	NiCl_2_ (10)	TBHP (2.0)	trace	

a Standard conditions: **1a** (0.20 mmol), catalyst (X mol %), 1,10-phenanthroline (20 mol %), [Ir{dFCF_3_ppy}_2_(bpy)]PF_6_ = Ir(ΙΙΙ) = (1.0 mol %), and DIPEA (10 mol %) in 1,4-dioxane (3.0 mL) were stirred for 4 h and irradiated with blue LED (λ_max_ = 462 nm, latent temperature ∼25–30 °C).

b Isolated reaction yield.

c ^1^H NMR analysis of the crude reaction mixture,

d Reaction mixture stirred at 10 h.

e TBADT used instead of Ir (III).

f [Ru(bpy)_3_](PF_6_)_2_ used instead of Ir (III).

g K_3_PO_4_ used instead of DIPEA.

h Na_2_CO_3_ used instead of DIPEA.

i TEA used instead of DIPEA.

The present method is applicable for the selective synthesis of fluorene derivatives under photoredox catalysis with *N*,*N*-dimethylaniline and Bu_3_Sn–H (hydrogen radical), as the radical initiators under slightly modified reaction conditions were evaluated (Table S1, see the Supporting Information for more details). In all cases, the electron-donating, halogen, and heterocyclic derivatives afforded the corresponding products in moderate to excellent yields, including simple derivatives (**2q**–**2w**) (Scheme 2). However, when the 2-methoxyethanol, tetrahydrothiophene, and cyclohexane as radical precursors were subjected to intermolecular bicyclization, no desired products **2p**, **2x**, and **2y** were obtained (Scheme 2). Next, these results prompted a more detailed examination of amino-tethered enynes, which showed a high tolerance and provided densely substituted indeno-fused azepine^10^ scaffolds (**4a**–**4g**) in good reaction yields (Scheme 3). Moreover, the structure of compounds **2a** (major:minor) and **2s** were confirmed by using X-ray crystallography. To assess the practicality of the radical cascade protocol, both sequential gram- and large-scale reactions were performed. No considerable decrease in the yield was observed in either process. As outlined in Scheme 4, the bicyclization products **2r** and **4a** yielded 72% and 76% yield, respectively (Scheme 4a,b). We then investigated the late-stage skeletal transformation of sterically hindered fluorenes, synthesizing hydroxy-fused fluorene (**5**) in high yield by reducing compound **2r** (Scheme 4c). Finally, to explore the reaction mechanistic information on the cascade bicyclization of the designed enynes, a couple of control experiments were conducted. For the former, when TEMPO and BHT were added to the reaction of **1a** under standard conditions, adduct **6** could be detected by ESI-HRMS (see the Supporting Information for more details) while the formation of **2a** was inhibited (Scheme 4d). This result revealed that the radical process might be involved in the cascade bicyclization process. Based on the above observations and previous studies on Ni/Ir dual photocatalysis,^12^ a plausible mechanism for the radical cyclization of 1,5-enynes is illustrated in Scheme 5. The oxidative addition of Ni^0^ to compound **1a** would produce a Ni^II^ complex (oxa-nickelacle), while tBuOOH would generate tBuO^•^ and ^•^OH radicals under the designed conditions and quickly extract the hydrogen atom from 1,4-dioxane to produce a dioxane radical species. Next, the dioxane radical reacts with intermediate [**A**] to produce Ni^III^ intermediate [**B**], followed by subsequent reductive elimination to produce a new C–C bond and oxygen-centered radical intermediate [**C**]. Next, the long-lived excited state Ir^III*^ photocatalyst transfers a single electron to Ni^I^ to generate both Ir^IV^ and Ni^0^, thus completing the nickel catalytic cycle. The tautomerization of the intermediate [**C**] would then generate a five- membered cyclic structure with the vinyl radical intermediate [**E**] via 5-*exo-dig* cyclization. Finally, the intramolecular reaction of the vinyl radical intermediate with nearby arenes produces a new cyclohexadienyl radical species [**F**], which is oxidized to cationic intermediates by reducing Ir^IV^ to Ir^III^. The cationic species eventually undergoes deprotonation to form product **2**.

**Scheme 3:**
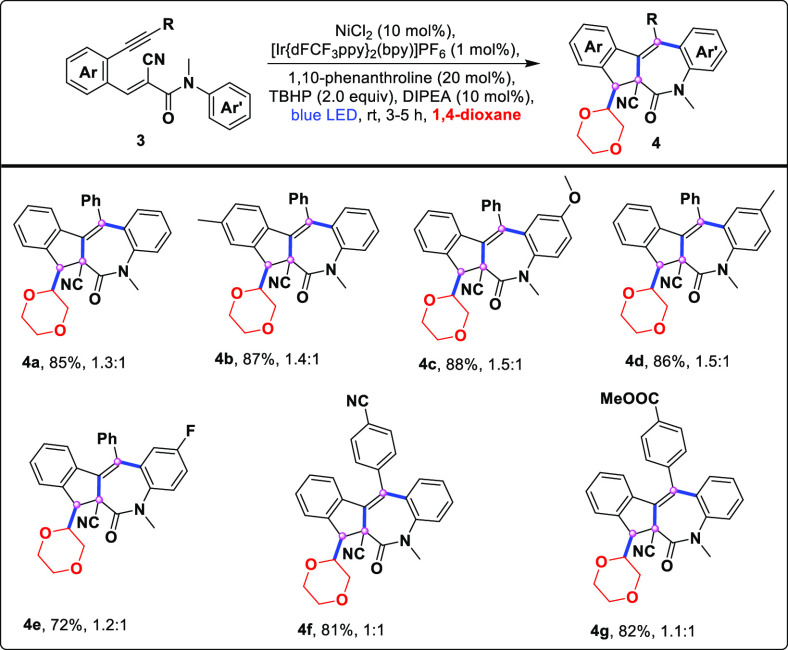
Substrate Scope for Azepine Derivatives– Standard conditions: **3** (0.2 mmol), NiCl_2_ (10 mol %), 1,10-phenanthroline (20 mol%), DIPEA (10 mol %), TBHP (70% aq., 2.0 equiv), 1,4-dioxane (3.0 mL), [Ir{dFCF_3_ppy}_2_(bpy)]PF_6_ (1.0 mol%), stirred for 3–5 h and irradiated with blue LEDs (λ_max_ = 462 nm, latent temperature ∼25–30 °C). Isolated yields. Diastereomeric ratio determined by ^1^H NMR analysis.

**Scheme 4:**
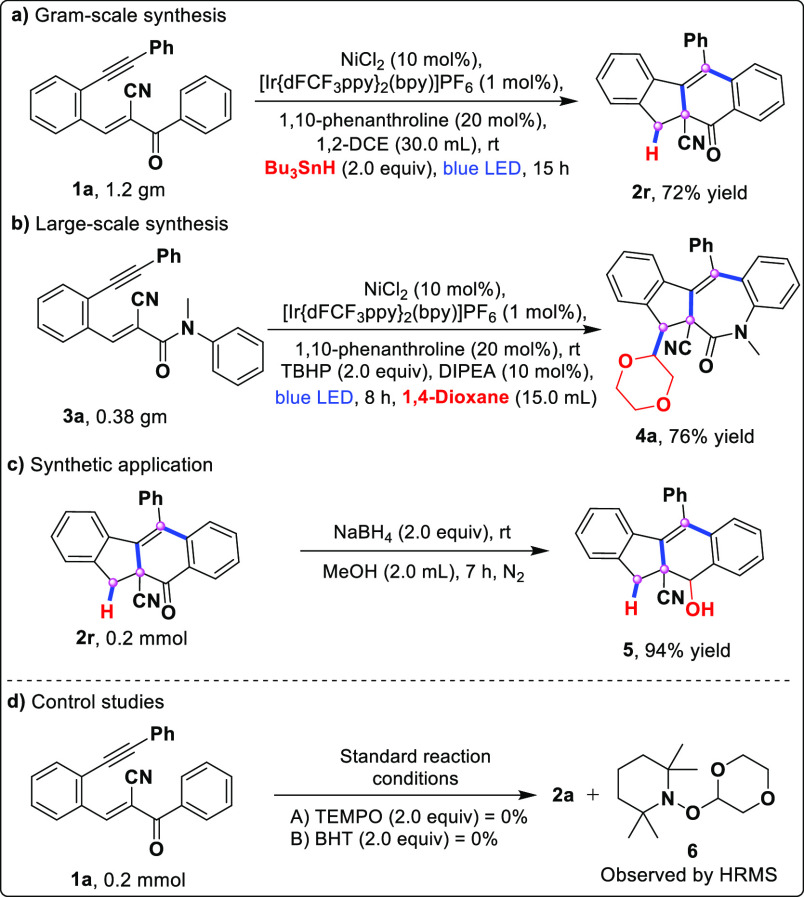
Synthetic Applications and Control Studies

**Scheme 5:**
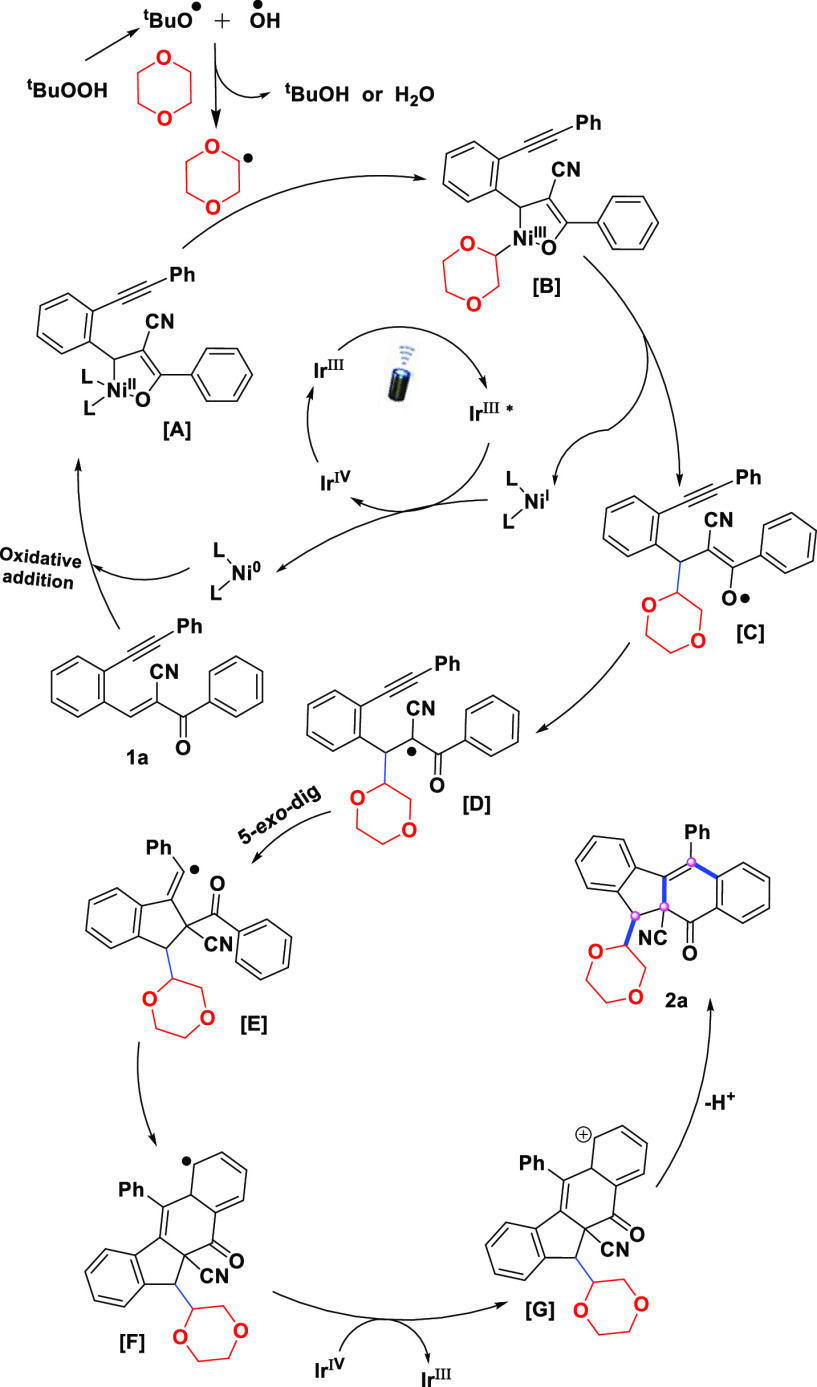
Plausible Mechanism of Intermolecular Radical Bicyclization of 1,5-Enynes

In conclusion, we have developed a radical tandem bicyclization strategy of conjugated internal 1,5-enynes to construct the substituted fluorenes and azepinones framework with various radical precursors under dual nickel (an earth-abundant metal)–Ir photoredox catalysis. This study provides a novel step- and atom-economical approach for synthesizing the carbo-/heterocyclic product with wide functionality and regioselectivity. These skeletons are also valuable feedstocks in medicinal chemistry, as they are difficult to synthesize using conventional approaches. Furthermore, the construction of highly chemo- and regioselective organic and medicinally important skeletons by using α,β-conjugated 1,5-enynes is in progress in our laboratory.

## Data Availability

The data underlying this study are available in the published article and its online Supporting Information.

## Supporting Information Available

Experimental data, spectral characterization, solvent system, method for crystal growth of **2a** and **2s**, and X-ray crystal data (PDF)
