# Supplementary material for: Photochemical Radical Bicyclization of 1,5-Enynes: Divergent Synthesis of Fluorenes and Azepinones
Source: Org Lett. 2024 Jan 17;26(3):757–62. doi: 10.1021/acs.orglett.3c04246 (PMC10825824; doi:10.1021/acs.orglett.3c04246)

Supplementary Information

Photochemical Radical Bicyclization of 1,5-Enynes: Divergent Synthesis of fluorenes and azepinones

Babasaheb Sopan Gore,[†] Chun-Cheng Chen,[†] Ping-Yu Lin,[†] and Jeh-Jeng Wang*[†‡]

[†]Department of Medicinal and Applied Chemistry, Kaohsiung Medical University, No. 100, Shih-Chuan 1st Rd, Sanmin District, Kaohsiung City, 807 (Taiwan).

[‡]Department of Medical Research, Kaohsiung Medical University Hospital, No. 100, Tzyou 1st Rd, Sanmin District, Kaohsiung City, 807 (Taiwan). E-mail: jjwang@kmu.edu.tw

1. Table of contents

1. Table of contents S2

2. Materials and methods....................S3

3. Experimental data....................S5

  3.1 General procedure for the synthesis of final compounds **2** and **4**....................S5

  3.2 Gram scale reaction for the synthesis of compound **2r**....................S6

  3.3 Large scale synthesis of compound **4a**....................S6

4. Optimization reaction condition for radical cyclization …....................S8

5. Spectral characterization S9

6. References....................S33

7. Solvent system and method for crystal growth of **2a**, and **2s**
(Ellipsoid contour % probability levels) S34

8. Plausible mechanism of azepine formation....................S35

9. Copies of ^{1}H, ^{13}C and TEMPO adduct Mass....................S36

10. X-ray crystal data....................S154

2. Materials and methods

All air- and moisture-insensitive reactions were carried out under ambient atmosphere and monitored by thin layer chromatography (TLC). Concentration under reduced pressure was performed by rotary evaporation at 35-45 °C at an appropriate pressure. Purified compounds were further dried under high vacuum. Yields refer to purified and spectroscopically pure compounds, unless otherwise stated. Mass spectra and High-Resolution Mass spectral (HRMS) data was carried out using an Agilent6890N GC (JEOL JMS-700) TOF instrument, and the ion source is electrospray ionization (ESI), electronic ionization (EI), CI, and FAB as ion source at National Taiwan Normal University, Taipei City, Taiwan and ESI-TOF(FT-MS solariX) at National Sun Yat-Sen University, Kaohsiung, Taiwan, and LTQ Orbitrap XL (Thermo Fischer Scientific) at National Chung Hsing University. Liquid-chromatography mass spectra (LCMS) were measured using the LC-MS/MS-8045 (Shimadzu Corporation, Japan) at Kaohsiung Medical University, Kaohsiung, Taiwan. Melting points were determined on an EZ-Melt (Automated melting point apparatus).

Solvents

Bottle grade DMA, DMF, CAN, Toluene, MeOH, DMSO were purchased from Alfa acer. Anhydrous DCM was obtained from Phoenix Solvent Drying Systems. All deuterated solvents were purchased from Sigma-Aldrich.

Chromatography

Thin layer chromatography (TLC) was performed using Merck TLC aluminum sheets (silica gel 60 F254) and visualized by fluorescence quenching under UV light. Flash column chromatography was performed using silica gel (40-63 µm particle size) purchased from Merck.

Photochemistry

All reactions with blue light were carried out using 1 x 40 W Kessil Blue LED (Kessil A160WE Controllable LED Aquarium Light) lamp purchased from Amazon (Taiwan) with an output centered at a wavelength of approximately λ max = 456 nm. The reaction vials were cooled with a fan while being irradiated under blue light.

See the following links for more details.

https://www.kessil.com/aquarium/saltwater_A160.php

https://www.kessil.com/support/downloadfiles/aquarium/A160WE_UserManual.pdf

Spectroscopy and Instruments

^1H, ^{13}C, and DEPT NMR spectra were recorded on a 400 MHz Varian Unity Plus or Varian Mercury plus spectrometer or JEOL ECS-400. The chemical shift (δ) values are reported in ppm, and the coupling constants (J) are given in Hz. For ^1H NMR: $CDCl_3$, δ 7.26; CD_3CN, δ 1.96; CD_2Cl_2, δ 5.32; $(CD_3)_2SO$, δ 2.50; For ^{13}C NMR: $CDCl_3$, δ 77.16; 1.32; CD_2Cl_2, δ 53.84; $(CD_3)_2SO$, δ 39.52.[1] The abbreviations used are as follows: s = singlet, d = doublet, t = triplet, q = quartet, dd = doublet of doublet, ddd = doublet of doublet of doublet, dt = doublet of triplets, td = triplet of doublet, m = multiplet, br = broad; coupling constants in Hz; integration.

Starting materials

All substrates were used as received from commercial suppliers, or prepared according to published procedures, respectively, unless otherwise stated. Nickel, Copper, Iron, Ir (III) catalysts, were purchased from Alfa Aesar, and stored in an under vacuum/dark hood cabinet. Aldehydes, Alkenes, N-substituted amines were purchased from Sigma-Aldrich, Acros, TCI, or Alfa Aesar.

3. Experimental data

3.1 General procedure for the synthesis of final compounds

A) General procedure for radical cyclization (2/4)

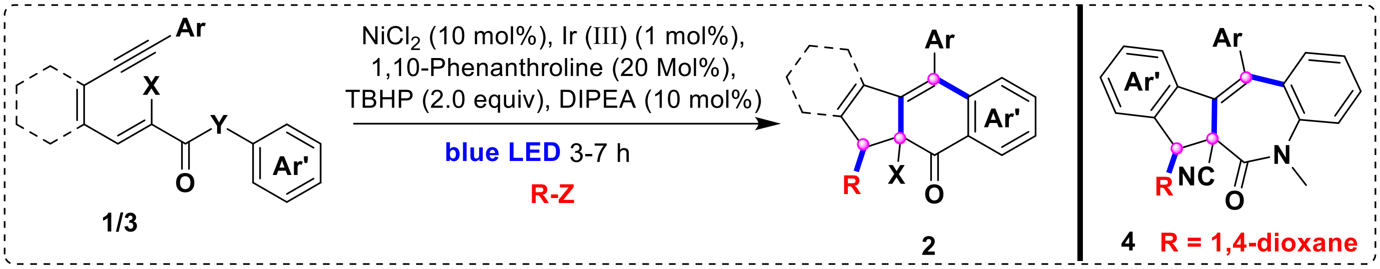

A clean vial (5 mL) equipped with a magnetic stir bar was added to **1/3** (0.2 mmol, 1.0 equiv.), 1,10-phenanthroline (0.04 mmol, 20 mol%), and $NiCl_2$ (0.02 mmol, 10 mol%). Next, 1,4-Dioxane/THF, (1,2-DCE 3.0 mL for *N*,*N*-dimethylaniline or *n*-Bu_3SnH) (3.0 mL) was added after which, aq. 70% TBHP (0.4 mmol, 2.0 equiv), DIPEA (0.02 mmol, 10 mol%) and (Ir[dF(CF_3)ppy]$_2$(dtbpy))PF_6 (0.002 mmol, 1 mol%) were added at room temperature, and then placed at a distance of approx. 3 cm from a 40 W blue LED, and the solution was stirred at room temperature under visible-light irradiation for 3-7 h. The progress of the reaction was monitored by thin layer chromatography. When the reaction was complete, water was added to quench the reaction mixture, followed by extraction with ethyl acetate (3×10 mL). Finally, the combined organic layer was dried over sodium sulfate, filtered, and concentrated under vacuum. The residue was purified by column chromatography (Hex/EA = 93:7) on silica gel to afford the corresponding **2/4** derivatives.

B) General Procedure for the Synthesis of analogous (1/3).[1]

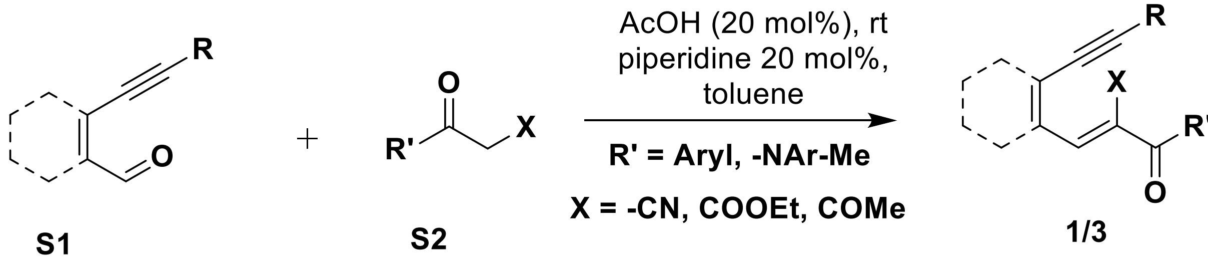

A reaction tube was charged with 3-oxo-3-arylpropanenitrile/ester/methyl/acrylamide derivatives (**S2**) (0.55 mmol, 1.0 equiv), 2-(aryl-(aryl)-alkynyl) benzaldehyde (**S1**) (0.6 mmol, 1.1 equiv.), acetic acid (0.02 mmol, 20 mol%) and pyrrolidine (0.02 mmol, 20 mol%) in 2.0 mL of toluene. The reaction suspension was stirred at room temperature and progress of reaction was monitored by TLC. Upon completion water was added to quench the reaction mixture and then extracted with the ethyl acetate (3 × 30 mL). Finally combined organic layer was dried

over sodium sulphate, filtered and concentrated in vacuum. The residue was purified by column chromatography on silica gel to afford the corresponding **1/3** derivatives.

3.2 Gram scale reaction for the synthesis of 10-oxo-5-phenyl-10,11-dihydro-10a*H*-benzo[*b*]fluorene-10*a*-carbonitrile (2r)

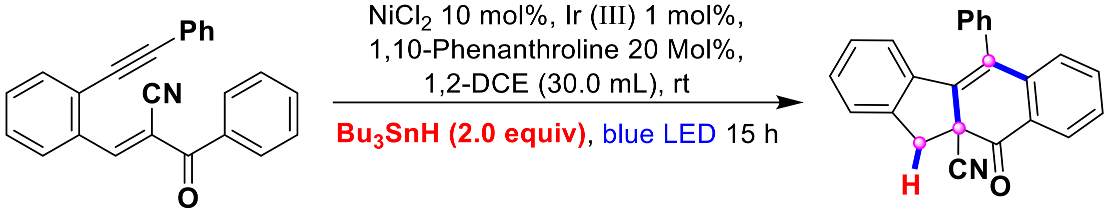

1a, 1.2 gm

2r, 72% yield

A clean vial (50 mL) equipped with a magnetic stir bar was added to **1a** (1.2 g, 3.59 mmol, 1.0 equiv.), 1,10-phenanthroline (139 mg, 0.71 mmol, 20 mol%), and $NiCl_2$ (47 mg, 0.359 mmol, 10 mol%). Next, 1,4-Dioxane/THF, (1,2-DCE 30 mL for *N*,*N*-dimethylaniline or *n*-Bu_3SnH) (2.1 g, 7.18 mmol, 2.0 equiv.) was added after which, TBHP (aq.70%, 2.0 equiv), DIPEA (10 mol%) and (Ir[dF(CF_3)ppy]$_2$(dtbpy))PF_6 (41 mg, 0.0359 mmol, 1 mol%) were added at room temperature, and then placed at a distance of approx. 3 cm from a 40 W blue LED, and the solution was stirred at room temperature under visible-light irradiation for 15 h. The progress of the reaction was monitored by thin layer chromatography. When the reaction was complete, water was added to quench the reaction mixture, followed by extraction with ethyl acetate (3×100 mL). Finally, the combined organic layer was dried over sodium sulfate, filtered, and concentrated under vacuum. The residue was purified by column chromatography (Hex/EA = 95:5) on silica gel to afford the corresponding **2r** (0.86 g, 72%).

3.3 Large scale synthesis of 7-(1,4-dioxan-2-yl)-5-methyl-6-oxo-12-phenyl-5,7-dihydrobenzo[b]indeno[1,2-e]azepine-6a(6H)-carbonitrile (4a)

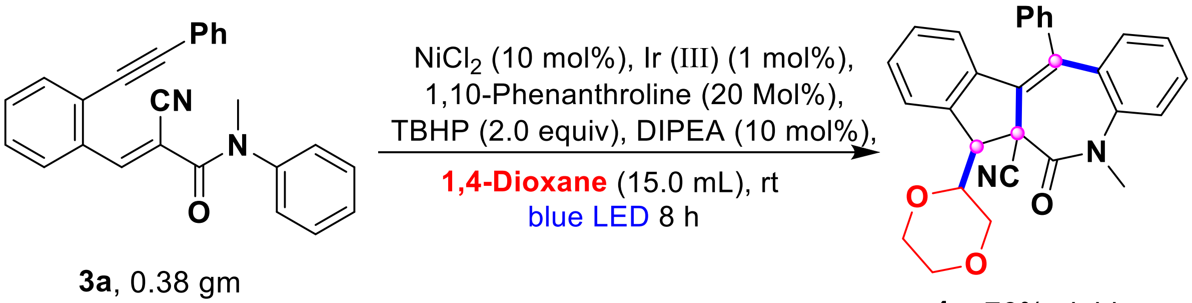

3a, 0.38 gm

4a, 76% yield

A clean vial (50 mL) equipped with a magnetic stir bar was added to **3a** (0.38 g, 1.04 mmol, 1.0 equiv.), 1,10-phenanthroline (37 mg, 0.20 mmol, 20 mol%), and $NiCl_2$ (14 mg, 0.10 mmol, 10 mol%). Next, 1,4-Dioxane, (15.0 mL) was added after which, aq.70% TBHP (268 mg, 2.08 mmol, 2.0 equiv), DIPEA (14 mg, 0.1 mmol, 10 mol%) and (Ir[dF(CF3)ppy]2(dtbpy))PF_6 (11 mg, 0.01 mmol, 1 mol%) were added at room temperature, and then placed at a distance of approx. 3 cm from a 40 W blue LED lamp, and the solution was stirred at room temperature under visible-light irradiation for 8 h. The progress of the reaction was monitored by thin layer chromatography. When the reaction was complete, water was added to quench the reaction mixture, followed by extraction with ethyl acetate (3×100 mL). Finally, the combined organic layer was dried over sodium sulfate, filtered, and concentrated under vacuum. The residue was purified by column chromatography (Hex/EA = 93:7) on silica gel to afford the corresponding **4a** as 2 separable (major/minor) products (360 mg; 76%)

4. Optimization reaction condition for radical cyclization

Table S1. Optimization of reaction conditions for radical cyclization with Bu_3Sn-H [a,b]

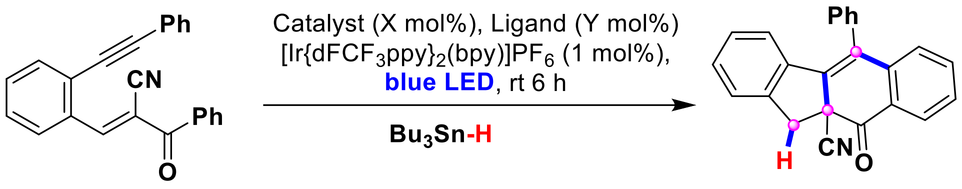

1a **2r**

Entry	Catalyst (mol%)	Ligands (mol%)	Yields
1[c]	$NiCl_2$ (10 mol%)	1,10-phenanthroline (20 mol%)	15
2[d]	$NiCl_2$ (10 mol%)	1,10-phenanthroline (20 mol%)	37
3[e]	$NiCl_2$ (10 mol%)	1,10-phenanthroline (20 mol%)	39
**4**	**$NiCl_2$ (10 mol%)**	**1,10-phenanthroline (20 mol%)**	**91**
5[f]	$NiCl_2$ (10 mol%)	1,10-phenanthroline (20 mol%)	60
6	$NiCl_2$ (10 mol%)	PPh_3 (20 mol%)	30
7	$NiCl_2$ (10 mol%)	PCy_3 (20 mol%)	43
8	$NiCl_2$ (10 mol%)	TFP (20 mol%)	32
9[g]	$NiCl_2$ (10 mol%)	1,10-phenanthroline (20 mol%)	trace
10[h]	$NiCl_2$ (10 mol%)	1,10-phenanthroline (20 mol%)	30

[a]Standard condition: **1a** (0.20 mmol), $NiCl_2$ (10 mol%), 1,10-phenanthroline (20 mol%), [Ir{dFCF$_3$ppy}$_2$(bpy)]PF_6 (1.0 mol%) in 1,2-DCE (2.0 mL) were stirred for 6 h and irradiated with blue LEDs (λ_{max} = 456 nm, latent temperature ~25°C-30°C). [b]Isolated reaction yield. [c]DIPEA (10 mol%), TBHP (70% aq., 2.0 equiv.), [d]Only TBHP (70% aq., 2.0 equiv.) was used. [e]Only DIPEA (10 mol%), was used. [f]reaction stirred at 12 h. [g]reaction was stirred under dark. [h]reaction was stirred in absence of blue LED.

5. Spectral characterization

2-cyano-*N*-methyl-*N*-phenyl-3-(2-(phenylethynyl)phenyl)acrylamide (3a): was prepared according to the general procedure B and purified by flash column chromatography (Hex/EA = 95:5) as a pale yellow solid (yield: 183 mg, 84%). m.p.= 104-106 °C; HRMS (ESI) m/z: [M+Na]+ Calcd for $C_{25}H_{18}N_2ONa$, 385.1309; Found, 385.1311. ^{1}H NMR (400 MHz, $CDCl_3$) δ 8.61 (s, 1H), 8.04 (dd, *J* = 8.0, 0.6 Hz, 1H), 7.67 – 7.57 (m, 3H), 7.46 – 7.30 (m, 8H), 7.29 – 7.23 (m, 2H), 3.48 (s, 3H); ^{13}C NMR (101 MHz, $CDCl_3$) δ 163.1, 150.8, 142.7, 133.4, 132.7, 131.7, 131.4, 129.8, 128.9, 128.5, 128.5, 128.1, 127.7, 127.1, 125.6, 122.4, 114.7, 108.8, 97.0, 86.1, 39.0.

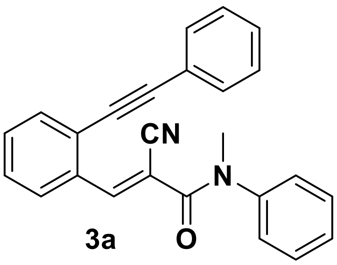

3a

2-cyano-*N*-methyl-3-(4-methyl-2-(phenylethynyl)phenyl)-*N*-phenylacrylamide (3b): was prepared according to the general procedure B and purified by flash column chromatography (Hex/EA = 95:5) as a pale yellow solid (yield: 181 mg, 80%). m.p.= 125-127 °C; HRMS (ESI) m/z: [M+Na]+ Calcd for $C_{26}H_{20}N_2ONa$, 399.1465; Found, 399.1467. ^{1}H NMR (400 MHz, $CDCl_3$) δ 8.60 (s, 1H), 8.00 (d, *J* = 8.2 Hz, 1H), 7.65 – 7.58 (m, 2H), 7.45 – 7.38 (m, 6H), 7.35 (dd, *J* = 5.0, 3.6 Hz, 2H), 7.26 – 7.12 (m, 2H), 3.47 (s, 3H), 2.36 (s, 3H); ^{13}C NMR (101 MHz, $CDCl_3$) δ 163.4, 150.8, 145.2, 142.9, 142.4, 133.2, 131.7, 129.8, 129.5, 129.1, 128.9, 128.5, 128.0, 127.6, 127.1, 122.5, 115.0, 107.4, 96.6, 86.3, 39.0, 21.4.

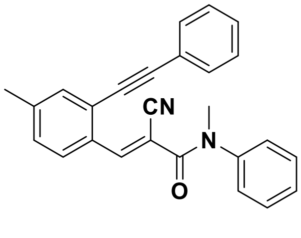

3b

2-cyano-*N*-(4-methoxyphenyl)-*N*-methyl-3-(2-(phenylethynyl)phenyl)acrylamide (3c): was prepared according to the general procedure B and purified by flash column chromatography (Hex/EA = 95:5) as a pale yellow solid (yield: 212 mg, 90%). m.p.= 125-127 °C; HRMS (ESI) m/z: [M+Na]+ Calcd for $C_{26}H_{20}N_2O_2Na$, 415.1415; Found, 415.1417. ^{1}H NMR (400 MHz, $CDCl_3$) δ 8.59 (s, 1H), 8.04 (d, *J* = 7.9 Hz, 1H), 7.61 (ddd, *J* = 8.8, 7.7, 2.6 Hz, 3H), 7.43 – 7.32 (m, 5H), 7.16 (dd, *J* = 9.5, 2.7 Hz, 2H), 6.94 – 6.85 (m, 2H), 3.80 (s, 3H), 3.42 (s, 3H); ^{13}C NMR (101 MHz, $CDCl_3$) δ 163.2, 159.2, 150.5, 135.3, 133.5, 132.6, 131.7, 131.3, 128.9, 128.5, 128.4, 127.7, 125.4, 122.4, 114.9, 114.8, 109.0, 96.9, 86.2, 55.4, 39.1.

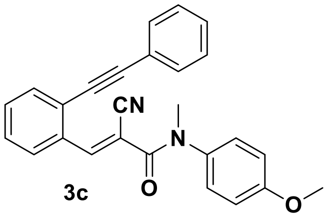

3c

2-cyano-*N*-methyl-3-(2-(phenylethynyl)phenyl)-*N*-(*p*-tolyl)acrylamide (3d): was prepared according to the general procedure B and purified by flash column chromatography (Hex/EA = 95:5) as a pale yellow solid (yield: 192 mg, 85%). m.p.= 134-135 °C; HRMS (ESI) m/z: [M+Na]+ Calcd for $C_{26}H_{20}N_2ONa$, 399.1465; Found, 399.1467. ^{1}H NMR (400 MHz, $CDCl_3$) δ 8.59 (s, 1H), 8.04 (dd, *J* = 7.9, 0.6 Hz, 1H), 7.65 – 7.58 (m, 3H), 7.44 – 7.38 (m, 4H), 7.34 (ddd, *J* = 8.0, 4.4, 0.7 Hz, 1H), 7.19 (dd, *J* = 8.6, 0.5 Hz, 2H), 7.16 – 7.10 (m, 2H), 3.44 (s, 3H), 2.36 (s, 3H); ^{13}C NMR (101 MHz, $CDCl_3$) δ 163.1, 150.6, 140.1, 138.1, 133.5, 132.6, 131.7, 131.3, 130.4, 128.9, 128.5, 128.5, 127.7, 126.9, 125.5, 122.5, 114.8, 109.0, 96.9, 86.1, 39.0, 21.1.

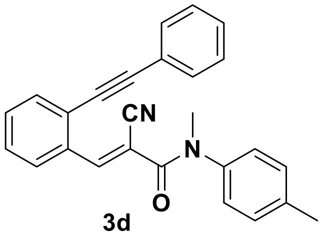

3d

2-cyano-N-(4-fluorophenyl)-N-methyl-3-(2-(phenylethynyl)phenyl)acrylamide (3e): was prepared according

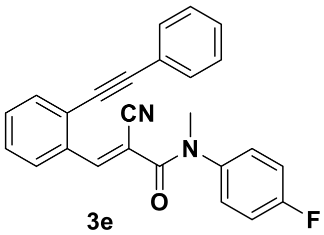

3e

to the general procedure B and purified by flash column chromatography (Hex/EA = 95:5) as a pale yellow solid (yield: 178 mg, 78%). m.p.= 126-128 °C; HRMS (ESI) m/z: [M+Na]+ Calcd for $C_{25}H_{17}N_2OFNa$, 403.1215; Found, 403.1217. ^{1}H NMR (400 MHz, $CDCl_3$) δ 8.62 (s, 1H), 8.05 (d, *J* = 7.9 Hz, 1H), 7.62 (ddd, *J* = 7.0, 4.1, 1.2 Hz, 3H), 7.46 – 7.32 (m, 5H), 7.23 (ddd, *J* = 6.8, 5.7, 3.4 Hz, 2H), 7.12 – 7.05 (m, 2H), 3.44 (s, 3H); ^{13}C NMR (101 MHz, $CDCl_3$) δ 163.1 (J_{C-F} = 3.97 Hz), 160.6 (J_{C-F} = 243.56 Hz), 151.2, 138.7, 138.7, 133.3, 132.7, 131.7, 131.5, 128.9 (J_{C-F} = 2.3 Hz), 128.9, 128.6, 128.5, 127.6, 125.6, 122.4, 116.9 (J_{C-F} = 22.5 Hz), 114.7, 108.4, 97.1, 39.2.

2-cyano-3-(2-((4-cyanophenyl)ethynyl)phenyl)-N-methyl-N-phenylacrylamide (3f): was prepared according

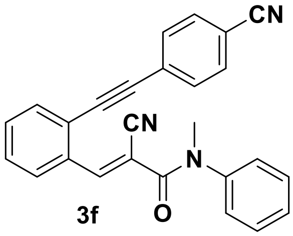

3f

to the general procedure B and purified by flash column chromatography (Hex/EA = 95:5) as a pale yellow solid (yield: 179 mg, 77%). m.p.= 157-159 °C; HRMS (ESI) m/z: [M+Na]+ Calcd for $C_{26}H_{17}N_3ONa$, 410.1262; Found, 410.1263. ^{1}H NMR (400 MHz, $CDCl_3$) δ 8.63 (s, 1H), 8.04 (d, *J* = 7.8 Hz, 1H), 7.75 – 7.67 (m, 4H), 7.62 (dd, *J* = 7.7, 1.1 Hz, 1H), 7.47 – 7.36 (m, 5H), 7.29 – 7.23 (m, 2H), 3.49 (s, 3H). ^{13}C NMR (101 MHz, $CDCl_3$) δ 162.8, 150.6, 142.6, 133.7, 132.8, 132.2, 132.2, 131.5, 129.8, 129.4, 128.3, 127.9, 127.3, 127.1, 124.4, 118.4, 114.4, 112.1, 109.3, 95.0, 90.2, 39.1.

methyl (E)-4-((2-(2-cyano-3-(methyl(phenyl)amino)-3-oxoprop-1-en-1-yl)phenyl)ethynyl)benzoate (3g):

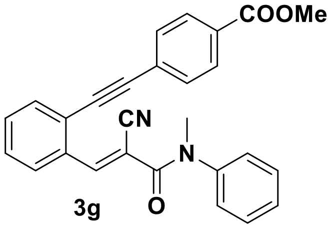

3g

was prepared according to the general procedure B and purified by flash column chromatography (Hex/EA = 95:5) as a pale yellow solid (yield: 199 mg, 79%). m.p.= 129-131 °C; HRMS (ESI) m/z: [M+Na]+ Calcd for $C_{27}H_{20}N_2O_3Na$, 443.1364; Found, 443.1366. ^{1}H NMR (400 MHz, $CDCl_3$) δ 8.61 (s, 1H), 8.11 – 8.02 (m, 3H), 7.72 – 7.67 (m, 2H), 7.62 (dd, J = 7.7, 1.1 Hz, 1H), 7.46 – 7.34 (m, 5H), 7.29 – 7.23 (m, 2H), 3.95 (s, 3H), 3.49 (s, 3H). 13C NMR (101 MHz, CDCl3) δ 166.4, 162.9, 150.6, 142.7, 133.6, 132.8, 131.6, 131.4, 130.0, 129.8, 129.6, 129.0, 128.2, 127.8, 127.1, 124.9, 114.6, 109.1, 96.0, 88.9, 52.3, 39.0.

11-(1,4-dioxan-2-yl)-10-oxo-5-phenyl-10,11-dihydro-10aH-benzo[*b*]fluorene-10a-carbonitrile (2a):

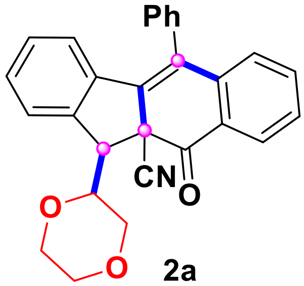

2a

A clean vial (5 mL) equipped with a magnetic stir bar was added to **1a** (67 mg, 0.2 mmol, 1.0 equiv), 1,10-phenanthroline (7 mg, 0.04 mmol, 20 mol%), and $NiCl_2$ (3 mg, 0.02, 10 mol%). Next, 1,4-Dioxane, (3.0 mL) was added after which, TBHP (aq.70%, 52 mg, 0.4 mmol, 2.0 equiv), DIPEA (3 mg, 0.02 mmol, 10 mol%) and $(Ir[dF(CF_3)ppy]_2(dtbpy))PF_6$ (2 mg, 0.002 mmol, 1 mol%) were added at room temperature, and then placed at a distance of approx. 3 cm from a 40 W blue LED lamp, and the solution was stirred at room temperature under visible-light irradiation for 4 h. The progress of the reaction was monitored by thin layer chromatography. When the reaction was complete, water was added to

quench the reaction mixture, followed by extraction with ethyl acetate (3×10 mL). Finally, the combined organic layer was dried over sodium sulfate, filtered, and concentrated under vacuum. The residue was purified by column chromatography (Hex/EA = 93:7) on silica gel to afford the corresponding **2a** as 2 separable (major/minor) products (yield: 73.83 mg, 88%, 1.5:1 dr, pale yellow solid), m.p.= 245-247 °C; HRMS (ESI) m/z: [M+H]+ Calcd for $C_{28}H_{22}NO_3$, 420.1521; Found, 420.1523. Major: ^{1}H NMR (400 MHz, $CDCl_3$) δ 7.97 (dd, *J* = 7.6, 1.2 Hz, 1H), 7.64 (d, *J* = 6.6 Hz, 2H), 7.57-7.39 (m, 5H), 7.30-7.26 (m, 1H), 6.98 (ddd, *J* = 7.3, 4.1, 1.0 Hz, 2H), 6.93-6.86 (m, 1H), 6.32 (d, *J* = 8.0 Hz, 1H), 4.46 (ddd, *J* = 10.4, 4.4, 2.6 Hz, 1H), 4.32 (dd, *J* = 11.8, 2.5 Hz, 1H), 3.96 (d, *J* = 4.4 Hz, 1H), 3.93-3.82 (m, 3H), 3.81-3.71 (m, 2H); ^{13}C NMR (101 MHz, $CDCl_3$) δ 193.8, 143.4, 140.3, 137.6, 136.4, 136.1, 135.1, 132.7, 130.6, 129.9, 129.6, 129.3, 128.8, 128.7, 128.5, 128.2, 127.9, 127.8, 127.7, 125.1, 124.6, 116.8, 74.5, 69.4, 67.6, 66.2, 53.7, 47.5.

Minor: ^{1}H NMR (597 MHz, $CDCl_3$) δ 7.97 (dd, *J* = 7.6, 1.2 Hz, 1H), 7.90 (d, *J* = 7.7 Hz, 1H), 7.63 (dd, *J* = 21.7, 7.0 Hz, 2H), 7.57 – 7.52 (m, 2H), 7.50 (d, *J* = 7.6 Hz, 1H), 7.42 (dd, *J* = 13.9, 6.4 Hz, 1H), 7.24 (d, *J* = 7.4 Hz, 1H), 6.99 (dd, *J* = 13.9, 6.5 Hz, 2H), 6.91 (d, *J* = 7.8 Hz, 1H), 6.32 (d, *J* = 7.9 Hz, 1H), 4.22 (ddd, *J* = 10.0, 7.6, 2.5 Hz, 1H), 4.07 (dd, *J* = 11.7, 2.4 Hz, 1H), 4.04 – 3.95 (m, 2H), 3.94 – 3.88 (m, 1H), 3.85 – 3.79 (m, 2H), 3.77 (d, *J* = 7.6 Hz, 1H); ^{13}C NMR (150 MHz, $CDCl_3$) δ 193.3, 144.6, 140.4, 137.3, 136.0, 135.9, 135.3, 132.4, 129.7, 128.7, 128.5, 127.9, 127.7, 127.7, 127.2, 124.3, 115.8, 109.9, 77.7, 70.6, 67.1, 66.5, 46.3.

11-(1,4-dioxan-2-yl)-2-methoxy-10-oxo-5-phenyl-10,11-dihydro-10aH-benzo[*b*]fluorene-10a-carbonitrile

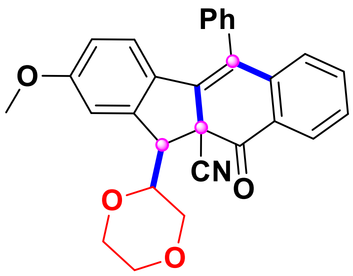

2b

(2b): A clean vial (5 mL) equipped with a magnetic stir bar was added to **1b** (73 mg, 0.2 mmol, 1.0 equiv), 1,10-phenanthroline (7 mg, 0.04 mmol, 20 mol%), and $NiCl_2$ (3 mg, 0.02 mmol, 10 mol%). Next, 1,4-Dioxane, (3.0 mL) was added after which, TBHP (aq.70%, 51.5 mg, 0.4 mmol, 2.0 equiv), DIPEA (3 mg, 0.02 mmol, 10 mol%) and (Ir[dF(CF_3)ppy]$_2$(dtbpy))PF_6 (2 mg, 0.002 mmol, 1 mol%) were added at room temperature, and then placed at a distance of approx. 3 cm from a 40 W blue LED lamp, and the solution was stirred at room temperature under visible-light irradiation for 4 h. The progress of the reaction was monitored by thin layer chromatography. The progress of the reaction was monitored by thin layer chromatography. When the reaction was complete, water was added to quench the reaction mixture, followed by extraction with ethyl acetate (3×10 mL). Finally, the combined organic layer was dried over sodium sulfate, filtered, and concentrated under vacuum. The residue was purified by column chromatography (Hex/EA = 93:7) on silica gel to afford the corresponding **2b** as 2 separable (major/minor) products (yield: 77.3 mg, 86%, 1.4:1 dr, pale yellow solid), m.p.= 282-284 °C; HRMS (ESI) m/z: [M+H]+ Calcd for $C_{29}H_{24}NO_4$, 450.1704; Found, 450.1707. Major: ^{1}H NMR (400 MHz, $CDCl_3$) δ 7.95 (dd, *J* = 7.6, 1.3 Hz, 1H), 7.63 (d, *J* = 6.0 Hz, 2H), 7.55-7.47 (m, 3H), 7.37 (td, *J* = 7.5, 1.1 Hz, 1H), 6.97 (dd, *J* = 12.3, 4.3 Hz, 2H), 6.86 (d, *J* = 7.8 Hz, 1H), 6.55 (dd, *J* = 8.8, 1.8 Hz, 1H), 6.24 (d, *J* = 8.8 Hz, 1H), 4.44 (ddd, *J* = 10.4, 4.2, 2.6 Hz, 1H), 4.31 (dd, *J* = 11.9, 2.5 Hz, 1H), 3.93-3.81 (m, 4H), 3.80 (d, *J* = 8.1 Hz, 3H), 3.78-3.71 (m, 2H);

^{13}C NMR (101 MHz, $CDCl_3$) δ 194.0, 161.0, 145.6, 140.7, 136.4, 135.1, 130.8, 130.1, 129.8, 129.3, 129.3, 128.5, 128.4, 127.9, 127.7, 127.4, 125.6, 116.9, 114.3, 114.2, 110.5, 77.3, 74.7, 69.3, 67.7, 66.3, 55.5, 47.5.

Minor: ^{1}H NMR (597 MHz, $CDCl_3$) δ 7.94 (dd, *J* = 7.6, 1.2 Hz, 1H), 7.67-7.58 (m, 2H), 7.56-7.45 (m, 4H), 7.37 (td, *J* = 7.5, 1.0 Hz, 1H), 7.00 (d, *J* = 6.9 Hz, 1H), 6.87 (d, *J* = 7.4 Hz, 1H), 6.55 (dd, *J* = 8.8, 1.9 Hz, 1H), 6.23 (d, *J* = 8.8 Hz, 1H), 4.20 (ddd, *J* = 10.3, 8.1, 2.5 Hz, 1H), 4.06 (dd, *J* = 11.7, 2.4 Hz, 1H), 4.02-3.95 (m, 2H), 3.91 (ddd, *J* = 11.5, 9.6, 4.3 Hz, 1H), 3.85-3.80 (m, 2H), 3.80-3.75 (m, 3H), 3.73 (d, *J* = 8.1 Hz, 1H); ^{13}C NMR (150 MHz, $CDCl_3$) δ 193.4, 161.1, 146.8, 140.7, 136.9, 136.2, 135.3, 130.7, 129.9, 129.7, 129.7, 128.8, 128.5, 128.4, 127.9, 127.7, 127.3, 125.3, 115.9, 114.2, 112.6, 77.8, 70.6, 67.1, 66.5, 55.4, 54.8, 46.1.

11-(1,4-dioxan-2-yl)-2-fluoro-10-oxo-5-phenyl-10,11-dihydro-10aH-benzo[*b*]fluorene-10a-carbonitrile (2c): A clean vial (5 mL) equipped with a magnetic stir bar was added to **1c** (70 mg, 0.2 mmol, 1.0 equiv), 1,10-phenanthroline (7 mg, 0.04 mmol, 20 mol%), and $NiCl_2$ (3 mg, 0.02 mmol, 10 mol%). Next, 1,4-Dioxane, (3.0 mL) was added after which, TBHP (aq.70%, 51.5 mg, 0.4 mmol, 2.0 equiv), DIPEA (3 mg, 0.02 mmol, 10 mol%) and (Ir[dF(CF_3)ppy]$_2$(dtbpy))PF_6 (2 mg, 0.002 mmol, 1 mol%) were added at room temperature, and then placed at a distance of approx. 3 cm from a 40 W blue LED lamp, and the solution was stirred at room temperature under visible-light irradiation for 4 h. The progress of the reaction was monitored by thin layer chromatography. When the reaction was complete, water was added to quench the reaction mixture, followed by extraction with ethyl acetate (3×10 mL). Finally, the combined organic layer was dried over sodium sulfate, filtered, and concentrated under vacuum. The residue was purified by column chromatography (Hex/EA = 93:7) on silica gel to afford the corresponding **2c** as 2 separable (major/minor) products (yield: 68.3 mg, 78%, 1.2:1 dr, pale yellow solid), m.p.= 265-267 °C; HRMS (ESI) m/z: [M+H]+ Calcd for $C_{28}H_{21}FNO_3$, 438.1503; Found, 438.1505. Major: ^{1}H NMR (400 MHz, $CDCl_3$) δ 7.98 (dd, *J* = 7.6, 1.1 Hz, 1H), 7.63 (dt, *J* = 14.2, 7.2 Hz, 2H), 7.58-7.47 (m, 3H), 7.41 (td, *J* = 7.5, 1.1 Hz, 1H), 7.15 (ddd, *J* = 8.7, 2.3, 1.0 Hz, 1H), 6.98 (d, *J* = 7.3 Hz, 1H), 6.89 (dd, *J* = 7.9, 0.6 Hz, 1H), 6.70 (tdd, *J* = 8.7, 2.4, 0.9 Hz, 1H), 6.27 (dd, *J* = 8.8, 5.3 Hz, 1H), 4.44 (ddd, *J* = 10.4, 4.0, 2.7 Hz, 1H), 4.30 (dd, *J* = 11.9, 2.6 Hz, 1H), 3.93 (d, *J* = 3.2 Hz, 1H), 3.91-3.69 (m, 5H); ^{13}C NMR (101 MHz, $CDCl_3$) δ 193.6, 164.7 (J_{C-F} = 250 Hz), 146.1, (J_{C-F} = 8.6 Hz), 140.2, 136.4, 135.9, 135.5, 135.3, 132.5 (J_{C-F} = 2.54 Hz), 132.2 (J_{C-F} = 2.68 Hz), 130.5 (J_{C-F} = 55.59 Hz), 129.4, 128.8, 128.6, 128.5, 128.2, 127.8 (J_{C-F} = 4.48 Hz), 126.1 (J_{C-F} = 8.85 Hz), 116.6, 115.8 (J_{C-F} = 22.75 Hz), 112.4, (J_{C-F} = 23.34 Hz), 74.1, 69.1, 67.7, 66.2, 53.8, 47.6, 47.5.

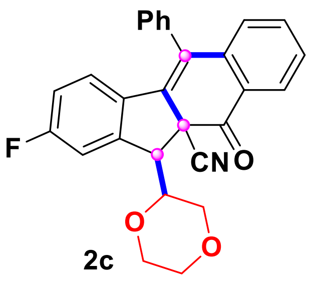

2c

Minor: ^{1}H NMR (597 MHz, $CDCl_3$) δ 7.97 (dd, *J* = 7.6, 1.0 Hz, 1H), 7.69-7.63 (m, 2H), 7.59 (d, *J* = 7.3 Hz, 1H), 7.57-7.52 (m, 2H), 7.50 (d, *J* = 7.4 Hz, 1H), 7.41 (td, *J* = 7.5, 1.1 Hz, 1H), 7.00 (d, *J* = 7.7 Hz, 1H), 6.90 (dd, *J* = 7.9, 0.6 Hz, 1H), 6.73-6.65 (m, 1H), 6.26 (dd, *J* = 8.8, 5.4 Hz, 1H), 4.18 (ddd, *J* = 10.5, 8.3, 2.4 Hz, 1H), 4.05 (dd, *J* = 11.7, 2.3 Hz, 1H), 3.97 (dd, *J* = 11.7, 10.0 Hz, 2H), 3.94-3.88 (m, 1H), 3.82 (dd, *J* = 10.2, 2.6 Hz, 2H), 3.74 (d, *J* = 8.2 Hz, 1H); ^{13}C NMR (150 MHz, $CDCl_3$) δ 192.9, 164.3, (J_{C-F} = 250.30 Hz), 147.2, (J_{C-F} = 9.27 Hz),

140.2, 136.0, 135.7, 135.4, 132.0 ($J_{C\text{-}F}$ = 2.55 Hz), 131.7 ($J_{C\text{-}F}$ = 2.52 Hz), 130.4, 130.1, 129.5, 128.8, 128.5, 128.4, 128.3, 127.8, 127.6, 125.7, 125.6, 115.7 ($J_{C\text{-}F}$ = 23.05 Hz), 115.5, 114.7, ($J_{C\text{-}F}$ = 21.40 Hz), 77.6, 70.6, 67.1, 66.5, 54.7, 46.1, 46.1.

2-chloro-11-(1,4-dioxan-2-yl)-10-oxo-5-phenyl-10,11-dihydro-10aH-benzo[*b*]fluorene-10a-carbonitrile

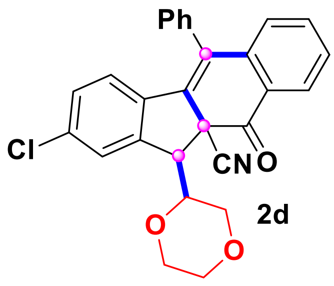

2d

(2d): A clean vial (5 mL) equipped with a magnetic stir bar was added to **1d** (74 mg, 0.2 mmol, 1.0 equiv), 1,10-phenanthroline (7 mg, 0.04 mmol, 20 mol%), and $NiCl_2$ (3 mg, 0.02 mmol, 10 mol%). Next, 1,4-Dioxane, (3.0 mL) was added after which, TBHP (aq.70%, 51.5 mg, 0.4 mmol, 2.0 equiv), DIPEA (3 mg, 0.02 mmol, 10 mol%) and (Ir[dF(CF_3)ppy]$_2$(dtbpy))PF_6 (2 mg, 0.002 mmol, 1 mol%) were added at room temperature, and then placed at a distance of approx. 3 cm from a 40 W blue LED lamp, and the solution was stirred at room temperature under visible-light irradiation for 4 h. The progress of the reaction was monitored by thin layer chromatography. When the reaction was complete, water was added to quench the reaction mixture, followed by extraction with ethyl acetate (3×10 mL). Finally, the combined organic layer was dried over sodium sulfate, filtered, and concentrated under vacuum. The residue was purified by column chromatography (Hex/EA = 93:7) on silica gel to afford the corresponding **2d** as 2 separable (major/minor) products (yield: 73 mg, 80%, 1.3:1, yellow solid), m.p.= 266-268 °C; HRMS (ESI) m/z: [M+H]+ Calcd for $C_{28}H_{21}ClNO_3$, 454.1204; Found, 454.1204. Major: ^{1}H NMR (597 MHz, $CDCl_3$) δ 7.98 (dd, *J* = 7.6, 1.4 Hz, 1H), 7.64 (d, *J* = 7.3 Hz, 1H), 7.60 (d, *J* = 7.2 Hz, 1H), 7.57 – 7.52 (m, 2H), 7.49 (t, *J* = 7.5 Hz, 1H), 7.45-7.39 (m, 2H), 7.01-6.93 (m, 2H), 6.90 (d, *J* = 7.9 Hz, 1H), 6.21 (d, *J* = 8.5 Hz, 1H), 4.44 (ddd, *J* = 10.4, 4.1, 2.7 Hz, 1H), 4.29 (dd, *J* = 11.8, 2.5 Hz, 1H), 3.95-3.82 (m, 4H), 3.82-3.72 (m, 2H); ^{13}C NMR (150 MHz, $CDCl_3$) δ 193.5, 145.2, 140.0, 136.4, 135.8, 135.7, 135.3, 134.9, 133.2, 130.4, 130.0, 129.4, 128.87, 128.7, 128.7, 128.5, 128.1, 127.8, 127.8, 125.4, 125.3, 116.5, 74.1, 69.2, 67.7, 66.2, 53.6, 47.5.

Minor: ^{1}H NMR (400 MHz, $CDCl_3$) δ 7.97 (dd, *J* = 7.6, 1.3 Hz, 1H), 7.94-7.89 (m, 1H), 7.65 (d, *J* = 7.3 Hz, 1H), 7.62-7.47 (m, 4H), 7.43 (td, *J* = 7.5, 1.1 Hz, 1H), 7.03-6.94 (m, 2H), 6.91 (d, *J* = 7.9 Hz, 1H), 6.20 (d, *J* = 8.5 Hz, 1H), 4.23-4.15 (m, 1H), 4.08-3.89 (m, 4H), 3.86-3.79 (m, 2H), 3.73 (d, *J* = 8.1 Hz, 1H); ^{13}C NMR (101 MHz, $CDCl_3$) δ 192.9, 146.2, 140.1, 135.7, 135.6, 135.4, 134.4, 132.8, 130.3, 130.1, 129.5, 128.9, 128.7, 128.5, 128.1, 127.9, 127.8, 127.5, 125.0, 115.5, 77.5, 70.5, 67.2, 66.5, 46.1.

11-(1,4-dioxan-2-yl)-7-methoxy-10-oxo-5-phenyl-10,11-dihydro-10a*H*-benzo[*b*]fluorene-10a-carbonitrile (2e): A clean vial (5 mL) equipped with a magnetic stir bar was added to **1e** (73 mg, 0.2 mmol, 1.0 equiv), 1,10-phenanthroline (7 mg, 0.04 mmol, 20 mol%), and $NiCl_2$ (3 mg, 0.02 mmol, 10 mol%). Next, 1,4-Dioxane, (3.0 mL) was added after which, TBHP (aq.70%, 51.5 mg, 0.4 mmol, 2.0 equiv), DIPEA (3 mg, 0.02 mmol, 10 mol%) and (Ir[dF(CF_3)ppy]$_2$(dtbpy))PF_6 (2 mg, 0.002 mmol, 1 mol%) were added at room temperature, and then placed at a distance of approx. 3 cm from a 40 W blue LED lamp, and the solution was stirred at room temperature under visible-light irradiation for 4 h. The progress of the reaction was monitored by thin layer chromatography. When the reaction was complete, water was added to quench the reaction mixture, followed by extraction with ethyl acetate (3×10 mL). Finally, the combined organic layer was dried over sodium sulfate, filtered, and concentrated under vacuum. The residue was purified by column chromatography (Hex/EA = 93:7) on silica gel to afford the corresponding **2e** as 2 separable (major/minor) products (yield: 81 mg, 90%, 1.6:1, yellow solid), m.p.= 280-282 °C; HRMS (ESI) m/z: [M+H]+ Calcd for $C_{29}H_{24}NO_4$, 450.1704; Found, 450.1707. Major: ^{1}H NMR (597 MHz, $CDCl_3$) δ 7.98 (d, *J* = 8.5 Hz, 1H), 7.67-7.59 (m, 2H), 7.55-7.51 (m, 1H), 7.50-7.43 (m, 2H), 7.28-7.25 (m, 1H), 7.01-6.94 (m, 2H), 6.89 (dd, *J* = 8.6, 2.5 Hz, 1H), 6.36 (d, *J* = 2.4 Hz, 1H), 6.30 (d, *J* = 8.0 Hz, 1H), 4.48 (ddd, *J* = 10.4, 4.1, 2.6 Hz, 1H), 4.31 (dd, *J* = 11.9, 2.6 Hz, 1H), 3.95-3.89 (m, 2H), 3.86 (ddd, *J* = 21.5, 9.6, 6.5 Hz, 2H), 3.81-3.75 (m, 2H), 3.75 (s, 3H); ^{13}C NMR (150 MHz, $CDCl_3$) δ 191.9, 165.3, 160.9, 143.7, 142.8, 138.7, 136.4, 136.1, 132.4, 130.6, 130.5, 130.3, 129.9, 129.8, 129.6, 129.3, 128.7, 128.3, 127.9, 127.9, 125.5, 125.2, 124.6, 124.4, 122.0, 117.2, 113.8, 113.2, 74.6, 72.7, 69.3, 68.9, 67.7, 66.2, 63.1, 55.5, 53.4, 47.8, 45.5, 30.3.

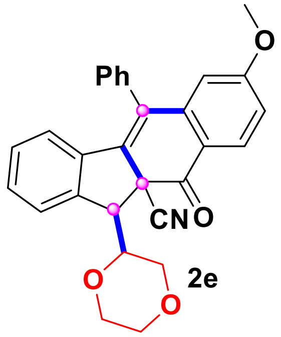

Minor: ^{1}H NMR (597 MHz, $CDCl_3$) δ 7.98 (d, *J* = 8.6 Hz, 1H), 7.89 (d, *J* = 7.7 Hz, 1H), 7.62 (dd, *J* = 21.4, 7.3 Hz, 2H), 7.56-7.45 (m, 2H), 7.24 (d, *J* = 7.2 Hz, 1H), 6.98 (dd, *J* = 16.1, 8.3 Hz, 2H), 6.89 (dd, *J* = 8.6, 2.4 Hz, 1H), 6.36 (d, *J* = 2.4 Hz, 1H), 6.30 (d, *J* = 8.0 Hz, 1H), 4.23 (td, *J* = 7.4, 3.7 Hz, 1H), 4.09 (dd, *J* = 11.7, 2.3 Hz, 1H), 4.02 (dd, *J* = 11.6, 10.1 Hz, 1H), 3.93 (dt, *J* = 9.3, 7.6 Hz, 2H), 3.85-3.79 (m, 2H), 3.75 (s, 3H), 3.72 (d, *J* = 7.4 Hz, 1H); ^{13}C NMR (150 MHz, $CDCl_3$) δ 191.3, 165.4, 144.7, 142.8, 138.3, 136.0, 135.9, 132.0, 130.4, 130.4, 129.9, 129.7, 129.3, 128.7, 128.4, 127.9, 127.2, 124.3, 121.9, 116.2, 113.6, 113.2, 77.8, 70.7, 67.1, 66.5, 55.5, 54.3, 46.5.

11-(1,4-dioxan-2-yl)-7-methyl-10-oxo-5-phenyl-10,11-dihydro-10aH-benzo[*b*]fluorene-10a-carbonitrile (2f): A clean vial (5 mL) equipped with a magnetic stir bar was added to **5e** (69 mg, 0.2 mmol, 1.0 equiv), 1,10-phenanthroline (7 mg, 0.04 mmol, 20 mol%), and $NiCl_2$ (3 mg, 0.02 mmol, 10 mol%). Next, 1,4-Dioxane, (3.0 mL) was added after which, TBHP (aq.70%, 51.5 mg, 0.4 mmol, 2.0 equiv), DIPEA (3 mg, 0.02 mmol, 10 mol%) and (Ir[dF(CF_3)ppy]$_2$(dtbpy))PF_6 (2 mg, 0.002 mmol, 1 mol%) were added at room temperature, and then placed at a distance of approx. 3 cm from a 40 W blue LED lamp,

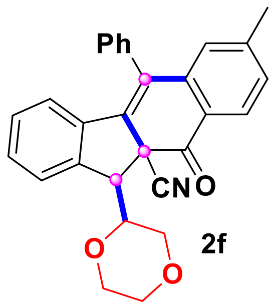

and the solution was stirred at room temperature under visible-light irradiation for 4 h. The progress of the reaction was monitored by thin layer chromatography. When the reaction was complete, water was added to quench the reaction mixture, followed by extraction with ethyl acetate (3×10 mL). Finally, the combined organic layer was dried over sodium sulfate, filtered, and concentrated under vacuum. The residue was purified by column chromatography (Hex/EA = 93:7) on silica gel to afford the corresponding **2f** as 2 separable (major/minor) products (yield: 75.4 mg, 87%, 1.5:1, pale yellow solid), m.p.= 212-214 °C; HRMS (ESI) m/z: [M+H]+ Calcd for $C_{29}H_{24}NO_3$, 434.1750; Found, 434.1749. Major: ^{1}H NMR (597 MHz, $CDCl_3$) δ 7.88 (d, *J* = 7.8 Hz, 1H), 7.63 (dd, *J* = 16.7, 7.3 Hz, 2H), 7.57-7.53 (m, 1H), 7.49 (d, *J* = 7.5 Hz, 1H), 7.44 (d, *J* = 7.7 Hz, 1H), 7.27-7.24 (m, 1H), 7.21 (dd, *J* = 7.8, 0.7 Hz, 1H), 6.97 (t, *J* = 7.7 Hz, 2H), 6.67 (s, 1H), 6.28 (d, *J* = 8.0 Hz, 1H), 4.46 (ddd, *J* = 10.4, 4.3, 2.6 Hz, 1H), 4.31 (dd, *J* = 11.8, 2.5 Hz, 1H), 3.94 (d, *J* = 4.2 Hz, 1H), 3.92-3.81 (m, 3H), 3.76 (ddd, *J* = 16.9, 12.7, 10.0 Hz, 2H), 2.30 (s, 3H); ^{13}C NMR (150 MHz, $CDCl_3$) δ 193.4, 146.4, 143.5, 140.3, 137.9, 136.5, 136.2, 132.8, 130.6, 129.8, 129.5, 129.3, 129.3, 128.6, 128.3, 128.3, 127.9, 127.9, 126.5, 125.1, 124.5, 117.0, 74.5, 69.4, 67.6, 66.2, 53.6, 47.6, 22.0.

Minor: ^{1}H NMR (597 MHz, $CDCl_3$) δ 7.92-7.86 (m, 2H), 7.69-7.58 (m, 2H), 7.53 (ddt, *J* = 32.8, 14.9, 4.3 Hz, 2H), 7.25-7.20 (m, 2H), 6.98 (ddd, *J* = 8.3, 5.3, 4.4 Hz, 2H), 6.68 (s, 1H), 6.27 (d, *J* = 8.0 Hz, 1H), 4.22 (ddd, *J* = 10.0, 7.6, 2.5 Hz, 1H), 4.07 (dd, *J* = 11.7, 2.4 Hz, 1H), 4.01 (dd, *J* = 11.7, 10.0 Hz, 1H), 3.96 (d, *J* = 10.8 Hz, 1H), 3.93 – 3.87 (m, 1H), 3.84-3.79 (m, 2H), 3.74 (d, *J* = 7.5 Hz, 1H), 2.34-2.27 (m, 3H); ^{13}C NMR (150 MHz, $CDCl_3$) δ 192.8, 146.6, 144.6, 140.4, 137.4, 136.1, 136.1, 132.3, 130.5, 129.9, 129.6, 129.3, 128.6, 128.3, 128.2, 127.9, 127.9, 127.2, 126.3, 124.3, 116.1, 77.8, 70.6, 67.1, 66.5, 54.4, 46.4, 22.1.

11-(1,4-dioxan-2-yl)-8-methyl-10-oxo-5-phenyl-10,11-dihydro-10a*H*-benzo[*b*]fluorene-10a-carbonitrile (2g): A clean vial (5 mL) equipped with a magnetic stir bar was added to **1g** (69 mg, 0.2 mmol, 1.0 equiv), 1,10-

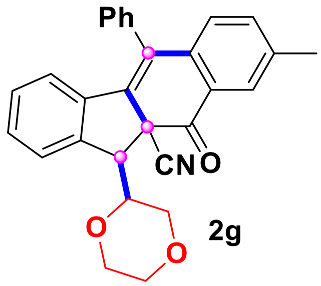

2g

phenanthroline (7 mg, 0.04 mmol, 20 mol%), and $NiCl_2$ (3 mg, 0.02 mmol, 10 mol%). Next, 1,4-Dioxane, (3.0 mL) was added after which, TBHP (aq.70%, 51.5 mg, 0.4 mmol, 2.0 equiv.), DIPEA (3 mg, 0.02 mmol, 10 mol%) and $(Ir[dF(CF_3)ppy]_2(dtbpy))PF_6$ (2 mg, 0.002 mmol, 1 mol%) were added at room temperature, and then placed at a distance of approx. 3 cm from a 40 W blue LED lamp, and the solution was stirred at room temperature under visible-light irradiation for 4 h. The progress of the reaction was monitored by thin layer chromatography. When the reaction was complete, water was added to quench the reaction mixture, followed by extraction with ethyl acetate (3×10 mL). Finally, the combined organic layer was dried over sodium sulfate, filtered, and concentrated under vacuum. The residue was purified by column chromatography (Hex/EA = 93:7) on silica gel to afford the corresponding **2g** as 2 separable (major/minor) products (yield: 73 mg, 84%, 1.1:1, pale yellow solid), m.p.= 192-194 °C; HRMS (ESI) m/z: [M+H]+ Calcd for $C_{29}H_{24}NO_3$, 434.1750; Found, 434.1749. Major: ^{1}H NMR (597 MHz, $CDCl_3$) δ 7.97 (ddd, *J* = 7.6, 1.4, 0.4 Hz, 1H), 7.64 (d, *J* = 10.3 Hz, 2H), 7.56-7.47 (m, 3H), 7.40 (td, *J* = 7.5, 1.1 Hz, 1H), 7.31 (d, *J* =

7.9 Hz, 1H), 7.08 (dd, *J* = 7.9, 0.6 Hz, 1H), 6.98 (d, *J* = 7.5 Hz, 1H), 6.91 (dd, *J* = 7.9, 0.6 Hz, 1H), 6.07 (s, 1H), 4.41 (ddd, *J* = 10.4, 4.6, 2.6 Hz, 1H), 4.31 (dd, *J* = 11.8, 2.5 Hz, 1H), 3.94 – 3.86 (m, 3H), 3.86 – 3.82 (m, 1H), 3.82 – 3.77 (m, 1H), 3.76-3.72 (m, 1H), 2.05 (d, *J* = 6.8 Hz, 3H); ^{13}C NMR (150 MHz, $CDCl_3$) δ 193.9, 140.7, 140.3, 137.8, 137.6, 136.5, 136.3, 135.1, 132.4, 130.6, 129.8, 129.2, 128.8, 128.6, 128.4, 128.3, 127.7, 125.1, 124.7, 116.9, 74.7, 69.5, 67.6, 66.2, 54.0, 47.3, 21.3.

Minor: ^{1}H NMR (597 MHz, $CDCl_3$) δ 7.97 (dd, *J* = 7.6, 1.3 Hz, 1H), 7.76 (d, *J* = 8.0 Hz, 1H), 7.63 (dd, *J* = 22.9, 7.3 Hz, 2H), 7.53 (ddd, *J* = 26.4, 13.0, 6.7 Hz, 3H), 7.40 (t, *J* = 7.5 Hz, 1H), 7.06 (d, *J* = 7.9 Hz, 1H), 7.00 (d, *J* = 7.4 Hz, 1H), 6.92 (d, *J* = 7.9 Hz, 1H), 6.06 (s, 1H), 4.19 (ddd, *J* = 10.0, 7.6, 2.5 Hz, 1H), 4.06 (dd, *J* = 11.7, 2.4 Hz, 1H), 4.02 – 3.93 (m, 2H), 3.93-3.87 (m, 1H), 3.84-3.79 (m, 2H), 3.72 (d, *J* = 7.5 Hz, 1H), 2.04 (s, 3H); ^{13}C NMR (150 MHz, $CDCl_3$) δ 193.3, 141.9, 140.4, 137.6, 137.4, 136.1, 136.1, 135.3, 131.9, 130.8, 130.5, 129.8, 129.2, 128.6, 128.6, 128.4, 128.3, 127.7, 127.6, 126.8, 124.8, 115.9, 77.8, 70.6, 67.1, 66.5, 54.6, 46.0, 21.3.

11-(1,4-dioxan-2-yl)-7-fluoro-10-oxo-5-phenyl-10,11-dihydro-10a*H*-benzo[*b*]fluorene-10a-carbonitrile

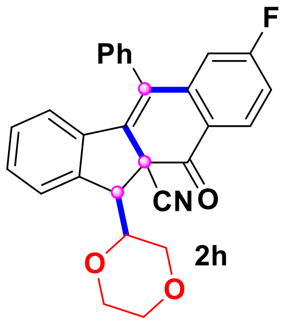

(2h): A clean vial (5 mL) equipped with a magnetic stir bar was added to **1h** (70 mg, 0.2 mmol, 1.0 equiv), 1,10-phenanthroline (7 mg, 0.04 mmol, 20 mol%), and $NiCl_2$ (3 mg, 0.02 mmol, 10 mol%). Next, 1,4-Dioxane, (3.0 mL) was added after which, TBHP (aq.70%, 51.5 mg, 0.4 mmol, 2.0 equiv), DIPEA (3 mg, 0.02 mmol, 10 mol%) and (Ir[dF(CF_3)ppy]$_2$(dtbpy))PF_6 (2 mg, 0.002 mmol, 1 mol%) were added at room temperature, and then placed at a distance of approx. 3 cm from a 40 W blue LED lamp, and the solution was stirred at room temperature under visible-light irradiation for 4 h. The progress of the reaction was monitored by thin layer chromatography. When the reaction was complete, water was added to quench the reaction mixture, followed by extraction with ethyl acetate (3×10 mL). Finally, the combined organic layer was dried over sodium sulfate, filtered, and concentrated under vacuum. The residue was purified by column chromatography (Hex/EA = 93:7) on silica gel to afford the corresponding **2h** as 2 separable (major/minor) products (yield: 66.5 mg, 76%, 1.2:1 dr, pale yellow solid), m.p.= 273-274 °C; HRMS (ESI) m/z: [M+H]+ Calcd for $C_{28}H_{21}FNO_3$, 438.1503; Found, 438.1505. Major: ^{1}H NMR (400 MHz, $CDCl_3$) δ 8.01 (dd, *J* = 8.5, 5.8 Hz, 1H), 7.68-7.49 (m, 4H), 7.47-7.43 (m, 1H), 7.29 (td, *J* = 7.6, 1.0 Hz, 1H), 7.12-7.05 (m, 1H), 7.00 (t, *J* = 7.7 Hz, 2H), 6.56 (dd, *J* = 10.0, 2.4 Hz, 1H), 6.33 (d, *J* = 8.0 Hz, 1H), 4.45 (ddd, *J* = 10.3, 4.4, 2.6 Hz, 1H), 4.31 (dd, *J* = 11.8, 2.5 Hz, 1H), 3.98-3.69 (m, 6H); ^{13}C NMR (101 MHz, $CDCl_3$) δ 192.1, 168.4 (J_{C-F} = 254.93 Hz), 143.8, 143.6 (J_{C-F} = 9.18 Hz), 139.4, 136.1, 135.5, 131.7, 130.7, 130.6 (J_{C-F} = 9.98 Hz), 130.0, (J_{C-F} = 105.62 Hz), 129.1, 128.2, 128.1, 125.1, 124.7, 116.6, 115.7 (J_{C-F} = 22.55 Hz), 114.8 (J_{C-F} = 28.06 Hz), 74.4, 69.4, 67.6, 66.2, 53.7, 47.6.

Minor: ^{1}H NMR (400 MHz, $CDCl_3$) δ 8.01 (dd, *J* = 8.5, 5.8 Hz, 1H), 7.91 (d, *J* = 7.9 Hz, 1H), 7.67 (t, *J* = 7.1 Hz, 1H), 7.63-7.49 (m, 3H), 7.28 (d, *J* = 7.9 Hz, 1H), 7.08 (td, *J* = 8.2, 2.4 Hz, 1H), 7.00 (dd, *J* = 10.0, 5.1 Hz, 2H),

6.57 (dd, J = 10.0, 2.4 Hz, 1H), 6.32 (d, J = 8.0 Hz, 1H), 4.22 (ddd, J = 10.0, 7.5, 2.7 Hz, 1H), 4.09-3.88 (m, 4H), 3.86-3.79 (m, 2H), 3.75 (d, J = 7.4 Hz, 1H); ^{13}C NMR (101 MHz, $CDCl_3$) δ 191.5, 168.9 (J_{C-F} = 257.46 Hz), 144.8, 143.7, 138.9, 135.6, 135.3, 130.8 (J_{C-F} = 9.98 Hz), 130.3, 130.2, 130.1, 129.6, 129.0, 128.2, 128.1, 127.3, 124.9, 124.5, 115.8 (J_{C-F} = 10.41 Hz), 115.5, 114.7 (J_{C-F} = 24.2 Hz), 77.6, 70.5, 67.1, 66.5, 54.4, 46.4.

7-chloro-11-(1,4-dioxan-2-yl)-10-oxo-5-phenyl-10,11-dihydro-10aH-benzo[*b*]fluorene-10a-carbonitrile

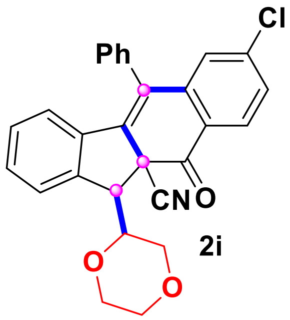

2i

(2i): A clean vial (5 mL) equipped with a magnetic stir bar was added to **1i** (74 mg, 0.2 mmol, 1.0 equiv), 1,10-phenanthroline (7 mg, 0.04 mmol, 20 mol%), and $NiCl_2$ (3 mg, 0.02 mmol, 10 mol%). Next, 1,4-Dioxane, (3.0 mL) was added after which, TBHP (aq.70%, 51.5 mg, 0.4 mmol, 2.0 equiv), DIPEA (3 mg, 0.02 mmol, 10 mol%) and (Ir[dF(CF_3)ppy]$_2$(dtbpy))PF_6 (2 mg, 0.002 mmol, 1 mol%) were added at room temperature, and then placed at a distance of approx. 3 cm from a 40 W blue LED, and the solution was stirred at room temperature under visible-light irradiation for 4 h. The progress of the reaction was monitored by thin layer chromatography. When the reaction was complete, water was added to quench the reaction mixture, followed by extraction with ethyl acetate (3×10 mL). Finally, the combined organic layer was dried over sodium sulfate, filtered, and concentrated under vacuum. The residue was purified by column chromatography (Hex/EA = 93:7) on silica gel to afford the corresponding **2i** as 2 separable (major/minor) products (yield: 68 mg, 75%, 1.3:1 dr, pale yellow solid), m.p.= 251-253 °C; HRMS (ESI) m/z: [M+H]+ Calcd for $C_{28}H_{21}ClNO_3$, 454.1212; Found, 454.1212. Major: ^{1}H NMR (597 MHz, $CDCl_3$) δ 7.91 (d, J = 8.2 Hz, 1H), 7.67 (t, J = 7.3 Hz, 1H), 7.61 (d, J = 7.4 Hz, 1H), 7.57 (tt, J = 7.5, 1.3 Hz, 1H), 7.51 (t, J = 7.4 Hz, 1H), 7.44 (d, J = 7.8 Hz, 1H), 7.37 (dd, J = 8.2, 2.0 Hz, 1H), 7.31-7.27 (m, 1H), 6.99 (t, J = 7.7 Hz, 2H), 6.85 (d, J = 1.9 Hz, 1H), 6.30 (d, J = 8.0 Hz, 1H), 4.44 (ddd, J = 10.4, 4.5, 2.6 Hz, 1H), 4.31 (dd, J = 11.8, 2.5 Hz, 1H), 3.95 (d, J = 4.5 Hz, 1H), 3.90-3.81 (m, 3H), 3.77 (ddd, J = 15.6, 11.3, 5.5 Hz, 2H); ^{13}C NMR (150 MHz, $CDCl_3$) δ 192.6, 143.7, 141.9, 141.9, 139.4, 136.1, 135.4, 131.6, 130.5, 130.1, 130.0, 129.6, 129.1, 129.0, 128.6, 128.2, 128.1, 127.6, 127.0, 125.1, 124.8, 116.5, 74.5, 69.4, 67.6, 66.2, 53.8, 47.5.

Minor: ^{1}H NMR (597 MHz, $CDCl_3$) δ 7.91 (td, J = 8.1, 0.5 Hz, 2H), 7.68 (t, J = 7.4 Hz, 1H), 7.57 (td, J = 7.5, 3.8 Hz, 2H), 7.51 (t, J = 7.4 Hz, 1H), 7.38 (dd, J = 8.2, 2.0 Hz, 1H), 7.28-7.26 (m, 1H), 7.00 (ddd, J = 8.1, 4.6, 3.7 Hz, 2H), 6.86 (d, J = 1.8 Hz, 1H), 6.29 (d, J = 8.0 Hz, 1H), 4.20 (dd, J = 7.5, 2.5 Hz, 1H), 4.08-3.94 (m, 3H), 3.90 (d, J = 4.1 Hz, 1H), 3.85-3.78 (m, 2H), 3.75 (d, J = 7.5 Hz, 1H); ^{13}C NMR (150 MHz, $CDCl_3$) δ 191.9, 144.8, 142.2, 142.0, 138.9, 135.7, 135.2, 131.2, 130.4, 130.2, 129.6, 129.2, 129.0, 128.6, 128.2, 128.1, 127.6, 127.3, 126.8, 124.5, 115.6, 77.6, 70.5, 67.1, 66.5, 54.4, 46.3.

11-(1,4-dioxan-2-yl)-10-oxo-5-(*p*-tolyl)-10,11-dihydro-10a*H*-benzo[*b*]fluorene-10*a*-carbonitrile (2j): A

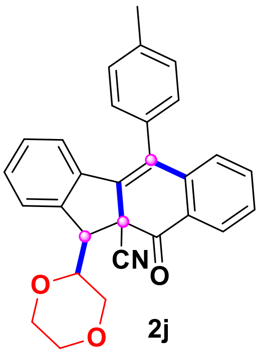

2j

clean vial (5 mL) equipped with a magnetic stir bar was added to **1j** (69 mg, 0.2 mmol, 1.0 equiv), 1,10-phenanthroline (7 mg, 0.04 mmol, 20 mol%), and $NiCl_2$ (3 mg, 0.02 mmol, 10 mol%). Next, 1,4-Dioxane, (3.0 mL) was added after which, TBHP (aq.70%, 51.5 mg, 0.4 mmol, 2.0 equiv), DIPEA (3 mg, 0.02 mmol, 10 mol%) and $(Ir[dF(CF_3)ppy]_2(dtbpy))PF_6$ (2 mg, 0.002 mmol, 1 mol%) were added at room temperature, and then placed at a distance of approx. 3 cm from a 40 W blue LED, and the solution was stirred at room temperature under visible-light irradiation for 4 h. The progress of the reaction was monitored by thin layer chromatography. When the reaction was complete, water was added to quench the reaction mixture, followed by extraction with ethyl acetate (3×10 mL). Finally, the combined organic layer was dried over sodium sulfate, filtered, and concentrated under vacuum. The residue was purified by column chromatography (Hex/EA = 93:7) on silica gel to afford the corresponding **2j** as 2 separable (major/minor) products (yield: 71 mg, 82%, 1.5:1, pale yellow solid), m.p.= 234-236 °C; HRMS (ESI) m/z: [M+H]+ Calcd for $C_{29}H_{24}NO_3$, 434.1750; Found, 434.1749. Major: ^{1}H NMR (400 MHz, $CDCl_3$) δ 7.96 (dd, *J* = 7.6, 1.4 Hz, 1H), 7.56-7.48 (m, 2H), 7.41 (ddd, *J* = 10.2, 8.5, 4.7 Hz, 3H), 7.32-7.26 (m, 2H), 7.01 (t, *J* = 7.7 Hz, 1H), 6.91 (d, *J* = 7.9 Hz, 1H), 6.86 (d, *J* = 7.4 Hz, 1H), 6.41 (d, *J* = 8.0 Hz, 1H), 4.45 (ddd, *J* = 10.4, 4.4, 2.6 Hz, 1H), 4.32 (dd, *J* = 11.8, 2.5 Hz, 1H), 3.95 (d, *J* = 4.4 Hz, 1H), 3.93-3.70 (m, 5H), 2.50 (s, 3H); ^{13}C NMR (101 MHz, $CDCl_3$) δ 193.9, 143.3, 140.5, 138.5, 137.5, 136.5, 135.1, 133.0, 132.8, 130.5, 130.5, 130.0, 129.5, 128.8, 128.4, 128.0, 127.9, 127.8, 127.6, 125.0, 124.6, 116.8, 74.5, 69.4, 67.6, 66.2, 53.7, 47.5, 21.4.

Minor: ^{1}H NMR (400 MHz, $CDCl_3$) δ 7.96 (dd, *J* = 7.6, 1.3 Hz, 1H), 7.90 (d, *J* = 7.9 Hz, 1H), 7.58-7.44 (m, 3H), 7.40 (t, *J* = 7.5 Hz, 1H), 7.34-7.26 (m, 1H), 7.01 (t, *J* = 7.6 Hz, 1H), 6.90 (dd, *J* = 17.6, 8.0 Hz, 2H), 6.40 (d, *J* = 7.8 Hz, 1H), 4.22 (ddd, *J* = 10.0, 7.6, 2.7 Hz, 1H), 4.10-3.88 (m, 4H), 3.82 (dd, *J* = 6.9, 3.7 Hz, 2H), 3.76 (d, *J* = 7.6 Hz, 1H), 2.50 (s, 3H); ^{13}C NMR (150 MHz, $CDCl_3$) δ 193.4, 144.5, 140.6, 138.5, 137.1, 136.1, 135.3, 132.8, 132.4, 130.6, 130.3, 130.0, 129.7, 128.6, 128.4, 128.1, 127.9, 127.7, 127.7, 127.2, 124.6, 115.9, 77.7, 70.6, 67.1, 66.5, 54.4, 46.3, 21.4.

11-(1,4-dioxan-2-yl)-5-(3-fluorophenyl)-10-oxo-10,11-dihydro-10aH-benzo[*b*]fluorene-10*a*-carbonitrile (2k): A clean vial (5 mL) equipped with a magnetic stir bar was added to **1k** (70 mg, 0.2 mmol, 1.0 equiv), 1,10-

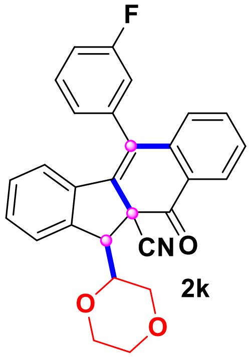

2k

phenanthroline (7 mg, 0.04 mmol, 20 mol%), and $NiCl_2$ (3 mg, 0.02 mmol, 10 mol%). Next, 1,4-Dioxane, (3.0 mL) was added after which, TBHP (aq.70%, 51.5 mg, 0.4 mmol, 2.0 equiv), DIPEA (3 mg, 0.02 mmol, 10 mol%) and $(Ir[dF(CF_3)ppy]_2(dtbpy))PF_6$ (2 mg, 0.002 mmol, 1 mol%) were added at room temperature, and then placed at a distance of approx. 3 cm from a 24 W blue LED, and the solution was stirred at room temperature under visible-light irradiation for 4 h. The progress of the reaction was monitored by thin layer chromatography. When the reaction was complete, water was added to quench the reaction

mixture, followed by extraction with ethyl acetate (3×10 mL). Finally, the combined organic layer was dried over sodium sulfate, filtered, and concentrated under vacuum. The residue was purified by column chromatography (Hex/EA = 93:7) on silica gel to afford the corresponding **2k** as 2 separable (major/minor) products (yield: 60 mg, 69%, 1.1:1 dr, yellow solid), m.p.= 273-274 °C; HRMS (ESI) m/z: [M+H]+ Calcd for $C_{28}H_{21}FNO_3$, 438.1503; Found, 438.1505. Major: ^{1}H NMR (400 MHz, $CDCl_3$) δ 7.98 (dd, *J* = 7.6, 1.1 Hz, 1H), 7.57 (ddd, *J* = 18.5, 9.2, 3.8 Hz, 2H), 7.42 (tt, *J* = 16.0, 8.4 Hz, 3H), 7.33-7.20 (m, 3H), 7.03 (t, *J* = 7.7 Hz, 1H), 6.88 (t, *J* = 7.1 Hz, 1H), 6.76 (dd, *J* = 22.2, 8.3 Hz, 1H), 6.37 (d, *J* = 8.0 Hz, 1H), 4.48-4.43 (m, 1H), 4.32 (dd, *J* = 11.8, 2.5 Hz, 1H), 3.96 (d, *J* = 4.4 Hz, 1H), 3.91-3.81 (m, 3H), 3.80-3.72 (m, 2H); ^{13}C NMR (101 MHz, $CDCl_3$) δ 193.5, 167.0, 164.9, 162.4, 143.6, 139.7, 138.3 (J_{C-F} = 11.43 Hz), 135.9, 135.2, 131.2, 131.1, 129.9, 128.7 (J_{C-F} = 34.56 Hz), 128.1, 127.8, 127.5, 126.4, 125.2, 124.5, 124.1, 117.9 (J_{C-F} = 30.55 Hz), 116.6, 116.0 (J_{C-F} = 18.81 Hz), 115.8, 115.7, 115.6, 115.4, 74.5, 69.4, 67.6, 66.2, 53.7, 52.9, 47.5.

Minor: ^{1}H NMR (597 MHz, $CDCl_3$) δ 7.98 (dd, *J* = 7.6, 1.1 Hz, 1H), 7.92 (d, *J* = 7.9 Hz, 1H), 7.68-7.48 (m, 2H), 7.41 (ddd, *J* = 36.8, 21.6, 4.8 Hz, 2H), 7.29-7.24 (m, 3H), 7.03 (t, *J* = 7.6 Hz, 1H), 6.94-6.85 (m, 1H), 6.77 (dd, *J* = 33.6, 7.9 Hz, 1H), 6.37 (d, *J* = 8.0 Hz, 1H), 4.22 (ddd, *J* = 10.0, 7.6, 2.5 Hz, 1H), 4.07 (dd, *J* = 11.6, 2.5 Hz, 1H), 4.04-3.95 (m, 2H), 3.94-3.88 (m, 1H), 3.85-3.80 (m, 2H), 3.76 (d, *J* = 7.6 Hz, 1H); ^{13}C NMR (150 MHz, $CDCl_3$) δ 192.9, 164.7 (J_{C-F} = 270.3 Hz), 144.8, 139.8, 138.2 (J_{C-F} = 7.62 Hz), 137.6, 135.6, 135.4, 131.6, 131.1, 130.8, 130.0, 128.7, 128.6, 128.1, 127.4, 126.3, 124.2, 117.7 (J_{C-F} = 11.73 Hz), 115.9, 115.8, 115.8, 115.7, 115.5, 77.7, 70.5, 67.1, 66.5, 54.4, 46.3.

11-(1,4-dioxan-2-yl)-10-oxo-5-phenyl-1,2,3,4,10,11-hexahydro-10a*H*-benzo[*b*]fluorene-10a-carbonitrile (2l): A clean vial (5 mL) equipped with a magnetic stir bar was added to **1l** (67 mg, 0.2 mmol, 1.0 equiv), 1,10-phenanthroline (7 mg, 0.04 mmol, 20 mol%), and $NiCl_2$ (3 mg, 0.02 mmol, 10 mol%). Next, 1,4-Dioxane, (3.0 mL) was added after which, TBHP (aq.70%, 51.5 mg, 0.4 mmol, 2.0 equiv), DIPEA (3 mg, 0.02 mmol, 10 mol%) and (Ir[dF(CF_3)ppy]$_2$(dtbpy))PF_6 (2 mg, 0.002 mmol, 1 mol%) were added at room temperature, and then placed at a distance of approx. 3 cm from a 40 W blue LED lamp, and the solution was stirred at room temperature under visible-light irradiation for 4 h. The progress of the reaction was monitored by thin layer chromatography. When the reaction was complete, water was added to quench the reaction mixture, followed by extraction with ethyl acetate (3×10 mL). Finally, the combined organic layer was dried over sodium sulfate, filtered, and concentrated under vacuum. The residue was purified by column chromatography (Hex/EA = 93:7) on silica gel to afford the corresponding **2l** as 2 separable (major/minor) products (yield: 70 mg, 83%, 1:1 dr, yellow solid), m.p.= 223-225 °C; HRMS (ESI) m/z: [M+H]+ Calcd for $C_{28}H_{26}NO_3$, 424.1916; Found, 424.1916. Major: ^{1}H NMR (597 MHz, $CDCl_3$) δ 7.917.86 (m, 1H), 7.47-7.43 (m, 3H), 7.42-7.39 (m, 1H), 7.37-7.33 (m, 1H), 7.31 (td, *J* = 7.5, 1.1 Hz, 1H), 7.05-7.00 (m, 1H), 6.76 (dd, *J* = 7.9, 0.6 Hz, 1H), 4.20-4.08 (m, 2H), 3.92-3.62 (m, 5H), 3.26 (d, *J* = 2.1 Hz, 1H), 2.34 (dd, *J* = 17.3, 7.2 Hz, 1H), 2.20-

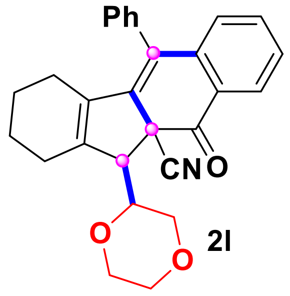

2l

2.10 (m, 1H), 1.70 (ddd, *J* = 12.7, 8.8, 4.2 Hz, 2H), 1.50-1.35 (m, 3H), 1.23-1.13 (m, 1H); ^{13}C NMR (150 MHz, $CDCl_3$) δ 194.6, 149.6, 143.0, 141.4, 136.7, 135.8, 134.9, 131.7, 129.7, 128.4, 128.3, 128.0, 128.0, 127.7, 127.6, 127.5, 127.4, 117.3, 73.3, 69.5, 67.3, 66.0, 52.1, 49.7, 25.9, 25.1, 22.1, 21.7.

Minor: ^{1}H NMR (597 MHz, $CDCl_3$) δ 7.91 (dd, *J* = 7.6, 1.2 Hz, 1H), 7.50-7.39 (m, 4H), 7.36 (td, *J* = 7.5, 0.8 Hz, 1H), 7.31 (td, *J* = 7.5, 1.0 Hz, 1H), 7.02 (d, *J* = 7.5 Hz, 1H), 6.76 (d, *J* = 7.5 Hz, 1H), 4.08 (ddd, *J* = 10.4, 4.8, 2.6 Hz, 1H), 3.94 (dd, *J* = 11.5, 2.5 Hz, 1H), 3.81 (dddd, *J* = 25.3, 13.3, 9.6, 2.9 Hz, 4H), 3.70 (td, *J* = 11.4, 3.2 Hz, 1H), 3.15 (s, 1H), 2.68-2.57 (m, 1H), 2.28-2.19 (m, 1H), 1.74-1.63 (m, 2H), 1.47-1.39 (m, 2H), 1.39-1.31 (m, 1H), 1.23-1.15 (m, 1H); ^{13}C NMR (150 MHz, $CDCl_3$) δ 193.8, 150.2, 142.1, 141.6, 136.7, 135.9, 135.1, 131.3, 129.7, 128.3, 128.2, 128.1, 128.0, 127.6, 127.5, 127.5, 127.4, 116.7, 76.4, 70.0, 67.0, 66.4, 53.6, 49.2, 28.3, 25.3, 21.9, 21.9.

10-oxo-5-phenyl-11-tetrahydrofuran-2-yl)-10,11-dihydro-10a*H*-benzo[*b*]fluorene-10*a*-carbonitrile (2m): A clean vial (5 mL) equipped with a magnetic stir bar was added to **1m** (67 mg, 0.2 mmol, 1.0 equiv), 1,10-phenanthroline (7 mg, 0.04 mmol, 20 mol%), and $NiCl_2$ (3 mg, 0.02 mmol, 10 mol%). Next, 1,4-Dioxane, (3.0 mL) was added after which, TBHP (aq.70%, 51.5 mg, 0.4 mmol, 2.0 equiv), DIPEA (3 mg, 0.02 mmol, 10 mol%) and (Ir[dF(CF_3)ppy]$_2$(dtbpy))PF_6 (2 mg, 0.002 mmol, 1 mol%) were added at room temperature, and then placed at a distance of approx. 3 cm from a 40 W blue LED lamp, and the solution was stirred at room temperature under visible-light irradiation for 4 h. The progress of the reaction was monitored by thin layer chromatography. When the reaction was complete, water was added to quench the reaction mixture, followed by extraction with ethyl acetate (3×10 mL). Finally, the combined organic layer was dried over sodium sulfate, filtered, and concentrated under vacuum. The residue was purified by column chromatography (Hex/EA = 93:7) on silica gel to afford the corresponding **2m** as 2 separable (major/minor) products. (yield: 57 mg, 71%, 1.2:1 dr, yellow solid), m.p.= 197-199 °C; HRMS (ESI) m/z: [M+Na]+ Calcd for $C_{28}H_{21}NO_2Na$, 426.1462; Found, 426.1464. Major: ^{1}H NMR (400 MHz, $CDCl_3$) δ 7.98 (dd, *J* = 7.5, 1.2 Hz, 1H), 7.64 (d, *J* = 7.4 Hz, 2H), 7.58 – 7.45 (m, 4H), 7.40 (dd, *J* = 7.5, 6.6 Hz, 1H), 7.22 (d, *J* = 7.3 Hz, 1H), 6.98 (dd, *J* = 15.5, 7.7 Hz, 2H), 6.90 (d, *J* = 7.7 Hz, 1H), 6.33 (d, *J* = 7.9 Hz, 1H), 4.65 (dd, *J* = 12.9, 6.8 Hz, 1H), 4.22 (d, *J* = 4.9 Hz, 1H), 4.12 (dd, *J* = 14.6, 7.0 Hz, 1H), 3.84 (dd, *J* = 14.5, 7.7 Hz, 1H), 2.41 (dd, *J* = 12.3, 4.9 Hz, 1H), 2.07 (dt, *J* = 11.9, 5.8 Hz, 2H), 1.99 – 1.88 (m, 1H); ^{13}C NMR (101 MHz, $CDCl_3$) δ 193.7, 144.3, 140.4, 137.7, 136.2, 135.0, 132.5, 130.6, 129.9, 129.6, 129.3, 129.0, 128.6, 128.4, 128.3, 127.8, 127.7, 125.2, 124.6, 117.1, 114.5, 79.0, 67.7, 54.0, 49.8, 29.1, 26.5.

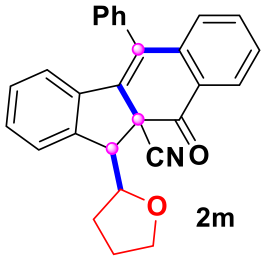

2m

Minor: ^{1}H NMR (400 MHz, $CDCl_3$) δ 8.01 (dd, *J* = 7.9, 0.6 Hz, 1H), 7.94 (dd, *J* = 7.6, 1.5 Hz, 1H), 7.63 (t, *J* = 8.8 Hz, 2H), 7.57 – 7.47 (m, 3H), 7.40 (td, *J* = 7.5, 1.1 Hz, 1H), 7.27 – 7.22 (m, 1H), 7.04 – 6.94 (m, 2H), 6.92 – 6.86 (m, 1H), 6.28 (d, *J* = 8.0 Hz, 1H), 4.32 (ddd, *J* = 9.2, 7.1, 6.3 Hz, 1H), 4.17 – 4.09 (m, 1H), 3.95 (ddd, *J* = 7.9, 6.7, 4.0 Hz, 2H), 2.26 (dddd, *J* = 13.8, 12.6, 6.7, 5.3 Hz, 3H), 2.07 – 1.96 (m, 1H); ^{13}C NMR (101 MHz,

$CDCl_3$) δ 193.9, 146.8, 140.3, 138.0, 136.2, 135.6, 135.1, 132.2, 130.5, 129.9, 129.3, 129.0, 128.6, 128.4, 128.3, 127.8, 127.6, 127.6, 127.3, 124.0, 116.4, 81.8, 68.2, 54.3, 48.6, 31.1, 25.6.

ethyl 11-(1,4-dioxan-2-yl)-10-oxo-5-phenyl-10,11-dihydro-10aH-benzo[b]fluorene-10a-carboxylate (2n): A

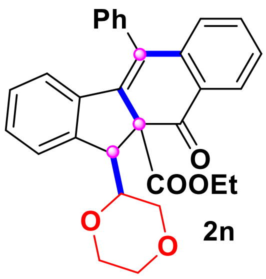

2n

clean vial (5 mL) equipped with a magnetic stir bar was added to **1n** (76 mg, 0.2 mmol, 1.0 equiv), 1,10-phenanthroline (7 mg, 0.04 mmol, 20 mol%), and $NiCl_2$ (3 mg, 0.02 mmol, 10 mol%). Next, 1,4-Dioxane, (3.0 mL) was added after which, TBHP (aq.70%, 51.5 mg, 0.4 mmol, 2.0 equiv), DIPEA (3 mg, 0.02 mmol, 10 mol%) and $(Ir[dF(CF_3)ppy]_2(dtbpy))PF_6$ (2 mg, 0.002 mmol, 1 mol%) were added at room temperature, and then placed at a distance of approx. 3 cm from a 40 W blue LED lamp, and the solution was stirred at room temperature under visible-light irradiation for 4 h. The progress of the reaction was monitored by thin layer chromatography. When the reaction was complete, water was added to quench the reaction mixture, followed by extraction with ethyl acetate (3×10 mL). Finally, the combined organic layer was dried over sodium sulfate, filtered, and concentrated under vacuum. The residue was purified by column chromatography (Hex/EA = 93:7) on silica gel to afford the corresponding **2n** as 2 separable (major/minor) products (yield: 69 mg, 74%, 1.3:1 dr, yellow solid), m.p.= 223-225 °C; HRMS (ESI) m/z: [M+Na]+ Calcd for $C_{30}H_{26}O_5Na$, 489.1669; Found, 489.1672. Major: ^{1}H NMR (400 MHz, $CDCl_3$) δ 8.17 (dd, *J* = 7.7, 1.3 Hz, 1H), 7.62 (t, *J* = 7.2 Hz, 1H), 7.56 – 7.43 (m, 4H), 7.34 (td, *J* = 7.6, 1.1 Hz, 1H), 7.16 (dt, *J* = 13.7, 7.2 Hz, 2H), 7.03 (d, *J* = 7.1 Hz, 1H), 6.92 (dd, *J* = 10.3, 4.5 Hz, 2H), 6.24 (d, *J* = 7.9 Hz, 1H), 4.47 – 4.38 (m, 1H), 4.27 (d, *J* = 3.5 Hz, 1H), 3.95 (dddd, *J* = 17.8, 10.8, 7.1, 3.6 Hz, 3H), 3.51 – 3.34 (m, 3H), 3.26 (dt, *J* = 17.0, 5.5 Hz, 2H), 0.96 (t, *J* = 7.1 Hz, 3H); ^{13}C NMR (101 MHz, $CDCl_3$) δ 193.8, 168.1, 143.9, 141.9, 141.4, 139.3, 137.6, 133.9, 130.7, 129.8, 129.5, 129.1, 129.0, 128.8, 127.9, 127.8, 127.2, 127.0, 126.9, 125.9, 124.3, 72.8, 69.2, 68.3, 66.8, 66.0, 62.2, 51.2, 13.6.

Minor: ^{1}H NMR (400 MHz, $CDCl_3$) δ 8.17 (dd, *J* = 7.7, 1.4 Hz, 1H), 7.63 (t, *J* = 7.3 Hz, 1H), 7.56 – 7.43 (m, 5H), 7.36 (td, *J* = 7.5, 1.1 Hz, 1H), 7.17 (td, *J* = 7.5, 1.0 Hz, 1H), 7.01 (d, *J* = 7.2 Hz, 1H), 6.96 – 6.89 (m, 2H), 6.19 (d, *J* = 7.9 Hz, 1H), 4.46 (d, *J* = 4.2 Hz, 1H), 4.34 (ddd, *J* = 10.4, 4.3, 2.4 Hz, 1H), 4.04 – 3.90 (m, 2H), 3.81 – 3.70 (m, 2H), 3.53 – 3.45 (m, 1H), 3.29 – 3.17 (m, 2H), 2.76 – 2.66 (m, 1H), 0.99 (t, *J* = 7.1 Hz, 3H); ^{13}C NMR (101 MHz, $CDCl_3$) δ 193.8, 167.5, 145.4, 141.1, 137.6, 136.9, 134.7, 131.1, 130.6, 129.8, 129.0, 128.8, 128.5, 128.5, 128.2, 127.5, 127.5, 127.2, 127.2, 123.8, 74.4, 68.1, 67.4, 66.9, 66.1, 62.5, 50.8, 13.6.

10*a*-acetyl-11-1,4-dioxan-2-yl)-5-phenyl-10*a*,11-dihydro-10H-benzo[*b*]fluoren-10-one (2o): A clean vial (5 mL) equipped with a magnetic stir bar was added to **1o** (73 mg, 0.2 mmol, 1.0 equiv), 1,10-phenanthroline (7 mg, 0.04 mmol, 20 mol%), and $NiCl_2$ (3 mg, 0.02 mmol, 10 mol%). Next, 1,4-Dioxane, (3.0 mL) was added after which, TBHP (aq.70%, 51.5 mg, 0.4 mmol, 2.0 equiv), DIPEA (3 mg, 0.02 mmol, 10 mol%) and (Ir[dF(CF_3)ppy]$_2$(dtbpy))PF_6 (2 mg, 0.002 mmol, 1 mol%) were added at room temperature, and then placed at a distance of approx. 3 cm from a 40 W blue LED lamp, and the solution was stirred at room temperature under visible-light irradiation for 4 h. The progress of the reaction was monitored by thin layer chromatography. When the reaction was complete, water was added to quench the reaction mixture, followed by extraction with ethyl acetate (3×10 mL). Finally, the combined organic layer was dried over sodium sulfate, filtered, and concentrated under vacuum. The residue was purified by column chromatography (Hex/EA = 93:7) on silica gel to afford the corresponding **2o** as an inseparable mixture of products (yield: 63 mg, 72%, pale yellow solid), m.p.= 211-213 °C; HRMS (ESI) m/z: [M+Na]+ Calcd for $C_{29}H_{24}O_4Na$, 459.1564; Found, 459.1566. ^{1}H NMR (400 MHz, $CDCl_3$) δ 7.92 (ddd, *J* = 13.3, 7.5, 1.5 Hz, 2H), 7.69 – 7.60 (m, 3H), 7.57 – 7.47 (m, 6H), 7.45 – 7.36 (m, 3H), 7.35 – 7.21 (m, 5H), 7.00 (ddd, *J* = 15.3, 9.9, 3.2 Hz, 4H), 6.83 – 6.73 (m, 2H), 6.36 (dd, *J* = 15.2, 7.9 Hz, 2H), 4.51 (d, *J* = 10.3 Hz, 1H), 4.33 – 3.91 (m, 7H), 3.82 – 3.48 (m, 10H), 2.08 (s, 3H), 2.07 (s, 3H); ^{13}C NMR (101 MHz, $CDCl_3$) δ 203.0, 202.2, 200.7, 199.2, 145.9, 144.0, 140.4, 140.0, 139.5, 139.1, 138.7, 137.2, 137.2, 133.9, 133.6, 132.0, 131.7, 131.3, 130.7, 130.0, 129.9, 129.9, 129.2, 129.2, 128.9, 128.7, 128.3, 128.0, 128.0, 127.6, 127.4, 126.9, 126.8, 126.7, 126.4, 124.2, 124.2, 123.7, 114.2, 74.8, 74.7, 74.2, 69.7, 69.1, 67.5, 66.9, 66.3, 65.8, 49.0, 48.7, 29.5, 29.0.

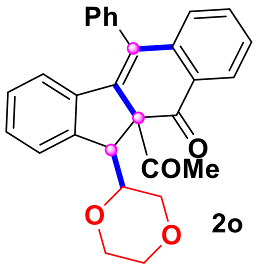

2o

11-((methyl(phenyl)amino)methyl)-10-oxo-5-phenyl-10,11-dihydro-10a*H*-benzo[*b*]fluorene-10*a*-carbonitrile (2q): A clean vial (5 mL) equipped with a magnetic stir bar was added to **1a** (67 mg, 0.2 mmol), 1,10-phenanthroline (7 mg, 0.04 mmol, 20 mol%), and $NiCl_2$ (3 mg, 0.02 mmol, 10 mol%). Next, 1,2-DCE (3.0 mL) was added after which, *N*,*N*-dimethylaniline (48mg, 0.4 mmol, 2.0 equiv) and (Ir[dF(CF_3)ppy]$_2$(dtbpy))PF_6 (2 mg, 0.002 mmol, 1 mol%) were added at room temperature, and then placed at a distance of approx. 3 cm from a 40 W blue LED lamp, and the solution was stirred at room temperature under visible-light irradiation for 6 h. The progress of the reaction was monitored by thin layer chromatography. When the reaction was complete, water was added to quench the reaction mixture, followed by extraction with ethyl acetate (3×10 mL). Finally, the combined organic layer was dried over sodium sulfate, filtered, and concentrated under vacuum. The residue was purified by column chromatography (Hex/EA = 95:5) on silica gel to afford the corresponding **2q** derivative. HRMS (ESI) m/z: [M+H]+ Calcd for $C_{32}H_{24}N_2O$, 452.1889; Found, 452.1892. ^{1}H NMR (400 MHz, $CDCl_3$) δ 8.10-8.03 (m, 1H), 7.66 (d, *J* = 7.3 Hz, 2H), 7.60-7.40 (m, 5H), 7.38-7.30 (m, 2H), 7.22-7.10 (m, 3H), 7.03-6.90 (m, 3H), 6.82 (t, *J* = 7.3 Hz, 1H), 6.32 (d, *J* = 7.9 Hz, 1H), 4.29-4.16 (m, 2H), 3.99 (dd, *J* = 13.6, 9.9 Hz, 1H), 3.23 (s, 3H); ^{13}C NMR

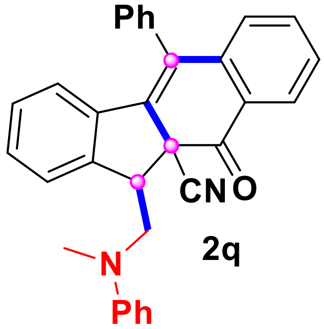

2q

(101 MHz, $CDCl_3$) δ 192.9, 149.7, 146.0, 140.6, 135.9, 135.5, 132.2, 130.5, 130.0, 129.9, 129.3, 128.6, 128.5, 128.4, 128.3, 127.8, 125.7, 124.4, 117.7, 115.9, 114.0, 56.2, 54.9, 42.9, 40.6; Yield: 66 mg, 73%, yellow solid, m.p.= 239-241 °C.

10-oxo-5-phenyl-10,11-dihydro-10a*H*-benzo[*b*]fluorene-10*a*-carbonitrile (2r): A clean vial (5 mL) equipped with a magnetic stir bar was added to **1a** (67 mg, 0.2 mmol), 1,10-phenanthroline (7 mg, 0.04 mmol, 20 mol%), and $NiCl_2$ (3 mg, 0.02 mmol, 10 mol%). Next, 1,2-DCE (3.0 mL) was added after which, Bu_3SnH (116 mg, 0.4 mmol, 2.0 equiv) TBHP (aq.70%, 51.5 mg, 0.4 mmol, 2.0 equiv), DIPEA (3 mg, 0.02 mmol, 10 mol%) and (Ir[dF(CF_3)ppy]$_2$(dtbpy))PF_6 (2 mg, 0.002 mmol, 1 mol%) were added at room temperature, and then placed at a distance of approx. 3 cm from a 40 W blue LED lamp, and the solution was stirred at room temperature under visible-light irradiation for 6 h. The progress of the reaction was monitored by thin layer chromatography. When the reaction was complete, water was added to quench the reaction mixture, followed by extraction with ethyl acetate (3×10 mL). Finally, the combined organic layer was dried over sodium sulfate, filtered, and concentrated under vacuum. The residue was purified by column chromatography (Hex/EA = 95:5) on silica gel to afford the corresponding **2r** derivative. HRMS (ESI) m/z: [M+H]+ Calcd for $C_{24}H_{16}NO$, 334.1151; Found, 334.1154. ^{1}H NMR (400 MHz, $CDCl_3$) δ 8.11-7.99 (m, 1H), 7.65 (d, J = 7.3 Hz, 2H), 7.54 (ddd, J = 16.3, 11.2, 4.2 Hz, 3H), 7.41 (ddd, J = 7.6, 7.0, 3.2 Hz, 2H), 7.29-7.21 (m, 1H), 7.06-6.90 (m, 3H), 6.35 (d, J = 8.0 Hz, 1H), 3.69 (s, 2H); ^{13}C NMR (101 MHz, $CDCl_3$) δ 192.7, 143.2, 140.7, 138.0, 136.4, 136.0, 135.4, 132.6, 130.6, 129.9, 129.7, 129.3, 128.6, 128.4, 128.3, 127.8, 127.8, 127.6, 125.6, 124.7, 118.4, 51.6, 37.4; Yield: 61 mg, 91%, yellow solid, m.p.= 210-212 °C.

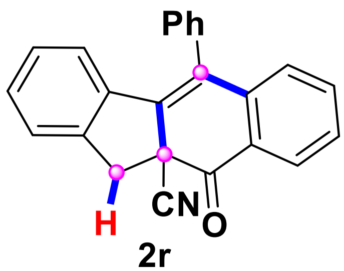

2r

10-oxo-5-phenyl-10,11-dihydro-10a*H*-benzo[6,7]fluoreno[2,3-*d*][1,3]dioxole-10*a*-carbonitrile (2s): A clean vial (5 mL) equipped with a magnetic stir bar was added to **1s** (76 mg, 0.2 mmol, 1.0 equiv), 1,10-phenanthroline (7 mg, 0.04 mmol, 20 mol%), and $NiCl_2$ (3 mg, 10 mol%). Next, 1,2-DCE (3.0 mL) was added after which, Bu_3SnH (116.4 mg, 0.4 mmol, 2.0 equiv) and (Ir[dF(CF_3)ppy]$_2$(dtbpy))PF_6 (2 mg, 0.002 mmol, 1 mol%) were added at room temperature, and then placed at a distance of approx. 3 cm from a 40 W blue LED lamp, and the solution was stirred at room temperature under visible-light irradiation for 6 h. The progress of the reaction was monitored by thin layer chromatography. When the reaction was complete, water was added to quench the reaction mixture, followed by extraction with ethyl acetate (3×10 mL). Finally, the combined organic layer was dried over sodium sulfate, filtered, and concentrated under vacuum. The residue was purified by column chromatography (Hex/EA = 95:5) on silica gel to afford the corresponding **2s** derivatives. HRMS (ESI) m/z: [M+H]+ Calcd for $C_{25}H_{16}NO_3$, 378.1124; Found, 378.1126. ^{1}H NMR (597 MHz, $CDCl_3$) δ 8.03 (dd, J = 7.6, 1.4 Hz, 1H), 7.62 (dd, J = 23.9, 7.4 Hz, 2H), 7.56-7.46 (m, 3H),

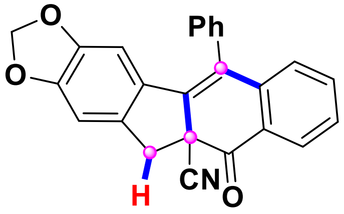

2s

7.38 (td, J = 7.5, 1.1 Hz, 1H), 7.02 (d, J = 7.4 Hz, 1H), 6.90 (dd, J = 8.0, 0.6 Hz, 1H), 6.83 (s, 1H), 5.90 (dd, J = 15.9, 1.4 Hz, 2H), 5.73 (s, 1H), 3.58 (s, 2H); ^{13}C NMR (150 MHz, $CDCl_3$) δ 192.5, 149.5, 147.7, 141.1, 138.6, 137.9, 135.9, 135.4, 130.6, 130.3, 130.2, 129.9, 129.3, 128.7, 128.5, 127.9, 127.9, 127.7, 127.5, 118.5, 105.7, 104.4, 101.7, 52.3, 37.2; Yield: 68 mg, 90%, yellow solid, m.p.= 238-240 °C.

10-oxo-5-(*p*-tolyl)-10,11-dihydro-10a*H*-benzo[*b*]fluorene-10*a*-carbonitrile (2t): A clean vial (5 mL) equipped

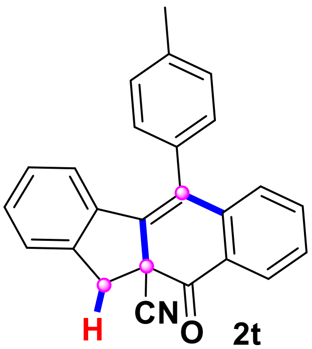

2t

with a magnetic stir bar was added to **1t** (69 mg, 0.2 mmol, 1.0 equiv), 1,10-phenanthroline (7 mg, 0.04 mmol, 20 mol%), and $NiCl_2$ (3 mg, 10 mol%). Next, 1,2-DCE (3.0 mL) was added after which, Bu_3SnH (116.4 mg, 0.4 mmol, 2.0 equiv) TBHP (aq.70%, 52 mg, 0.4 mmol, 2.0 equiv), DIPEA (3 mg, 0.02 mmol, 10 mol%) and $(Ir[dF(CF_3)ppy]_2(dtbpy))PF_6$ (2 mg, 0.002 mmol, 1 mol%) were added at room temperature, and then placed at a distance of approx. 3 cm from a 40 W blue LED lamp, and the solution was stirred at room temperature under visible-light irradiation for 6 h. The progress of the reaction was monitored by thin layer chromatography. When the reaction was complete, water was added to quench the reaction mixture, followed by extraction with ethyl acetate (3×10 mL). Finally, the combined organic layer was dried over sodium sulfate, filtered, and concentrated under vacuum. The residue was purified by column chromatography (Hex/EA = 95:5) on silica gel to afford the corresponding **2t** derivative. HRMS (ESI) m/z: [M+H]+ calcd for $C_{25}H_{18}NO$, 348.1310; found, 348.1314. ^{1}H NMR (400 MHz, $CDCl_3$) δ 8.04 (dd, J = 7.6, 1.1 Hz, 1H), 7.62 – 7.50 (m, 2H), 7.49 – 7.38 (m, 3H), 7.30 (d, J = 7.8 Hz, 1H), 7.24 (d, J = 7.5 Hz, 1H), 7.05 – 6.94 (m, 2H), 6.90 (d, J = 7.9 Hz, 1H), 6.44 (d, J = 7.9 Hz, 1H), 3.69 (s, 2H), 2.50 (s, 3H); ^{13}C NMR (101 MHz, $CDCl_3$) δ 192.8, 143.2, 141.0, 138.4, 137.9, 136.6, 135.3, 132.9, 132.7, 130.5, 129.9, 129.6, 128.3, 128.1, 127.9, 127.7, 127.6, 125.5, 124.8, 118.5, 51.6, 37.4, 21.4; Yield: 61 mg, 88%, pale yellow solid, m.p.= 203-205 °C.

2-fluoro-10-oxo-5-phenyl-10,11-dihydro-10a*H*-benzo[*b*]fluorene-10a-carbonitrile (2u): A clean vial (5 mL)

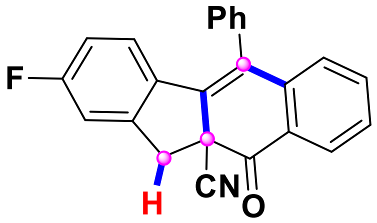

2u

equipped with a magnetic stir bar was added to **1c** (70 mg, 0.2 mmol, 1.0 equiv), 1,10-phenanthroline (7 mg, 0.04 mmol, 20 mol%), and $NiCl_2$ (3 mg, 10 mol%). Next, 1,2-DCE (3.0 mL) was added after which, Bu_3SnH (116.4 mg, 0.4 mmol, 2.0 equiv) TBHP (aq.70%, 52 mg, 0.4 mmol, 2.0 equiv), DIPEA (3 mg, 0.02 mmol, 10 mol%) and $(Ir[dF(CF_3)ppy]_2(dtbpy))PF_6$ (2 mg, 0.002 mmol, 1 mol%) were added at room temperature, and then placed at a distance of approx. 3 cm from a 24 W blue LED, and the solution was stirred at room temperature under visible-light irradiation for 6 h. The progress of the reaction was monitored by thin layer chromatography. When the reaction was complete, water was added to quench the reaction mixture, followed by extraction with ethyl acetate (3×10 mL). Finally, the combined organic layer was dried over sodium sulfate, filtered, and concentrated under vacuum. The residue was purified by column chromatography (Hex/EA = 95:5) on silica gel to afford the corresponding **2u** derivative.

HRMS (ESI) m/z: [M+H]+ Calcd for $C_{24}H_{15}FNO$, 352.1059; Found, 352.1062. ^{1}H NMR (400 MHz, $CDCl_3$) δ 8.05 (ddd, *J* = 7.6, 1.5, 0.5 Hz, 1H), 7.65 (dt, *J* = 14.8, 7.4 Hz, 2H), 7.59-7.47 (m, 3H), 7.42 (td, *J* = 7.5, 1.1 Hz, 1H), 7.12 – 7.07 (m, 1H), 7.01 (d, *J* = 7.3 Hz, 1H), 6.96-6.91 (m, 1H), 6.70 (td, *J* = 8.8, 2.5 Hz, 1H), 6.30 (dd, *J* = 8.8, 5.3 Hz, 1H), 3.68 (s, 2H); ^{13}C NMR (101 MHz, $CDCl_3$) δ 192.4, 164.7 ($J_{C\text{-}F}$ = 250.23 Hz), 162.2, 145.6 ($J_{C\text{-}F}$ = 8.86 Hz), 140.6, 136.7, 135.7, 135.5, 132.6, 132.1, 130.5, 129.9, ($J_{C\text{-}F}$ = 58.42 Hz), 128.8, 128.5, 128.3, 128.1, 127.8, 127.8, 126.2 ($J_{C\text{-}F}$ = 8.86 Hz), 118.2, 115.4 ($J_{C\text{-}F}$ = 22.69 Hz), 112.9, ($J_{C\text{-}F}$ = 22.86 Hz), 51.9, 37.3 ($J_{C\text{-}F}$ = 2.14 Hz); Yield: 63 mg, 90%, pale yellow solid, m.p.= 227-229 °C.

7-methoxy-10-oxo-5-phenyl-10,11-dihydro-10a*H*-benzo[*b*]fluorene-10*a*-carbonitrile (2v): A clean vial (5 mL) equipped with a magnetic stir bar was added to **1e** (73 mg, 0.2 mmol, 1.0 equiv), 1,10-phenanthroline (7 mg, 0.04 mmol, 20 mol%), and $NiCl_2$ (3 mg, 10 mol%). Next, 1,2-DCE (3.0 mL) was added after which, Bu_3SnH (116.4 mg, 0.4 mmol, 2.0 equiv) and (Ir[dF(CF_3)ppy]$_2$(dtbpy))PF_6 (2 mg, 0.002 mmol, 1 mol%) were added at room temperature, and then placed at a distance of approx. 3 cm from a 40 W blue LED, and the solution was stirred at room temperature under visible-light irradiation for 6 h. The progress of the reaction was monitored by thin layer chromatography. When the reaction was complete, water was added to quench the reaction mixture, followed by extraction with ethyl acetate (3×10 mL). Finally, the combined organic layer was dried over sodium sulfate, filtered, and concentrated under vacuum. The residue was purified by column chromatography (Hex/EA = 95:5) on silica gel to afford the corresponding **2v** derivative. HRMS (ESI) m/z: [M+H]+ Calcd for $C_{25}H_{18}NO_2$, 364.1332; Found, 364.1333. ^{1}H NMR (400 MHz, $CDCl_3$) δ 8.07 (d, *J* = 8.6 Hz, 1H), 7.69 – 7.59 (m, 2H), 7.57 – 7.44 (m, 2H), 7.39 (d, *J* = 7.6 Hz, 1H), 7.23 (d, *J* = 7.6 Hz, 1H), 7.06 – 6.94 (m, 2H), 6.91 (dd, *J* = 8.6, 2.5 Hz, 1H), 6.40 (d, *J* = 2.4 Hz, 1H), 6.33 (d, *J* = 7.9 Hz, 1H), 3.76 (s, 3H), 3.67 (q, *J* = 16.7 Hz, 2H); ^{13}C NMR (101 MHz, $CDCl_3$) δ 190.8, 165.4, 143.3, 143.2, 139.0, 136.5, 135.9, 132.3, 130.5, 130.4, 129.8, 129.7, 129.3, 128.6, 128.4, 127.6, 125.6, 124.7, 121.7, 118.8, 113.7, 113.2, 55.5, 51.5, 37.6; Yield: 59 mg, 81%, yellow solid, m.p.= 212-214 °C.

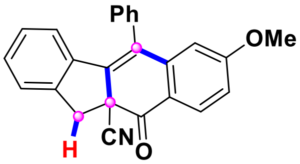

2v

9-oxo-4-phenyl-9,10-dihydro-9a*H*-benzo[5,6]indeno[2,1-*b*]thiophene-9*a*-carbonitrile (2w): A clean vial (5 mL) equipped with a magnetic stir bar was added to **1w** (68 mg, 0.2 mmol, 1.0 equiv), 1,10-phenanthroline (7 mg, 0.04 mmol, 20 mol%), and $NiCl_2$ (3 mg, 10 mol%). Next, 1,2-DCE (3.0 mL) was added after which, Bu_3SnH (116.4 mg, 0.4 mmol, 2.0 equiv) and (Ir[dF(CF_3)ppy]$_2$(dtbpy))PF_6 (2 mg, 0.002 mmol, 1 mol%) were added at room temperature, and then placed at a distance of approx. 3 cm from a 40 W blue LED, and the solution was stirred at room temperature under visible-light irradiation for 6 h. The progress of the reaction was monitored by thin layer chromatography. When the reaction was complete, water was added to quench the reaction mixture, followed by extraction with ethyl acetate (3×10 mL). Finally, the combined organic layer was dried over sodium sulfate, filtered, and concentrated under vacuum. The residue was

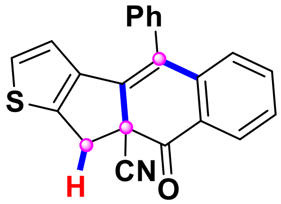

2w

purified by column chromatography (Hex/EA = 95:5) on silica gel to afford the corresponding **2w** derivative. HRMS (ESI) m/z: [M+H]+ Calcd for $C_{22}H_{14}NOS$, 340.0790; Found, 340.0792. ^{1}H NMR (400 MHz, $CDCl_3$) δ 8.03 (dd, *J* = 7.7, 1.5 Hz, 1H), 7.69 – 7.43 (m, 5H), 7.40 (td, *J* = 7.5, 1.1 Hz, 1H), 7.07 (d, *J* = 5.1 Hz, 2H), 7.01 (dd, *J* = 7.9, 0.6 Hz, 1H), 5.94 (d, *J* = 5.1 Hz, 1H), 3.74 (d, *J* = 3.4 Hz, 2H); ^{13}C NMR (101 MHz, $CDCl_3$) δ 192.1, 147.9, 142.7, 140.5, 136.0, 135.4, 134.6, 131.0, 130.2, 128.9, 128.6, 128.6, 128.0, 127.9, 127.7, 121.1, 118.2, 56.5, 35.0. Yield: 63 mg, 93%, yellow solid, m.p.= 240-242 °C.

7-(1,4-dioxan-2-yl)-5-methyl-6-oxo-12-phenyl-5,7-dihydrobenzo[*b*]indeno[1,2-e]azepine-6a(6*H*)-carbonitrile (4a): A clean vial (5 mL) equipped with a magnetic stir bar was added to **3a** (72 mg, 0.2 mmol, 1.0 equiv), 1,10-phenanthroline (7 mg, 0.04 mmol, 20 mol%), and $NiCl_2$ (3 mg, 0.02 mmol, 10 mol%). Next, 1,4-Dioxane, (3.0 mL) was added after which, TBHP (aq.70%, 51.5 mg, 0.4 mmol, 2.0 equiv), DIPEA (3 mg, 0.02 mmol, 10 mol%) and (Ir[dF(CF_3)ppy]$_2$(dtbpy))PF_6 (2 mg, 0.002 mmol, 1 mol%) were added at room temperature, and then placed at a distance of approx. 3 cm from a 40 W blue LED lamp, and the solution was stirred at room temperature under visible-light irradiation for 4 h. The progress of the reaction was monitored by thin layer chromatography. When the reaction was complete, water was added to quench the reaction mixture, followed by extraction with ethyl acetate (3×10 mL). Finally, the combined organic layer was dried over sodium sulfate, filtered, and concentrated under vacuum. The residue was purified by column chromatography (Hex/EA = 93:7) on silica gel to afford the corresponding **4a** as 2 separable (major/minor) products (yield: 76 mg, 85%, 1.3:1 dr, Off-white solid), m.p.= 223-225 °C; HRMS (ESI) m/z: [M+H]+ Calcd for $C_{29}H_{25}N_2O_3$, 449.1787; Found, 449.1786. Major: ^{1}H NMR (400 MHz, $CDCl_3$) δ 7.75-7.67 (m, 1H), 7.50-7.42 (m, 5H), 7.27-7.15 (m, 4H), 6.94 (ddd, *J* = 15.1, 6.4, 1.6 Hz, 2H), 6.40 (d, *J* = 8.0 Hz, 1H), 4.39 (d, *J* = 2.4 Hz, 1H), 4.14 (dt, *J* = 10.2, 2.3 Hz, 1H), 3.98 (dd, *J* = 10.9, 2.0 Hz, 1H), 3.81 (t, *J* = 10.6 Hz, 1H), 3.73-3.58 (m, 3H), 3.54 (s, 3H), 3.50-3.41 (m, 1H); ^{13}C NMR (101 MHz, $CDCl_3$) δ 162.8, 143.4, 141.0, 139.4, 137.5, 137.2, 134.3, 132.6, 131.3, 130.9, 130.0, 129.5, 129.4, 128.9, 128.8, 128.7, 127.6, 127.4, 125.9, 125.2, 125.1, 124.4, 122.3, 122.1, 114.4, 68.6, 67.2, 66.1, 53.5, 49.9, 38.5.

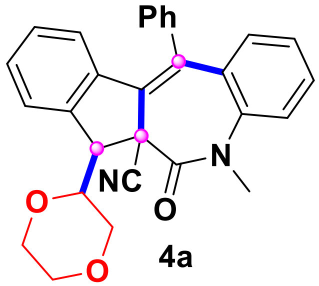

4a

Minor: ^{1}H NMR (400 MHz, $CDCl_3$) δ 7.66-7.58 (m, 1H), 7.52-7.39 (m, 6H), 7.23-7.17 (m, 2H), 7.16-7.11 (m, 1H), 7.00-6.87 (m, 2H), 6.33 (d, *J* = 8.1 Hz, 1H), 4.65 (d, *J* = 5.0 Hz, 1H), 4.07 (ddd, *J* = 10.2, 5.0, 2.4 Hz, 1H), 3.88-3.74 (m, 3H), 3.64 (dd, *J* = 11.4, 2.2 Hz, 1H), 3.55 (d, *J* = 3.2 Hz, 3H), 3.51 (dd, *J* = 11.1, 7.6 Hz, 1H), 3.29 (dd, *J* = 11.2, 10.3 Hz, 1H); ^{13}C NMR (101 MHz, $CDCl_3$) δ 162.4, 144.2, 140.9, 139.2, 136.6, 135.9, 134.1, 134.0, 130.7, 130.6, 129.9, 129.7, 129.5, 129.3, 128.9, 128.8, 127.7, 126.7, 125.3, 124.3, 122.3, 114.3, 68.2, 67.3, 66.2, 51.4, 49.9, 38.8.

7-(1,4-dioxan-2-yl)-5,9-dimethyl-6-oxo-12-phenyl-5,7-dihydrobenzo[*b*]indeno[1,2-*e*]azepine-6*a*(6*H*)-carbonitrile (4b): A clean vial (5 mL) equipped with a magnetic stir bar was added to **3a** (72 mg, 0.2 mmol, 1.0 equiv), 1,10-phenanthroline (7 mg, 0.04 mmol, 20 mol%), and $NiCl_2$ (3 mg, 0.02 mmol, 10 mol%). Next, 1,4-Dioxane, (3.0 mL) was added after which, TBHP (aq.70%, 51.5 mg, 0.4 mmol, 2.0 equiv), DIPEA (3 mg, 0.02 mmol, 10 mol%) and (Ir[dF(CF_3)ppy]$_2$(dtbpy))PF_6 (2 mg, 0.002 mmol, 1 mol%) were added at room temperature, and then placed at a distance of approx. 3 cm from a 40 W blue LED lamp, and the solution was stirred at room temperature under visible-light irradiation for 4 h. The progress of the reaction was monitored by thin layer chromatography. When the reaction was complete, water was added to quench the reaction mixture, followed by extraction with ethyl acetate (3×10 mL). Finally, the combined organic layer was dried over sodium sulfate, filtered, and concentrated under vacuum. The residue was purified by column chromatography (Hex/EA = 93:7) on silica gel to afford the corresponding **4b** as 2 separable (major/minor) products (yield: 80.5 mg, 87%, 1.4:1 dr, Off-white solid), m.p.= 235-237 °C; HRMS (ESI) m/z: [M+H]+ Calcd for $C_{30}H_{27}N_2O_3$, 463.1943; Found, 463.1945. Major: ^{1}H NMR (400 MHz, $CDCl_3$) δ 7.66-7.56 (m, 1H), 7.54-7.41 (m, 5H), 7.35 (d, *J* = 7.9 Hz, 1H), 7.24-7.12 (m, 2H), 7.08-6.89 (m, 2H), 6.07 (s, 1H), 4.59 (d, *J* = 5.1 Hz, 1H), 4.04 (ddd, *J* = 10.2, 5.1, 2.4 Hz, 1H), 3.88-3.73 (m, 3H), 3.64 (dd, *J* = 11.4, 2.2 Hz, 1H), 3.56 (d, *J* = 5.8 Hz, 3H), 3.51 (dd, *J* = 11.3, 3.6 Hz, 1H), 3.35-3.27 (m, 1H), 2.00 (s, 3H); ^{13}C NMR (101 MHz, $CDCl_3$) δ 162.4, 141.5, 140.9, 139.3, 137.3, 136.8, 136.0, 134.1, 133.7, 130.8, 130.6, 130.4, 129.9, 129.6, 129.5, 128.8, 128.7, 126.3, 125.3, 124.8, 122.3, 114.4, 68.3, 67.3, 66.2, 51.7, 49.7, 38.7, 21.4.

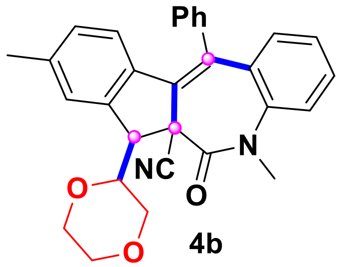

4b

Minor: ^{1}H NMR (400 MHz, $CDCl_3$) δ 7.74-7.66 (m, 1H), 7.54-7.40 (m, 5H), 7.24-7.10 (m, 3H), 7.02 (d, *J* = 6.8 Hz, 1H), 6.98-6.92 (m, 1H), 6.14 (s, 1H), 4.34 (d, *J* = 2.2 Hz, 1H), 4.12 (dt, *J* = 10.2, 2.3 Hz, 1H), 3.97 (dd, *J* = 10.9, 2.2 Hz, 1H), 3.85-3.78 (m, 1H), 3.72-3.58 (m, 3H), 3.55 (d, *J* = 7.5 Hz, 3H), 3.51-3.44 (m, 1H), 2.01 (s, 3H); ^{13}C NMR (101 MHz, $CDCl_3$) δ 162.9, 140.9, 140.6, 139.5, 137.5, 137.3, 137.2, 134.3, 132.3, 131.3, 130.9, 130.0, 129.9, 129.4, 129.3, 128.8, 128.6, 125.5, 125.2, 125.0, 122.1, 114.5, 68.6, 67.3, 66.1, 53.7, 49.6, 38.5, 21.5.

7-1,4-dioxan-2-yl)-2-methoxy-5-methyl-6-oxo-12-phenyl-5,7-dihydrobenzo[*b*]indeno[1,2-*e*]azepine-6a(6*H*)-carbonitrile (4c): A clean vial (5 mL) equipped with a magnetic stir bar was added to **3c** (mg, 0.2 mmol, 1.0 equiv), 1,10-phenanthroline (79 mg, 0.04 mmol, 20 mol%), and $NiCl_2$ (3 mg, 0.02 mmol, 10 mol%). Next, 1,4-Dioxane, (3.0 mL) was added after which, TBHP (aq.70%, 51.5 mg, 0.4 mmol, 2.0 equiv), DIPEA (3 mg, 0.02 mmol, 10 mol%) and (Ir[dF(CF_3)ppy]$_2$(dtbpy))PF_6 (2 mg, 0.002 mmol, 1 mol%) were added at room temperature, and then placed at a distance of approx. 3 cm from a 40 W blue LED lamp, and the solution was stirred at room temperature under visible-light irradiation for 4 h. The progress of the reaction was monitored by thin layer chromatography. When the reaction was complete, water was added to quench the reaction mixture, followed by extraction with ethyl acetate (3×10 mL). Finally, the combined organic layer was

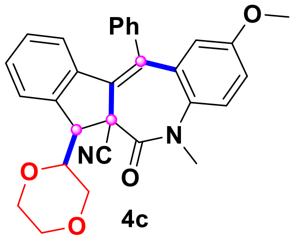

4c

dried over sodium sulfate, filtered, and concentrated under vacuum. The residue was purified by column chromatography (Hex/EA = 90:10) on silica gel to afford the corresponding **4c** as 2 separable (major/minor) products (yield: 84 mg, 88%, 1.5:1 dr, Off-white solid), m.p.= 256-258 °C; HRMS (ESI) m/z: [M+Na]+ Calcd for $C_{30}H_{26}N_2O_4Na$, 501.1782; Found, 501.1784. Major: ^{1}H NMR (400 MHz, $CDCl_3$) δ 7.68 (s, 1H), 7.46 (d, *J* = 2.7 Hz, 3H), 7.38 (d, *J* = 9.0 Hz, 1H), 7.20 (dd, *J* = 16.1, 9.0 Hz, 2H), 7.08 – 6.88 (m, 3H), 6.58 (d, *J* = 2.9 Hz, 1H), 6.38 (d, *J* = 8.2 Hz, 1H), 4.37 (s, 1H), 4.14 (t, *J* = 10.0 Hz, 1H), 3.99 (d, *J* = 10.5 Hz, 1H), 3.81 (t, *J* = 10.6 Hz, 1H), 3.73 (d, *J* = 11.6 Hz, 1H), 3.68 (s, 3H), 3.62 (d, *J* = 10.9 Hz, 2H), 3.50 (d, *J* = 9.1 Hz, 3H), 3.44 (dd, *J* = 11.4, 3.6 Hz, 1H); ^{13}C NMR (101 MHz, $CDCl_3$) δ 162.6, 156.3, 143.5, 139.3, 137.5, 137.3, 135.4, 134.8, 132.5, 131.2, 130.0, 129.4, 128.9, 128.8, 128.7, 127.6, 125.9, 124.5, 123.5, 116.4, 114.5, 114.3, 68.6, 67.2, 66.1, 55.3, 53.5, 49.9, 38.6.

Minor: ^{1}H NMR (400 MHz, $CDCl_3$) δ 7.63 – 7.56 (m, 1H), 7.52 – 7.43 (m, 4H), 7.37 (d, *J* = 9.0 Hz, 1H), 7.20 (t, *J* = 7.3 Hz, 1H), 7.03 (dd, *J* = 9.0, 3.0 Hz, 1H), 7.01 – 6.95 (m, 1H), 6.91 (t, *J* = 7.7 Hz, 1H), 6.56 (d, *J* = 2.9 Hz, 1H), 6.31 (d, *J* = 8.1 Hz, 1H), 4.64 (d, *J* = 4.9 Hz, 1H), 4.14 – 4.03 (m, 1H), 3.87 – 3.75 (m, 3H), 3.67 (d, *J* = 5.4 Hz, 3H), 3.63 (s, 1H), 3.55 (dd, *J* = 10.2, 5.5 Hz, 1H), 3.51 – 3.43 (m, 3H), 3.27 (t, *J* = 10.8 Hz, 1H); ^{13}C NMR (101 MHz, $CDCl_3$) δ 162.2, 156.3, 144.3, 139.1, 136.7, 135.9, 135.2, 134.7, 133.9, 130.7, 129.9, 129.6, 129.3, 130.0, 128.8, 127.7, 126.7, 124.4, 123.6, 116.7, 114.4, 114.0, 68.3, 67.3, 66.2, 55.3, 51.4, 49.9, 38.8.

7-1,4-dioxan-2-yl)-2,5-dimethyl-6-oxo-12-phenyl-5,7-dihydrobenzo[*b*]indeno[1,2-*e*]azepine-6a(6*H*)-carbonitrile (4d): A clean vial (5 mL) equipped with a magnetic stir bar was added to **3d** (75 mg, 0.2 mmol, 1.0 equiv), 1,10-phenanthroline (7 mg, 0.04 mmol, 20 mol%), and $NiCl_2$ (3 mg, 0.02 mmol, 10 mol%). Next, 1,4-Dioxane, (3.0 mL) was added after which, TBHP (aq.70%, 51.5 mg, 0.4 mmol, 2.0 equiv), DIPEA (3 mg, 0.02 mmol, 10 mol%) and $(Ir[dF(CF_3)ppy]_2(dtbpy))PF_6$ (2 mg, 0.002 mmol, 1 mol%) were added at room temperature, and then placed at a distance of approx. 3 cm from a 40 W blue LED lamp, and the solution was stirred at room temperature under visible-light irradiation for 4 h. The progress of the reaction was monitored by thin layer chromatography. When the reaction was complete, water was added to quench the reaction mixture, followed by extraction with ethyl acetate (3×10 mL). Finally, the combined organic layer was dried over sodium sulfate, filtered, and concentrated under vacuum. The residue was purified by column chromatography (Hex/EA = 93:7) on silica gel to afford the corresponding **4d** as 2 separable (major/minor) products (yield: 80 mg, 86%, 1.5:1 dr, Off-white solid), m.p.= 253-255 °C; HRMS (ESI) m/z: [M+Na]+ Calcd for $C_{30}H_{26}N_2O_3Na$, 485.1835; Found, 485.1835. ^{1}H NMR (400 MHz, $CDCl_3$) δ 7.70 (dd, *J* = 7.8, 3.0 Hz, 1H), 7.47 (dd, *J* = 5.8, 3.0 Hz, 3H), 7.35 (d, *J* = 8.4 Hz, 1H), 7.31 – 7.25 (m, 2H), 7.20 (d, *J* = 7.7 Hz, 1H), 6.98 – 6.88 (m, 3H), 6.35 (d, *J* = 8.0 Hz, 1H), 4.38 (d, *J* = 2.3 Hz, 1H), 4.18 – 4.11 (m, 1H), 3.98 (dd, *J* = 11.0, 2.0 Hz, 1H), 3.81 (t, *J* = 10.6 Hz, 1H), 3.74 – 3.57 (m, 3H), 3.52 (d, *J* = 8.8 Hz, 3H), 3.47 (dd, *J* = 11.1, 3.8 Hz, 1H), 2.27 (s, 3H); ^{13}C NMR (101 MHz,

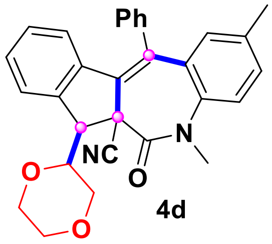

4d

$CDCl_3$) δ 162.7, 143.3, 139.5, 138.8, 137.6, 137.0, 134.9, 134.0, 132.7, 131.3, 130.8, 130.7, 130.0, 129.4, 128.9, 128.7, 128.6, 127.6, 125.9, 124.4, 122.0, 114.5, 68.6, 67.3, 66.1, 53.5, 49.9, 38.5, 21.0.

Minor: ^{1}H NMR (400 MHz, $CDCl_3$) δ 7.64 – 7.57 (m, 1H), 7.48 (dd, *J* = 6.5, 2.9 Hz, 4H), 7.34 (d, *J* = 8.4 Hz, 1H), 7.31 – 7.26 (m, 1H), 7.19 (td, *J* = 7.6, 1.0 Hz, 1H), 6.96 (dd, *J* = 5.6, 2.3 Hz, 1H), 6.93 – 6.86 (m, 2H), 6.27 (d, *J* = 8.1 Hz, 1H), 4.63 (d, *J* = 4.9 Hz, 1H), 4.12 – 4.03 (m, 1H), 3.88 – 3.75 (m, 3H), 3.64 (dd, *J* = 11.4, 2.1 Hz, 1H), 3.55 – 3.52 (m, 3H), 3.51 (d, *J* = 3.7 Hz, 1H), 3.33 – 3.22 (m, 1H), 2.27 (s, 3H); ^{13}C NMR (101 MHz, $CDCl_3$) δ 162.3, 144.2, 139.3, 138.7, 136.3, 136.0, 135.1, 134.1, 133.8, 130.9, 130.7, 130.5, 129.9, 129.5, 129.2, 128.9, 128.7, 127.6, 126.7, 124.3, 122.1, 114.4, 114.3, 68.2, 67.3, 66.2, 51.4, 49.9, 38.7, 23.9, 21.0.

7-1,4-dioxan-2-yl)-2-fluoro-5-methyl-6-oxo-12-phenyl-5,7-dihydrobenzo[*b*]indeno[1,2-*e*]azepine-6*a*(6*H*)-carbonitrile (4e): A clean vial (5 mL) equipped with a magnetic stir bar was added to **3e** (76 mg, 0.2 mmol, 1.0 equiv), 1,10-phenanthroline (7 mg, 0.04 mmol, 20 mol%), and $NiCl_2$ (3 mg, 0.02 mmol, 10 mol%). Next, 1,4-Dioxane, (3.0 mL) was added after which, TBHP (aq.70%, 51.5 mg, 0.4 mmol, 2.0 equiv), DIPEA (3 mg, 0.02 mmol, 10 mol%) and (Ir[dF(CF_3)ppy]$_2$(dtbpy))PF_6 (2 mg, 0.002 mmol, 1 mol%) were added at room temperature, and then placed at a distance of approx. 3 cm from a 40 W blue LED lamp, and the solution was stirred at room temperature under visible-light irradiation for 4 h. The progress of the reaction was monitored by thin layer chromatography. When the reaction was complete, water was added to quench the reaction mixture, followed by extraction with ethyl acetate (3×10 mL). Finally, the combined organic layer was dried over sodium sulfate, filtered, and concentrated under vacuum. The residue was purified by column chromatography (Hex/EA = 93:7) on silica gel to afford the corresponding **4e** as 2 separable (major/minor) products (yield: 67 mg, 72%, 1.2:1 dr, Off-white solid), m.p.= 253-255 °C; HRMS (ESI) m/z: [M+Na]+ Calcd for $C_{29}H_{23}N_2O_3FNa$, 489.1582; Found, 489.1584. ^{1}H NMR (400 MHz, $CDCl_3$) δ 7.72 – 7.64 (m, 1H), 7.51 – 7.41 (m, 4H), 7.27 – 7.15 (m, 3H), 6.95 (ddd, *J* = 14.7, 5.9, 1.4 Hz, 2H), 6.83 (dd, *J* = 9.6, 3.0 Hz, 1H), 6.40 (d, *J* = 8.0 Hz, 1H), 4.38 (d, *J* = 2.3 Hz, 1H), 4.14 (dt, *J* = 10.2, 2.2 Hz, 1H), 3.99 (dd, *J* = 10.9, 1.9 Hz, 1H), 3.80 (t, *J* = 10.6 Hz, 1H), 3.66 (ddd, *J* = 22.9, 12.2, 2.4 Hz, 3H), 3.52 (d, *J* = 9.1 Hz, 3H), 3.46 (dd, *J* = 11.1, 4.0 Hz, 1H); ^{13}C NMR (101 MHz, $CDCl_3$) δ 162.6, 160.4 (J_{C-F} = 245.38 Hz), 157.9, 143.6, 143.6, 138.8, 138.2, 138.1, 137.3, 137.2, 136.1, 131.6, 131.2, 130.7, 129.9, 129.5, 129.1, 129.0, 127.7, 125.9, 125.1, 124.6, 124.1 (J_{C-F} = 8.59 Hz), 117.3 (J_{C-F} = 23.28 Hz), 116.6 (J_{C-F} = 22.89 Hz), 114.3, 72.9, 68.8, 68.5, 67.2, 66.1, 62.7, 53.5, 50.0, 38.7.

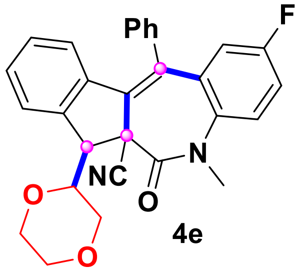

4e

Minor: ^{1}H NMR (400 MHz, $CDCl_3$) δ 7.64 – 7.55 (m, 1H), 7.53 – 7.39 (m, 5H), 7.28 – 7.14 (m, 2H), 6.95 (ddd, *J* = 19.5, 10.1, 5.0 Hz, 2H), 6.80 (dd, *J* = 9.6, 2.9 Hz, 1H), 6.32 (d, *J* = 8.0 Hz, 1H), 4.65 (d, *J* = 4.9 Hz, 1H), 4.07 (ddd, *J* = 10.1, 5.0, 2.4 Hz, 1H), 3.87 – 3.72 (m, 3H), 3.69 – 3.60 (m, 1H), 3.59 – 3.53 (m, 1H), 3.53 – 3.47 (m, 3H), 3.30 (t, *J* = 10.8 Hz, 1H); ^{13}C NMR (101 MHz, $CDCl_3$) δ 162.2, 160.4, (J_{C-F} = 245.75 Hz), 157.9, 144.4,

138.6, 137.6, 137.2, 135.9, 135.7, 130.7, 129.8 ($J_{C\text{-}F}$ = 7.57 Hz), 129.6, 129.1, 129.1, 127.8, 126.7, 124.5, 124.2 ($J_{C\text{-}F}$ = 8.57 Hz), 117.5 ($J_{C\text{-}F}$ = 23.3 Hz), 116.4 ($J_{C\text{-}F}$ = 22.98 Hz), 114.2, 68.2, 67.3, 66.1, 51.4, 50.0, 38.9.

12-(4-cyanophenyl)-7-1,4-dioxan-2-yl)-5-methyl-6-oxo-5,7-dihydrobenzo[*b*]indeno[1,2-e]azepine-6*a*(6*H*)-

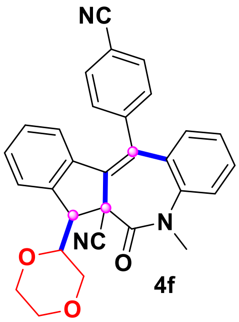

carbonitrile (4f): A clean vial (5 mL) equipped with a magnetic stir bar was added to **3f** (69 mg, 0.2 mmol, 1.0 equiv), 1,10-phenanthroline (8 mg, 0.04 mmol, 20 mol%), and $NiCl_2$ (3 mg, 0.02 mmol, 10 mol%). Next, 1,4-Dioxane, (3.0 mL) was added after which, TBHP (aq.70%, 51.5 mg, 0.4 mmol, 2.0 equiv), DIPEA (3 mg, 0.02 mmol, 10 mol%) and $(Ir[dF(CF_3)ppy]_2(dtbpy))PF_6$ (2 mg, 0.002 mmol, 1 mol%) were added at room temperature, and then placed at a distance of approx. 3 cm from a 40 W blue LED lamp, and the solution was stirred at room temperature under visible-light irradiation for 4 h. The progress of the reaction was monitored by thin layer chromatography. When the reaction was complete, water was added to quench the reaction mixture, followed by extraction with ethyl acetate (3×10 mL). Finally, the combined organic layer was dried over sodium sulfate, filtered, and concentrated under vacuum. The residue was purified by column chromatography (Hex/EA = 93:7) on silica gel to afford the corresponding **4f** as 2 separable (major/minor) products (yield: 76.6 mg, 81%, 1:1 dr, Off-white solid), m.p.= 274.8-276 °C; HRMS (ESI) m/z: [M+H] + Calcd for $C_{30}H_{23}N_3O_3$, 474.1739; Found, 474.1739. ^{1}H NMR (400 MHz, $CDCl_3$) δ 7.92 – 7.84 (m, 1H), 7.76 (d, *J* = 8.6 Hz, 2H), 7.56 – 7.44 (m, 2H), 7.28 (d, *J* = 6.6 Hz, 1H), 7.26 – 7.19 (m, 2H), 7.17 – 7.10 (m, 1H), 7.08 – 6.94 (m, 2H), 6.39 (d, *J* = 8.0 Hz, 1H), 4.38 (d, *J* = 2.3 Hz, 1H), 4.13 (d, *J* = 10.2 Hz, 1H), 4.00 (d, *J* = 11.0 Hz, 1H), 3.79 (t, *J* = 10.6 Hz, 1H), 3.74 – 3.60 (m, 3H), 3.55 (d, *J* = 8.9 Hz, 3H), 3.45 (td, *J* = 11.2, 3.6 Hz, 1H); ^{13}C NMR (101 MHz, $CDCl_3$) δ 162.6, 144.3, 144.0, 141.2, 138.7, 136.7, 133.2, 133.2, 132.6, 132.2, 131.2, 130.5, 130.3, 130.0, 129.5, 127.8, 126.2, 125.5, 124.2, 122.4, 118.5, 114.0, 112.5, 77.3, 68.5, 67.3, 66.1, 50.0, 38.6.

Minor: ^{1}H NMR (400 MHz, $CDCl_3$) δ 7.78 (d, *J* = 9.8 Hz, 3H), 7.56 – 7.44 (m, 3H), 7.26 – 7.19 (m, 2H), 7.13 (d, *J* = 7.9 Hz, 1H), 7.05 – 6.92 (m, 2H), 6.31 (d, *J* = 8.1 Hz, 1H), 4.64 (d, *J* = 4.9 Hz, 1H), 4.06 (ddd, *J* = 10.3, 4.9, 2.5 Hz, 1H), 3.79 (ddd, *J* = 10.3, 8.9, 2.6 Hz, 3H), 3.63 (s, 1H), 3.56 (s, 3H), 3.53 (d, *J* = 3.2 Hz, 1H), 3.37 – 3.28 (m, 1H); ^{13}C NMR (101 MHz, $CDCl_3$) δ 162.1, 145.0, 144.1, 141.1, 138.0, 135.1, 133.4, 133.1, 132.7, 131.7, 131.2, 130.3, 130.3, 130.0, 128.0, 127.0, 125.6, 124.1, 122.5, 118.4, 113.9, 112.7, 77.3, 68.2, 67.3, 66.1, 50.0, 38.8, 23.9.

methyl 4-6*a*-cyano-7-1,4-dioxan-2-yl)-5-methyl-6-oxo-5,6,6*a*,7-tetrahydrobenzo[*b*]indeno[1,2-*e*]azepin-12-yl)benzoate (4g): A clean vial (5 mL) equipped with a magnetic stir bar was added to **3g** (84 mg, 0.2 mmol, 1.0 equiv), 1,10-phenanthroline (7 mg, 0.04 mmol, 20 mol%), and $NiCl_2$ (3 mg, 0.02 mmol, 10 mol%). Next, 1,4-Dioxane, (3.0 mL) was added after which, TBHP (aq.70%, 51.5 mg, 0.4 mmol, 2.0 equiv), DIPEA (3 mg, 0.02 mmol, 10 mol%) and (Ir[dF(CF_3)ppy]$_2$(dtbpy))PF_6 (2 mg, 0.002 mmol, 1 mol%) were added at room temperature, and then placed at a distance of approx. 3 cm from a 40 W blue LED lamp, and the solution was stirred at room temperature under visible-light irradiation for 4 h. The progress of the reaction was monitored by thin layer chromatography. When the reaction was complete, water was added to quench the reaction mixture, followed by extraction with ethyl acetate (3×10 mL). Finally, the combined organic layer was dried over sodium sulfate, filtered, and concentrated under vacuum. The residue was purified by column chromatography (Hex/EA = 93:7) on silica gel to afford the corresponding **4g** as 2 separable (major/minor) products (yield: 83 mg, 82%, 1.1:1 dr, Off-white solid), m.p.= 284-286 °C; HRMS (ESI) m/z: [M+Na]+ Calcd for $C_{31}H_{28}N_2O_5Na$, 531.1889; Found, 531.1890. ^{1}H NMR (400 MHz, $CDCl_3$) δ 8.21 – 8.08 (m, 2H), 7.86 – 7.76 (m, 1H), 7.52 – 7.44 (m, 2H), 7.27 – 7.18 (m, 3H), 7.07 (dd, *J* = 11.2, 4.7 Hz, 2H), 6.94 (t, *J* = 7.0 Hz, 1H), 6.42 (d, *J* = 8.0 Hz, 1H), 4.39 (d, *J* = 2.3 Hz, 1H), 4.14 (dt, *J* = 10.2, 2.2 Hz, 1H), 4.01 (d, *J* = 1.9 Hz, 1H), 3.98 (s, 3H), 3.80 (t, *J* = 10.6 Hz, 1H), 3.74 – 3.60 (m, 3H), 3.56 (d, *J* = 8.9 Hz, 3H), 3.47 (dd, *J* = 11.1, 4.1 Hz, 1H); ^{13}C NMR (101 MHz, $CDCl_3$) δ 166.7, 162.7, 144.2, 143.7, 141.1, 138.0, 137.0, 133.7, 131.4, 131.3, 130.7, 130.6, 130.3, 130.1, 129.8, 129.2, 127.7, 126.0, 125.4, 124.4, 122.3, 114.3, 114.2, 77.3, 68.5, 67.2, 66.1, 53.7, 52.3, 49.9, 38.6.

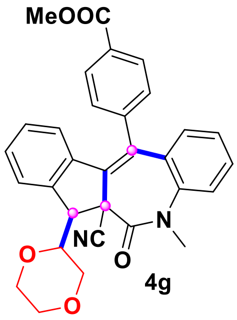

Minor: ^{1}H NMR (400 MHz, $CDCl_3$) δ 8.20 – 8.10 (m, 2H), 7.73 (dd, *J* = 8.3, 1.5 Hz, 1H), 7.53 – 7.45 (m, 3H), 7.25 – 7.15 (m, 2H), 7.11 – 7.02 (m, 2H), 6.92 (t, *J* = 7.7 Hz, 1H), 6.34 (d, *J* = 8.1 Hz, 1H), 4.65 (d, *J* = 4.9 Hz, 1H), 4.07 (ddd, *J* = 10.2, 5.0, 2.4 Hz, 1H), 3.98 (s, 3H), 3.87 – 3.74 (m, 3H), 3.68 – 3.61 (m, 1H), 3.57 (d, *J* = 7.3 Hz, 3H), 3.55 – 3.48 (m, 1H), 3.36 – 3.26 (m, 1H); ^{13}C NMR (101 MHz, $CDCl_3$) δ 166.6, 162.2, 144.6, 143.9, 141.0, 137.3, 135.4, 133.5, 132.8, 130.9, 130.8, 130.5, 130.4, 130.3, 130.1, 130.0, 129.7, 127.8, 126.8, 125.4, 124.3, 122.4, 114.1, 77.3, 68.2, 67.3, 66.1, 52.3, 51.5, 49.9, 38.8.

10-hydroxy-5-phenyl-10,11-dihydro-10a*H*-benzo[*b*]fluorene-10*a*-carbonitrile (5): To a 25 mL Schlenk tube with a magnetic bar were added **2r** (67 mg, 0.2 mmol, 1.0 equiv) in MeOH (2.0 mL) and $NaBH_4$ (23 mg, 0.6 mmol, 3.0 equiv.) at 0 °C. Then the reaction was warmed to r.t. The reaction was monitored by TLC until completion (6h). The mixture was washed with water (5 mL), extracted with EtOAc (3×10 mL), dried over Na_2SO_4 and concentrated in vacuo. The crude product was purified by column chromatography (silica gel, EtOAc/petroleum ether = 2:1) to afford **5** as white solid (yield: 63 mg, 94%, m.p. 241-243°C; HRMS (ESI) m/z: [M+H]+ Calcd for $C_{24}H_{18}NO$, 336.1382; Found, 336.1385. ^{1}H NMR (597 MHz, $CDCl_3$) δ 7.74 (d, *J* = 7.6 Hz, 1H), 7.50 (dt, *J* = 17.2, 7.7 Hz, 4H), 7.38 (ddd, *J* = 16.6, 11.5, 4.3 Hz, 2H), 7.28 – 7.21 (m, 3H), 6.98 (t, *J* = 7.7

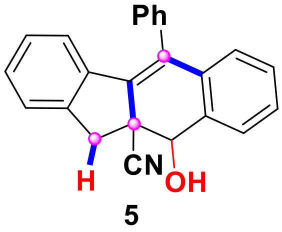

Hz, 1H), 6.91 (dd, *J* = 7.8, 1.1 Hz, 1H), 6.39 (d, *J* = 7.9 Hz, 1H), 4.98 (s, 1H), 3.78 (d, *J* = 16.5 Hz, 1H), 3.42 (d, *J* = 16.5 Hz, 1H); ^{13}C NMR (150 MHz, $CDCl_3$) δ 143.4, 137.1, 136.8, 135.5, 135.3, 135.2, 134.1, 129.2, 128.4, 128.4, 128.2, 127.5, 127.3, 125.3, 124.6, 123.5, 120.3, 109.9, 75.9, 48.7, 41.5.

6. Reference

1. a) Gore, B. S.; Chiang, C.-H.; Lee, C. C.; Shih, Y.-L.; Wang J.-J. De Novo Protocol for the Construction of Benzo[*a*]fluorenes via Nitrile/Alkene Activation. *Org. Lett*. **2020**, *22*, 7848-7852. b) Gore B. S.; Lin J.-H.; Wang, J.-J. Unraveling innate substrate-controlled arylation and bicyclization of 1,5-enynes with α,β conjugates: synthesis of substituted benzo[*a*]fluorenes. *Green Chem*., **2021**, *23*, 4144; (c) Gore, B. S.; Kuo, C.-Y. Wang, J.-J. Visible light-assisted Ni-/Ir-catalysed atom-economic synthesis of spiro[furan-3,10 -indene] derivatives. *Chem. Commun*., **2022**, *58*, 4087.

7. Crystal data for compound 2a (major/minor) and 2s (ellipsoid contour 50% probability levels)

The crystal of **2a (major/minor),** and **2s** were obtained by crystallization from a solution in ethyl acetate (**2a**) and chloroform (**2s**) after purification by column chromatography respectively. The instrumentation used for the crystal measurement is Rigaku Oxford XtaLab Proll. The crystallographic data were deposited with the Cambridge Crystallographic Data Centre as supplementary publication with CCDC numbers: **2a** ((major: 2226549), (minor: 2226550)), **2s** (2226770).

8. Plausible mechanism of azepine formation

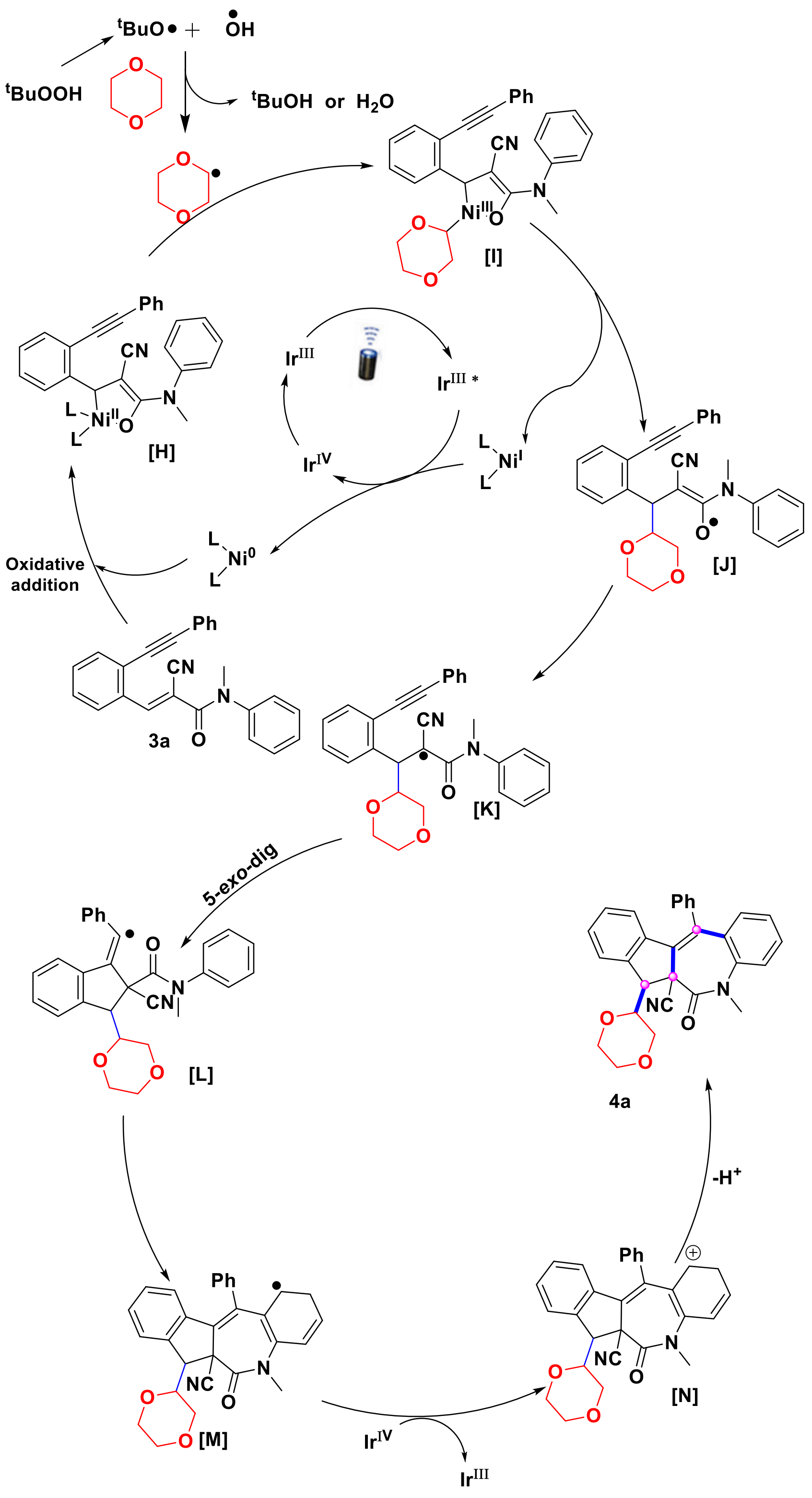

Solvent CDCl3
Spectrometer Frequency 400

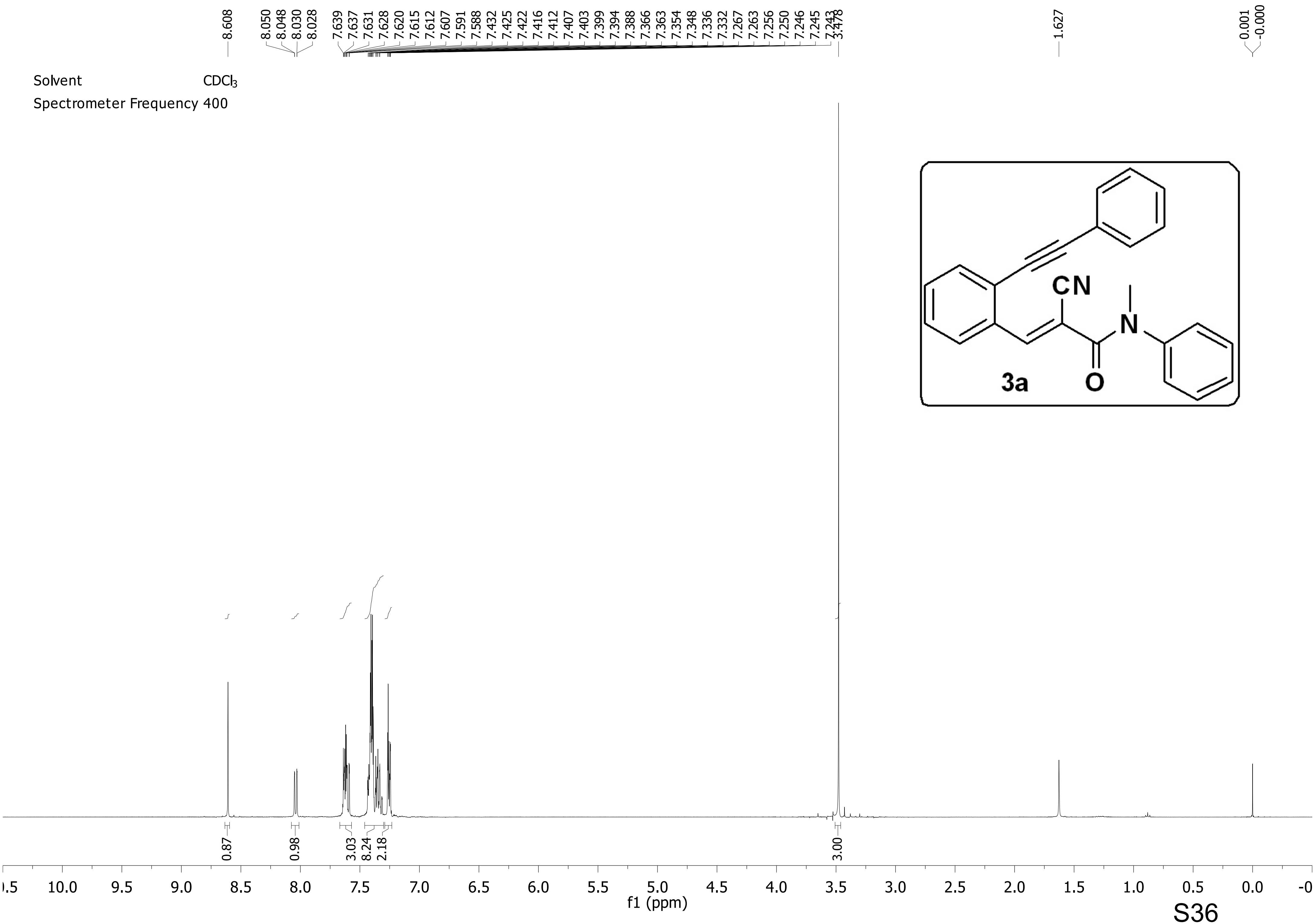

Solvent CDCl3

Spectrometer Frequency 100

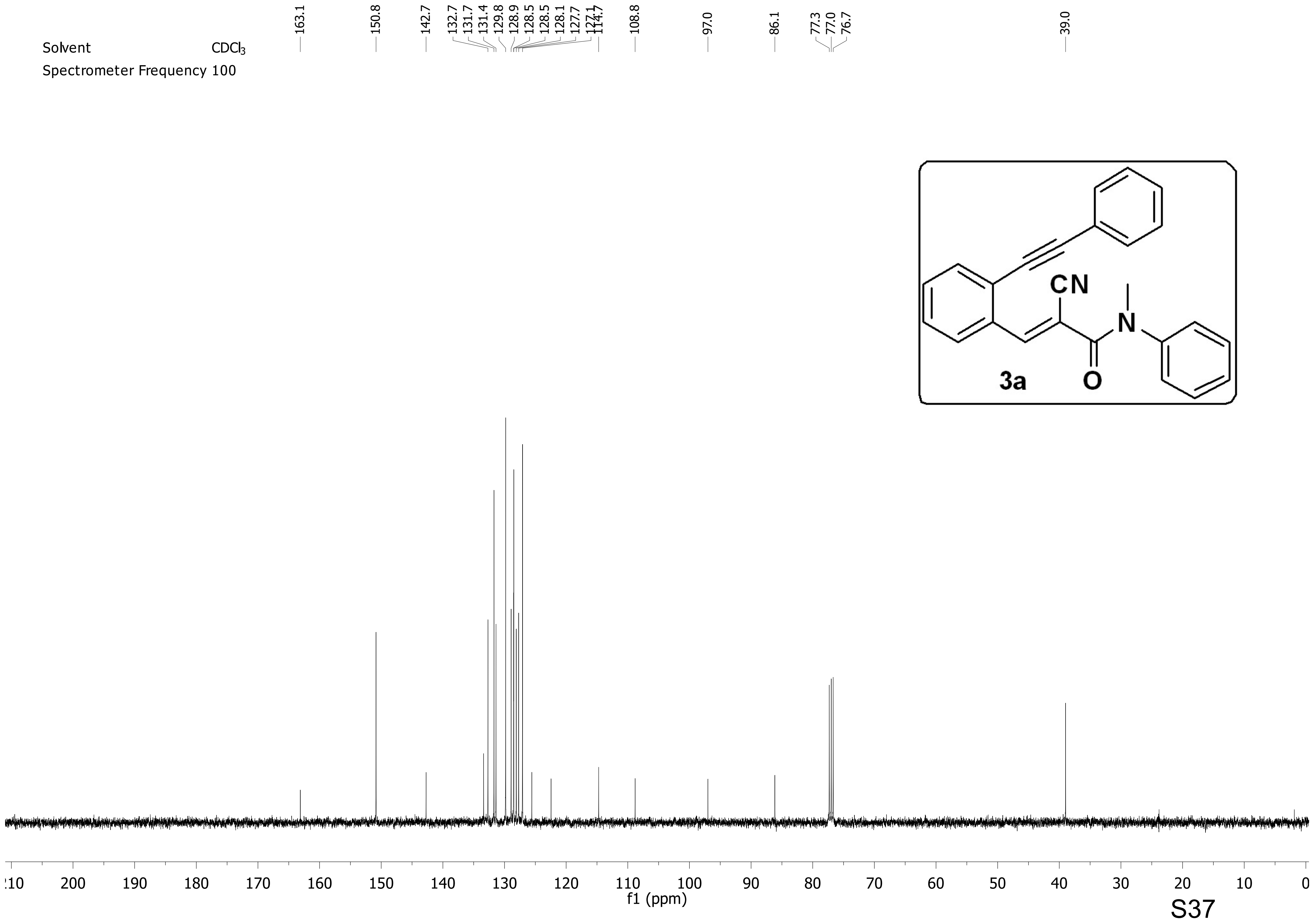

Solvent CDCl3
Spectrometer Frequency 400

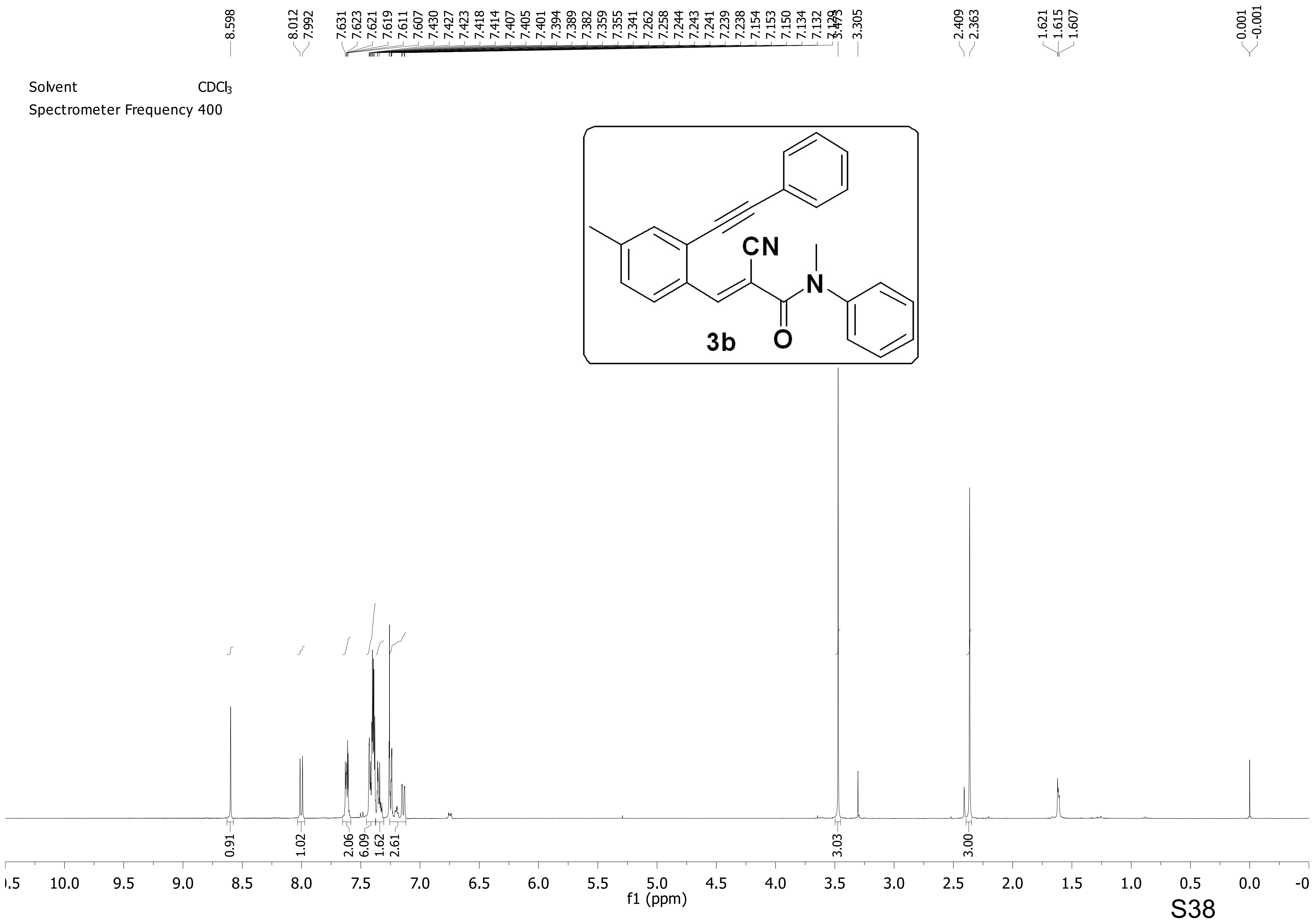

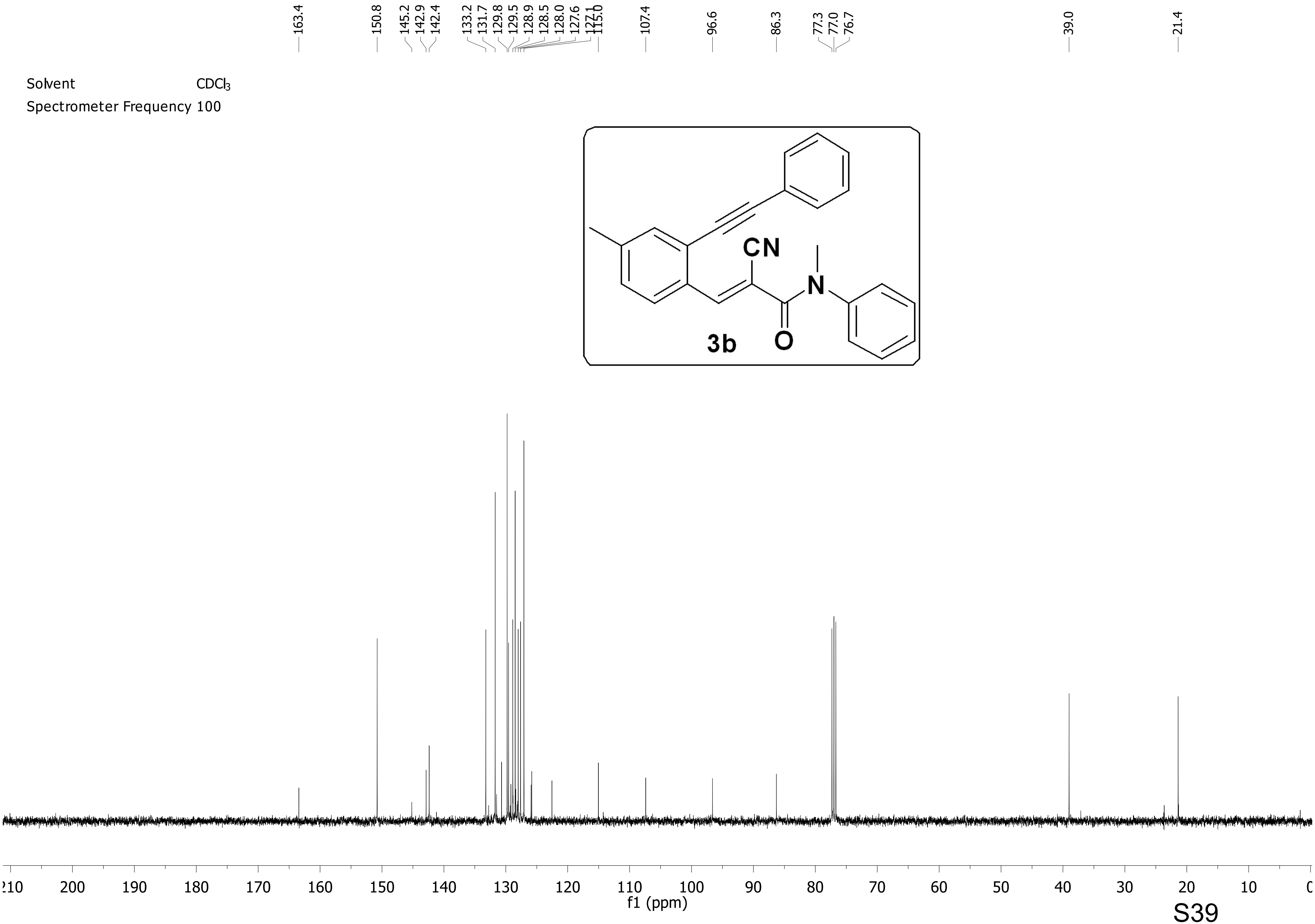
163.4
150.8
145.2
142.9
142.4
133.2
131.7
129.8
129.5
128.9
128.5
128.0
127.6
127.1
115.0
107.4
96.6
86.3
77.3
77.0
76.7
39.0
21.4
Solvent CDCl3
Spectrometer Frequency 100
CN
N
O
3b
210
200
190
180
170
160
150
140
130
120
110
100
90
80
70
60
50
40
30
20
10
0
f1 (ppm)

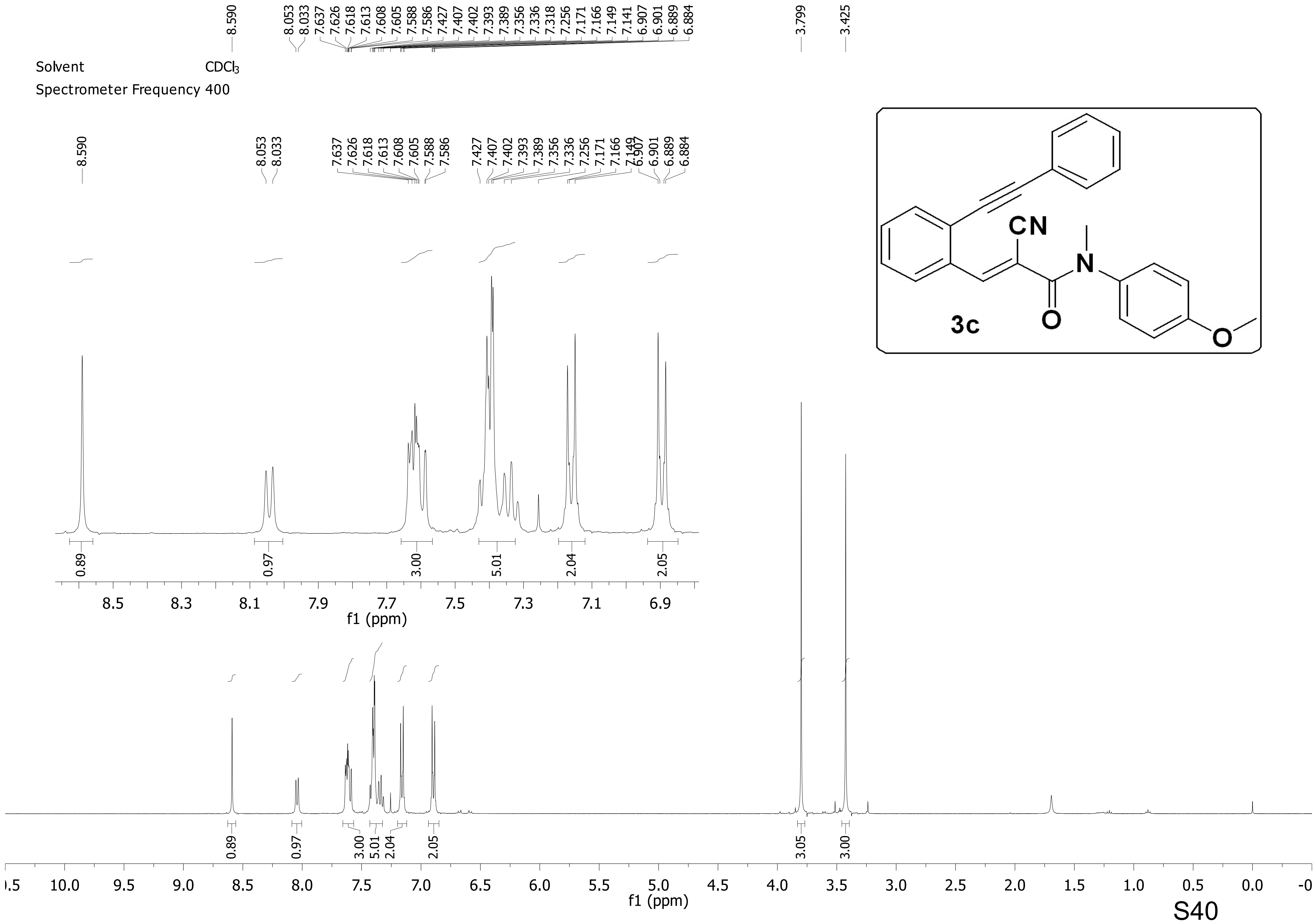
Solvent CDCl3
Spectrometer Frequency 400
8.590
8.053
8.033
7.637
7.626
7.618
7.613
7.608
7.605
7.588
7.586
7.427
7.407
7.402
7.393
7.389
7.356
7.336
7.318
7.256
7.171
7.166
7.149
7.141
6.907
6.901
6.889
6.884
3.799
3.425
0.89
0.97
3.00
5.01
2.04
2.05
3.05
3.00
f1 (ppm)
CN
N
O
O

3c

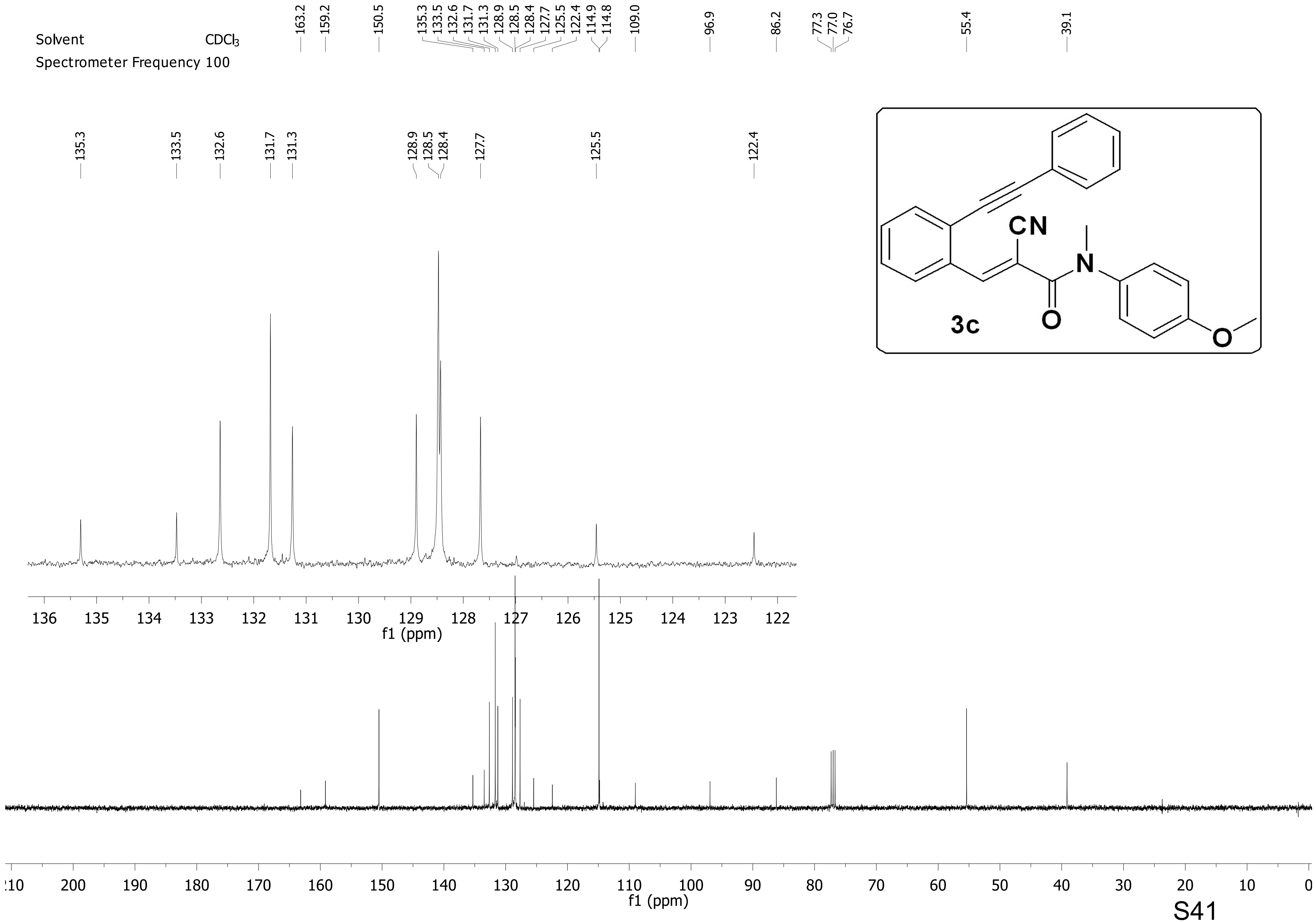
Solvent CDCl3
Spectrometer Frequency 100
163.2 159.2 150.5 135.3 133.5 132.6 131.7 131.3 128.9 128.5 128.4 127.7 125.5 122.4 114.9 114.8 109.0 96.9 86.2 77.3 77.0 76.7 55.4 39.1
135.3 133.5 132.6 131.7 131.3 128.9 128.5 128.4 127.7 125.5 122.4
136 135 134 133 132 131 130 129 128 127 126 125 124 123 122
f1 (ppm)
CN
N
O
3c
210 200 190 180 170 160 150 140 130 120 110 100 90 80 70 60 50 40 30 20 10 0
f1 (ppm)

Solvent CDCl$_3$
Spectrometer Frequency 400

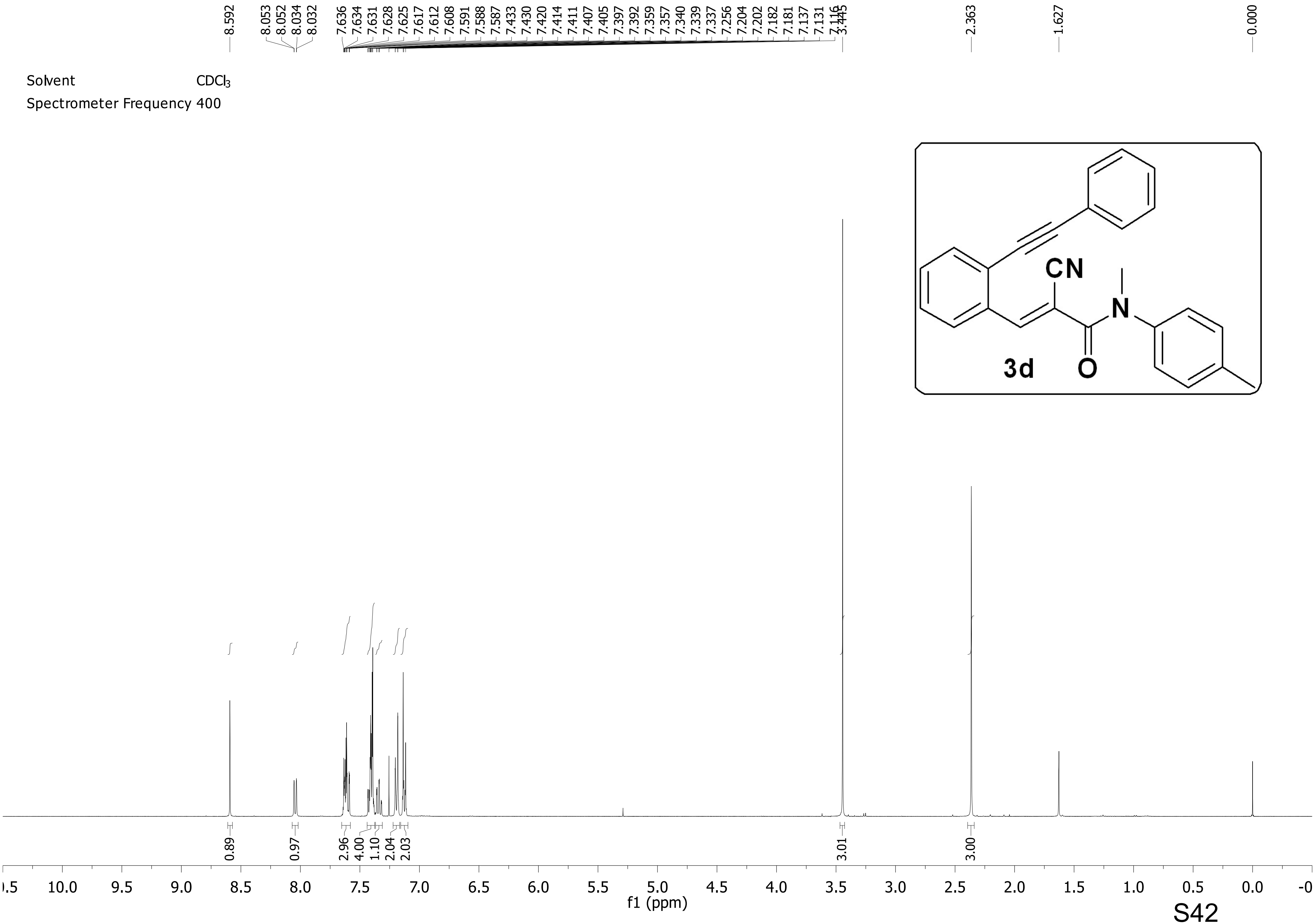

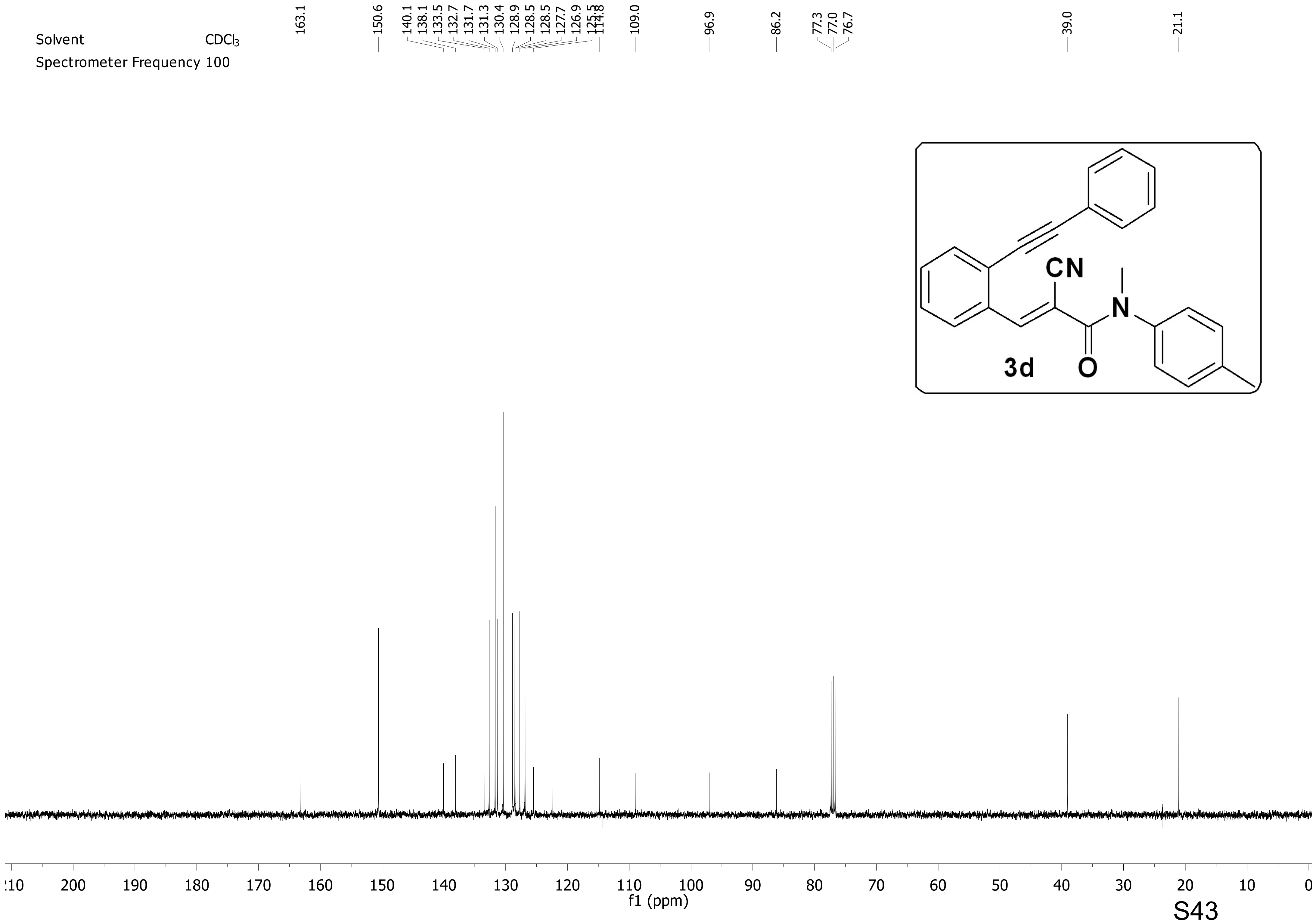
Solvent CDCl3
Spectrometer Frequency 100
163.1
150.6
140.1
138.1
133.5
132.7
131.7
131.3
130.4
128.9
128.5
128.5
127.7
126.9
125.5
114.8
109.0
96.9
86.2
77.3
77.0
76.7
39.0
21.1
CN
N
O
3d
f1 (ppm)

Solvent CDCl$_3$
Spectrometer Frequency 400

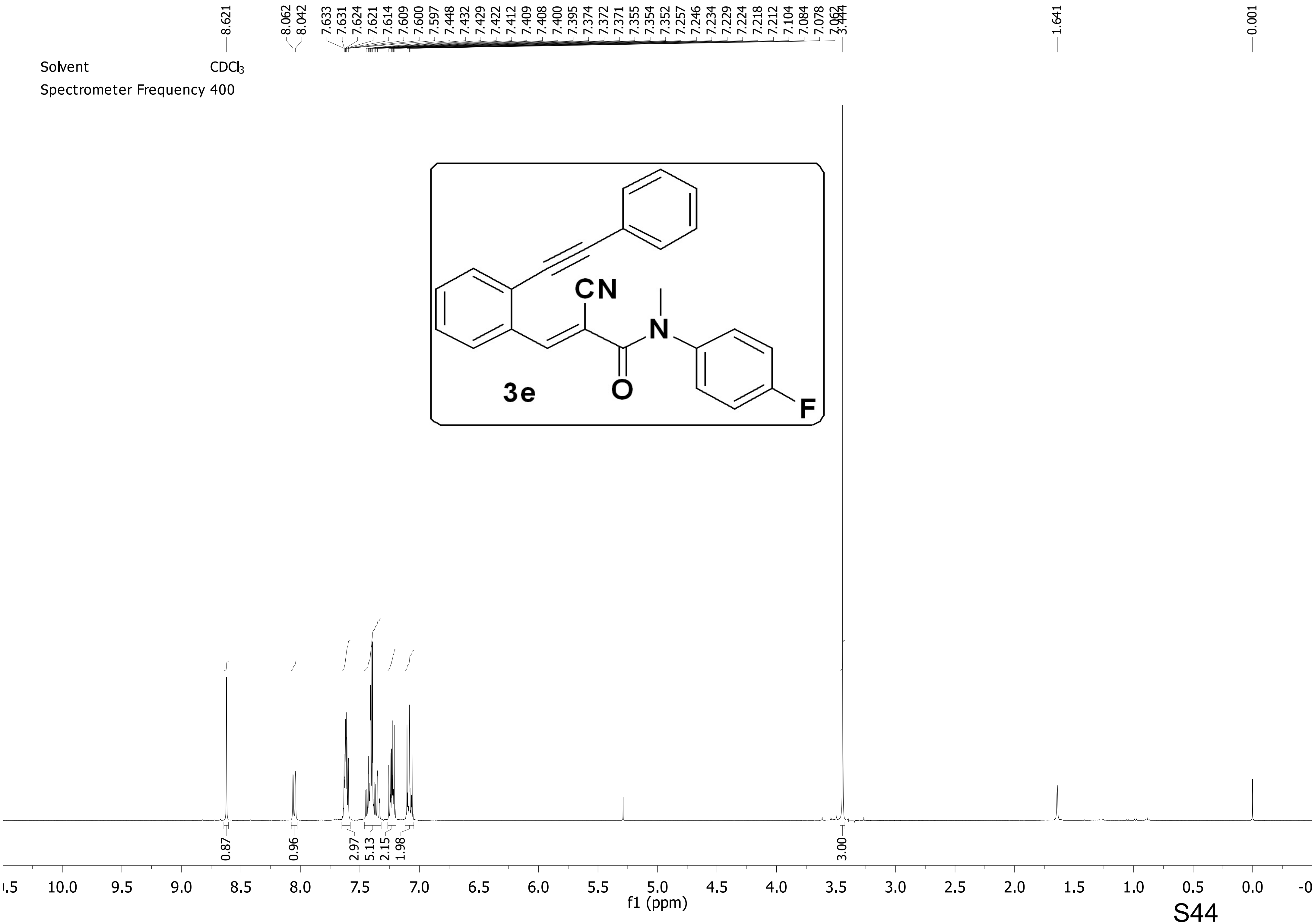

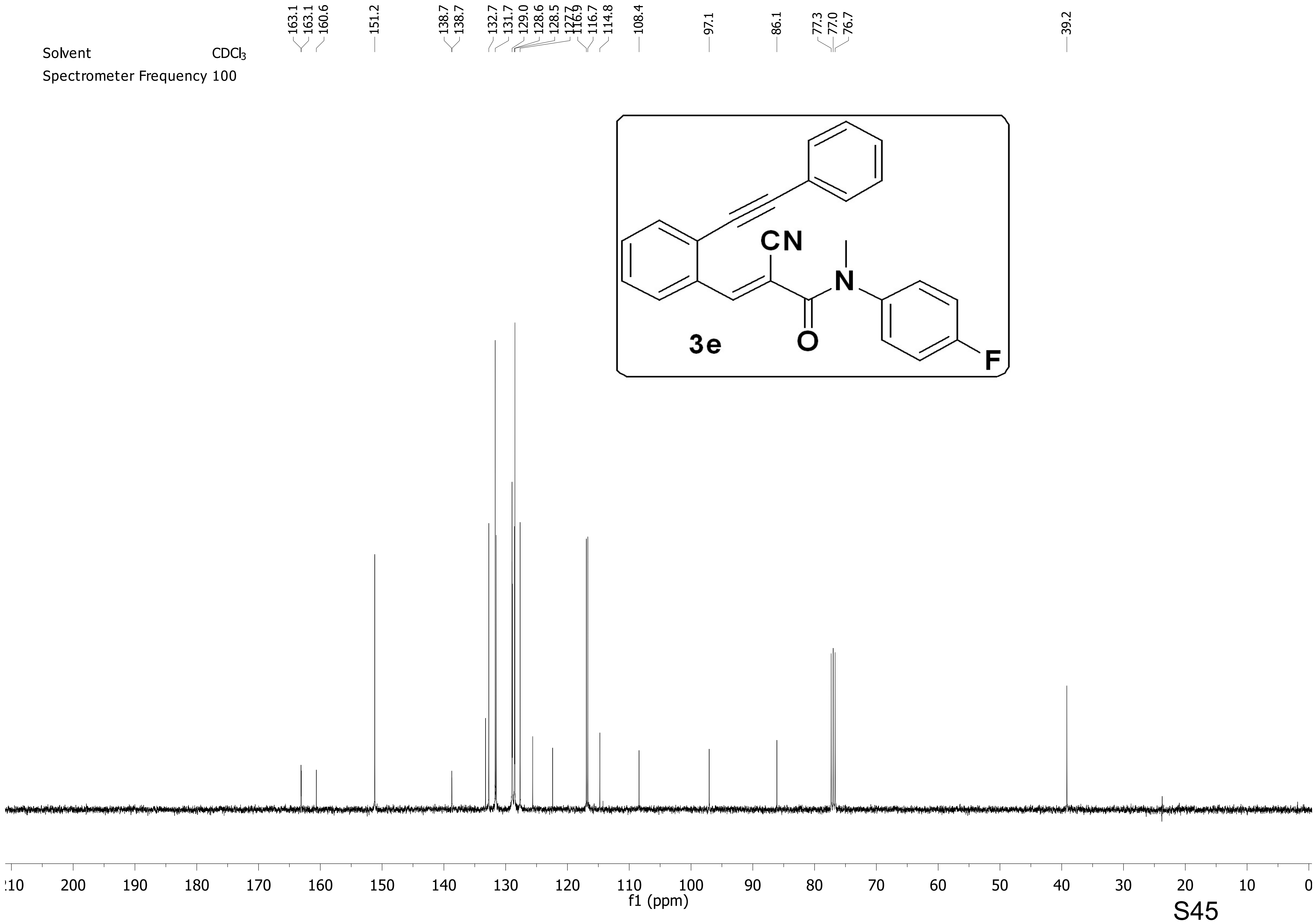
Solvent CDCl3
Spectrometer Frequency 100
163.1
163.1
160.6
151.2
138.7
138.7
132.7
131.7
129.0
128.6
128.5
127.7
116.9
116.7
114.8
108.4
97.1
86.1
77.3
77.0
76.7
39.2
CN
N
O
F
3e
210
200
190
180
170
160
150
140
130
120
110
100
90
80
70
60
50
40
30
20
10
0
f1 (ppm)

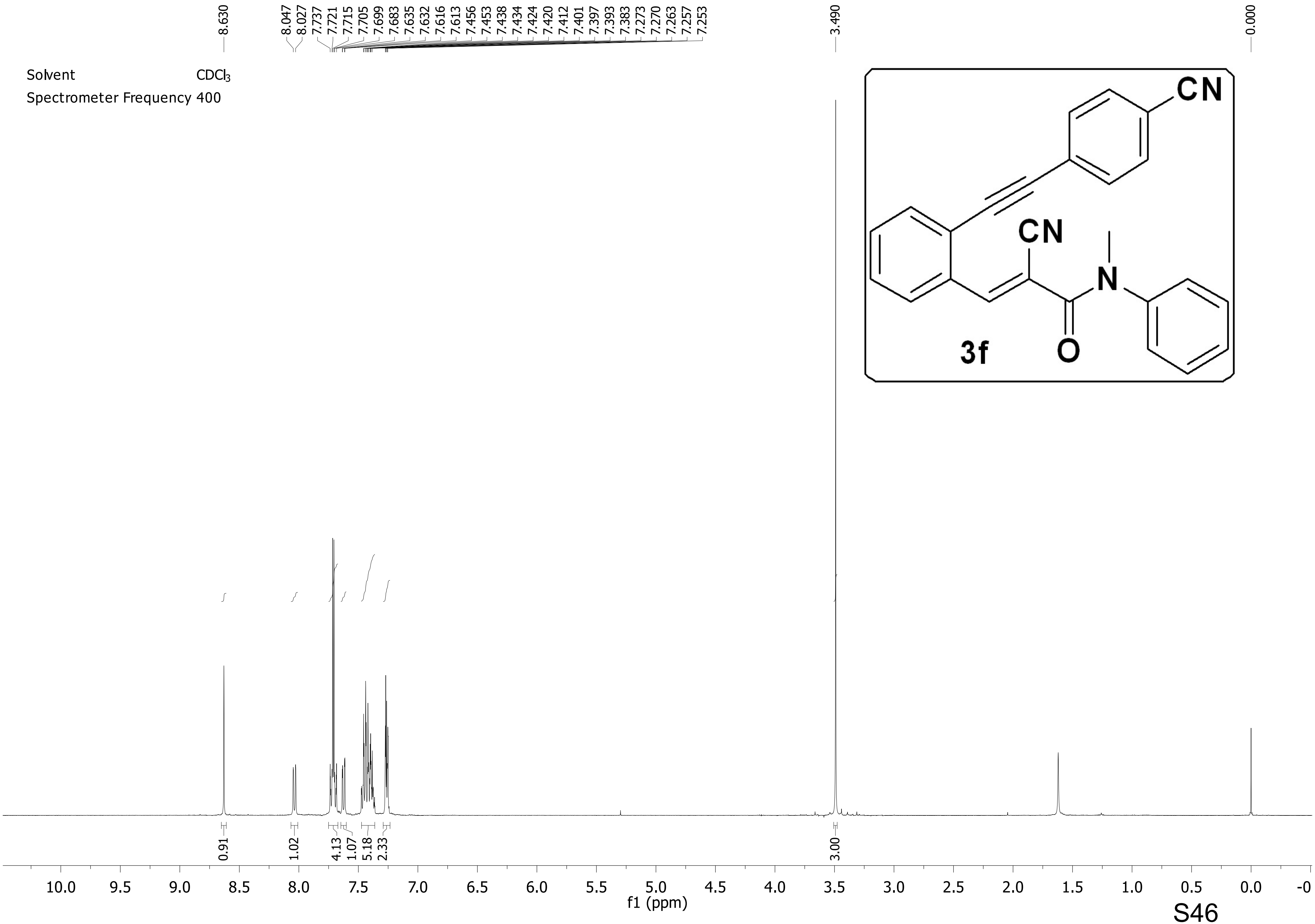

Solvent CDCl$_3$
Spectrometer Frequency 400
8.630
8.047
8.027
7.737
7.721
7.715
7.705
7.699
7.683
7.635
7.632
7.616
7.613
7.456
7.453
7.438
7.434
7.424
7.420
7.412
7.401
7.397
7.393
7.383
7.273
7.270
7.263
7.257
7.253
3.490
0.000
CN
CN
N
O
3f
0.91
1.02
4.13
1.07
5.18
2.33
3.00
10.0
9.5
9.0
8.5
8.0
7.5
7.0
6.5
6.0
5.5
5.0
4.5
4.0
3.5
3.0
2.5
2.0
1.5
1.0
0.5
0.0
-0
f1 (ppm)

Solvent CDCl3
Spectrometer Frequency 100

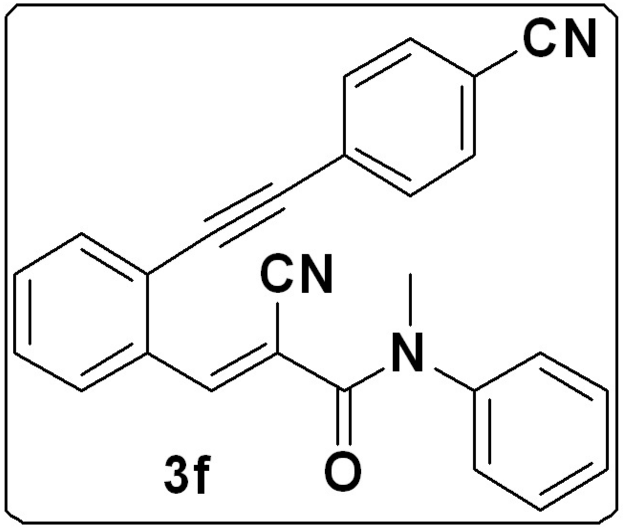

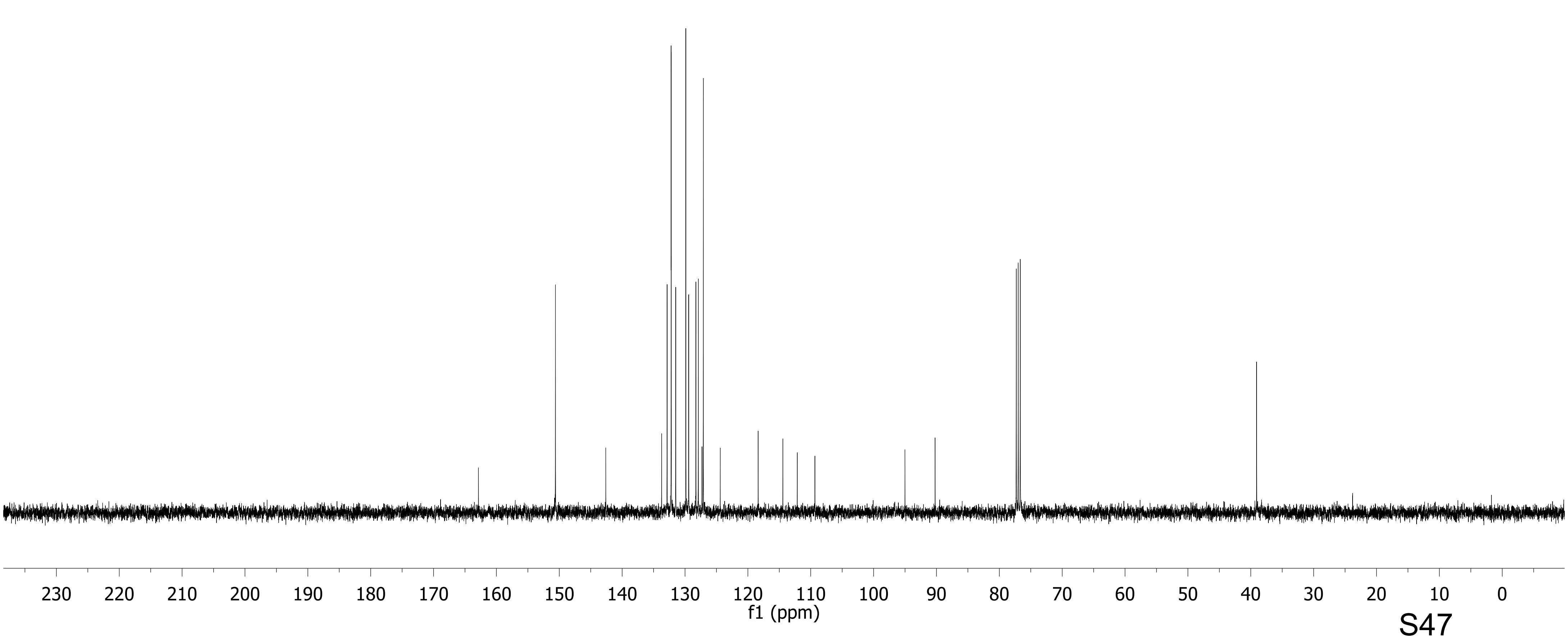

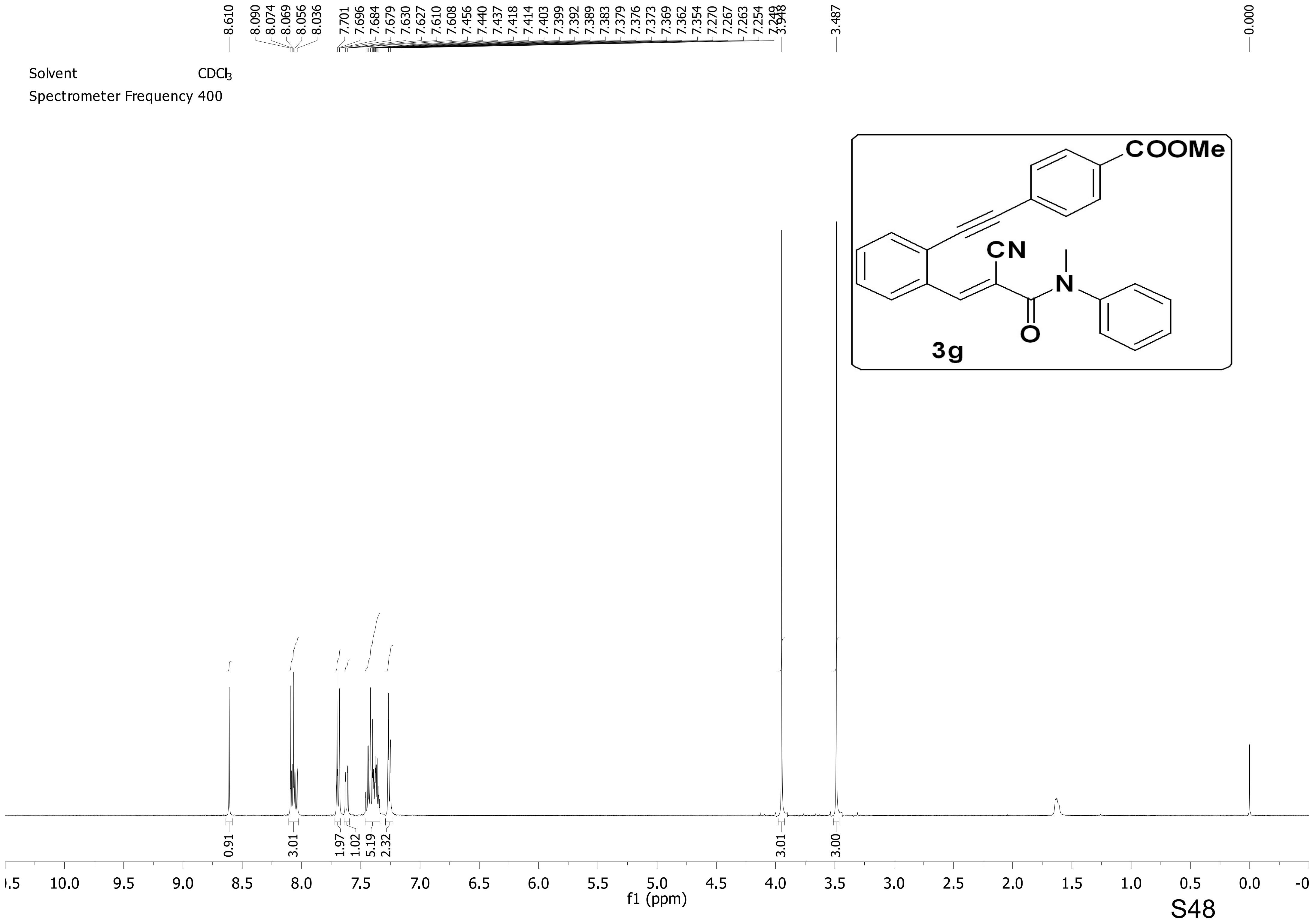
8.610
8.090
8.074
8.069
8.056
8.036
7.701
7.696
7.684
7.679
7.630
7.627
7.610
7.608
7.456
7.440
7.437
7.418
7.414
7.403
7.399
7.392
7.389
7.383
7.379
7.376
7.373
7.369
7.362
7.354
7.270
7.267
7.263
7.254
7.249
3.948
3.487
0.000
Solvent CDCl3
Spectrometer Frequency 400
COOMe
CN
N
O
3g
0.91
3.01
1.97
1.02
5.19
2.32
3.01
3.00
f1 (ppm)

Solvent CDCl$_3$
Spectrometer Frequency 100

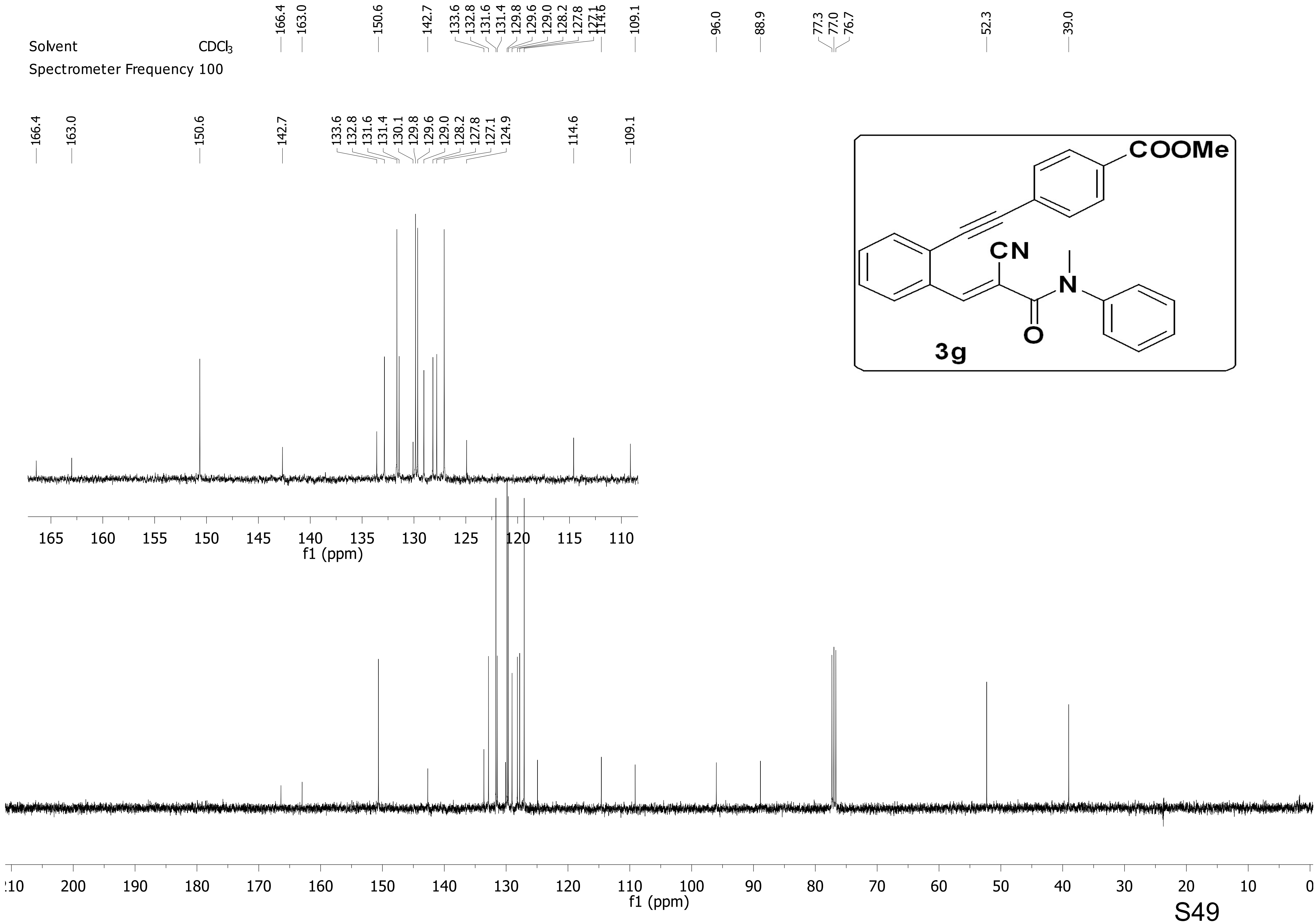

3g

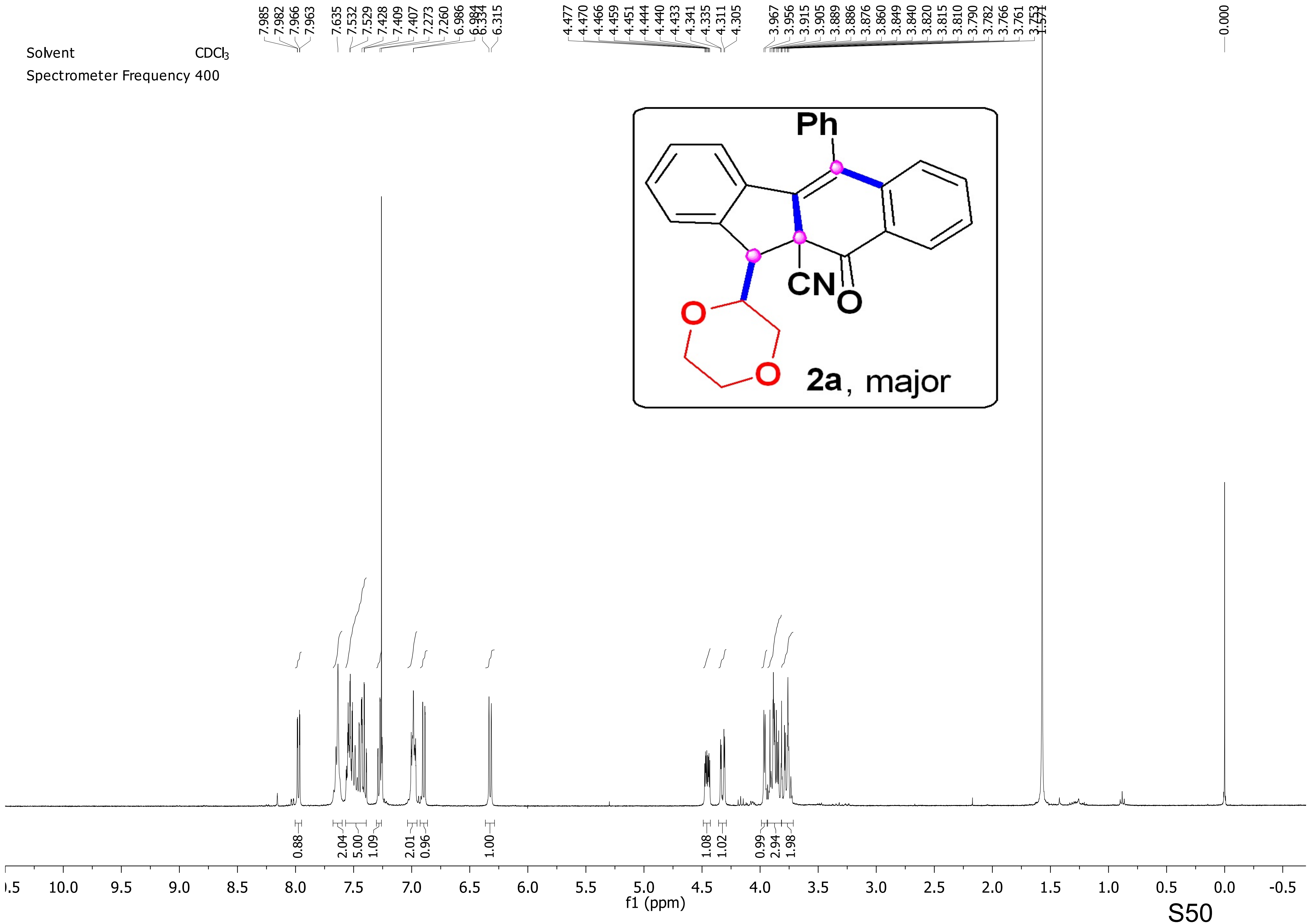
Solvent CDCl3
Spectrometer Frequency 400
7.985
7.982
7.966
7.963
7.635
7.532
7.529
7.428
7.409
7.407
7.273
7.260
6.986
6.315
4.477
4.470
4.466
4.459
4.451
4.444
4.440
4.433
4.341
4.335
4.311
4.305
3.967
3.956
3.915
3.905
3.889
3.886
3.876
3.860
3.849
3.840
3.820
3.815
3.810
3.790
3.782
3.766
3.761
1.571
0.000
Ph
CN
O
2a, major
0.88
2.04
5.00
1.09
2.01
0.96
1.00
1.08
1.02
0.99
2.94
1.98
f1 (ppm)

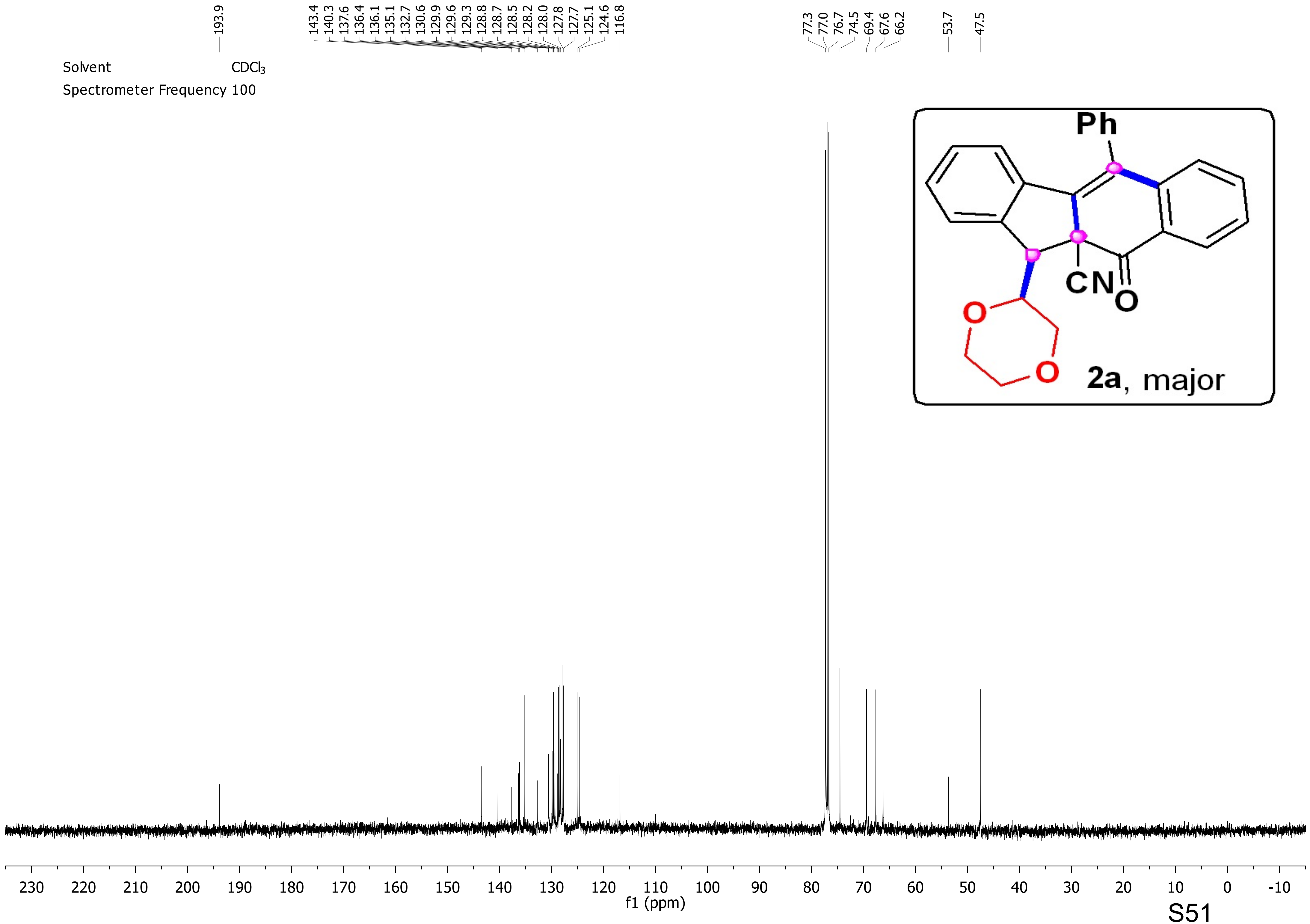
193.9
143.4
140.3
137.6
136.4
136.1
135.1
132.7
130.6
129.9
129.6
129.3
128.8
128.7
128.5
128.2
128.0
127.8
127.7
125.1
124.6
116.8
77.3
77.0
76.7
74.5
69.4
67.6
66.2
53.7
47.5
Solvent CDCl3
Spectrometer Frequency 100
Ph
CN
O
O
O
2a, major
230 220 210 200 190 180 170 160 150 140 130 120 110 100 90 80 70 60 50 40 30 20 10 0 -10
f1 (ppm)

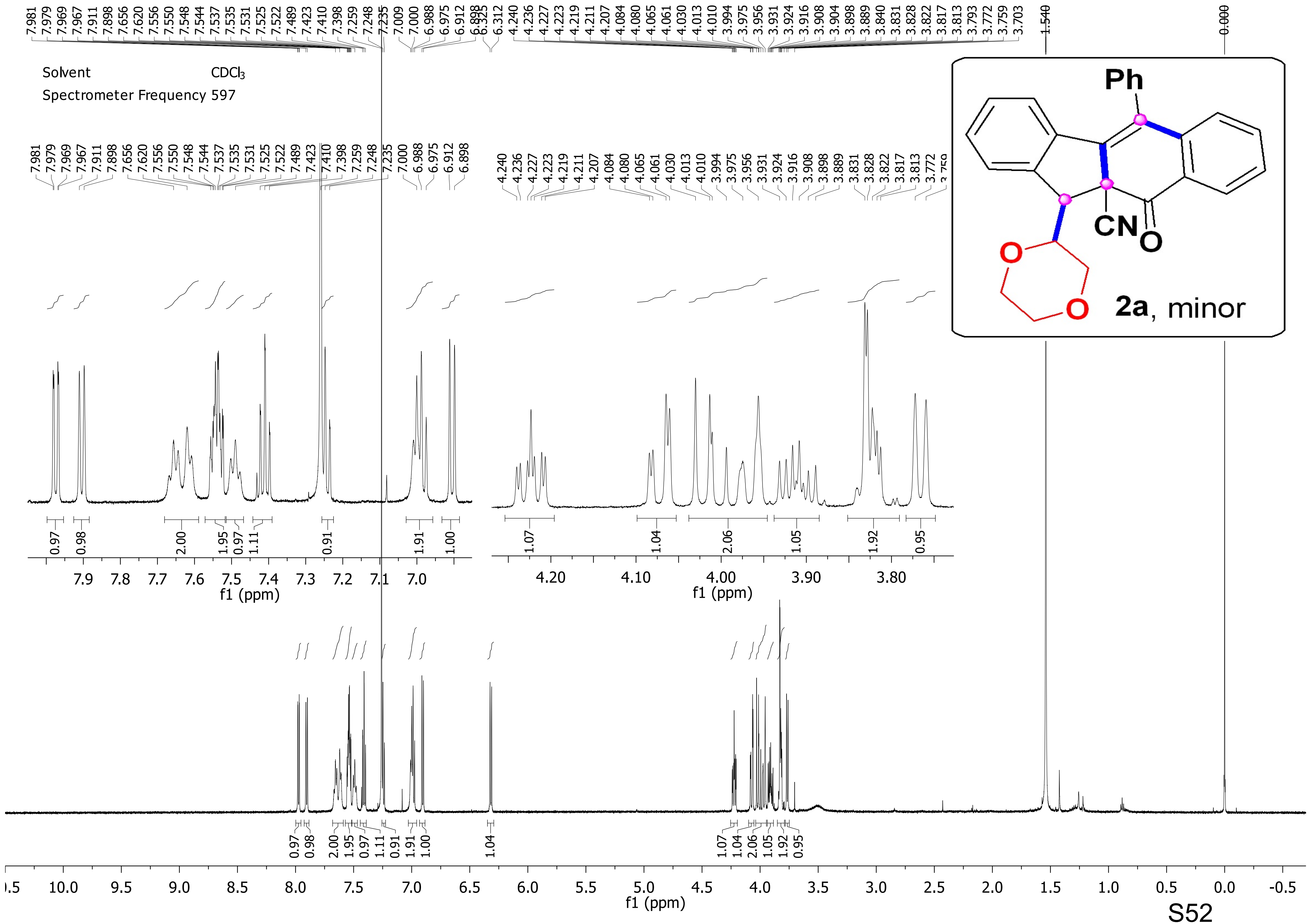
Solvent CDCl3
Spectrometer Frequency 597
Ph
CN
O
2a, minor
f1 (ppm)

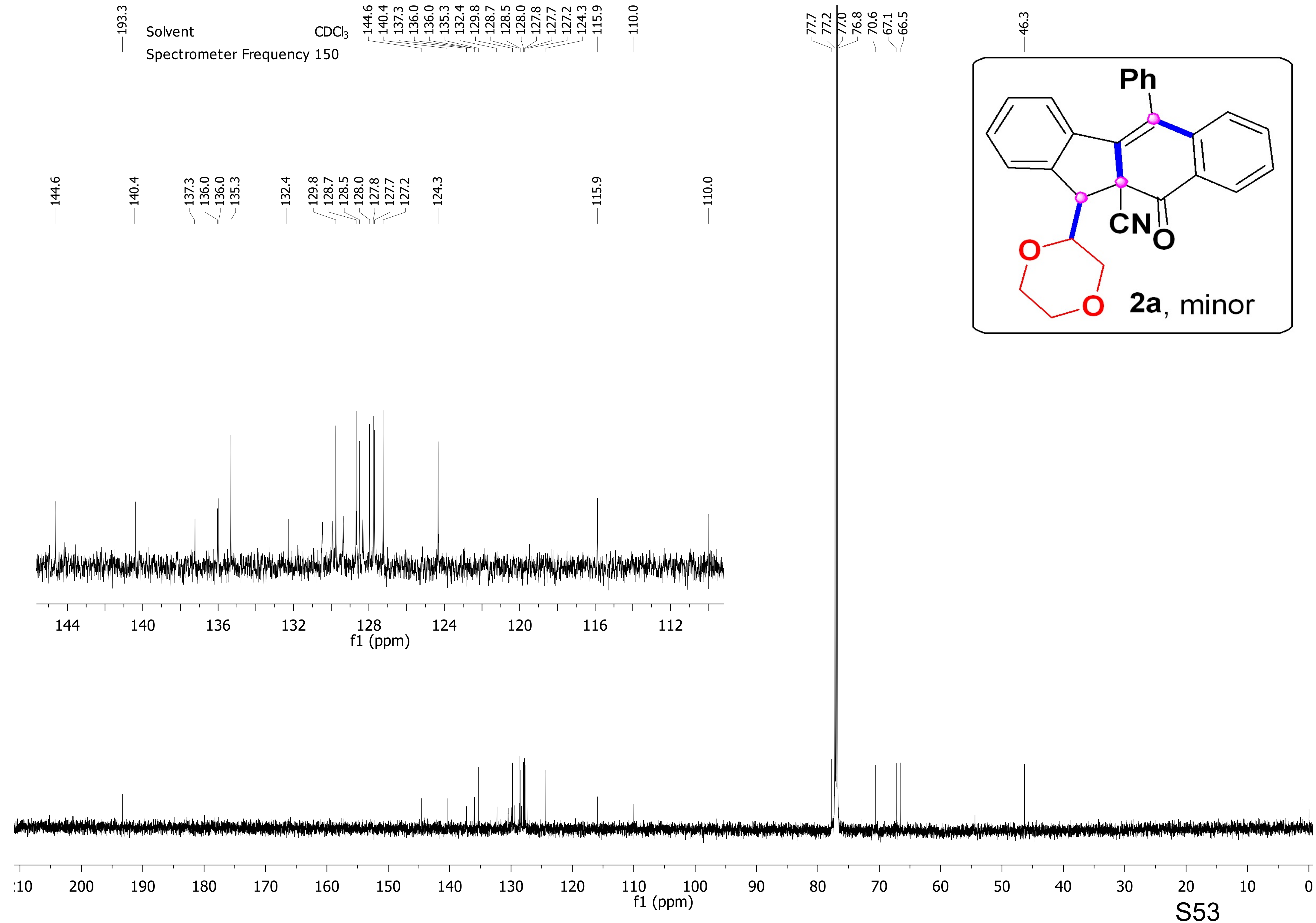
193.3
Solvent CDCl3
Spectrometer Frequency 150
144.6 140.4 137.3 136.0 136.0 135.3 132.4 129.8 128.7 128.5 128.0 127.8 127.7 127.2 124.3 115.9 110.0
77.7 77.2 77.0 76.8 70.6 67.1 66.5
46.3
Ph
CN
O
O
O
2a, minor
f1 (ppm)
f1 (ppm)

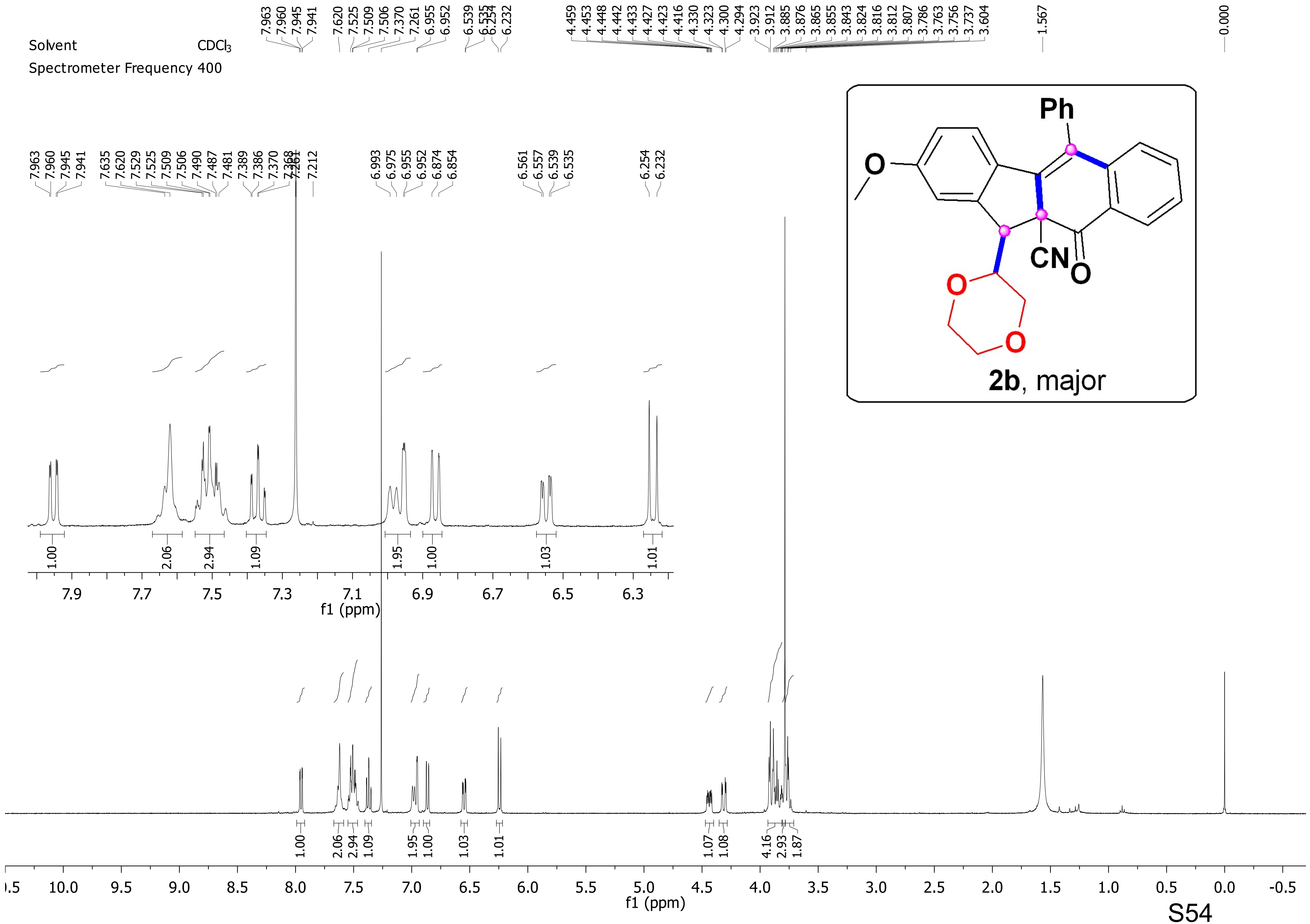
Solvent CDCl3
Spectrometer Frequency 400
7.963 7.960 7.945 7.941 7.620 7.525 7.509 7.506 7.370 7.261 6.955 6.952 6.539 6.535 6.254 6.232
4.459 4.453 4.448 4.442 4.433 4.427 4.423 4.416 4.330 4.323 4.300 4.294 3.923 3.912 3.885 3.876 3.865 3.855 3.843 3.824 3.816 3.812 3.807 3.786 3.763 3.756 3.737 3.604
1.567
0.000
7.963 7.960 7.945 7.941 7.635 7.620 7.529 7.525 7.509 7.506 7.490 7.487 7.481 7.389 7.386 7.370 7.368 7.261 7.212
6.993 6.975 6.955 6.952 6.874 6.854 6.561 6.557 6.539 6.535 6.254 6.232
1.00 2.06 2.94 1.09 1.95 1.00 1.03 1.01
f1 (ppm)
1.00 2.06 2.94 1.09 1.95 1.00 1.03 1.01 1.07 1.08 4.16 2.93 1.87
f1 (ppm)
Ph
O
CN
O
O
O
2b, major

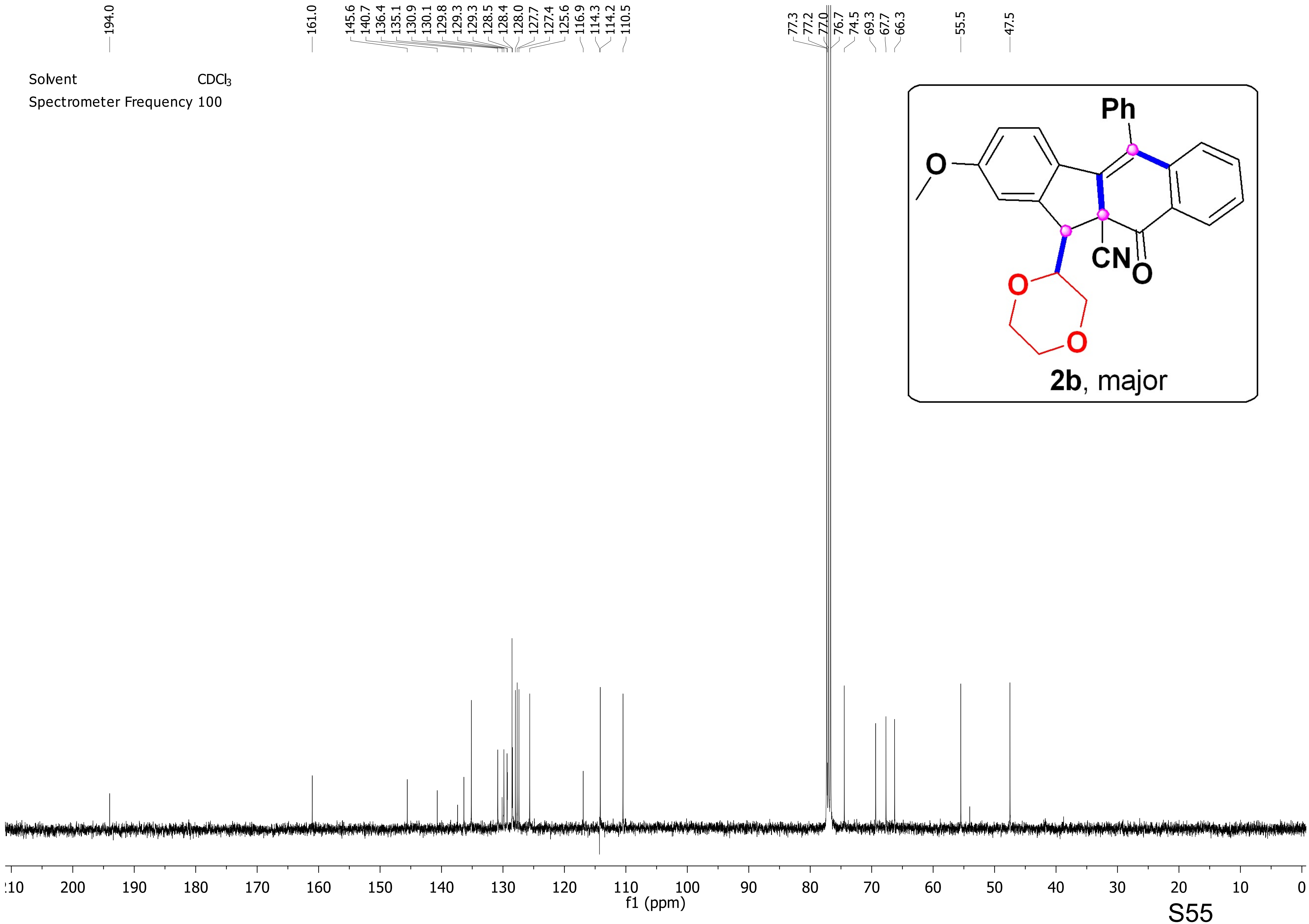
194.0
161.0
145.6
140.7
136.4
135.1
130.9
130.1
129.8
129.3
129.3
128.5
128.4
128.0
127.7
127.4
125.6
116.9
114.3
114.2
110.5
77.3
77.2
77.0
76.7
74.5
69.3
67.7
66.3
55.5
47.5
Solvent CDCl3
Spectrometer Frequency 100
Ph
O
CN
O
O
O
2b, major
210 200 190 180 170 160 150 140 130 120 110 100 90 80 70 60 50 40 30 20 10 0
f1 (ppm)

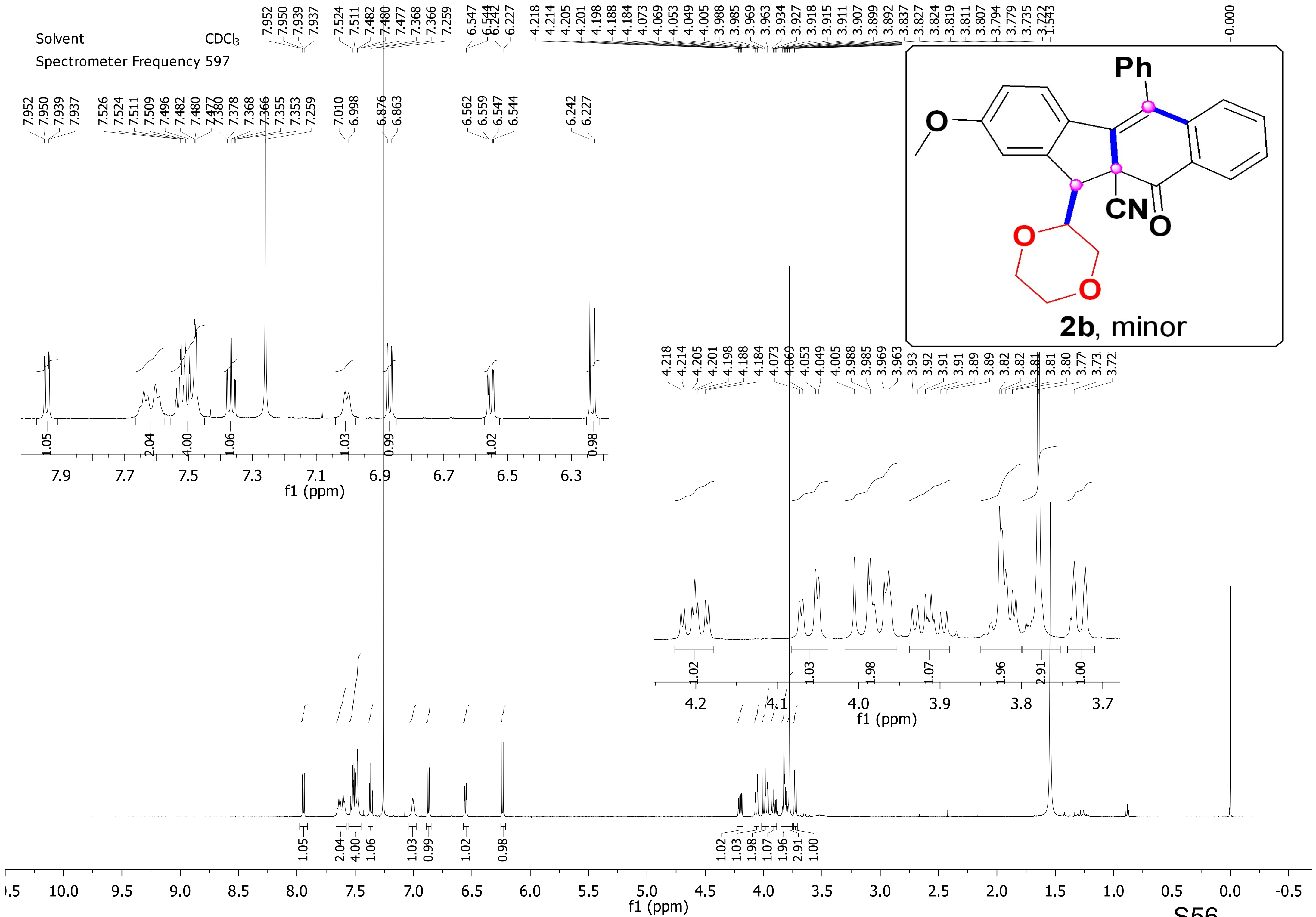
Solvent CDCl3
Spectrometer Frequency 597
Ph
O
CN
O
O
O
2b, minor
f1 (ppm)
f1 (ppm)
f1 (ppm)

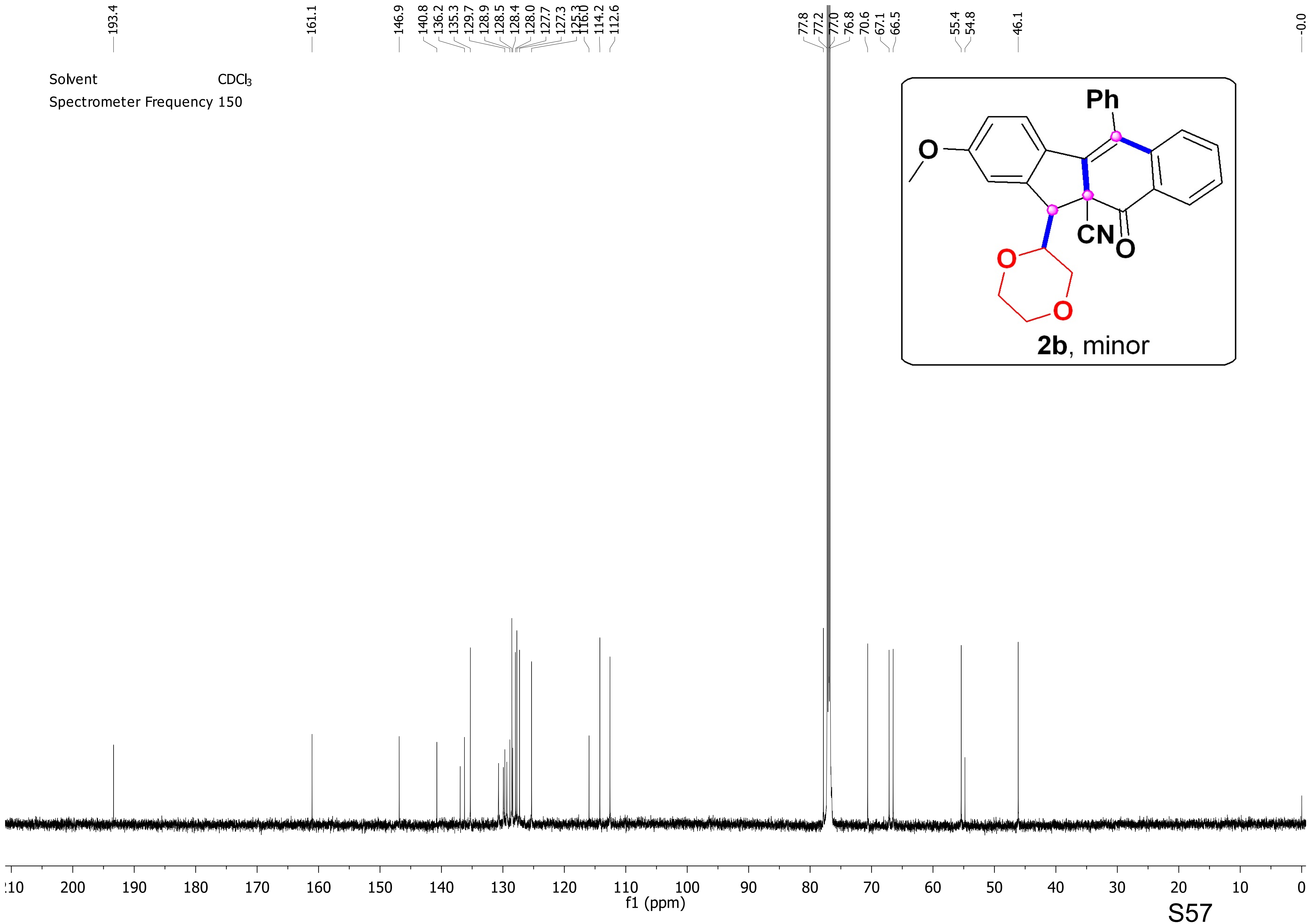
193.4
161.1
146.9
140.8
136.2
135.3
129.7
128.9
128.5
128.4
128.0
127.7
127.3
125.3
116.0
114.2
112.6
77.8
77.2
77.0
76.8
70.6
67.1
66.5
55.4
54.8
46.1
-0.0
Solvent CDCl3
Spectrometer Frequency 150
Ph
CN
2b, minor
f1 (ppm)

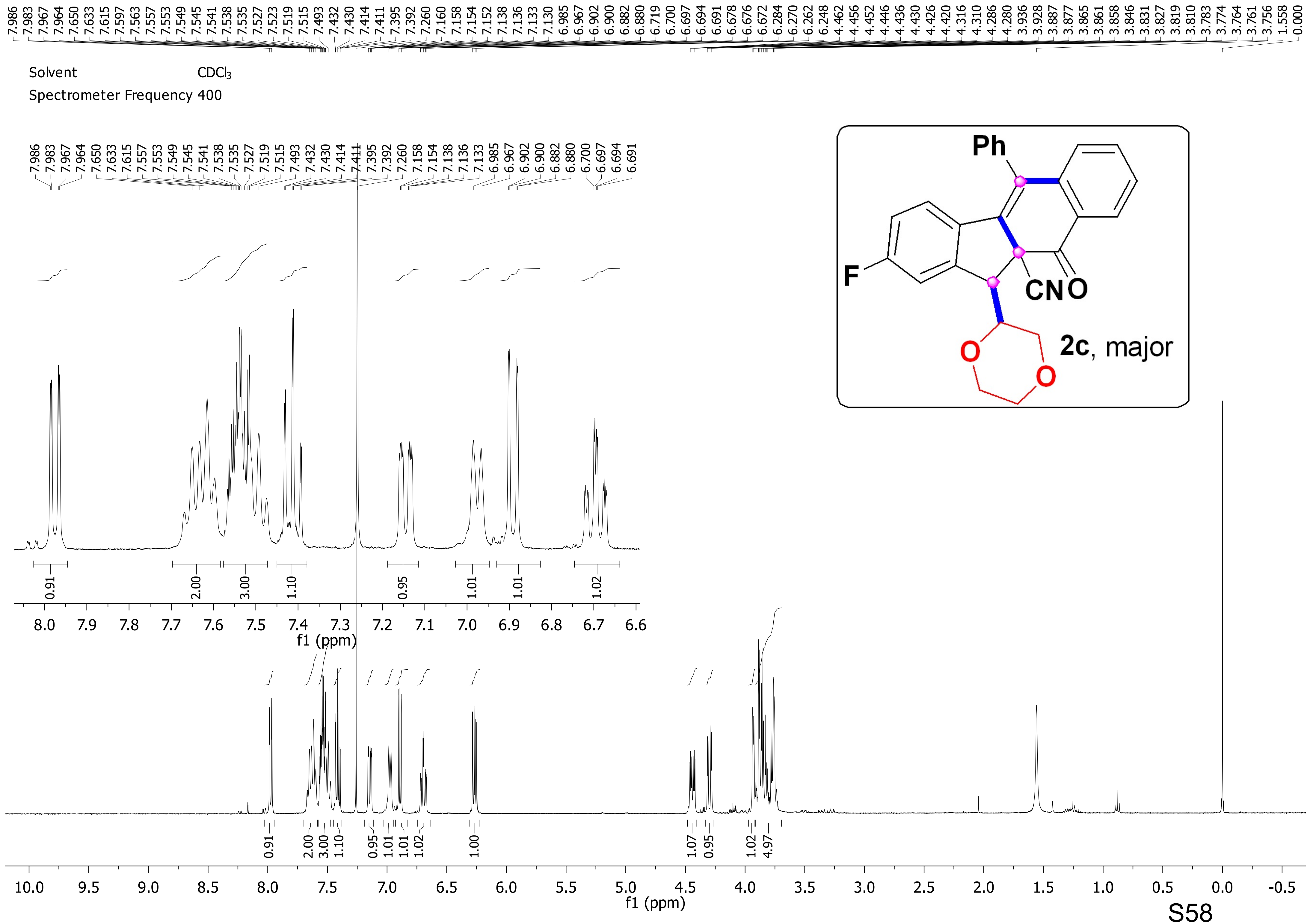
Solvent CDCl3
Spectrometer Frequency 400
Ph
F
CN
O
2c, major
f1 (ppm)

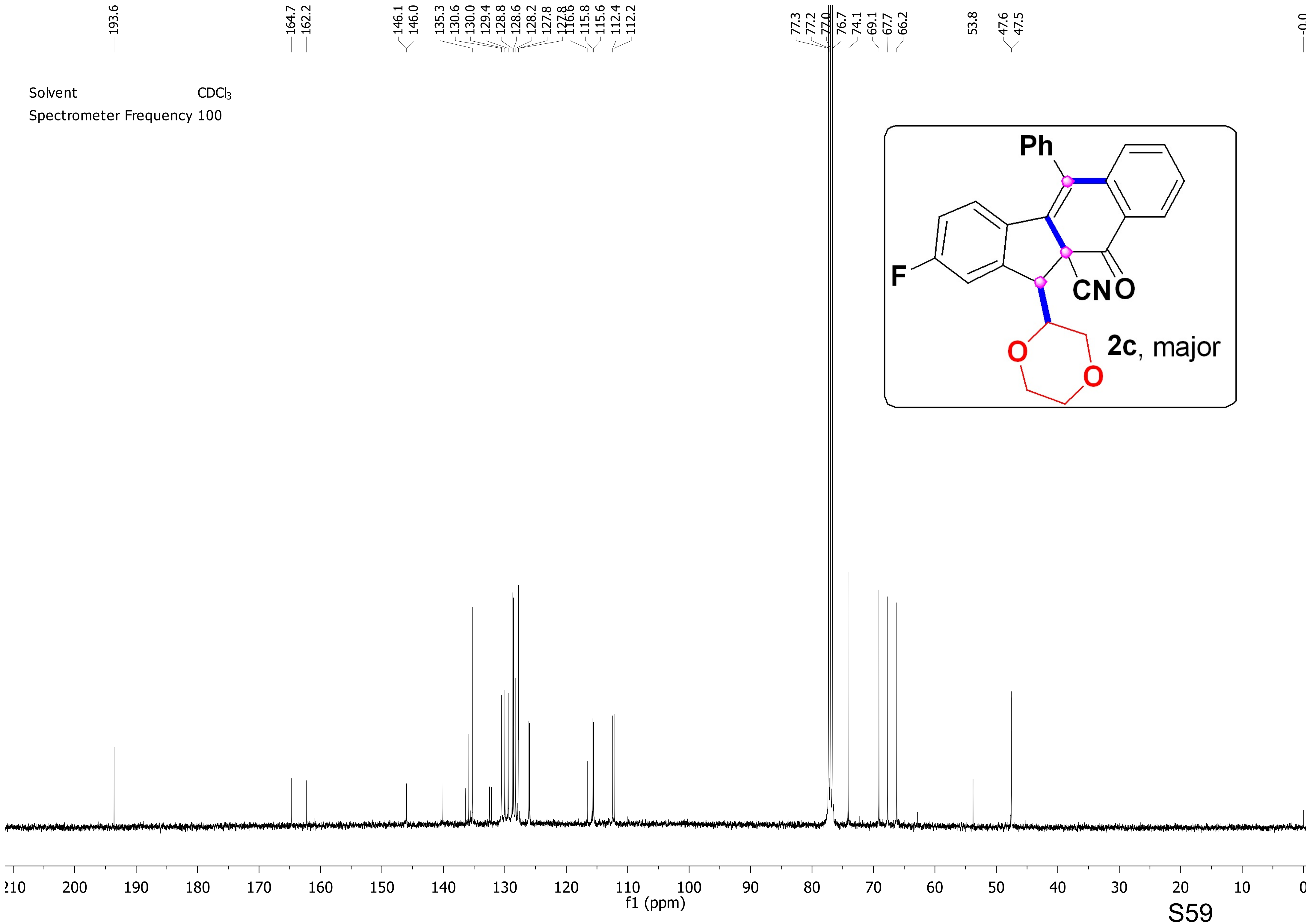

193.6
164.7
162.2
146.1
146.0
135.3
130.6
130.0
129.4
128.8
128.6
128.2
127.8
127.8
116.6
115.8
115.6
112.4
112.2
77.3
77.2
77.0
76.7
74.1
69.1
67.7
66.2
53.8
47.6
47.5
-0.0
Solvent CDCl3
Spectrometer Frequency 100
Ph
F
CN
O
O
O
2c, major
210 200 190 180 170 160 150 140 130 120 110 100 90 80 70 60 50 40 30 20 10 0
f1 (ppm)

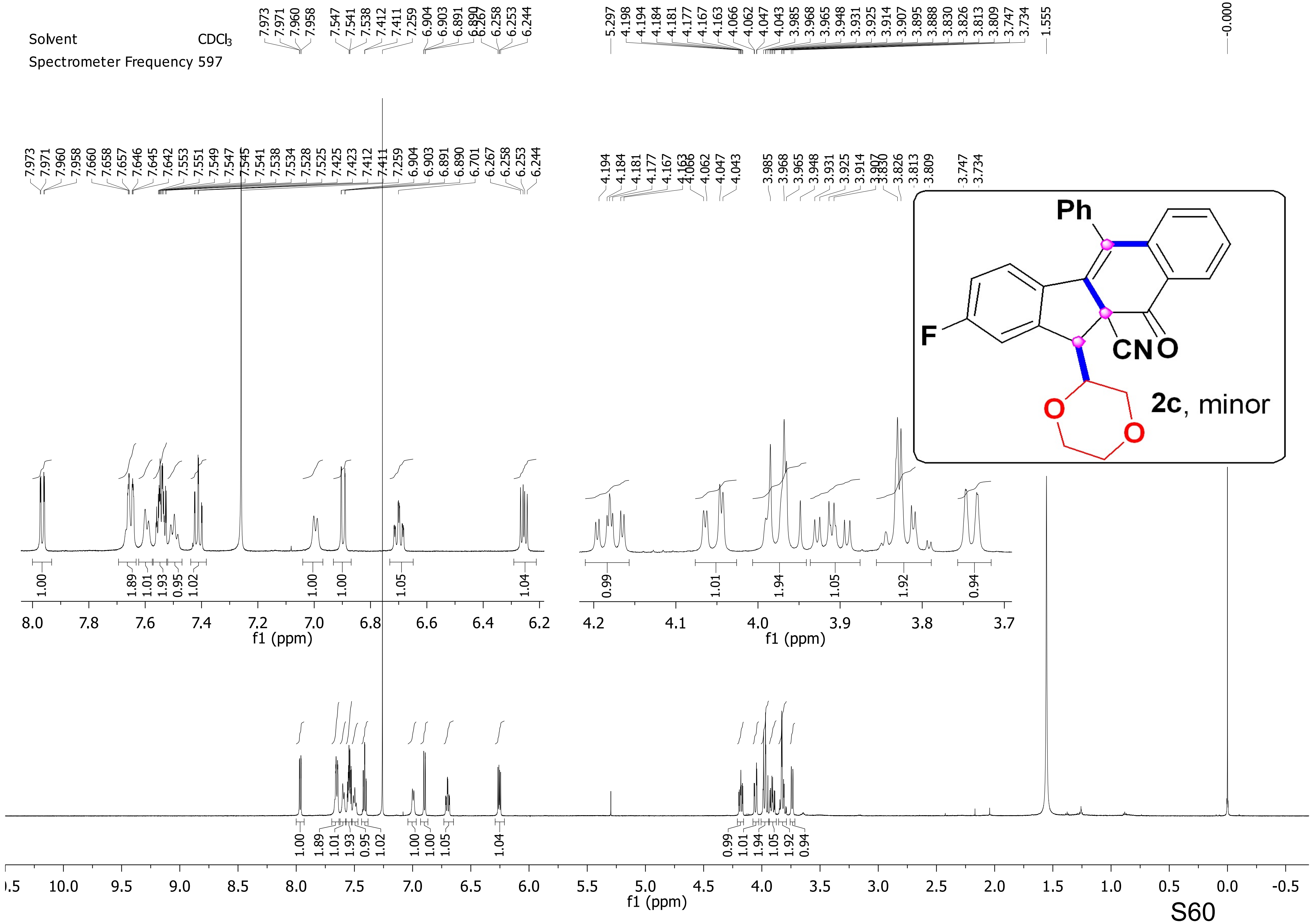
Solvent CDCl3
Spectrometer Frequency 597
Ph
F
CN
O
O
O
2c, minor
f1 (ppm)

Solvent CDCl3
Spectrometer Frequency 150

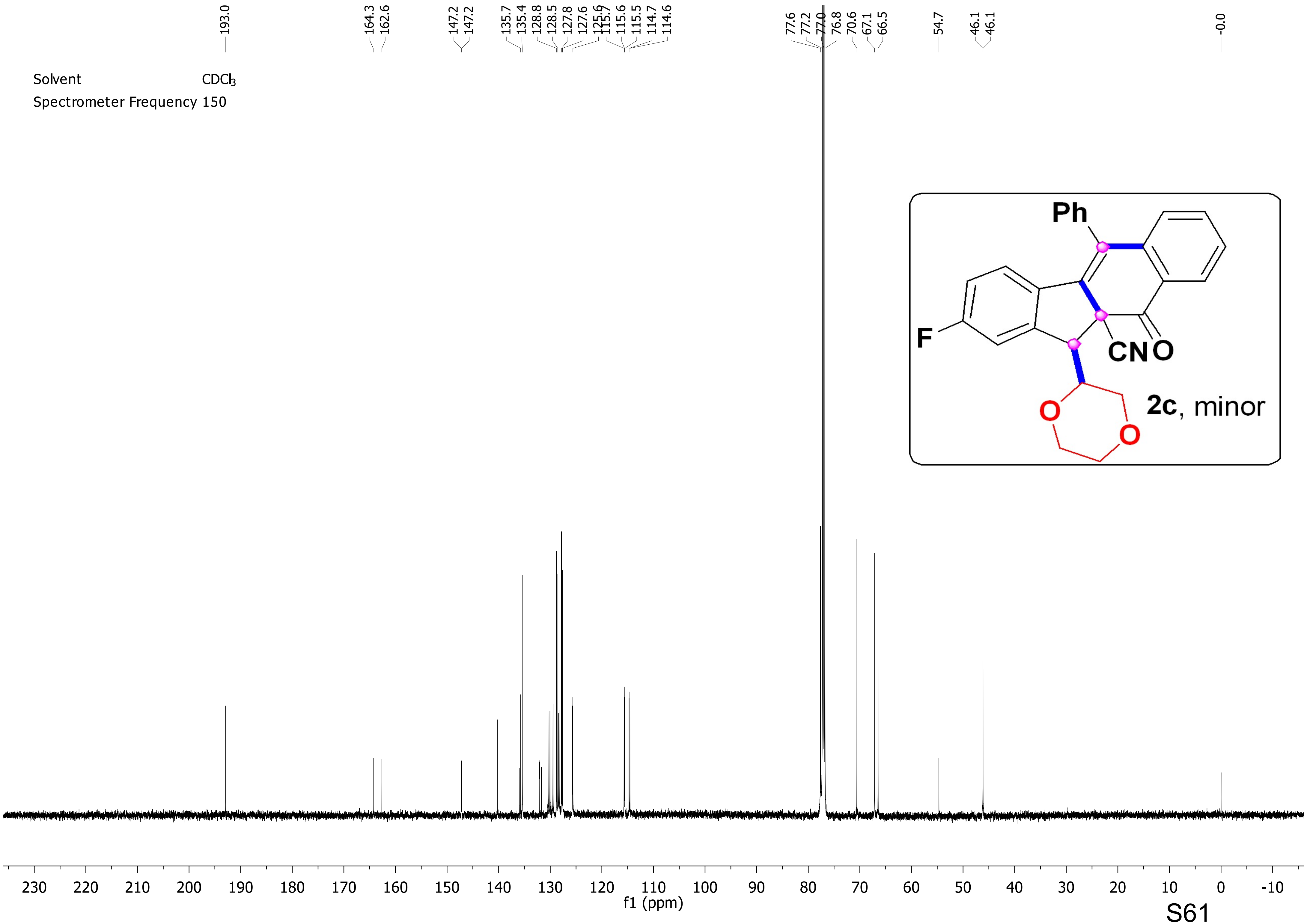

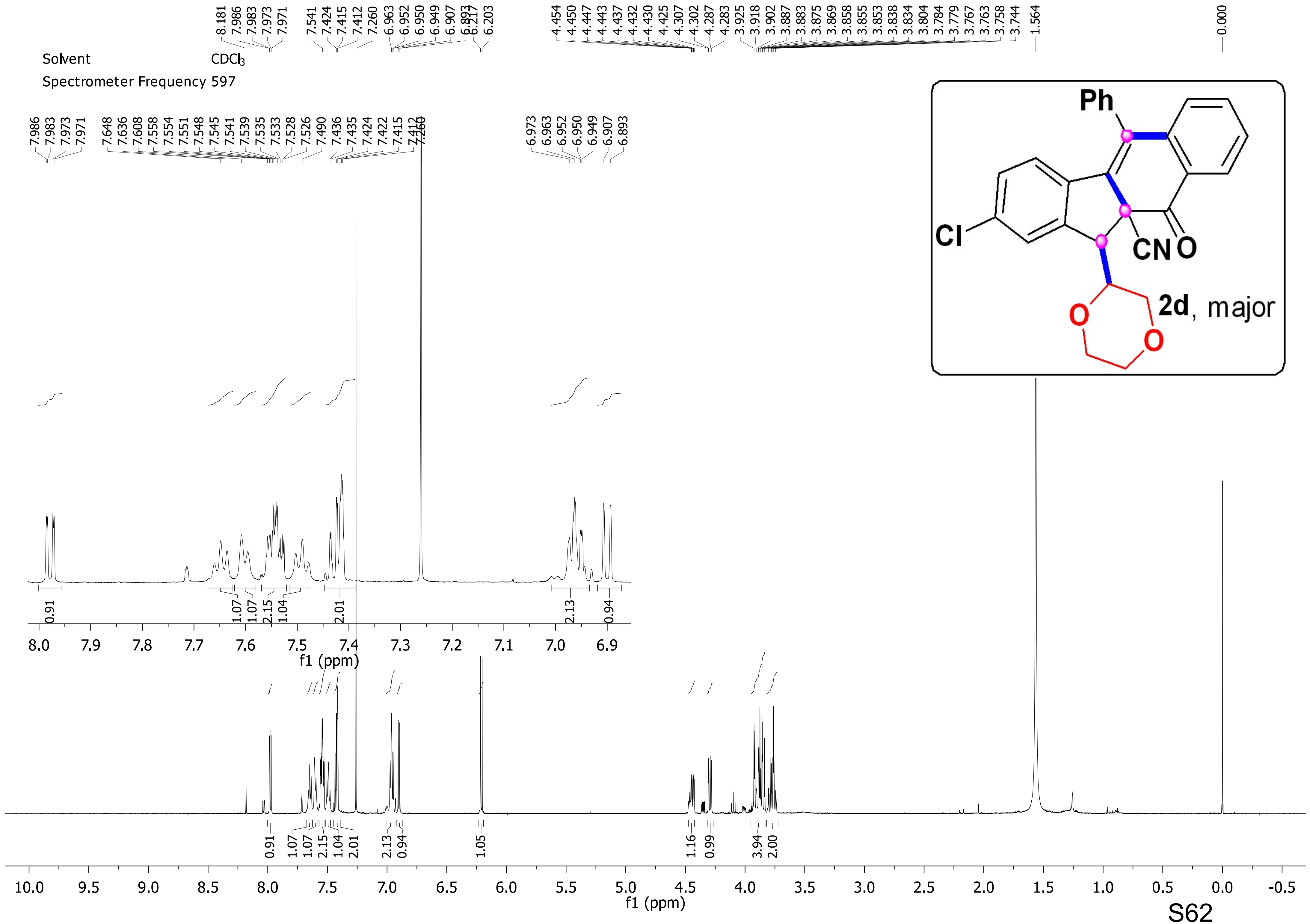
Solvent CDCl$_3$
Spectrometer Frequency 597
8.181 7.986 7.983 7.973 7.971 7.541 7.424 7.415 7.412 7.260 6.963 6.952 6.950 6.949 6.907 6.893 6.217 6.203 4.454 4.450 4.447 4.443 4.437 4.432 4.430 4.425 4.307 4.302 4.287 4.283 3.925 3.918 3.902 3.887 3.883 3.875 3.869 3.858 3.855 3.853 3.838 3.834 3.804 3.784 3.779 3.767 3.763 3.758 3.744 1.564 0.000
7.986 7.983 7.973 7.971 7.648 7.636 7.608 7.558 7.554 7.551 7.548 7.545 7.541 7.539 7.535 7.533 7.528 7.526 7.490 7.436 7.435 7.424 7.422 7.415 7.412 7.260
6.973 6.963 6.952 6.950 6.949 6.907 6.893
0.91 1.07 1.07 2.15 1.04 2.01 2.13 0.94
0.91 1.07 1.07 2.15 1.04 2.01 2.13 0.94 1.05 1.16 0.99 3.94 2.00
f1 (ppm)
Ph
Cl
CN
O
O
O
2d, major

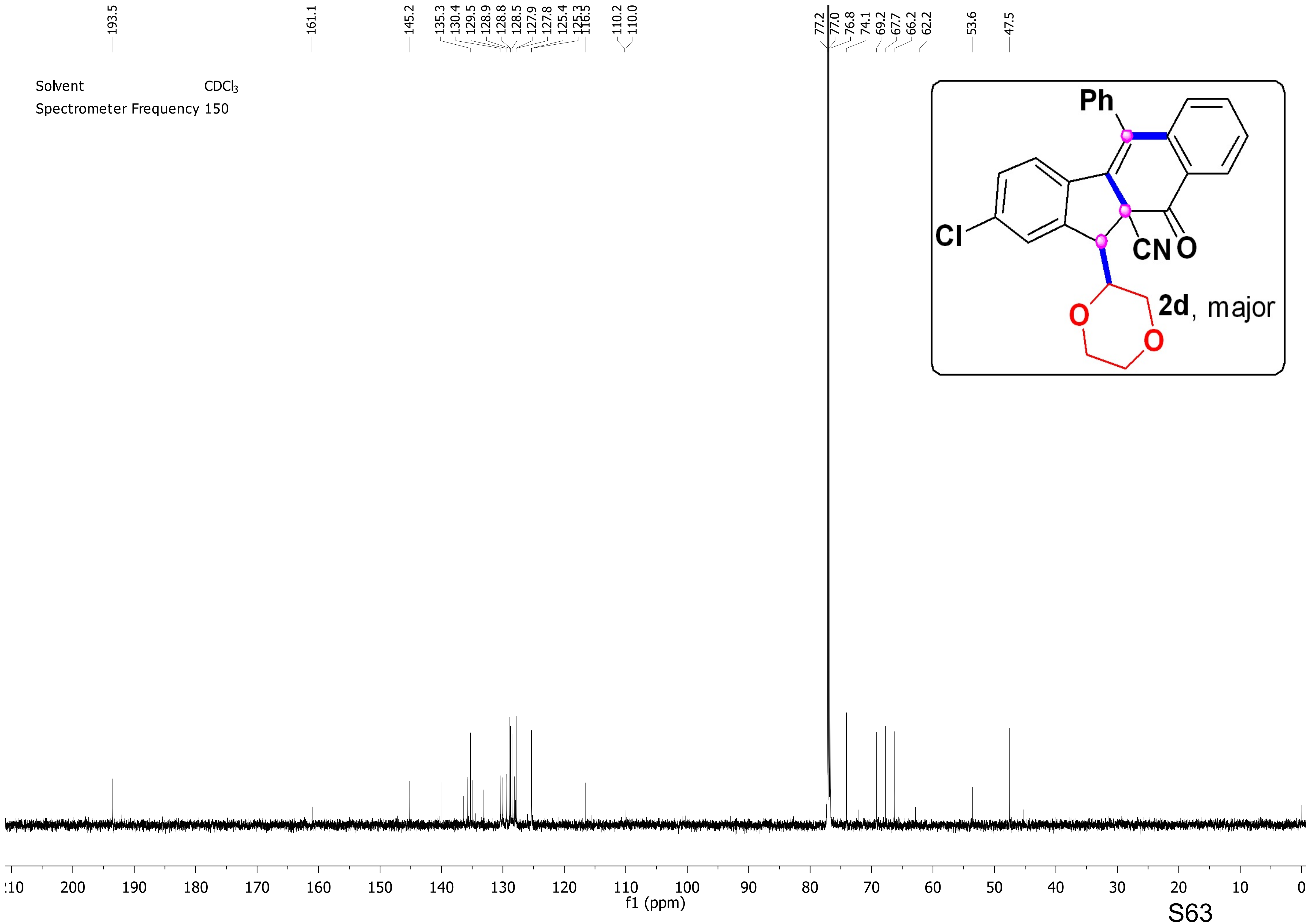
193.5
161.1
145.2
135.3
130.4
129.5
128.9
128.8
128.5
127.9
127.8
125.4
125.3
116.5
110.2
110.0
77.2
77.0
76.8
74.1
69.2
67.7
66.2
62.2
53.6
47.5
Solvent CDCl3
Spectrometer Frequency 150
Ph
Cl
CN
O
O
O
2d, major
f1 (ppm)

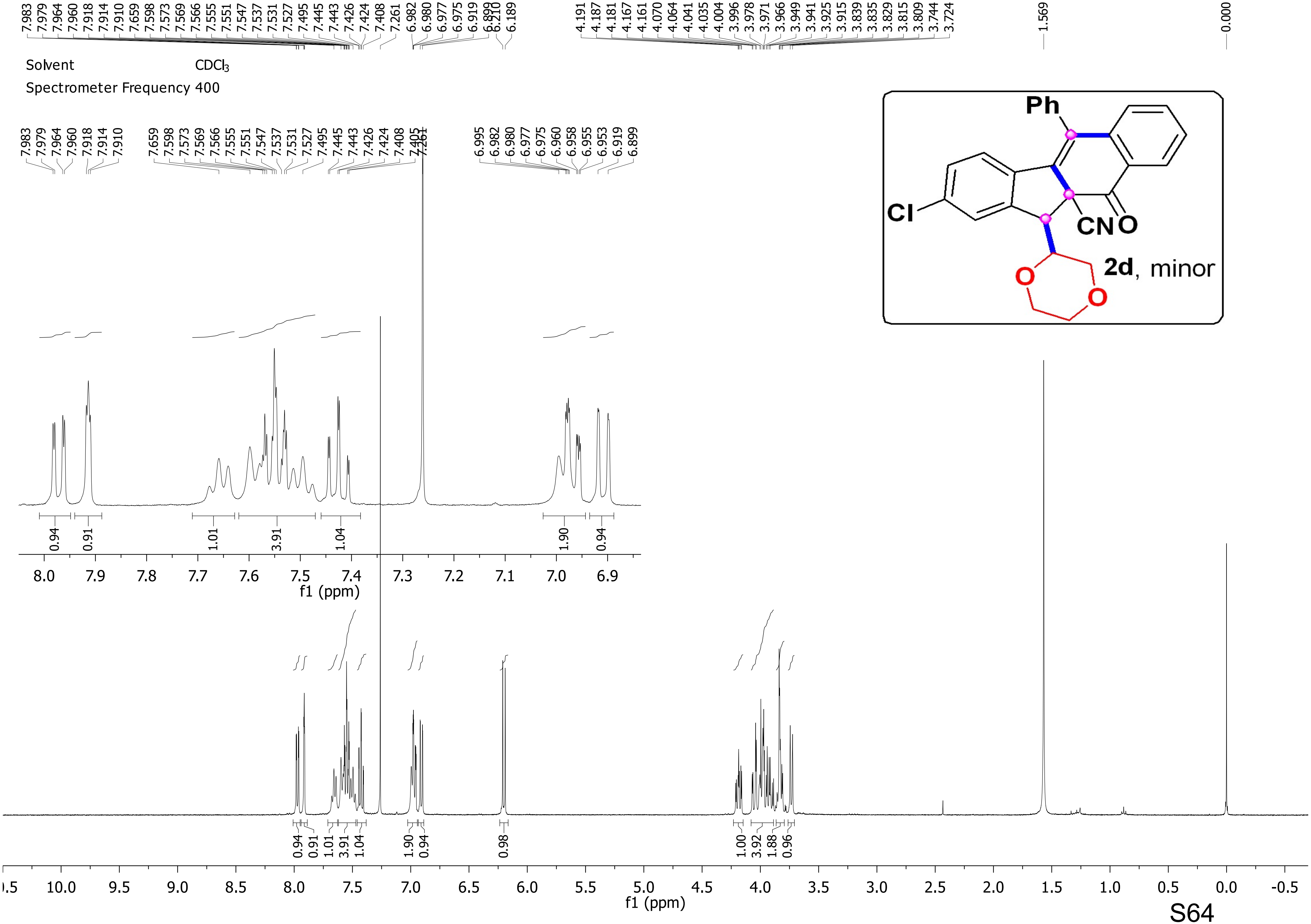

Solvent CDCl3
Spectrometer Frequency 400
Ph
Cl
CN
O
2d, minor
f1 (ppm)

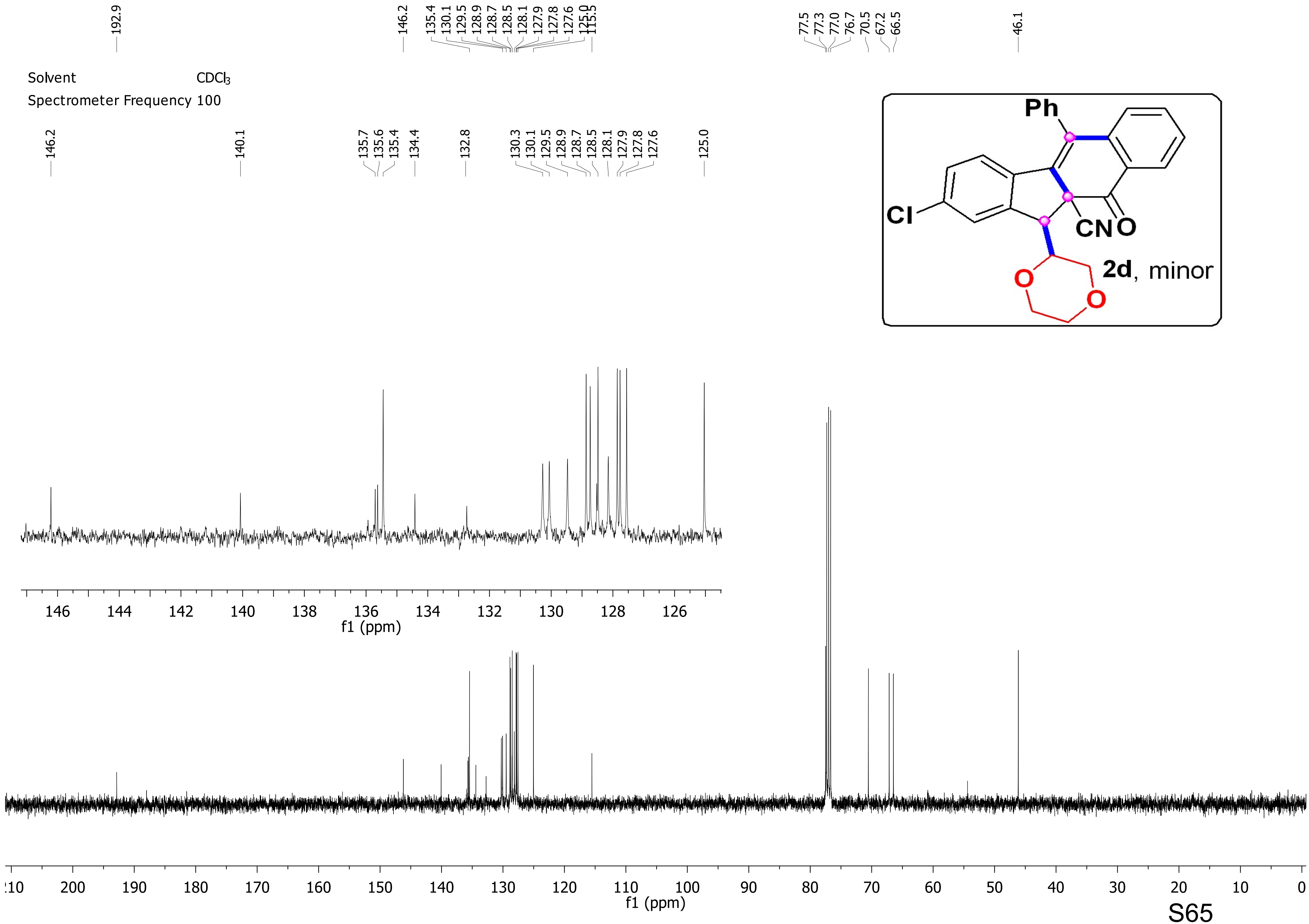
192.9
146.2
135.4
130.1
129.5
128.9
128.7
128.5
128.1
127.9
127.8
127.6
125.0
115.5
77.5
77.3
77.0
76.7
70.5
67.2
66.5
46.1
Solvent CDCl3
Spectrometer Frequency 100
146.2
140.1
135.7
135.6
135.4
134.4
132.8
130.3
130.1
129.5
128.9
128.7
128.5
128.1
127.9
127.8
127.6
125.0
f1 (ppm)
Ph
Cl
CN
O
2d, minor
f1 (ppm)

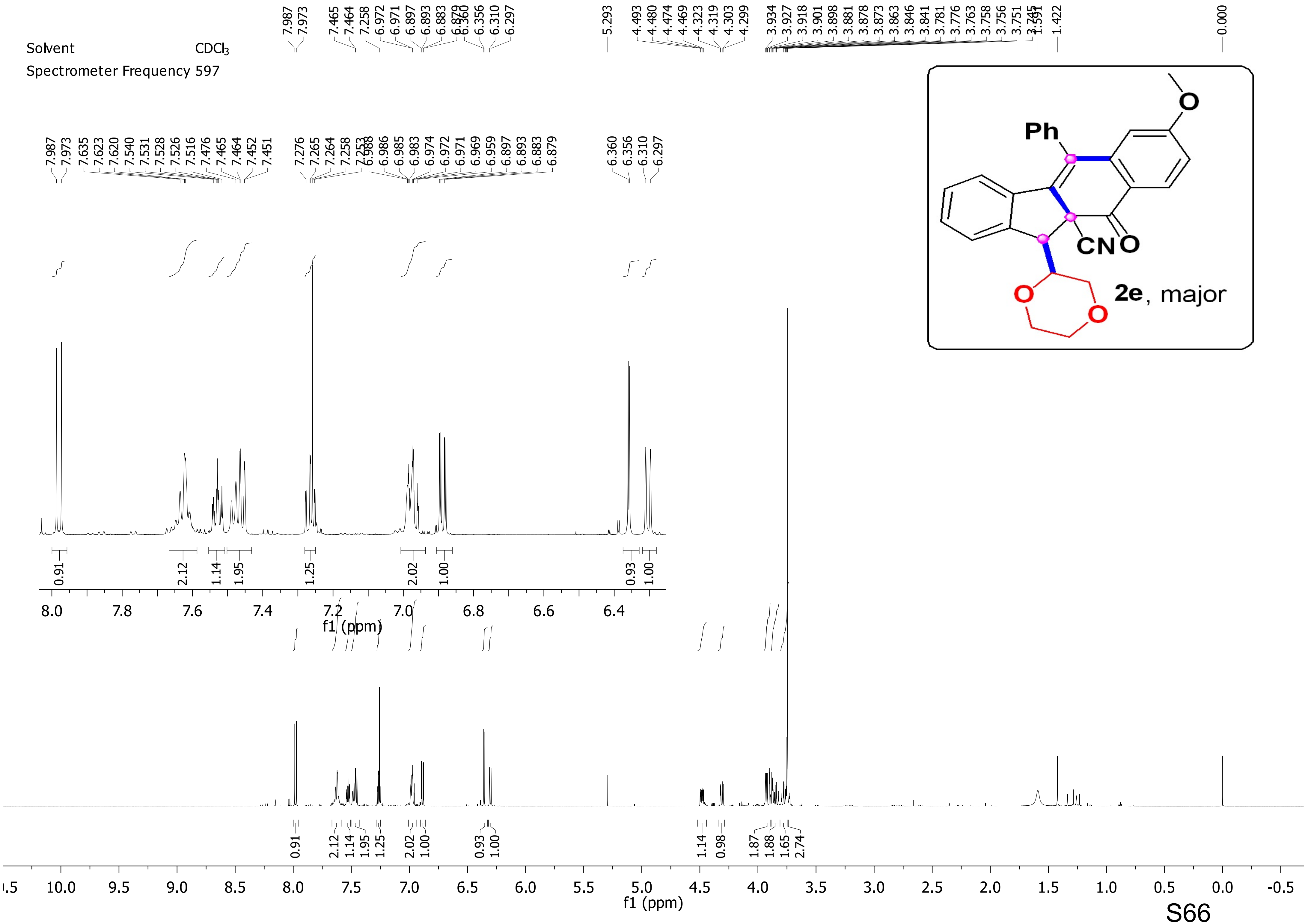

Solvent CDCl3
Spectrometer Frequency 597
7.987 7.973 7.465 7.464 7.258 6.972 6.971 6.897 6.893 6.883 6.879 6.360 6.356 6.310 6.297 5.293 4.493 4.480 4.474 4.469 4.323 4.319 4.303 4.299 3.934 3.927 3.918 3.901 3.898 3.881 3.878 3.873 3.863 3.846 3.841 3.781 3.776 3.763 3.758 3.756 3.751 3.745 1.591 1.422 0.000
7.987 7.973 7.635 7.623 7.620 7.540 7.531 7.528 7.526 7.516 7.476 7.465 7.464 7.452 7.451 7.276 7.265 7.264 7.258 7.253 6.988 6.986 6.985 6.983 6.974 6.972 6.971 6.969 6.959 6.897 6.893 6.883 6.879 6.360 6.356 6.310 6.297
0.91 2.12 1.14 1.95 1.25 2.02 1.00 0.93 1.00
0.91 2.12 1.14 1.95 1.25 2.02 1.00 0.93 1.00 1.14 0.98 1.87 1.88 1.65 2.74
f1 (ppm)
Ph
O
CN
O
O
O
2e, major

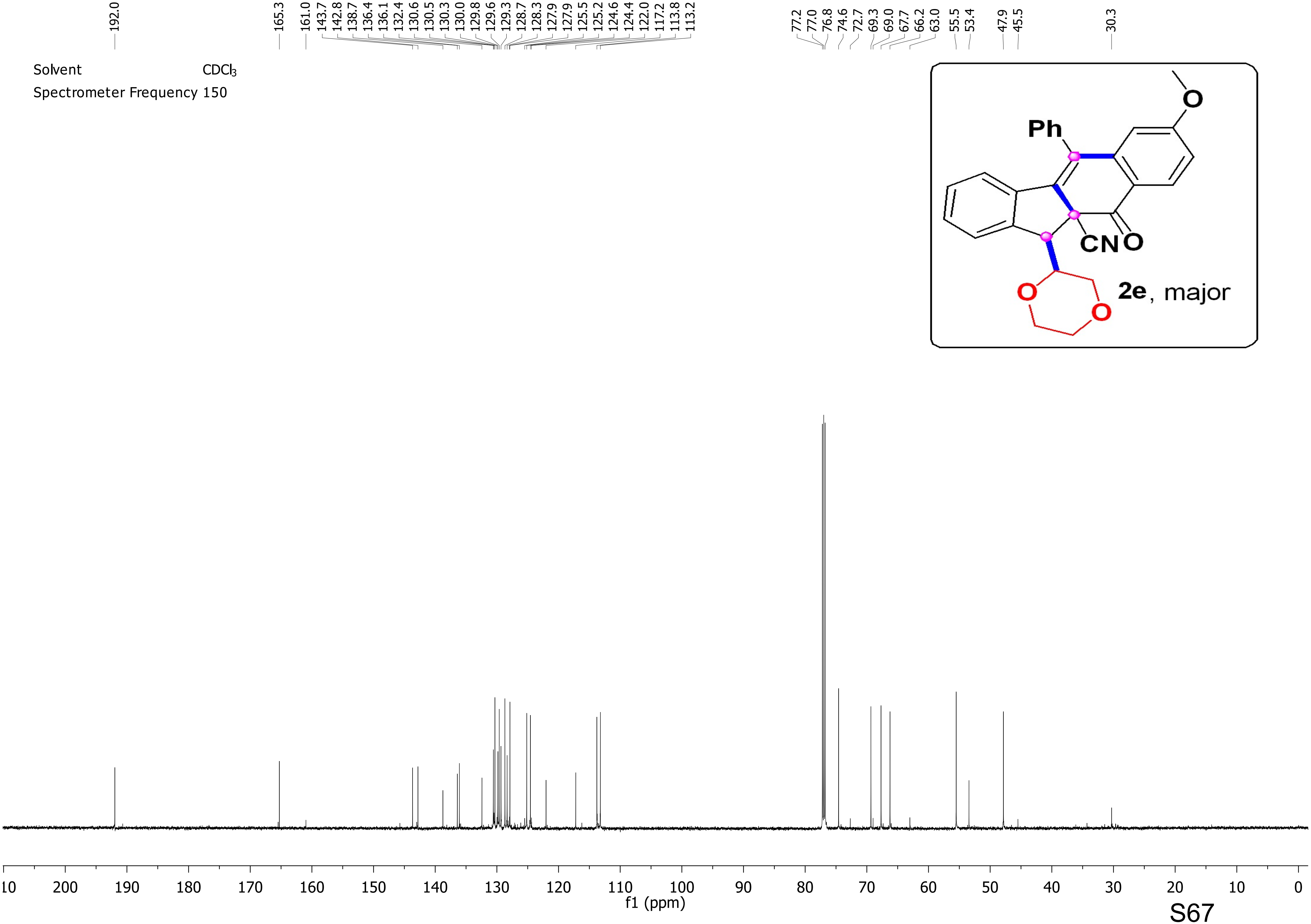
192.0
165.3
161.0
143.7
142.8
138.7
136.4
136.1
132.4
130.6
130.5
130.3
130.0
129.8
129.6
129.3
128.7
128.3
127.9
127.9
125.5
125.2
124.6
124.4
122.0
117.2
113.8
113.2
77.2
77.0
76.8
74.6
72.7
69.3
69.0
67.7
66.2
63.0
55.5
53.4
47.9
45.5
30.3
Solvent CDCl3
Spectrometer Frequency 150
Ph
O
CN
O
O
O
2e, major
f1 (ppm)

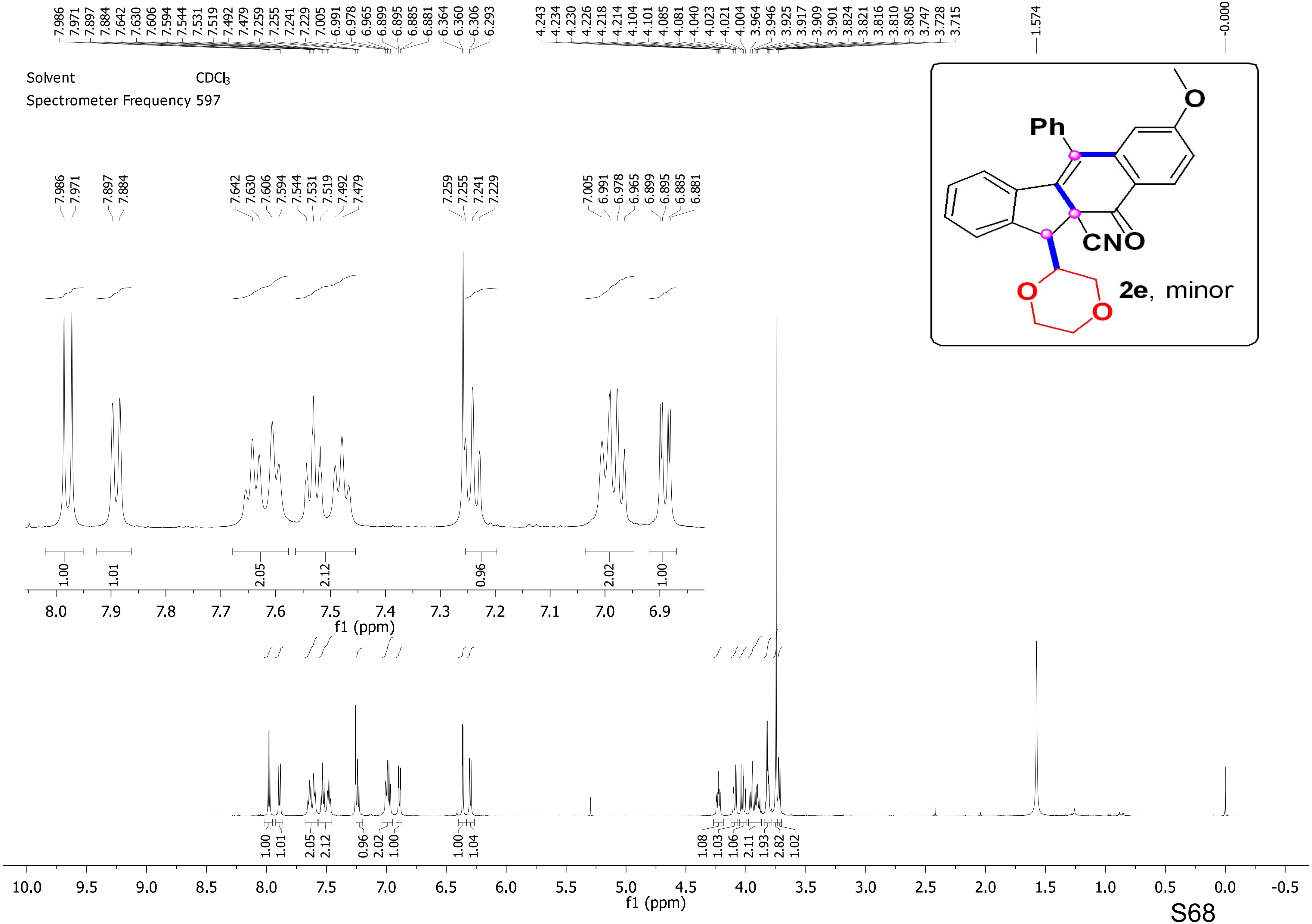
Solvent CDCl3
Spectrometer Frequency 597
Ph
O
CN
O
O
O
2e, minor
f1 (ppm)

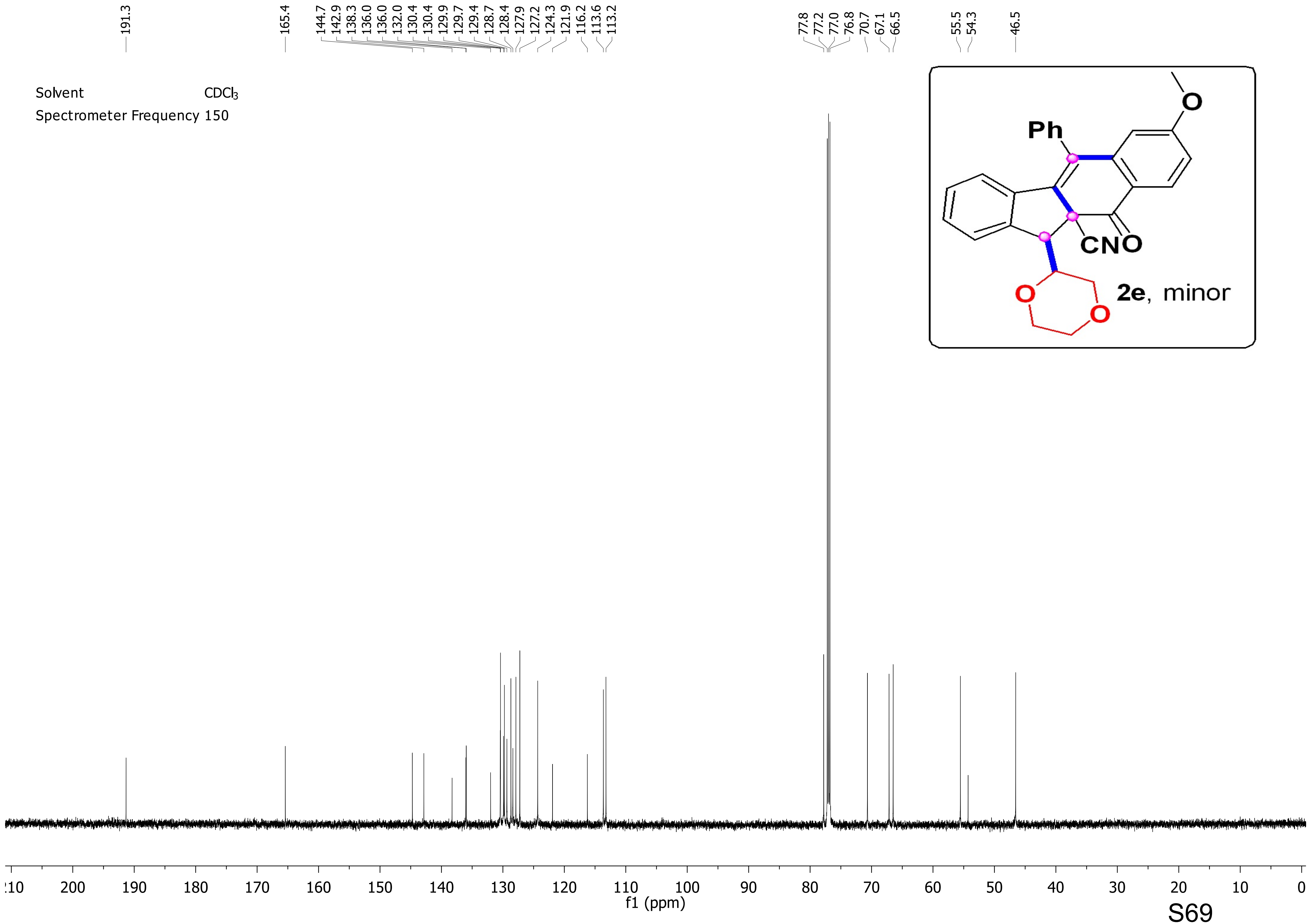

191.3
165.4
144.7
142.9
138.3
136.0
136.0
132.0
130.4
130.4
129.9
129.7
129.4
128.7
128.4
127.9
127.2
124.3
121.9
116.2
113.6
113.2
77.8
77.2
77.0
76.8
70.7
67.1
66.5
55.5
54.3
46.5
Solvent CDCl3
Spectrometer Frequency 150
Ph
O
CN
O
O
O
2e, minor
210
200
190
180
170
160
150
140
130
120
110
100
90
80
70
60
50
40
30
20
10
0
f1 (ppm)

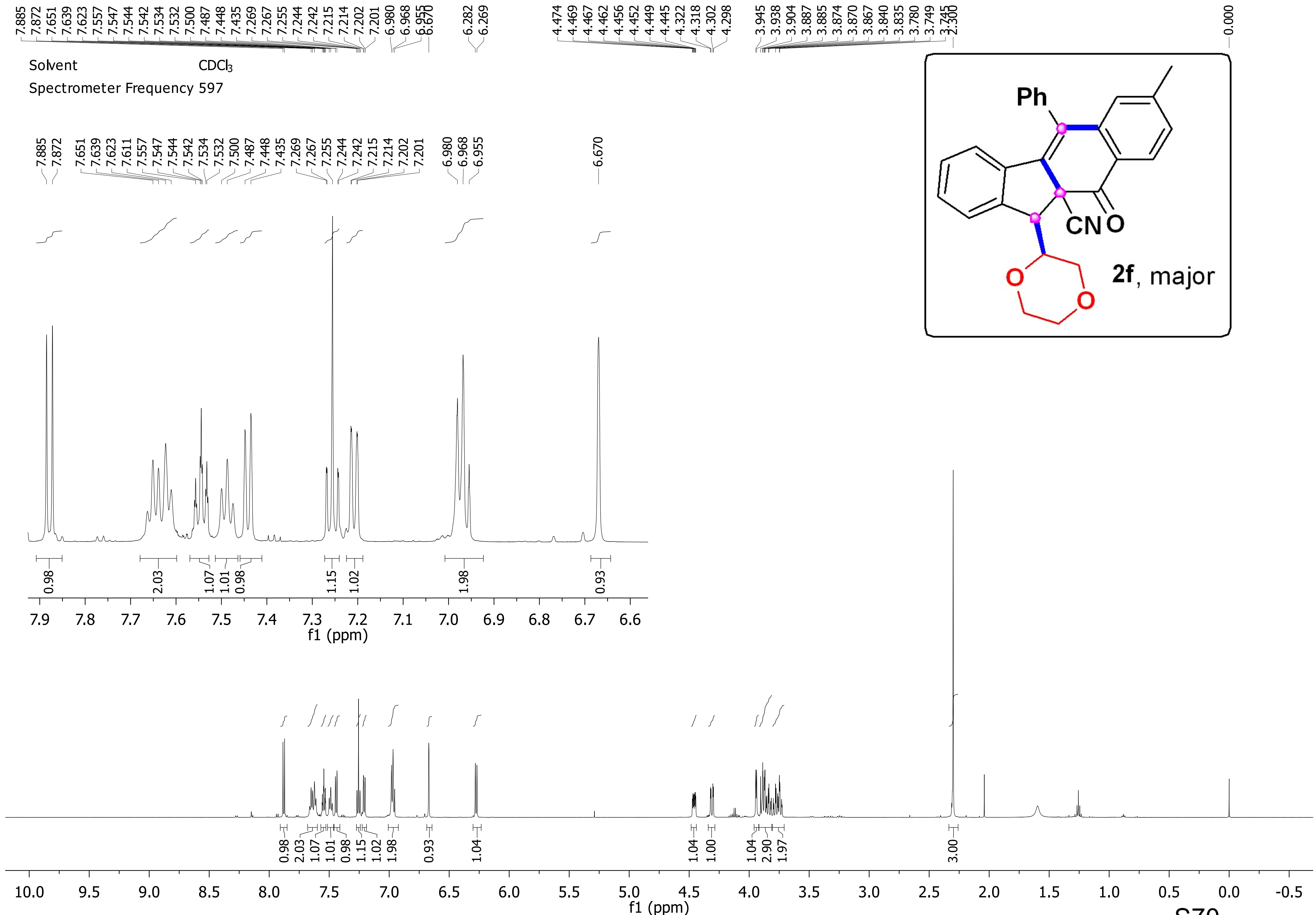
7.885 7.872 7.651 7.639 7.623 7.557 7.547 7.544 7.542 7.534 7.532 7.500 7.487 7.448 7.435 7.269 7.267 7.255 7.244 7.242 7.215 7.214 7.202 7.201 6.980 6.968 6.955 6.670 6.282 6.269 4.474 4.469 4.467 4.462 4.456 4.452 4.449 4.445 4.322 4.318 4.302 4.298 3.945 3.938 3.904 3.887 3.885 3.874 3.870 3.867 3.840 3.835 3.780 3.749 3.745 2.300 0.000
Solvent CDCl3
Spectrometer Frequency 597
Ph
CN
O
2f, major
f1 (ppm)

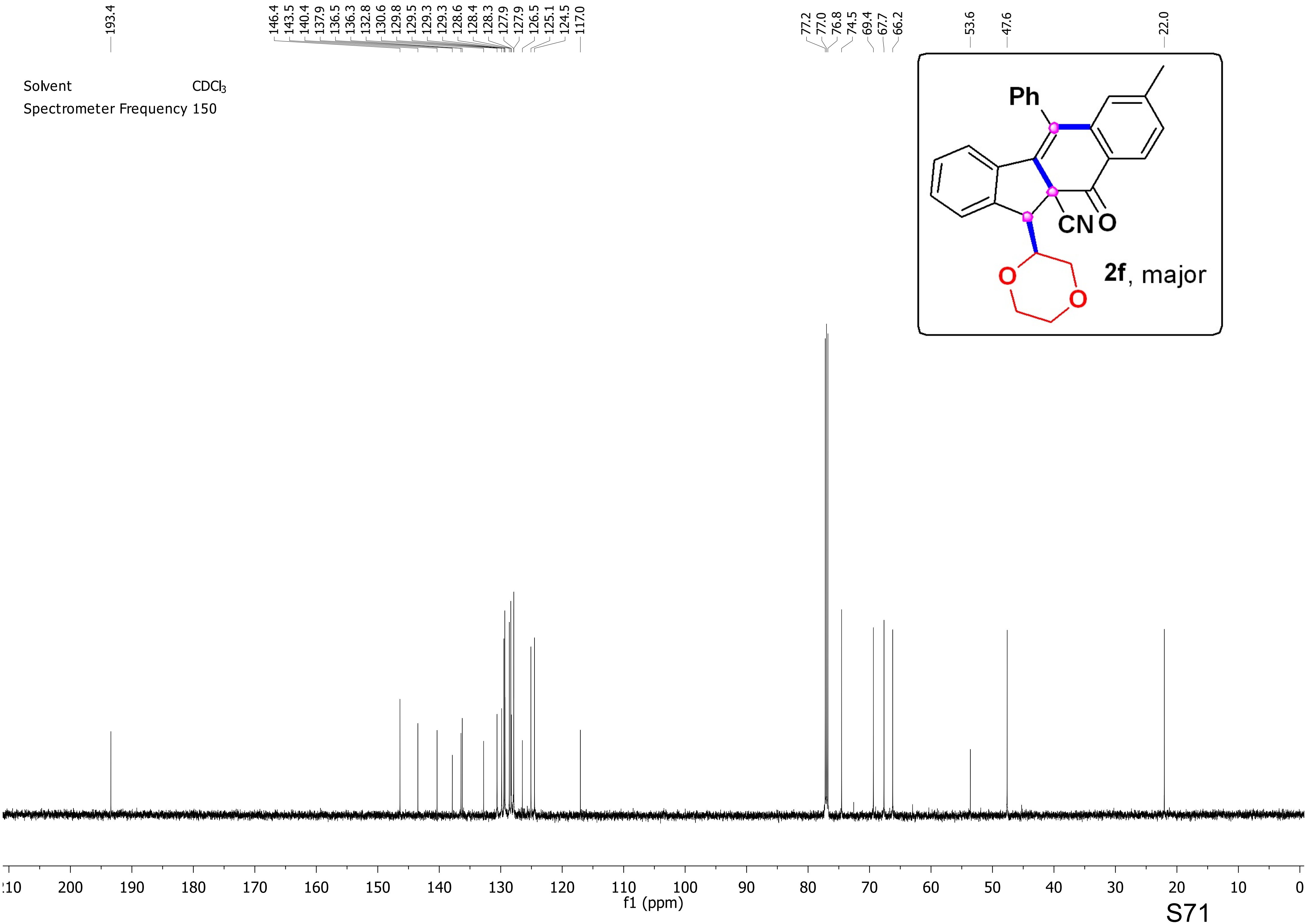
193.4
146.4
143.5
140.4
137.9
136.5
136.3
132.8
130.6
129.8
129.5
129.3
129.3
128.6
128.4
128.3
127.9
127.9
126.5
125.1
124.5
117.0
77.2
77.0
76.8
74.5
69.4
67.7
66.2
53.6
47.6
22.0
Solvent CDCl3
Spectrometer Frequency 150
Ph
CN
O
O
O
2f, major
f1 (ppm)
210 200 190 180 170 160 150 140 130 120 110 100 90 80 70 60 50 40 30 20 10 0

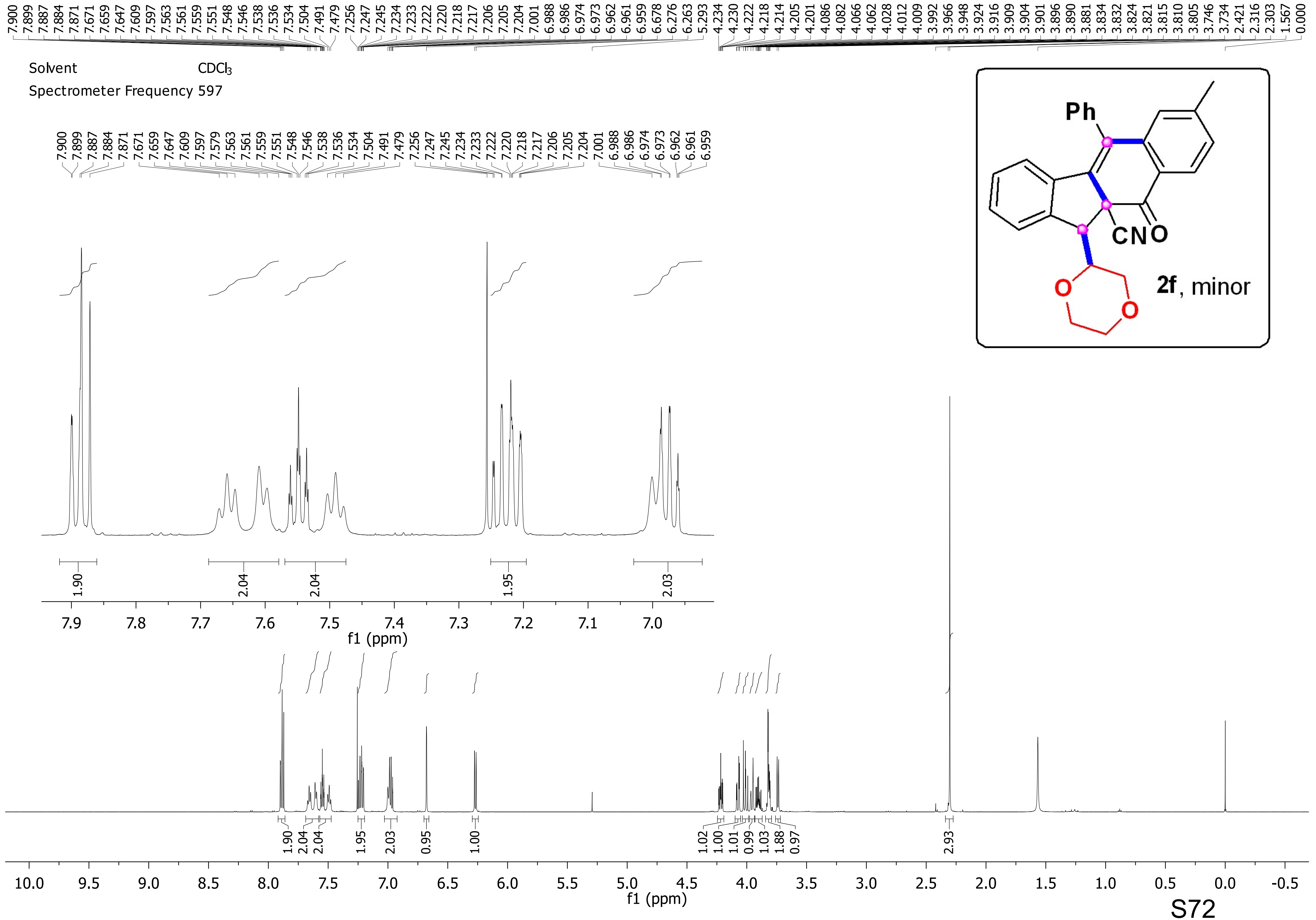
7.900 7.899 7.887 7.884 7.871 7.671 7.659 7.647 7.609 7.597 7.563 7.561 7.559 7.551 7.548 7.546 7.538 7.536 7.534 7.504 7.491 7.479 7.256 7.247 7.245 7.234 7.233 7.222 7.220 7.218 7.217 7.206 7.205 7.204 7.001 6.988 6.986 6.974 6.973 6.962 6.961 6.959 6.678 6.276 6.263 5.293 4.234 4.230 4.222 4.218 4.214 4.205 4.201 4.086 4.082 4.066 4.062 4.028 4.012 4.009 3.992 3.966 3.948 3.924 3.916 3.909 3.904 3.901 3.896 3.890 3.881 3.834 3.832 3.824 3.821 3.815 3.810 3.805 3.746 3.734 2.421 2.316 2.303 1.567 0.000
Solvent CDCl3
Spectrometer Frequency 597
Ph
CN
O
2f, minor
f1 (ppm)
f1 (ppm)

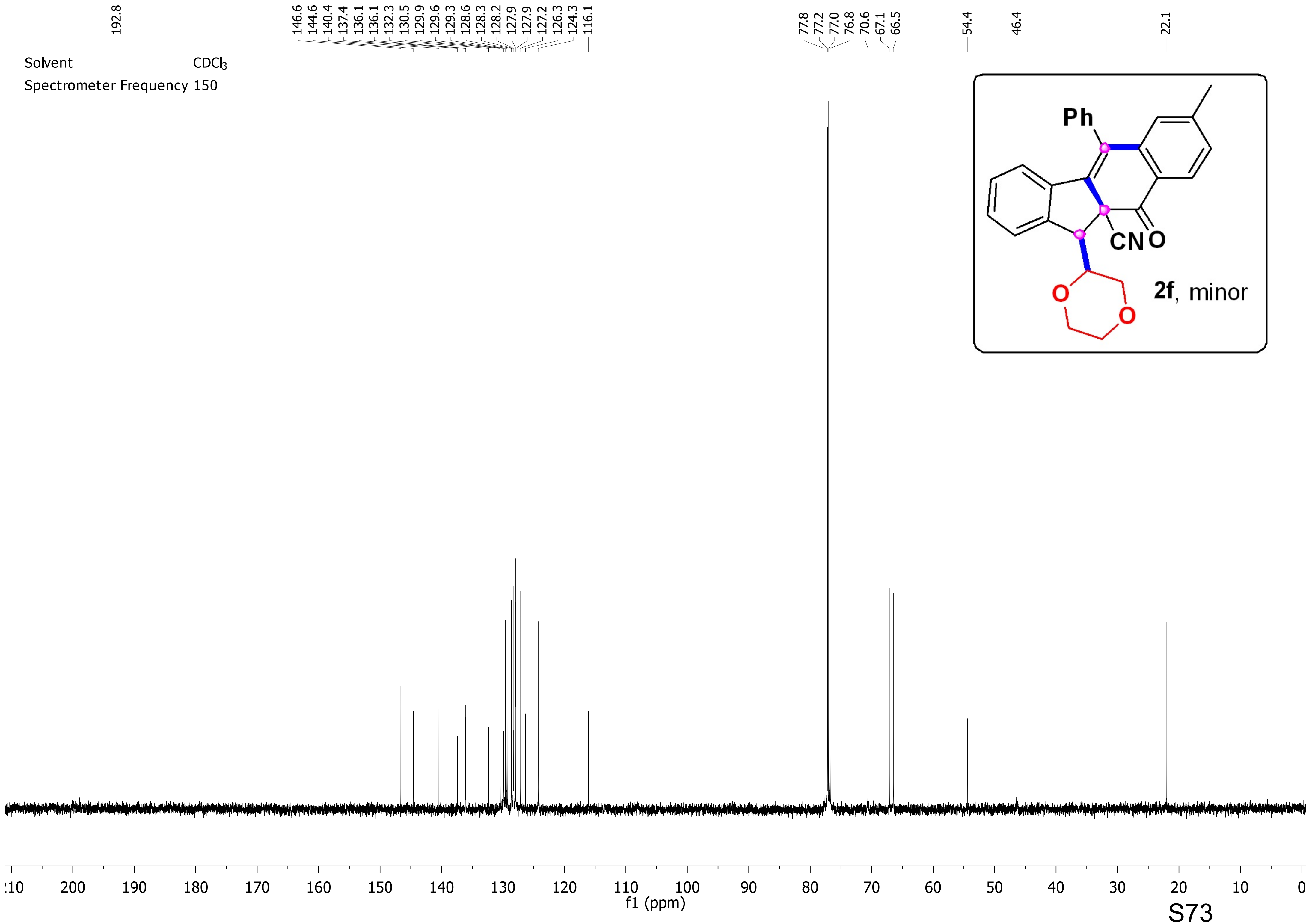
192.8
146.6
144.6
140.4
137.4
136.1
136.1
132.3
130.5
129.9
129.6
129.3
128.6
128.3
128.2
127.9
127.9
127.2
126.3
124.3
116.1
77.8
77.2
77.0
76.8
70.6
67.1
66.5
54.4
46.4
22.1
Solvent CDCl3
Spectrometer Frequency 150
Ph
CN
O
O
O
2f, minor
f1 (ppm)

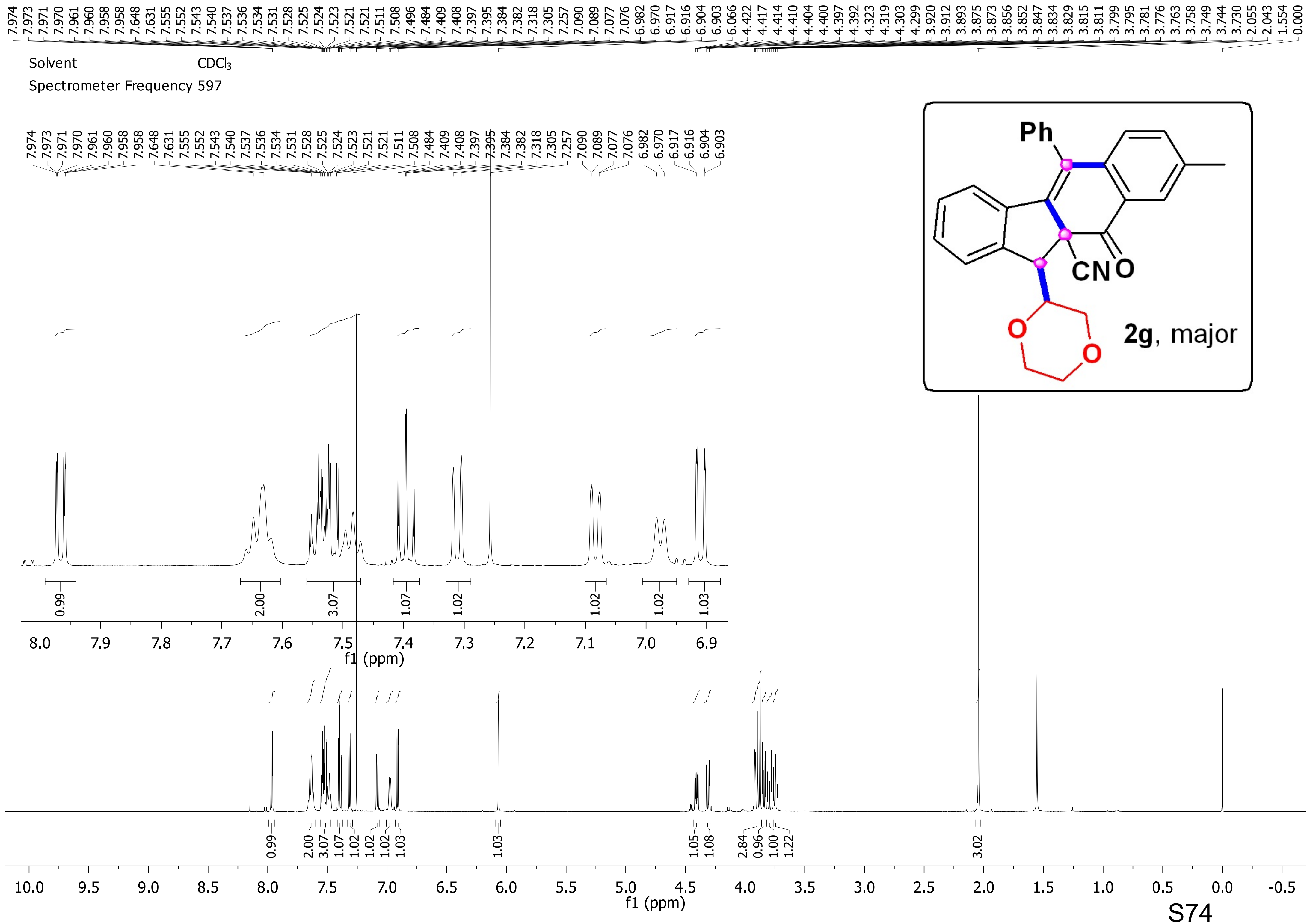
Solvent CDCl$_3$
Spectrometer Frequency 597
Ph
CN
O
2g, major
f1 (ppm)

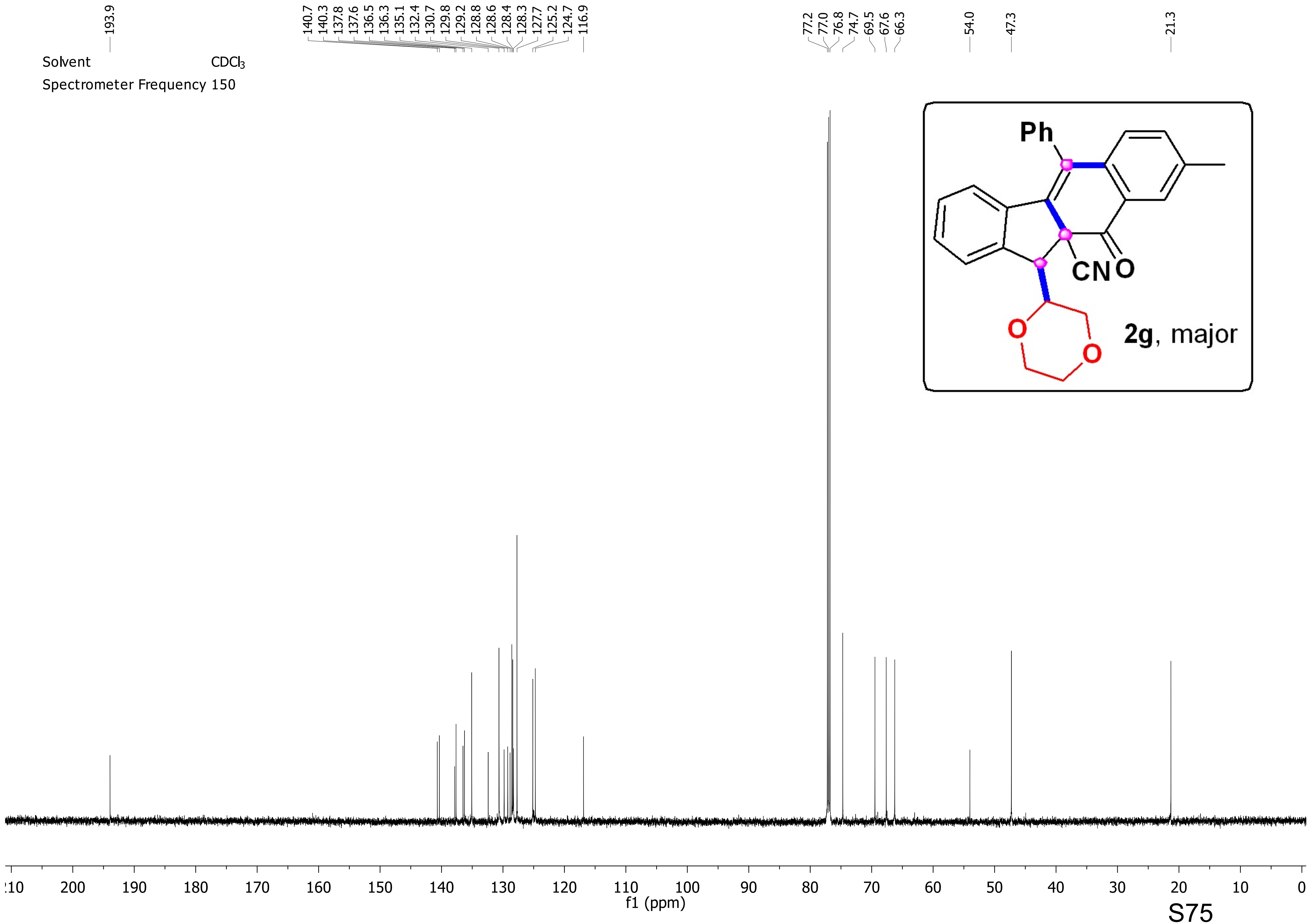

193.9
140.7
140.3
137.8
137.6
136.5
136.3
135.1
132.4
130.7
129.8
129.2
128.8
128.6
128.4
128.3
127.7
125.2
124.7
116.9
77.2
77.0
76.8
74.7
69.5
67.6
66.3
54.0
47.3
21.3
Solvent CDCl3
Spectrometer Frequency 150
Ph
CN
O
O
O
2g, major
f1 (ppm)

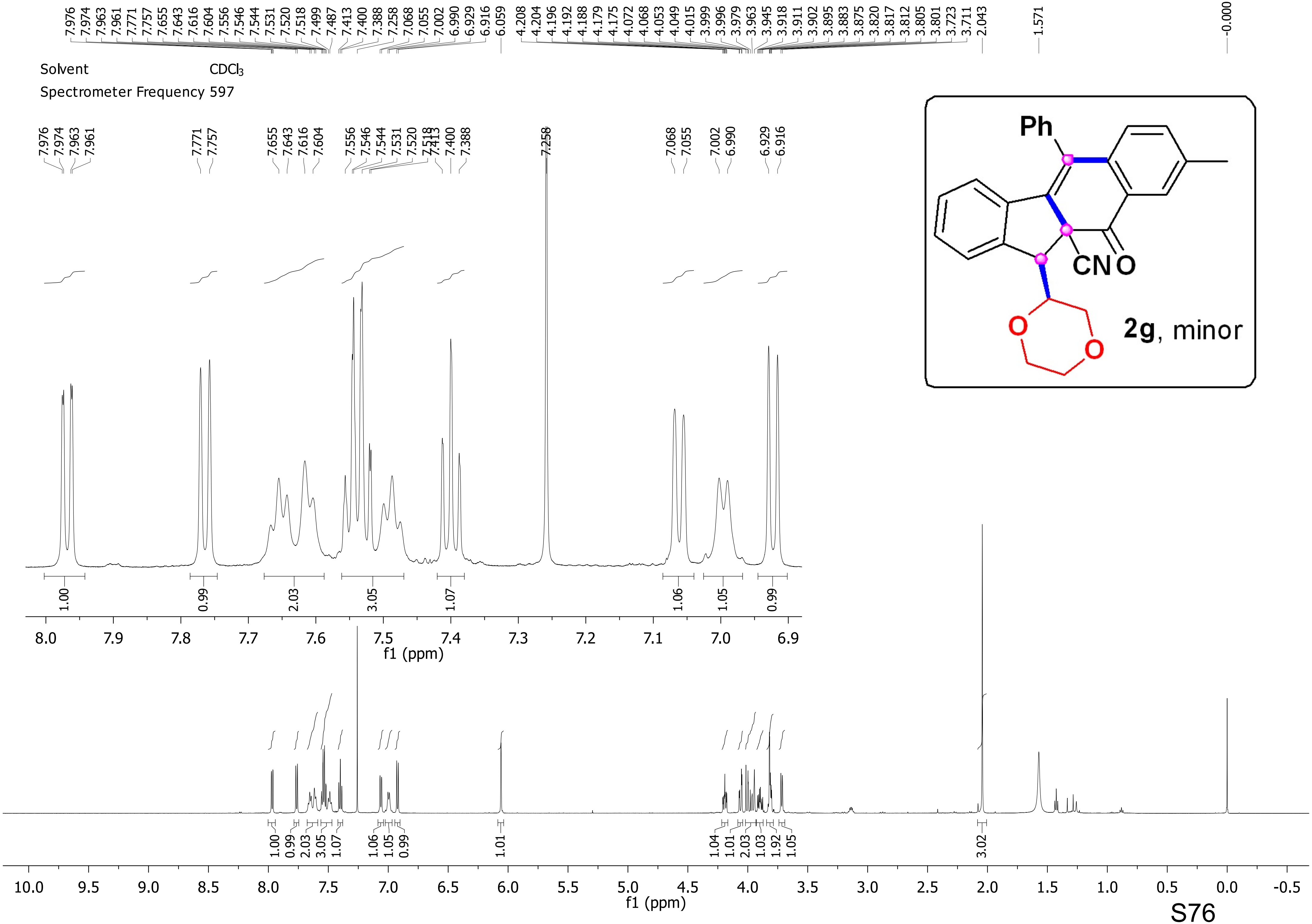
Solvent CDCl3
Spectrometer Frequency 597
f1 (ppm)
Ph
CN
O
O
O
2g, minor

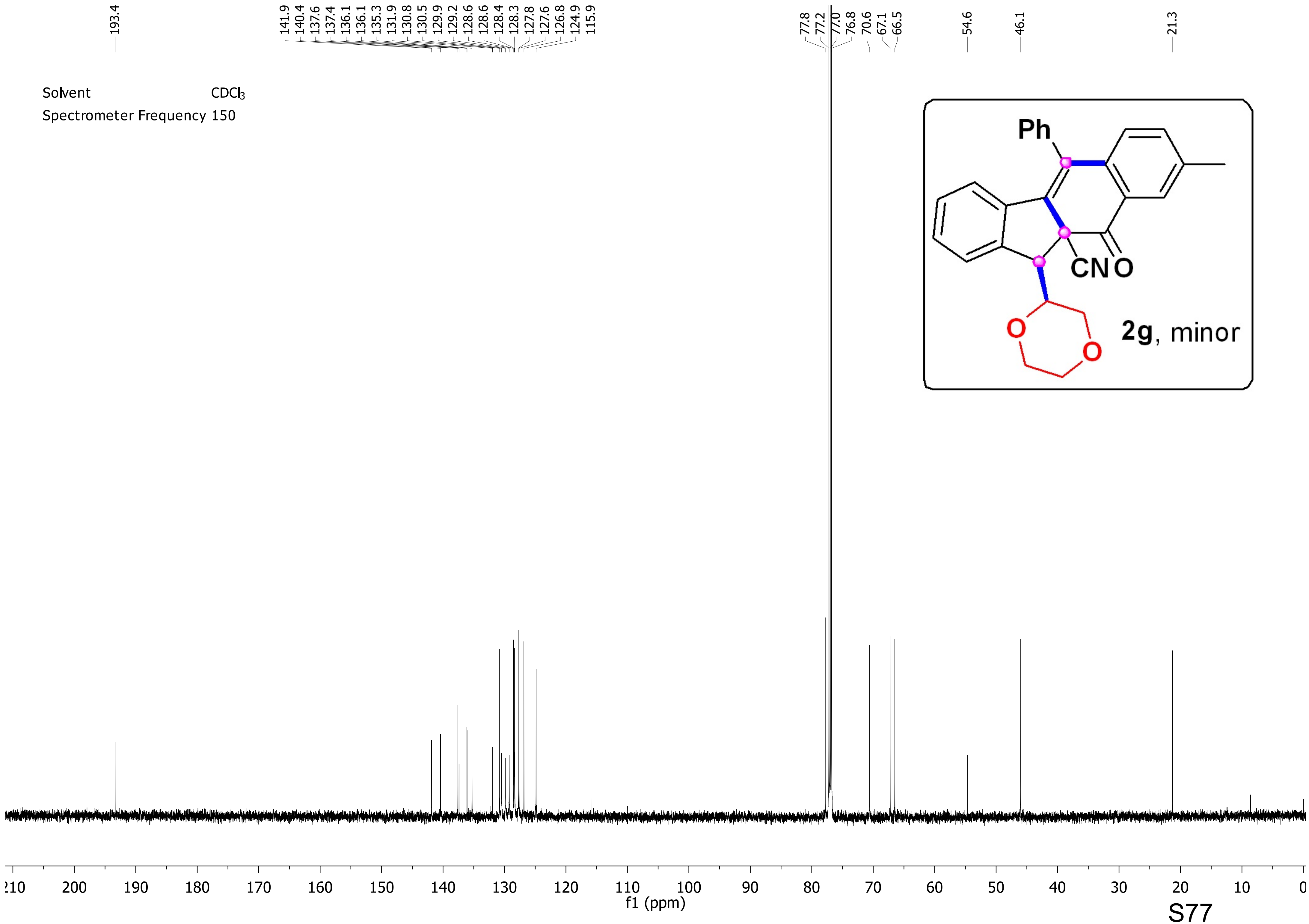
193.4
141.9
140.4
137.6
137.4
136.1
136.1
135.3
131.9
130.8
130.5
129.9
129.2
128.6
128.6
128.4
128.3
127.8
127.6
126.8
124.9
115.9
77.8
77.2
77.0
76.8
70.6
67.1
66.5
54.6
46.1
21.3
Solvent CDCl3
Spectrometer Frequency 150
Ph
CN
O
O
O
2g, minor
f1 (ppm)

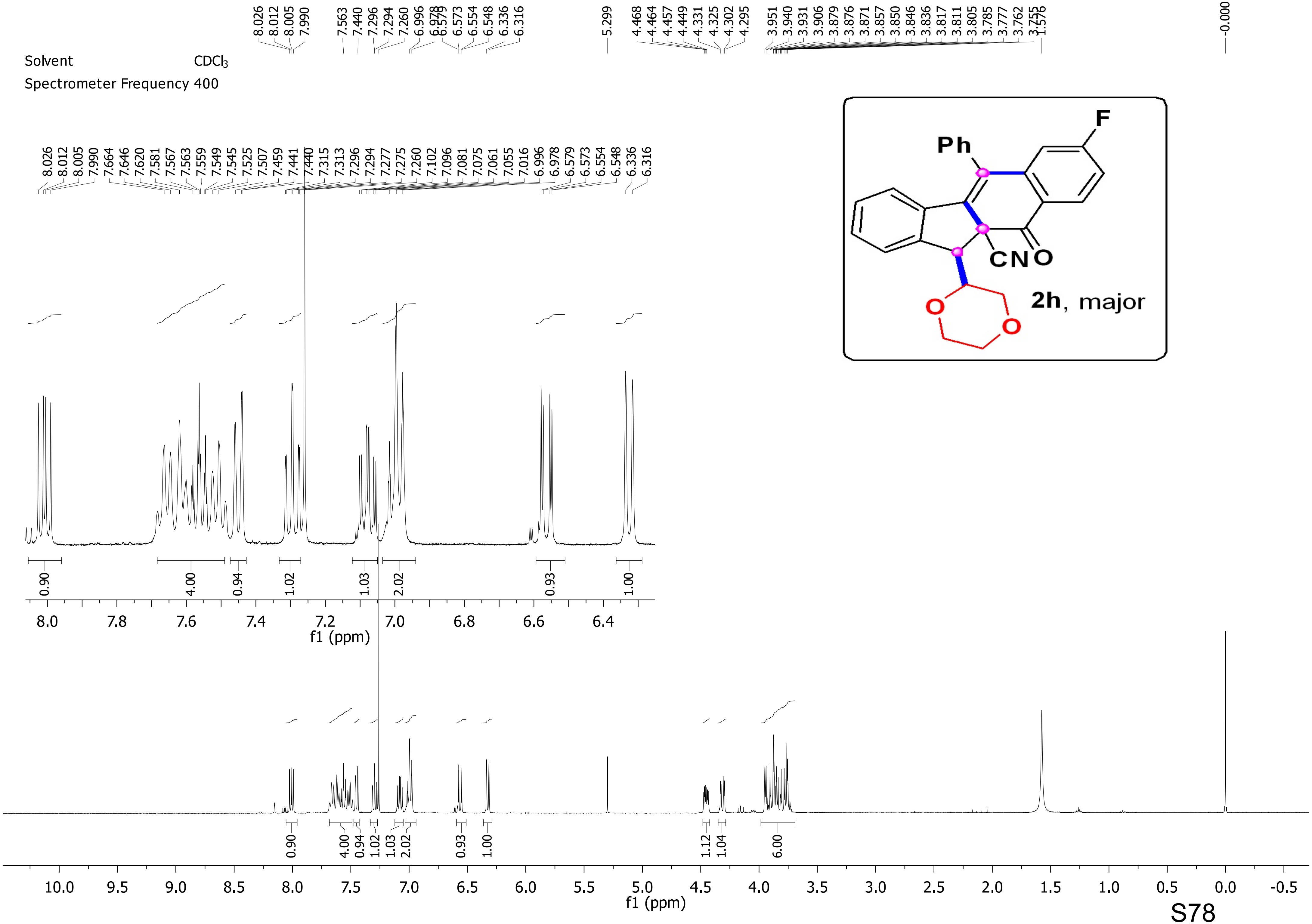
Solvent CDCl3
Spectrometer Frequency 400
8.026 8.012 8.005 7.990 7.563 7.440 7.296 7.294 7.260 6.996 6.978 6.579 6.573 6.554 6.548 6.336 6.316 5.299 4.468 4.464 4.457 4.449 4.331 4.325 4.302 4.295 3.951 3.940 3.931 3.906 3.879 3.876 3.871 3.857 3.850 3.846 3.836 3.817 3.811 3.805 3.785 3.777 3.762 3.755 1.576 -0.000
Ph
F
CN
O
2h, major
f1 (ppm)

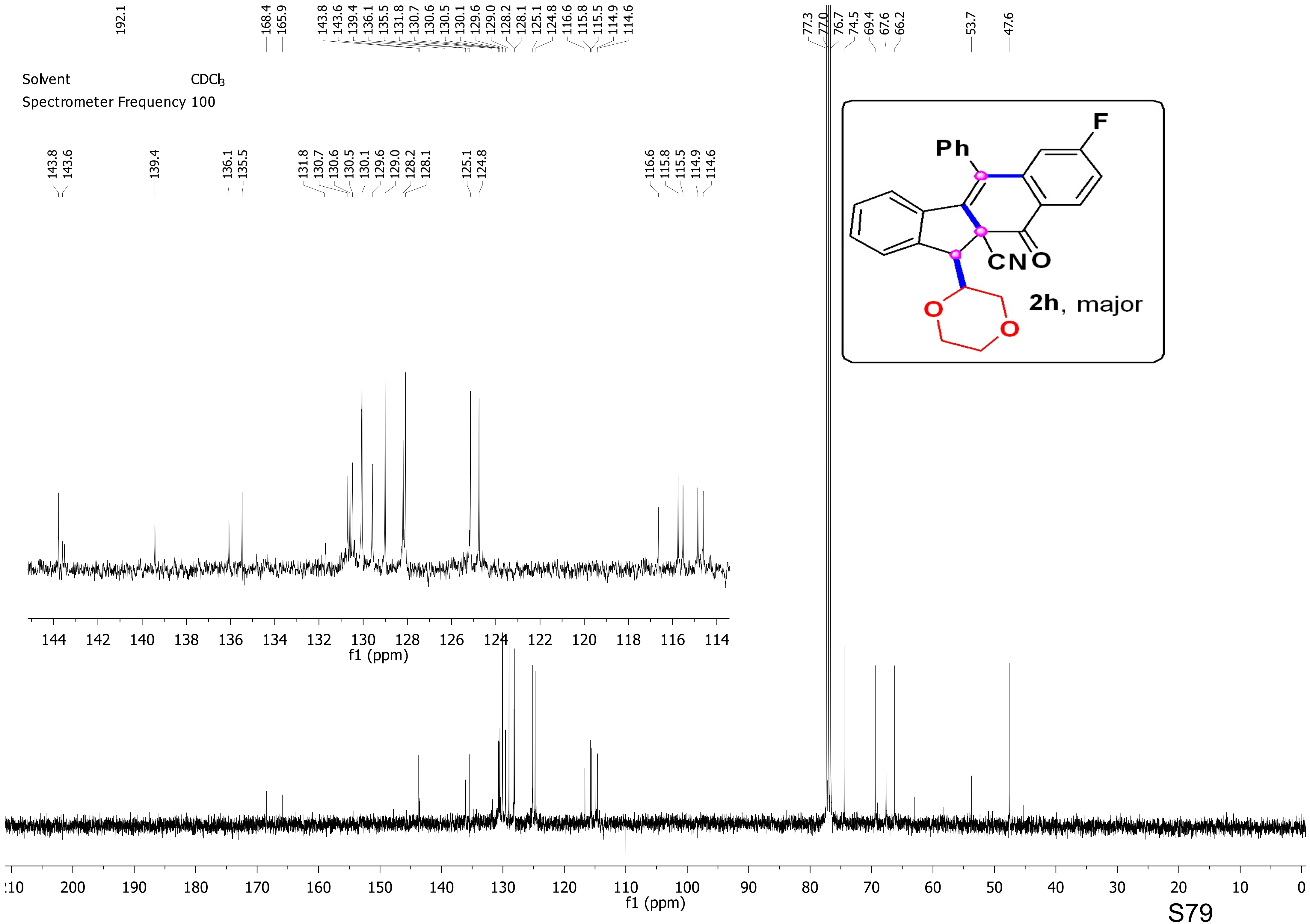
192.1
168.4
165.9
143.8
143.6
139.4
136.1
135.5
131.8
130.7
130.6
130.5
130.1
129.6
129.0
128.2
128.1
125.1
124.8
116.6
115.8
115.5
114.9
114.6
77.3
77.0
76.7
74.5
69.4
67.6
66.2
53.7
47.6
Solvent CDCl3
Spectrometer Frequency 100
Ph
F
CN
O
2h, major
f1 (ppm)

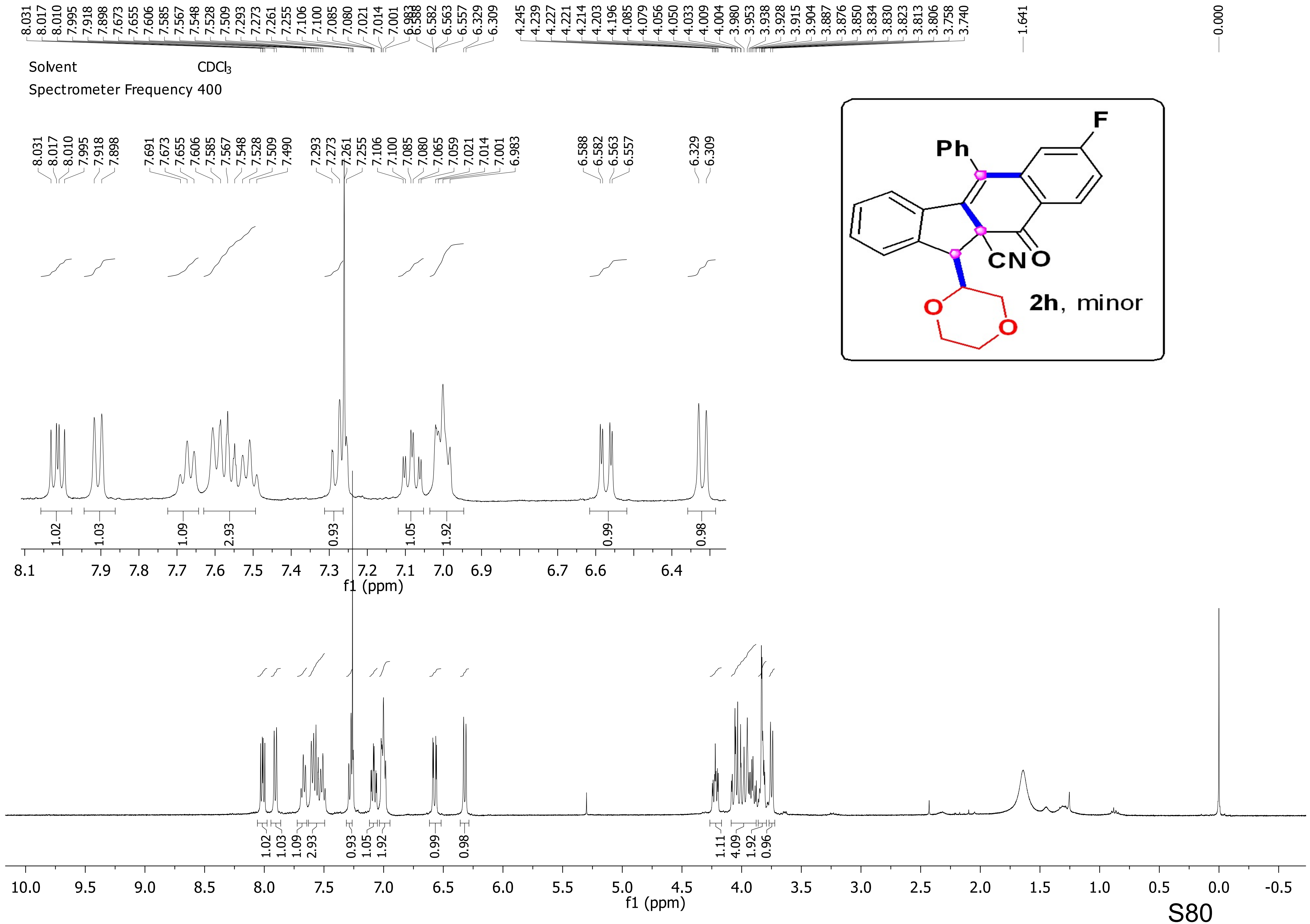
8.031 8.017 8.010 7.995 7.918 7.898 7.673 7.655 7.606 7.585 7.567 7.548 7.528 7.509 7.293 7.273 7.261 7.255 7.106 7.100 7.085 7.080 7.021 7.014 7.001 6.983 6.588 6.582 6.563 6.557 6.329 6.309
4.245 4.239 4.227 4.221 4.214 4.203 4.196 4.085 4.079 4.056 4.050 4.033 4.009 4.004 3.980 3.953 3.938 3.928 3.915 3.904 3.887 3.876 3.850 3.834 3.830 3.823 3.813 3.806 3.758 3.740
1.641
0.000
Solvent CDCl3
Spectrometer Frequency 400
8.031 8.017 8.010 7.995 7.918 7.898
7.691 7.673 7.655 7.606 7.585 7.567 7.548 7.528 7.509 7.490
7.293 7.273 7.261 7.255 7.106 7.100 7.085 7.080 7.065 7.059 7.021 7.014 7.001 6.983
6.588 6.582 6.563 6.557
6.329 6.309
F
Ph
CN O
O
O
2h, minor
1.02 1.03 1.09 2.93 0.93 1.05 1.92 0.99 0.98
8.1 7.9 7.8 7.7 7.6 7.5 7.4 7.3 7.2 7.1 7.0 6.9 6.7 6.6 6.4
f1 (ppm)
1.02 1.03 1.09 2.93 0.93 1.05 1.92 0.99 0.98 1.11 4.09 1.92 0.96
10.0 9.5 9.0 8.5 8.0 7.5 7.0 6.5 6.0 5.5 5.0 4.5 4.0 3.5 3.0 2.5 2.0 1.5 1.0 0.5 0.0 -0.5
f1 (ppm)

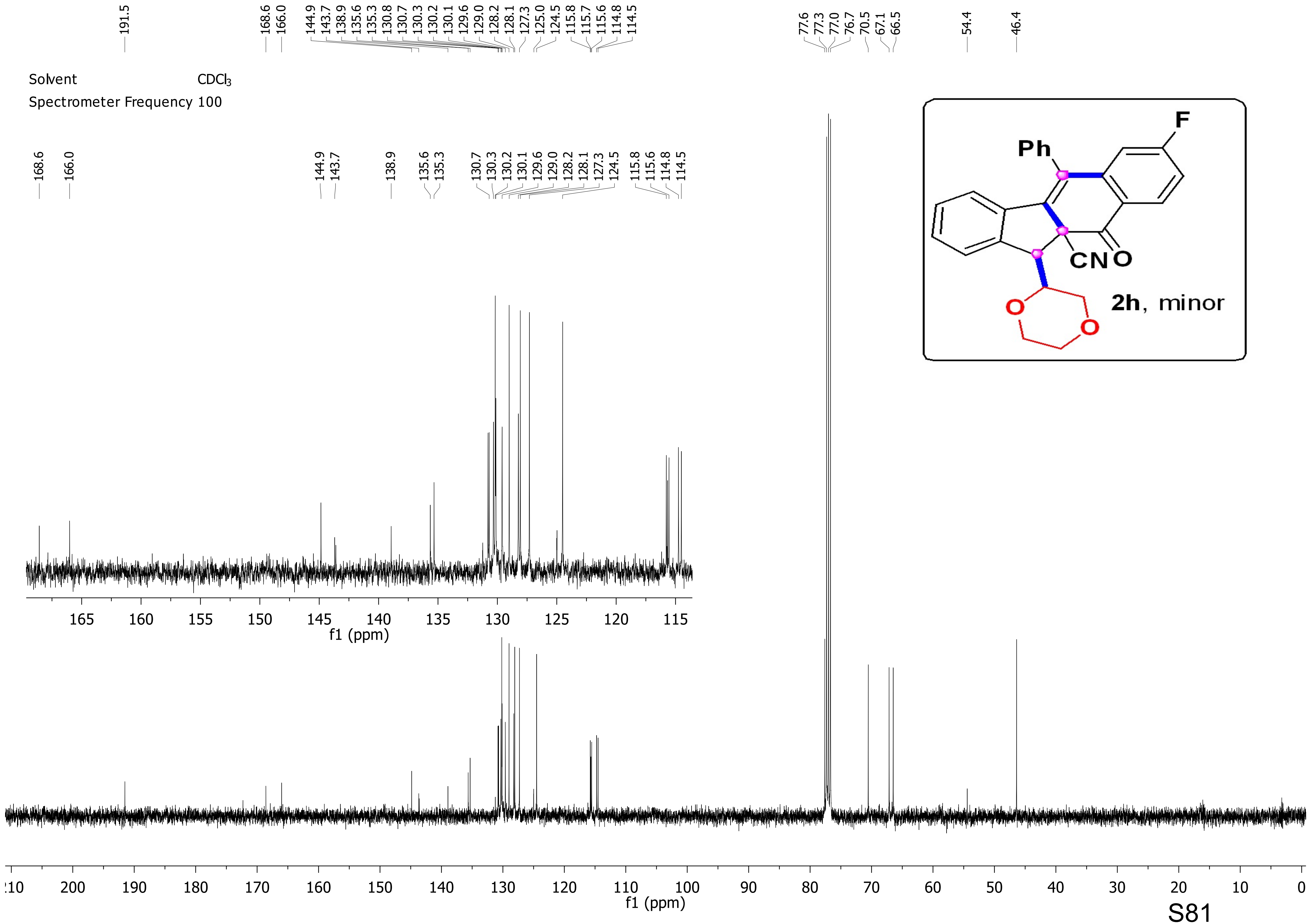
191.5
168.6
166.0
144.9
143.7
138.9
135.6
135.3
130.8
130.7
130.3
130.2
130.1
129.6
129.0
128.2
128.1
127.3
125.0
124.5
115.8
115.7
115.6
114.8
114.5
77.6
77.3
77.0
76.7
70.5
67.1
66.5
54.4
46.4
Solvent CDCl3
Spectrometer Frequency 100
Ph
F
CN
O
2h, minor
f1 (ppm)
f1 (ppm)

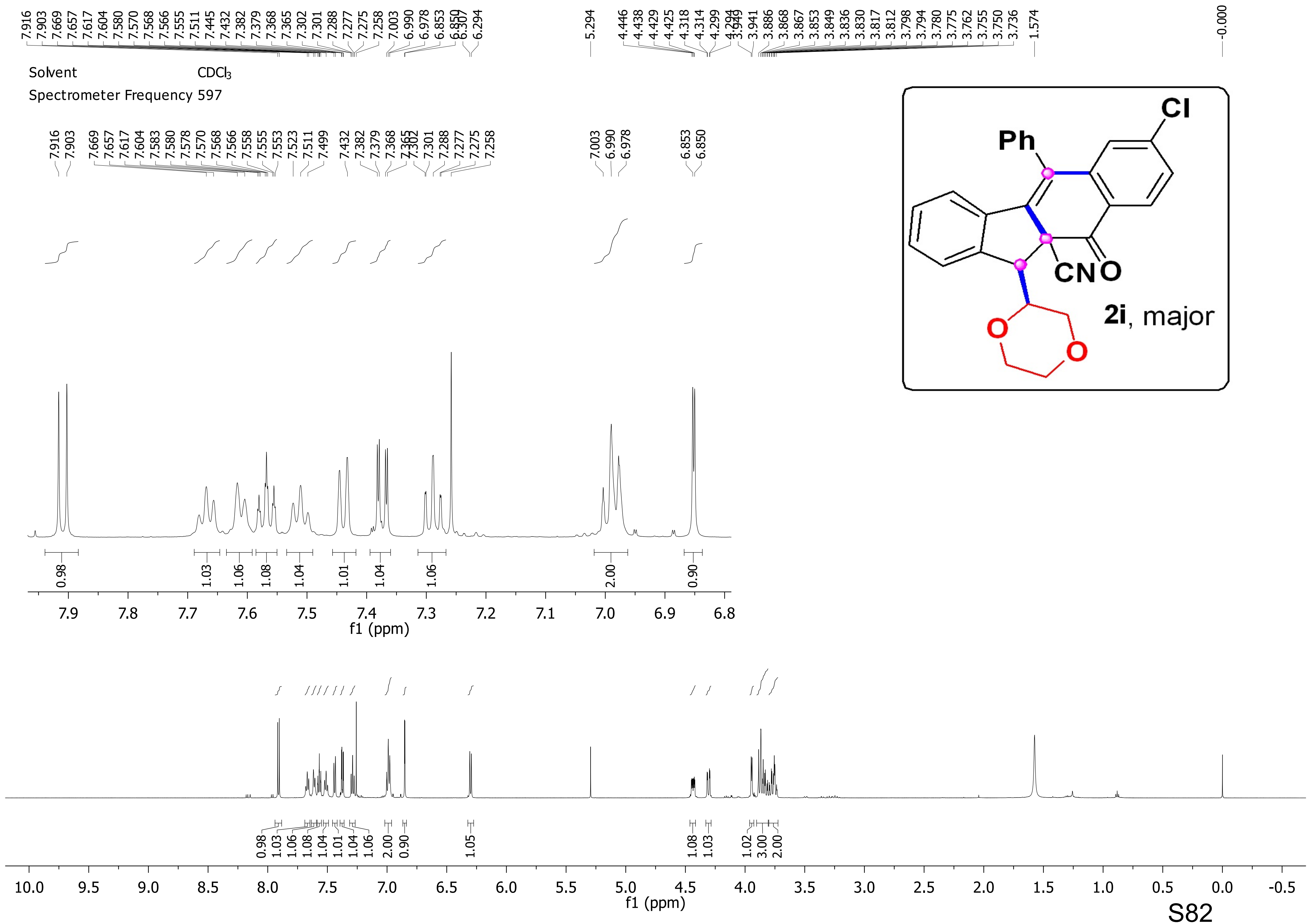

7.916 7.903 7.669 7.657 7.617 7.604 7.580 7.570 7.568 7.566 7.555 7.511 7.445 7.432 7.382 7.379 7.368 7.365 7.302 7.301 7.288 7.277 7.275 7.258 7.003 6.990 6.978 6.853 6.850 6.307 6.294
5.294
4.446 4.438 4.429 4.425 4.318 4.314 4.299 4.294 3.949 3.941 3.886 3.868 3.867 3.853 3.849 3.836 3.830 3.817 3.812 3.798 3.794 3.780 3.775 3.762 3.755 3.750 3.736
1.574
-0.000
Solvent CDCl3
Spectrometer Frequency 597
Ph
Cl
CN
O
2i, major
f1 (ppm)

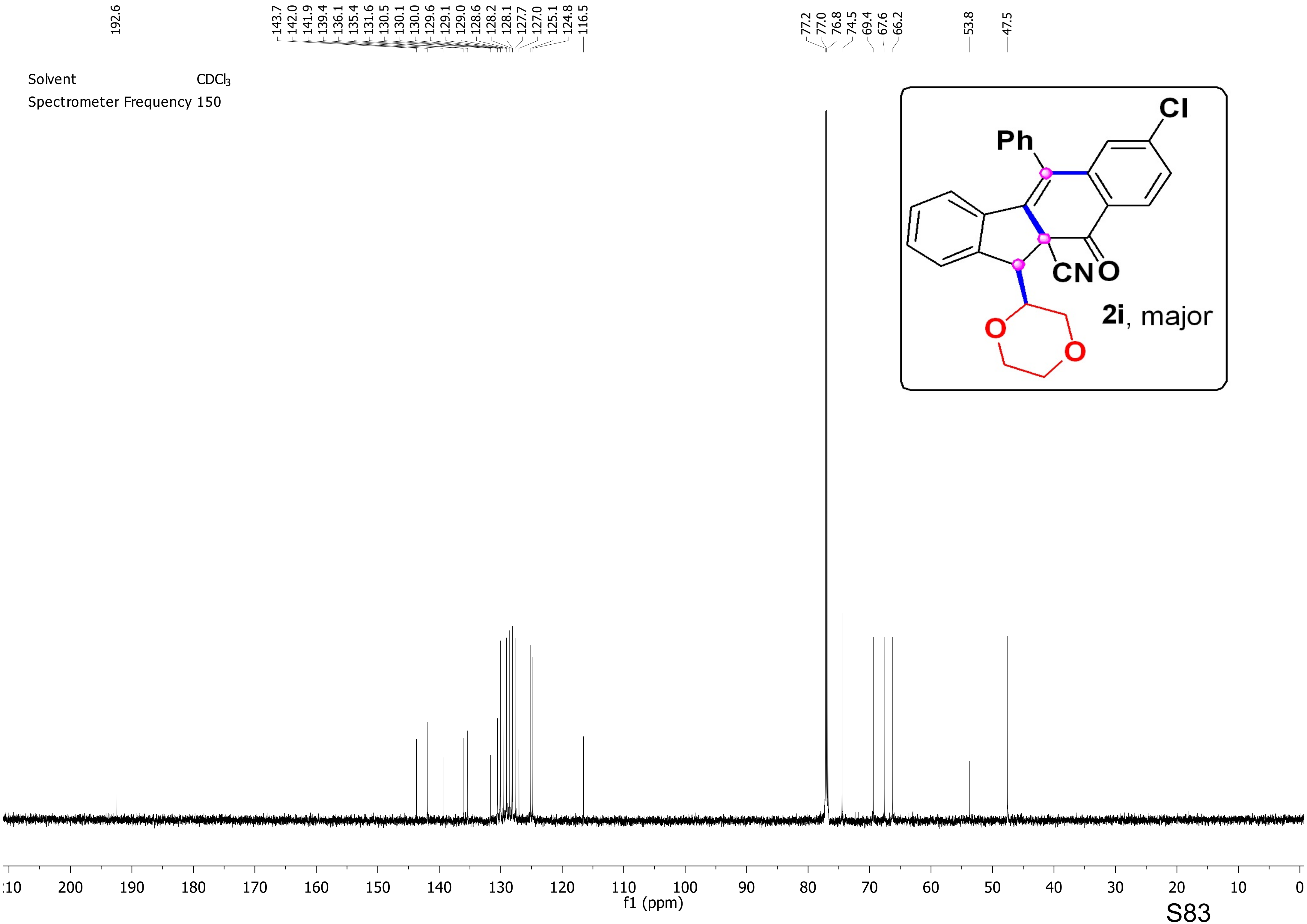
192.6
143.7 142.0 141.9 139.4 136.1 135.4 131.6 130.5 130.1 130.0 129.6 129.1 129.0 128.6 128.2 128.1 127.7 127.0 125.1 124.8 116.5
77.2 77.0 76.8 74.5 69.4 67.6 66.2
53.8
47.5
Solvent CDCl3
Spectrometer Frequency 150
Cl
Ph
CN
O
O
O
2i, major
f1 (ppm)

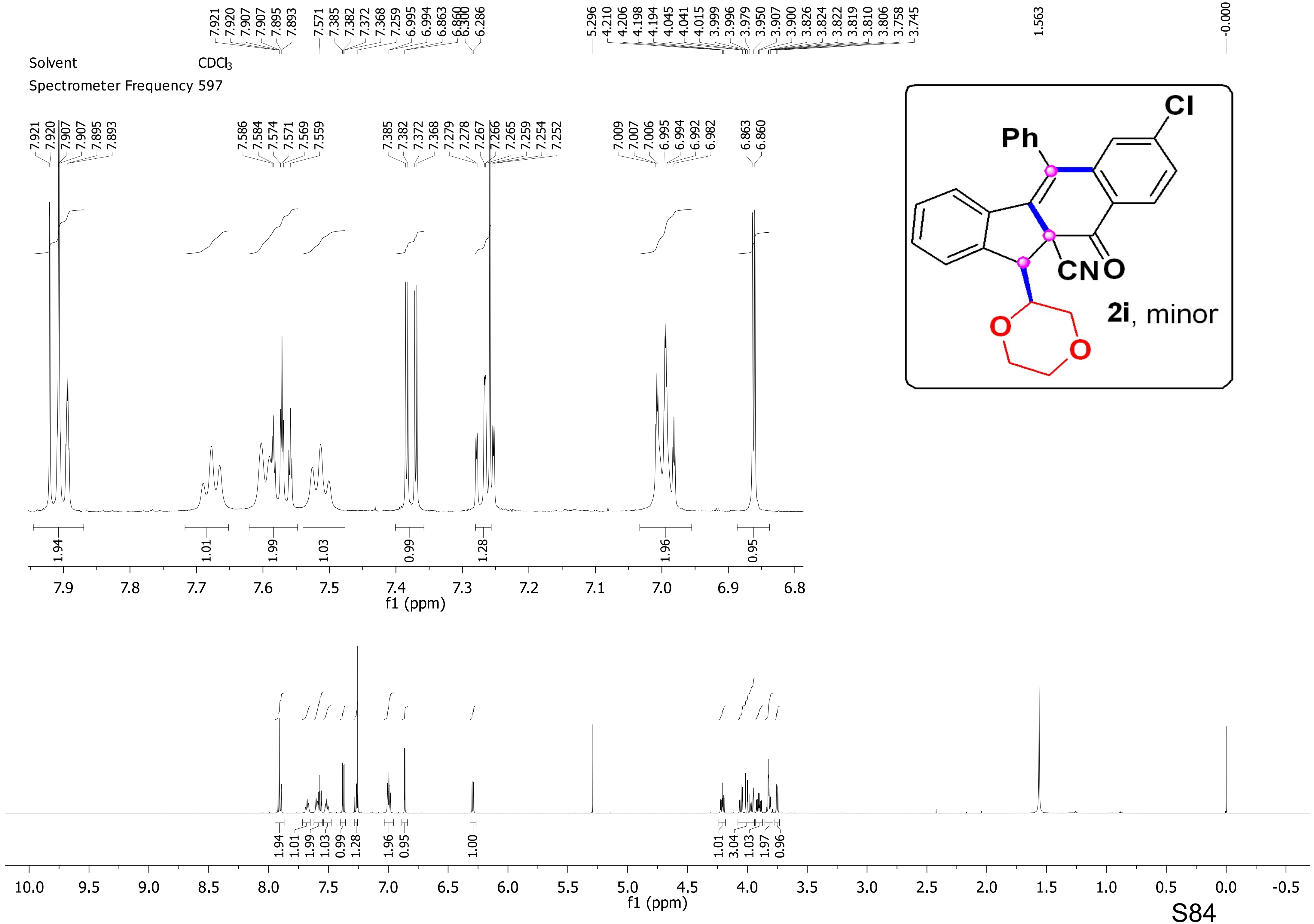
Solvent CDCl3
Spectrometer Frequency 597
7.921 7.920 7.907 7.907 7.895 7.893 7.571 7.385 7.382 7.372 7.368 7.259 6.995 6.994 6.863 6.860 6.300 6.286
5.296 4.210 4.206 4.198 4.194 4.045 4.041 4.015 3.999 3.996 3.979 3.950 3.907 3.900 3.826 3.824 3.822 3.819 3.810 3.806 3.758 3.745
1.563
-0.000
7.921 7.920 7.907 7.907 7.895 7.893
7.586 7.584 7.574 7.571 7.569 7.559
7.385 7.382 7.372 7.368 7.279 7.278 7.267 7.266 7.265 7.259 7.254 7.252
7.009 7.007 7.006 6.995 6.994 6.992 6.982
6.863 6.860
1.94 1.01 1.99 1.03 0.99 1.28 1.96 0.95
f1 (ppm)
Ph
Cl
CN
O
O
O
2i, minor
1.94 1.01 1.99 1.03 0.99 1.28 1.96 0.95 1.00 1.01 3.04 1.03 1.97 0.96
f1 (ppm)

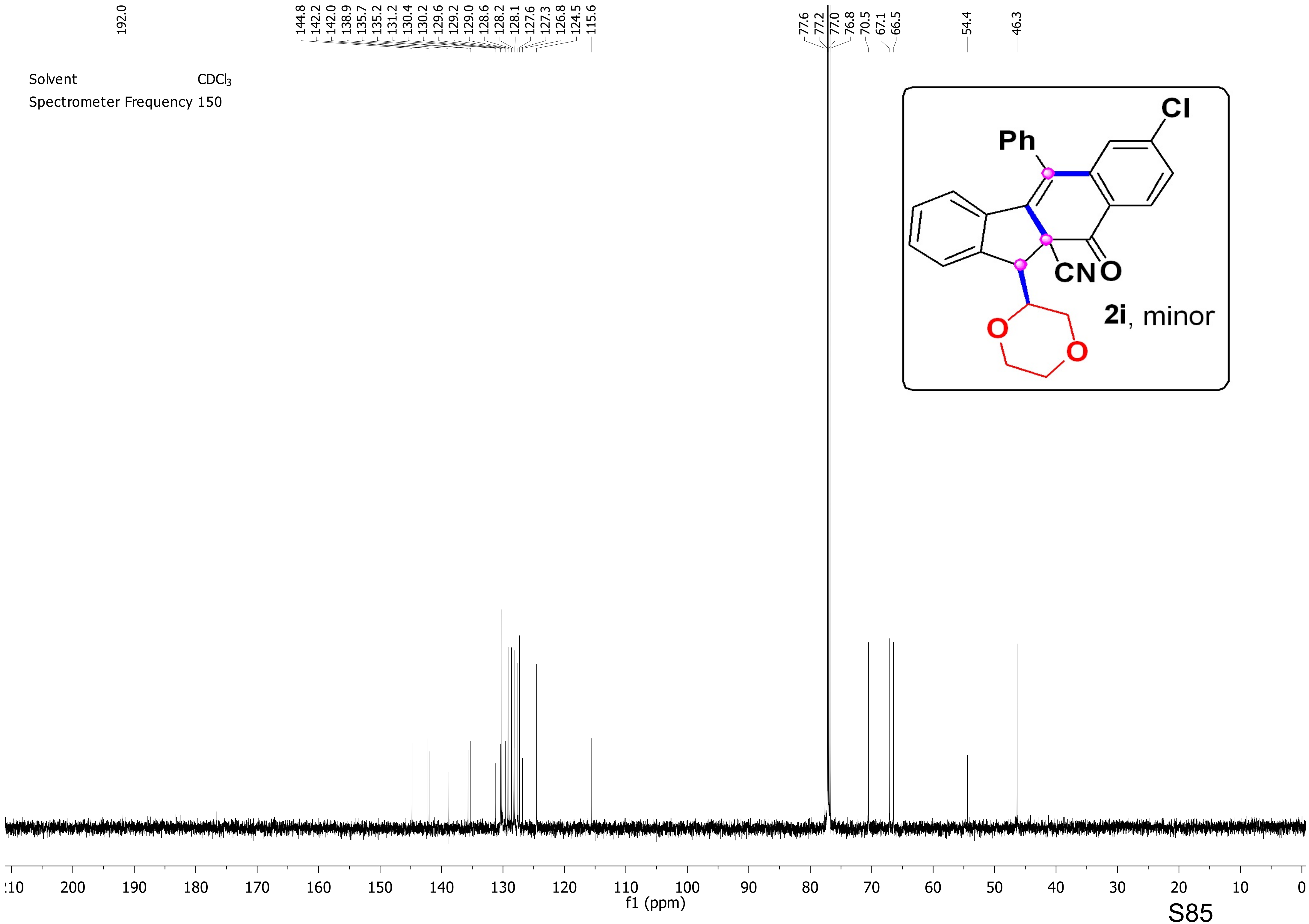
192.0
144.8
142.2
142.0
138.9
135.7
135.2
131.2
130.4
130.2
129.6
129.2
129.0
128.6
128.2
128.1
127.6
127.3
126.8
124.5
115.6
77.6
77.2
77.0
76.8
70.5
67.1
66.5
54.4
46.3
Solvent CDCl3
Spectrometer Frequency 150
Cl
Ph
CN
O
O
O
2i, minor
210 200 190 180 170 160 150 140 130 120 110 100 90 80 70 60 50 40 30 20 10 0
f1 (ppm)

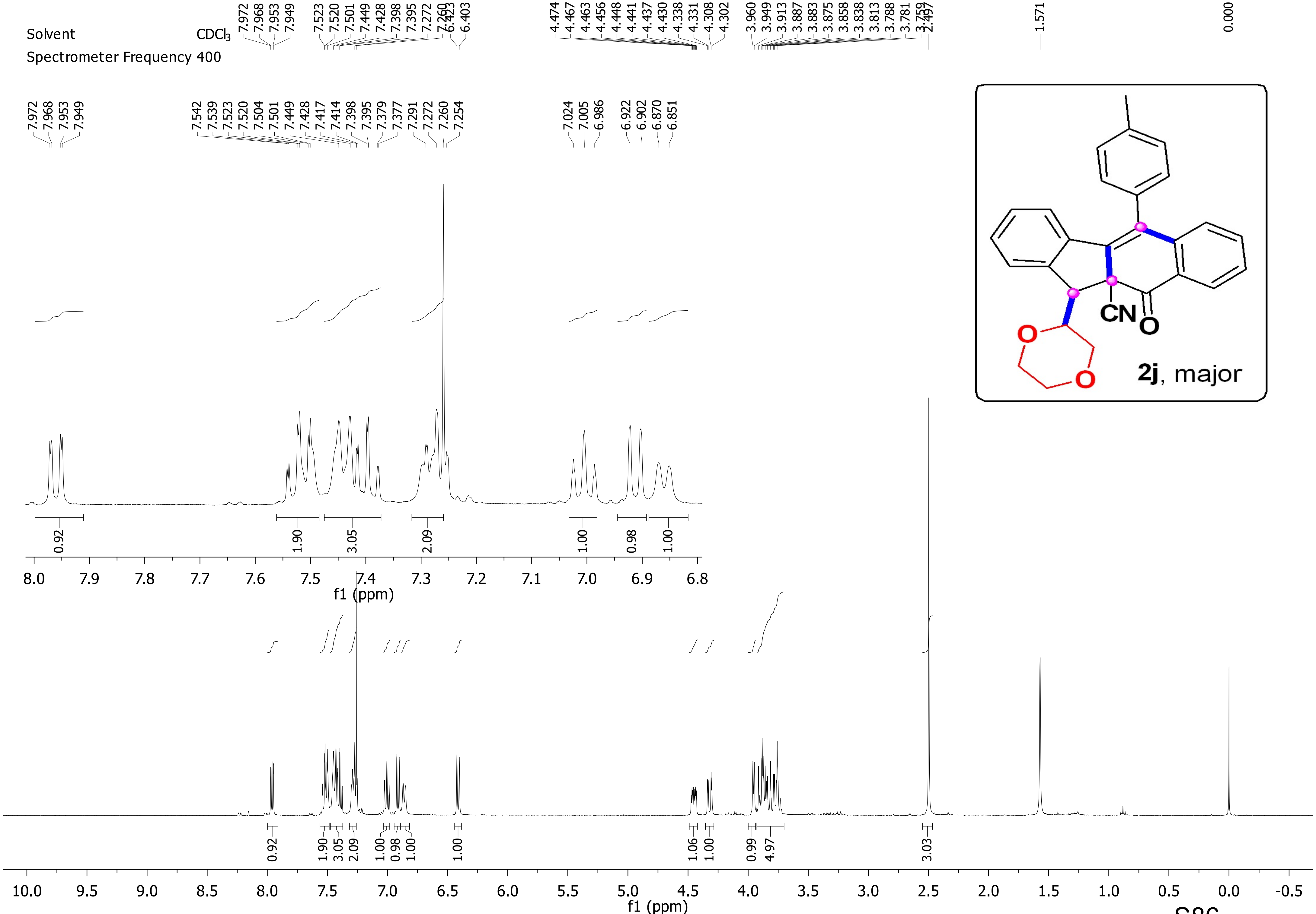

Solvent CDCl3
Spectrometer Frequency 400
7.972 7.968 7.953 7.949 7.523 7.520 7.501 7.449 7.428 7.398 7.395 7.272 7.260 6.423 6.403
4.474 4.467 4.463 4.456 4.448 4.441 4.437 4.430 4.338 4.331 4.308 4.302
3.960 3.949 3.913 3.887 3.883 3.875 3.858 3.838 3.813 3.788 3.781 3.759 2.497
1.571
0.000
7.972 7.968 7.953 7.949 7.542 7.539 7.523 7.520 7.504 7.501 7.449 7.428 7.417 7.414 7.398 7.395 7.379 7.377 7.291 7.272 7.260 7.254
7.024 7.005 6.986 6.922 6.902 6.870 6.851
0.92 1.90 3.05 2.09 1.00 0.98 1.00
f1 (ppm)
0.92 1.90 3.05 2.09 1.00 0.98 1.00 1.00 1.06 1.00 0.99 4.97 3.03
f1 (ppm)
CN
O
O
O
2j, major

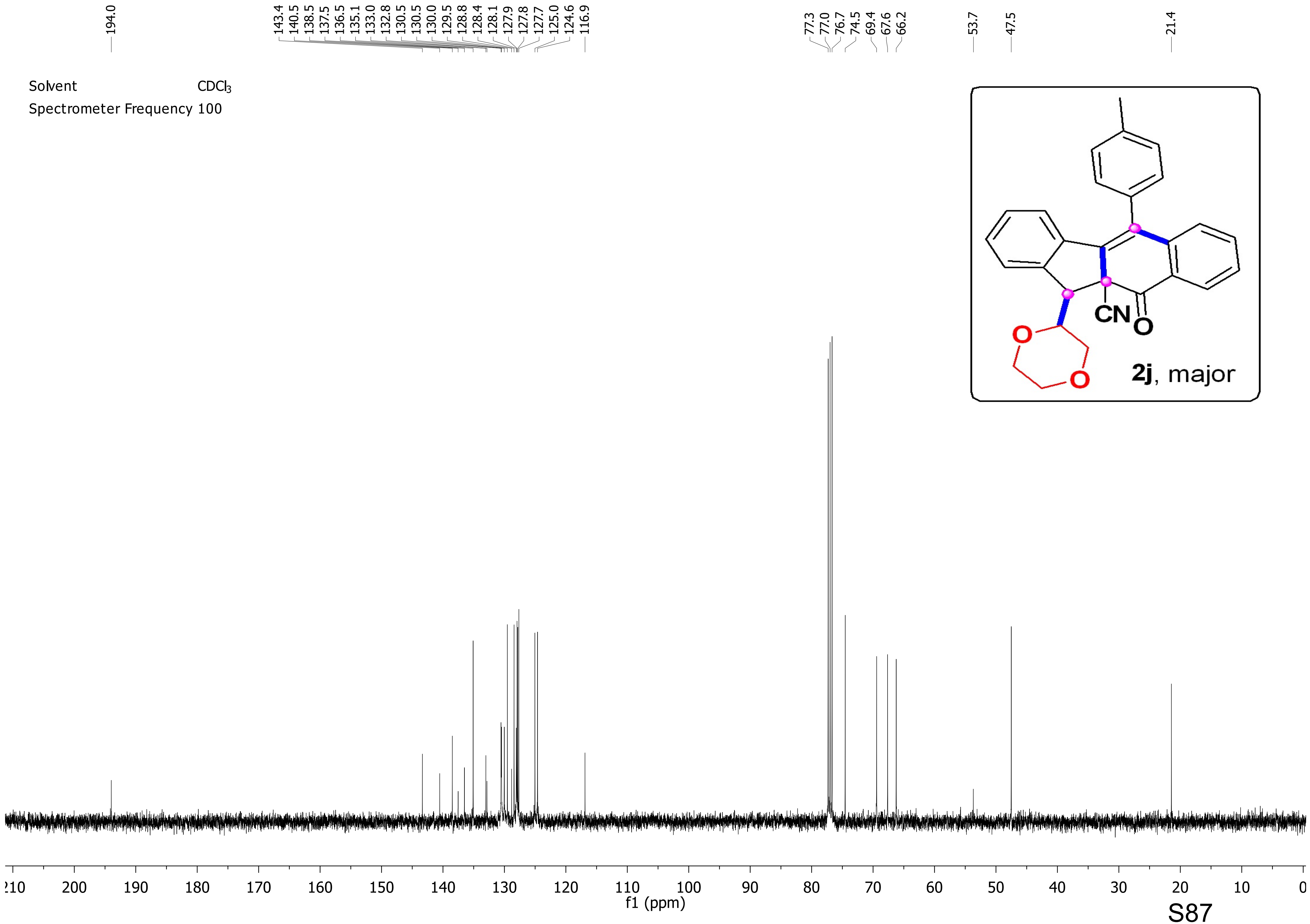
194.0
143.4
140.5
138.5
137.5
136.5
135.1
133.0
132.8
130.5
130.5
130.0
129.5
128.8
128.4
128.1
127.9
127.8
127.7
125.0
124.6
116.9
77.3
77.0
76.7
74.5
69.4
67.6
66.2
53.7
47.5
21.4
Solvent CDCl3
Spectrometer Frequency 100
CN
O
O
O
2j, major
210 200 190 180 170 160 150 140 130 120 110 100 90 80 70 60 50 40 30 20 10 0
f1 (ppm)

Solvent CDCl$_3$

Spectrometer Frequency 400

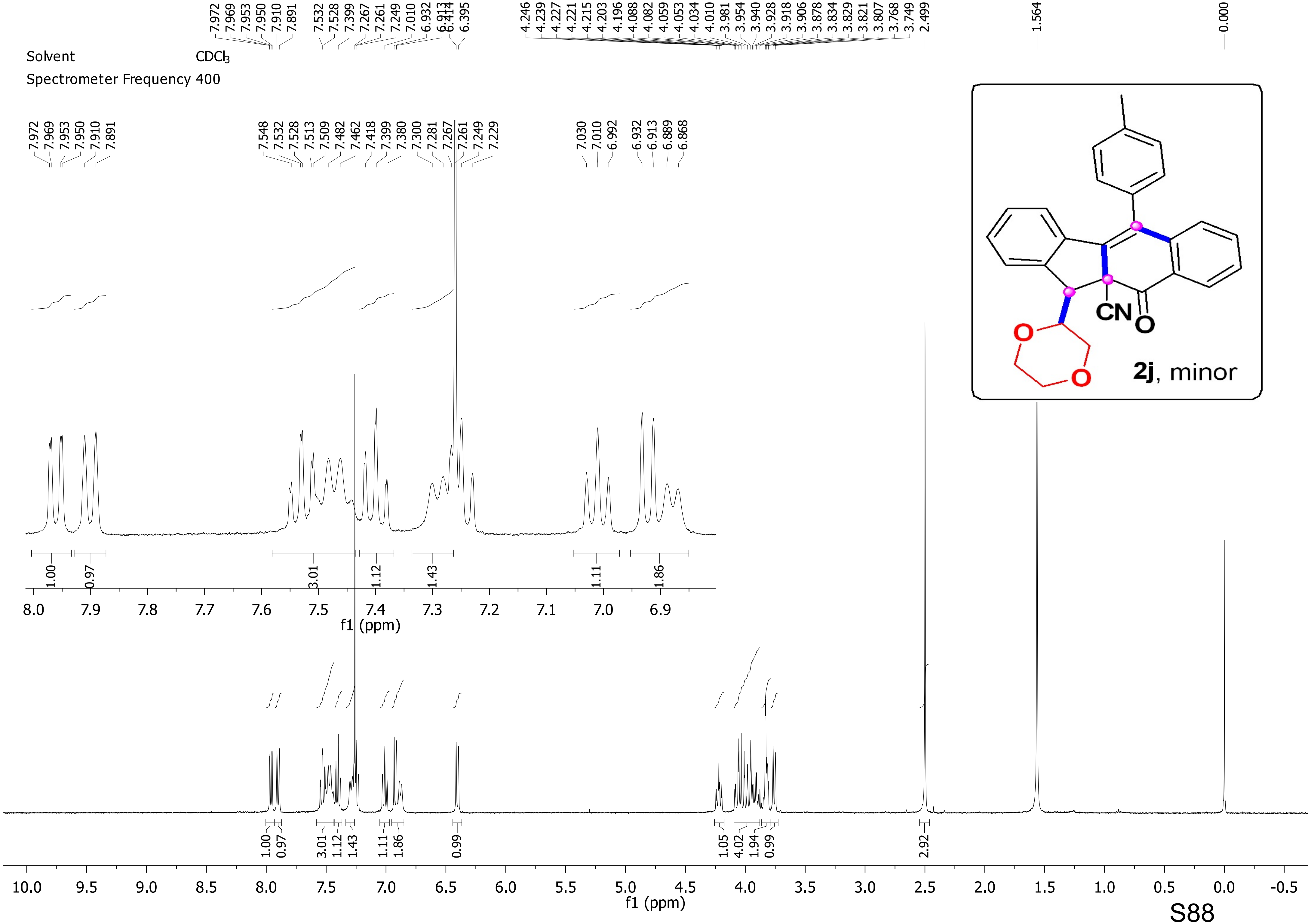

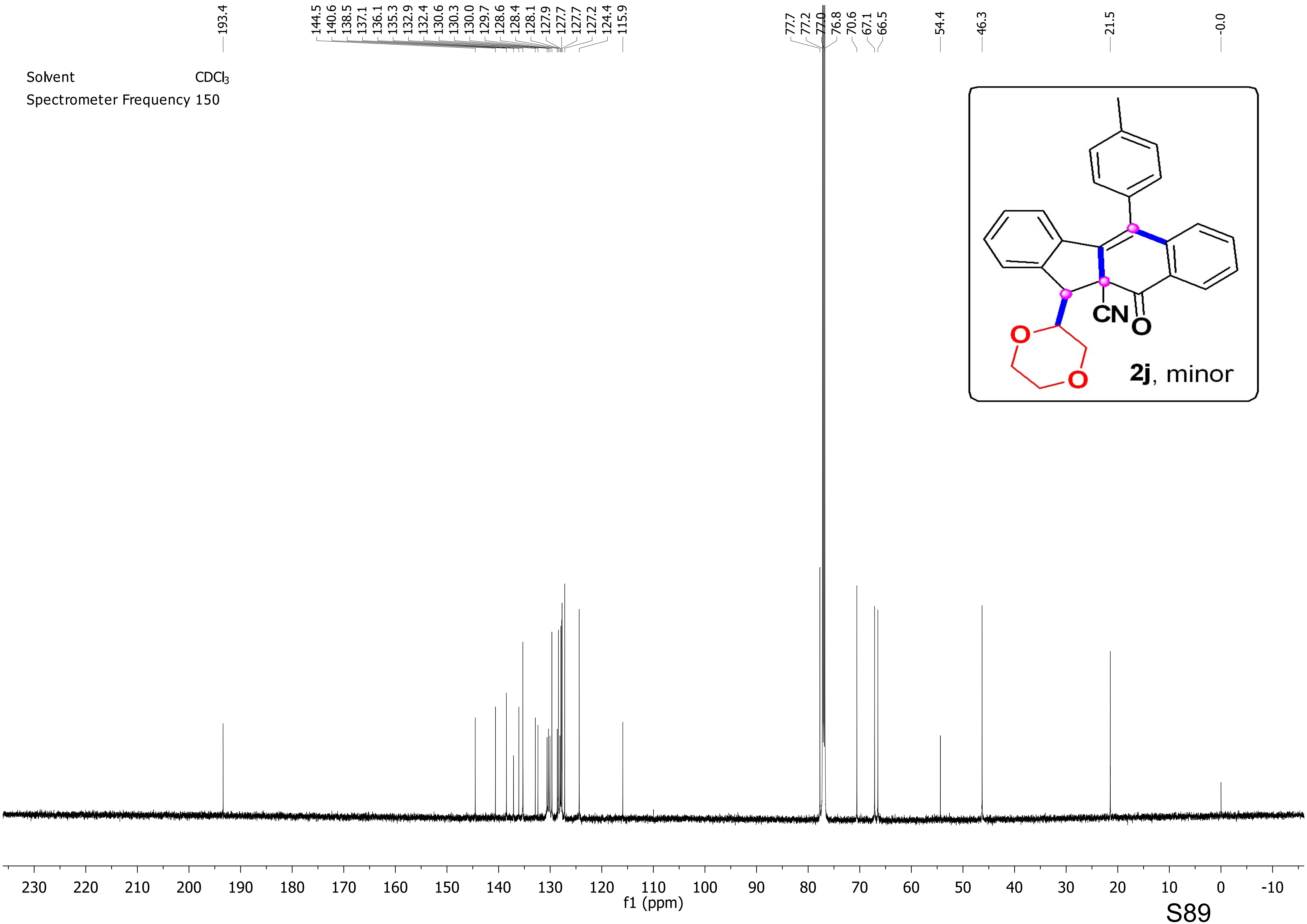
193.4
144.5
140.6
138.5
137.1
136.1
135.3
132.9
132.4
130.6
130.3
130.0
129.7
128.6
128.4
128.1
127.9
127.7
127.7
127.2
124.4
115.9
77.7
77.2
77.0
76.8
70.6
67.1
66.5
54.4
46.3
21.5
-0.0
Solvent CDCl3
Spectrometer Frequency 150
CN
O
O
O
2j, minor
230 220 210 200 190 180 170 160 150 140 130 120 110 100 90 80 70 60 50 40 30 20 10 0 -10
f1 (ppm)

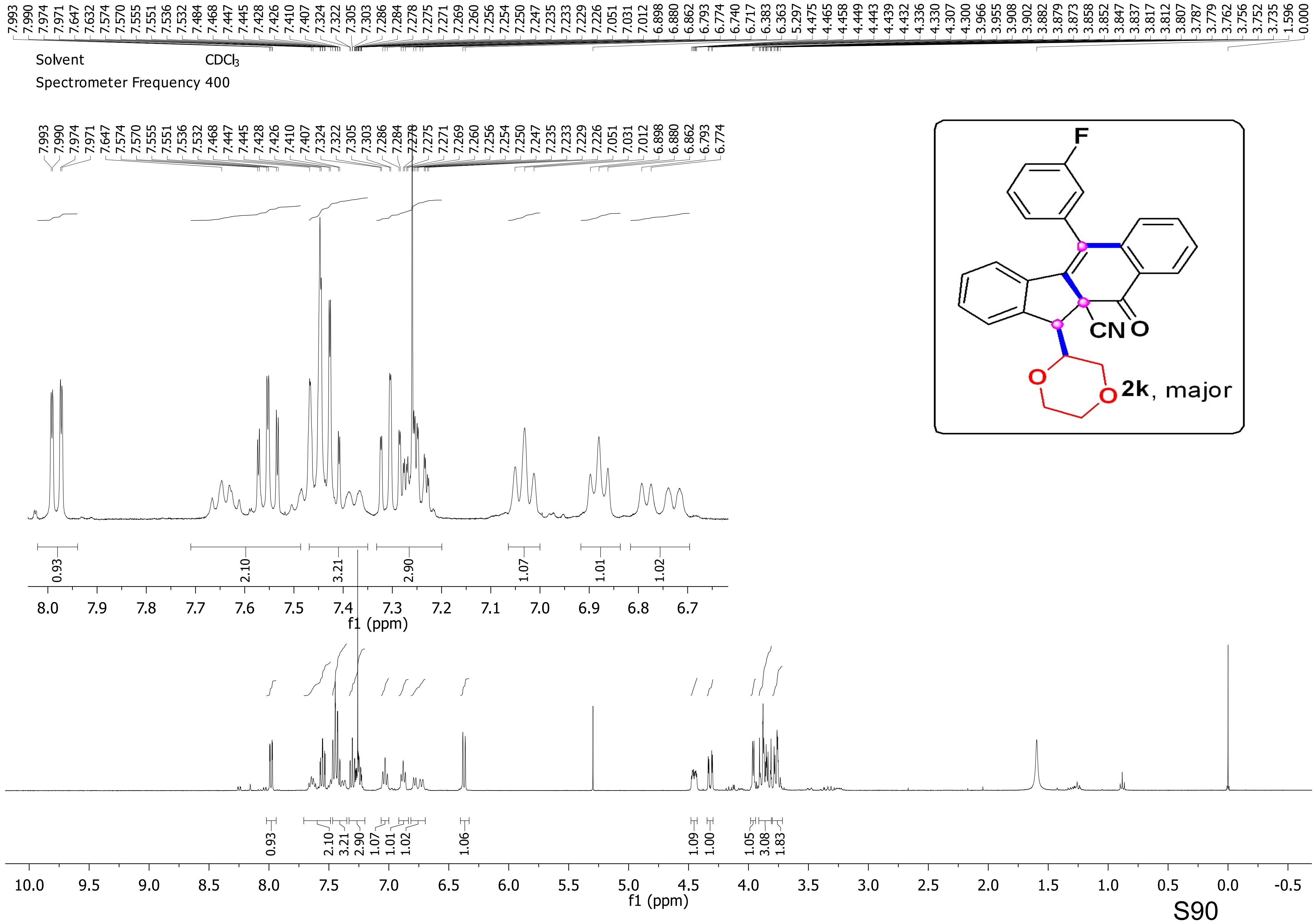
7.993 7.990 7.974 7.971 7.647 7.632 7.574 7.570 7.555 7.551 7.536 7.532 7.484 7.468 7.447 7.445 7.428 7.426 7.410 7.407 7.324 7.322 7.305 7.303 7.286 7.284 7.278 7.275 7.271 7.269 7.260 7.256 7.254 7.250 7.247 7.235 7.233 7.229 7.226 7.051 7.031 7.012 6.898 6.880 6.862 6.793 6.774 6.740 6.717 6.383 6.363 5.297 4.475 4.465 4.458 4.449 4.443 4.439 4.432 4.336 4.330 4.307 4.300 3.966 3.955 3.908 3.902 3.882 3.879 3.873 3.858 3.852 3.847 3.837 3.817 3.812 3.807 3.787 3.779 3.762 3.756 3.752 3.735 1.596 0.000
Solvent CDCl3
Spectrometer Frequency 400
F
CN
O
2k, major
f1 (ppm)

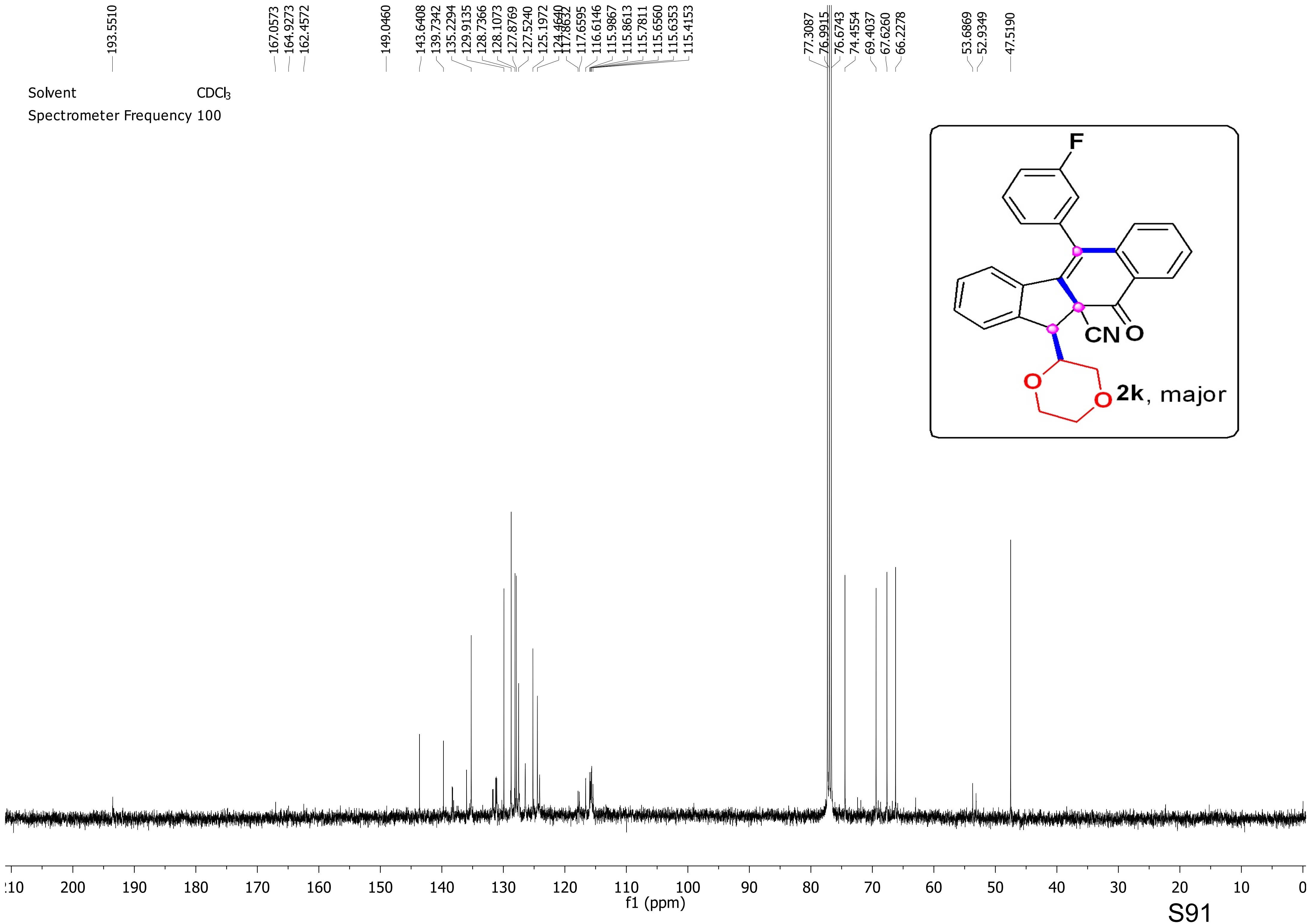
193.5510
167.0573
164.9273
162.4572
149.0460
143.6408
139.7342
135.2294
129.9135
128.7366
128.1073
127.8769
127.5240
125.1972
124.4640
117.8632
117.6595
116.6146
115.9867
115.8613
115.7811
115.6560
115.6353
115.4153
77.3087
76.9915
76.6743
74.4554
69.4037
67.6260
66.2278
53.6869
52.9349
47.5190
Solvent CDCl3
Spectrometer Frequency 100
F
CN
O
O
O
2k, major
210 200 190 180 170 160 150 140 130 120 110 100 90 80 70 60 50 40 30 20 10 0
f1 (ppm)

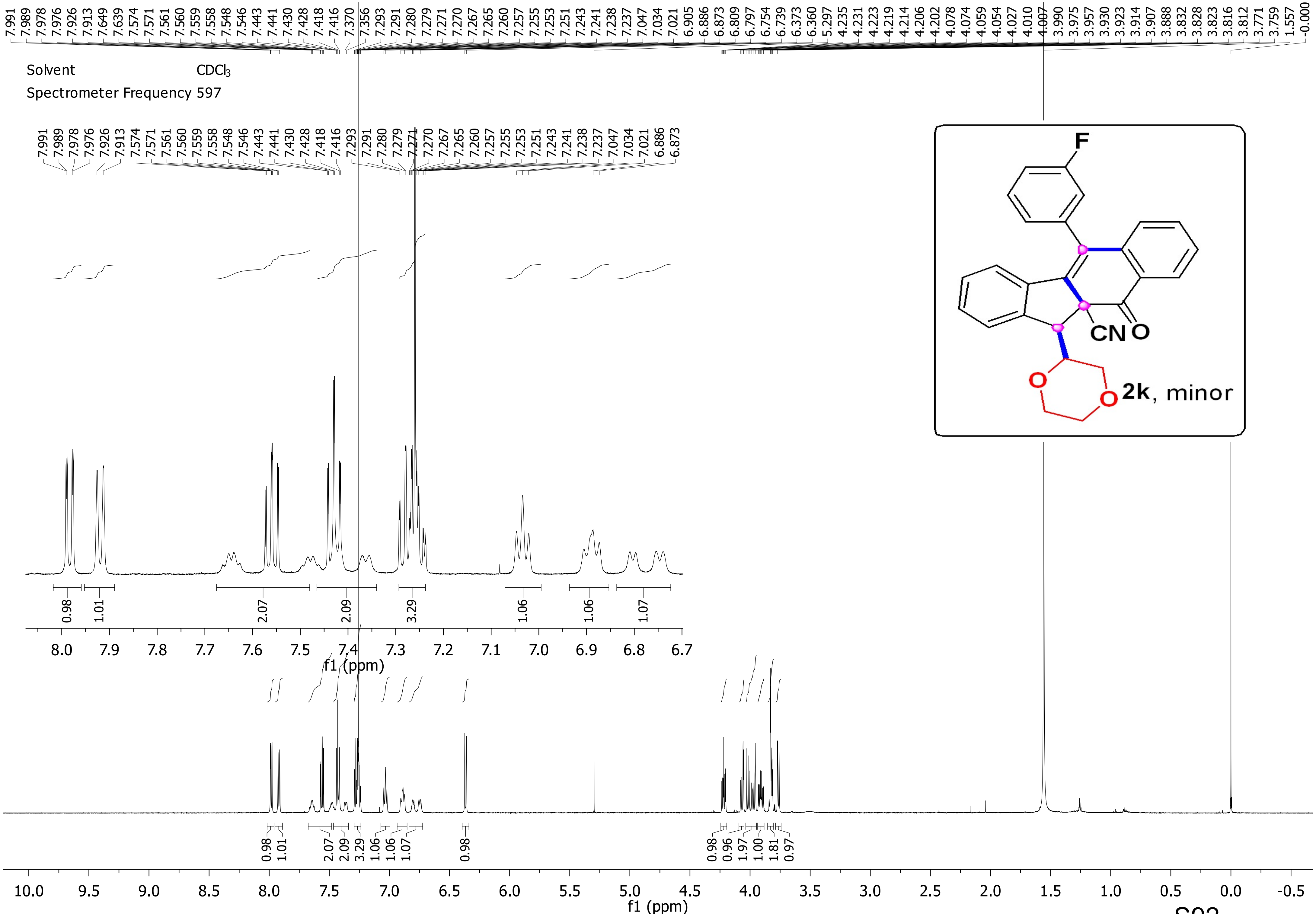
Solvent CDCl3
Spectrometer Frequency 597
7.991 7.989 7.978 7.976 7.926 7.913 7.649 7.639 7.574 7.571 7.561 7.560 7.559 7.558 7.548 7.546 7.443 7.441 7.430 7.428 7.418 7.416 7.370 7.356 7.293 7.291 7.280 7.279 7.271 7.270 7.267 7.265 7.260 7.257 7.255 7.253 7.251 7.243 7.241 7.238 7.237 7.047 7.034 7.021 6.905 6.886 6.873 6.809 6.797 6.754 6.739 6.373 6.360 5.297 4.235 4.231 4.223 4.219 4.214 4.206 4.202 4.078 4.074 4.059 4.054 4.027 4.010 4.007 3.990 3.975 3.957 3.930 3.923 3.914 3.907 3.888 3.832 3.828 3.823 3.816 3.812 3.771 3.759 1.557 -0.000
F
CN
O
2k, minor
f1 (ppm)
f1 (ppm)

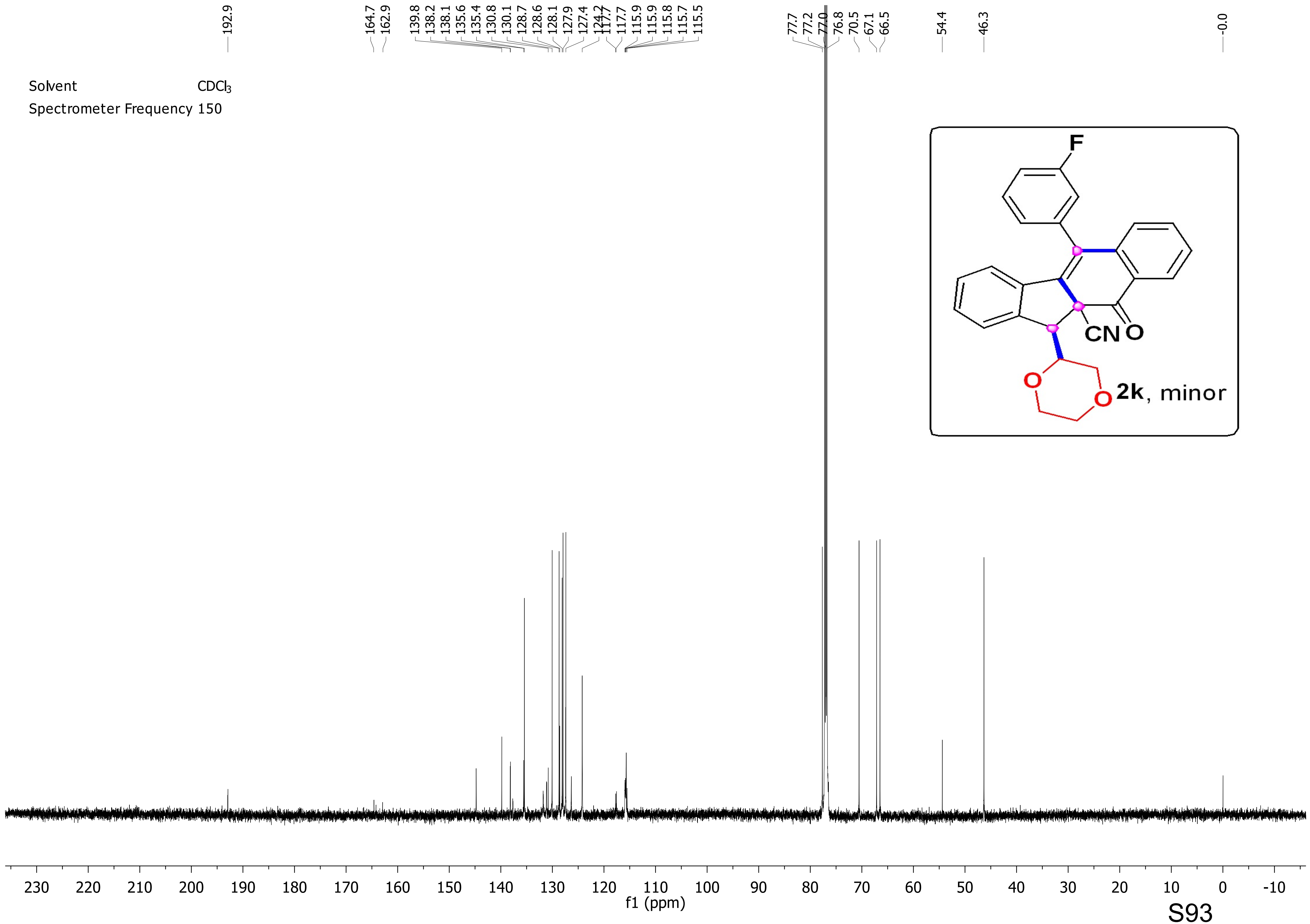

192.9
164.7
162.9
139.8
138.2
138.1
135.6
135.4
130.8
130.1
128.7
128.6
128.1
127.9
127.4
124.2
117.7
117.7
115.9
115.9
115.8
115.7
115.5
77.7
77.2
77.0
76.8
70.5
67.1
66.5
54.4
46.3
-0.0
Solvent CDCl3
Spectrometer Frequency 150
F
CN
O
O
O
2k, minor
f1 (ppm)

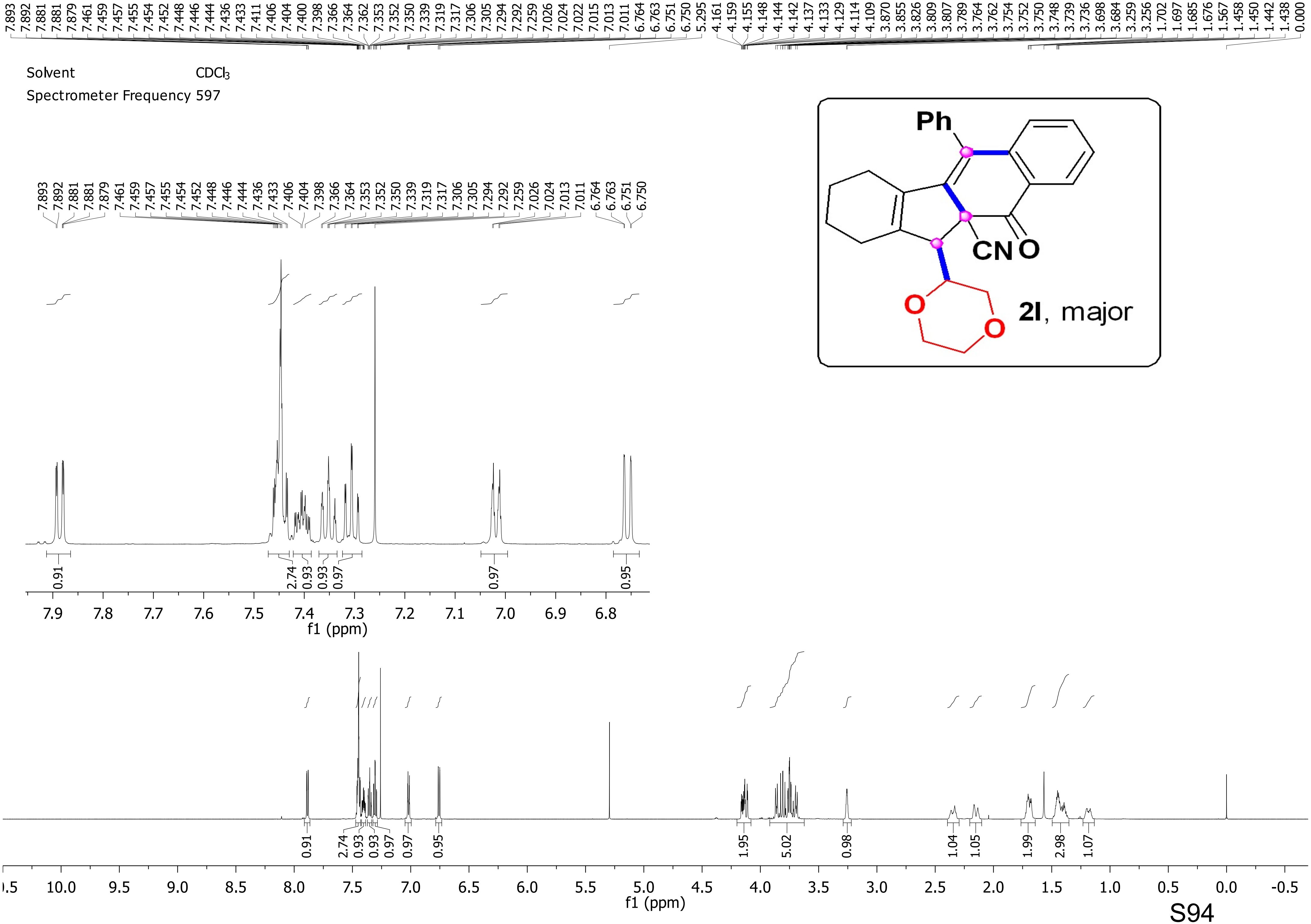

Solvent CDCl3
Spectrometer Frequency 597
7.893 7.892 7.881 7.881 7.879 7.461 7.459 7.457 7.455 7.454 7.452 7.448 7.446 7.444 7.436 7.433 7.411 7.406 7.404 7.400 7.398 7.366 7.364 7.362 7.353 7.352 7.350 7.339 7.319 7.317 7.306 7.305 7.294 7.292 7.259 7.026 7.024 7.022 7.015 7.013 7.011 6.764 6.763 6.751 6.750 5.295 4.161 4.159 4.155 4.148 4.144 4.142 4.137 4.133 4.129 4.114 4.109 3.870 3.855 3.826 3.809 3.807 3.789 3.764 3.762 3.754 3.752 3.750 3.748 3.739 3.736 3.698 3.684 3.259 3.256 1.702 1.697 1.685 1.676 1.567 1.458 1.450 1.442 1.438 0.000
Ph
CN
O
O
O
2l, major
0.91
2.74
0.93
0.93
0.97
0.97
0.95
1.95
5.02
0.98
1.04
1.05
1.99
2.98
1.07
f1 (ppm)

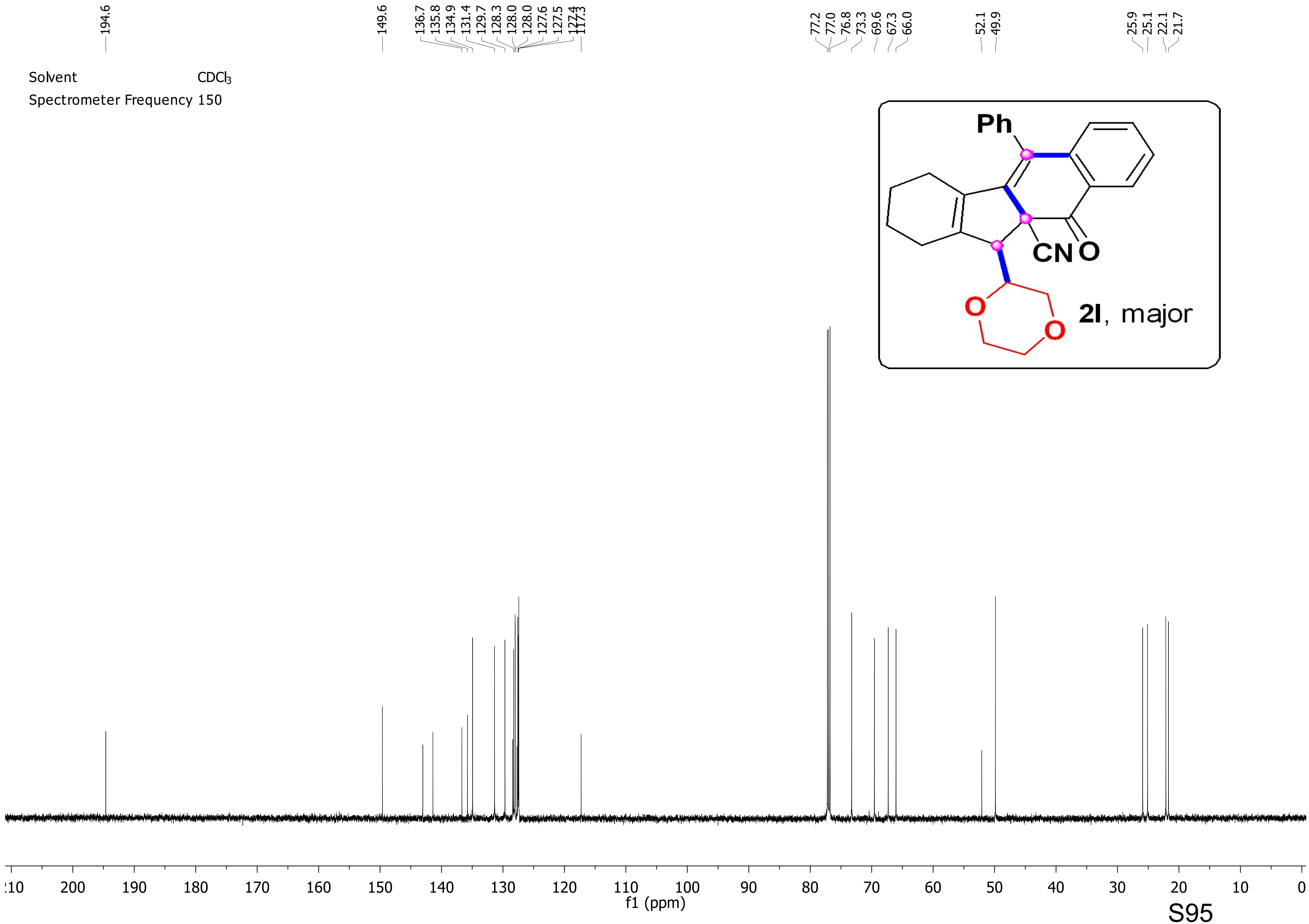
194.6
149.6
136.7
135.8
134.9
131.4
129.7
128.3
128.0
128.0
127.6
127.5
127.4
117.3
77.2
77.0
76.8
73.3
69.6
67.3
66.0
52.1
49.9
25.9
25.1
22.1
21.7
Solvent CDCl3
Spectrometer Frequency 150
Ph
CN
O
O
O
2l, major
f1 (ppm)

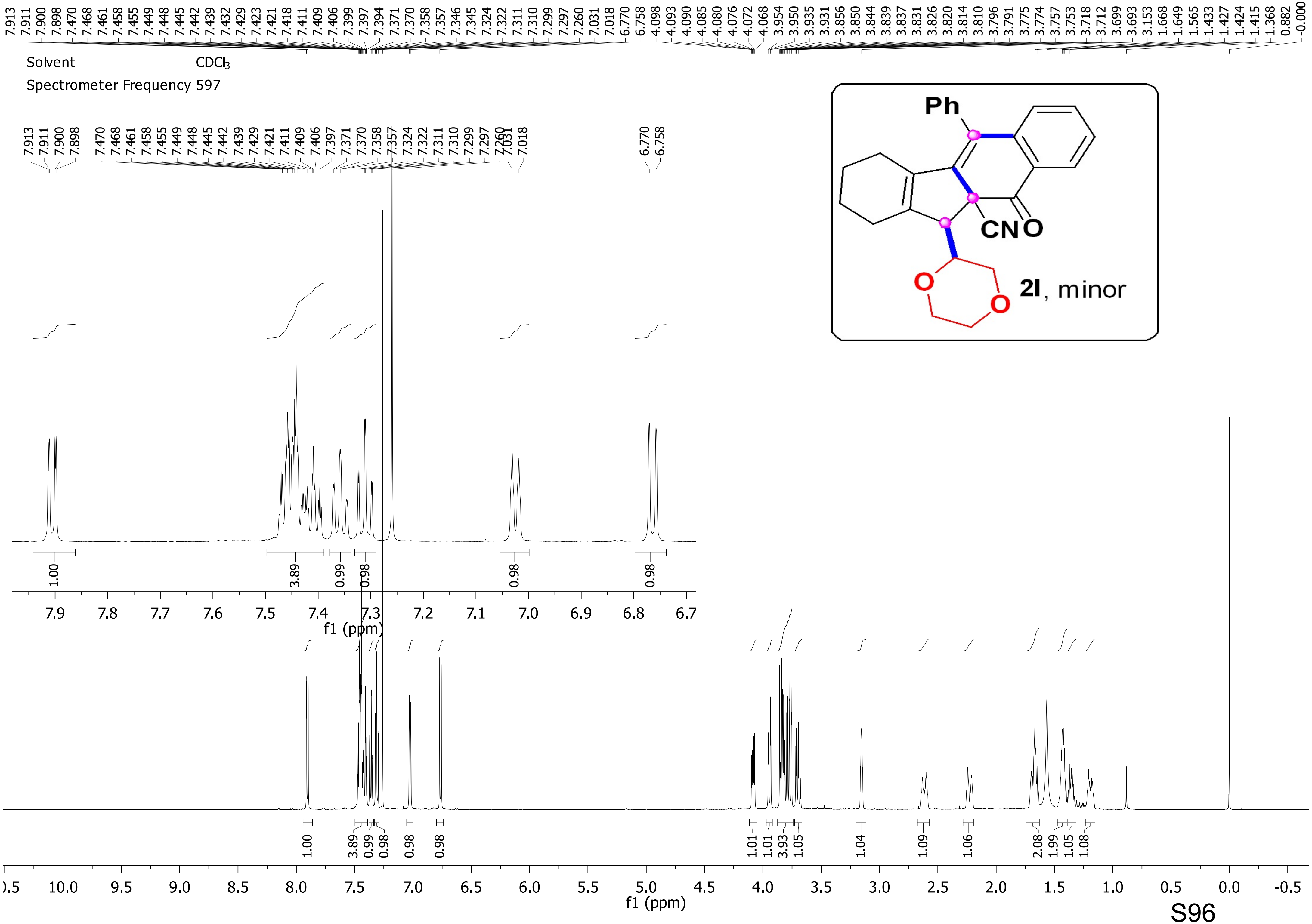
7.913 7.911 7.900 7.898 7.470 7.468 7.461 7.458 7.455 7.449 7.448 7.445 7.442 7.439 7.432 7.429 7.423 7.421 7.418 7.411 7.409 7.406 7.399 7.397 7.394 7.371 7.370 7.358 7.357 7.346 7.345 7.324 7.322 7.311 7.310 7.299 7.297 7.260 7.031 7.018 6.770 6.758 4.098 4.093 4.090 4.085 4.080 4.076 4.072 4.068 3.954 3.950 3.935 3.931 3.856 3.850 3.844 3.839 3.837 3.831 3.826 3.820 3.814 3.810 3.796 3.791 3.775 3.774 3.757 3.753 3.718 3.712 3.699 3.693 3.153 1.668 1.649 1.565 1.433 1.427 1.424 1.415 1.368 0.882 -0.000
Solvent CDCl3
Spectrometer Frequency 597
Ph
CN
O
O
O
2l, minor
1.00
3.89
0.99
0.98
0.98
0.98
f1 (ppm)
1.01
1.01
3.93
1.05
1.04
1.09
1.06
2.08
1.99
1.05
1.08
f1 (ppm)

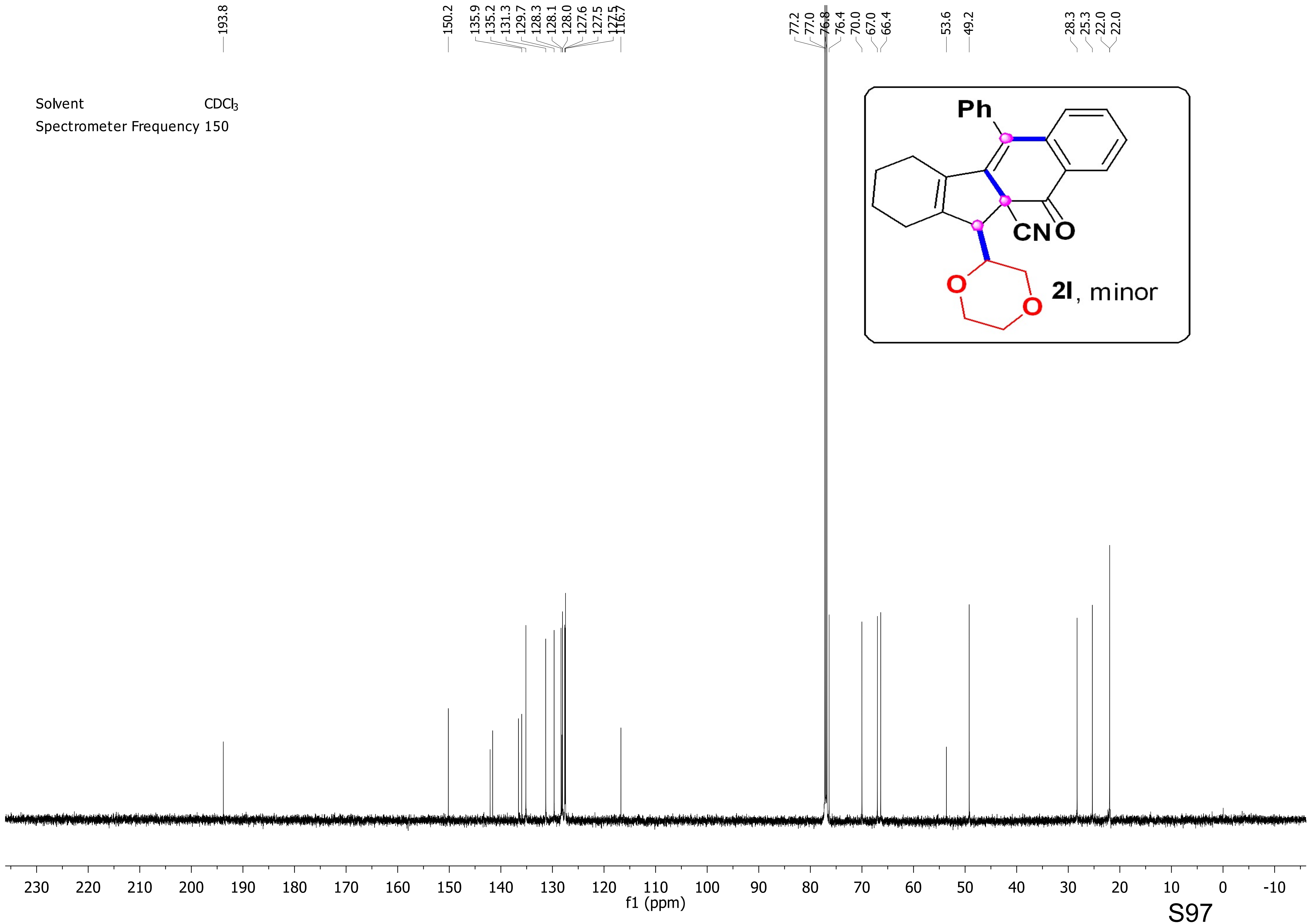
193.8
150.2
135.9
135.2
131.3
129.7
128.3
128.1
128.0
127.6
127.5
127.5
116.7
77.2
77.0
76.8
76.4
70.0
67.0
66.4
53.6
49.2
28.3
25.3
22.0
22.0
Solvent CDCl3
Spectrometer Frequency 150
Ph
CN
O
O
O
2l, minor
230 220 210 200 190 180 170 160 150 140 130 120 110 100 90 80 70 60 50 40 30 20 10 0 -10
f1 (ppm)

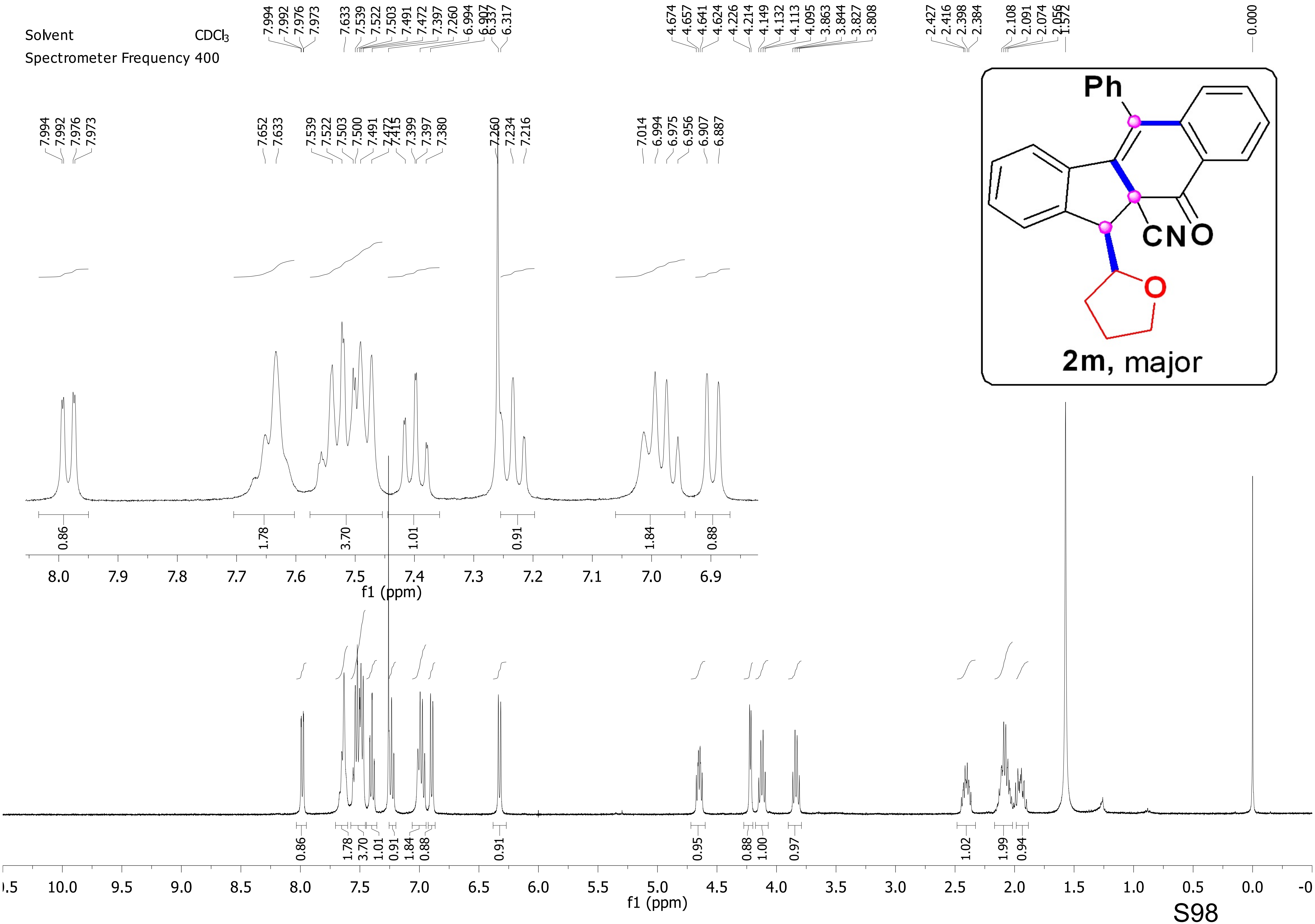
Solvent CDCl3
Spectrometer Frequency 400
7.994 7.992 7.976 7.973 7.633 7.539 7.522 7.503 7.491 7.472 7.397 7.260 6.994 6.907 6.337 6.317
4.674 4.657 4.641 4.624 4.226 4.214 4.149 4.132 4.113 4.095 3.863 3.844 3.827 3.808
2.427 2.416 2.398 2.384 2.108 2.091 2.074 2.056 1.572
0.000
7.994 7.992 7.976 7.973 7.652 7.633 7.539 7.522 7.503 7.500 7.491 7.472 7.415 7.399 7.397 7.380 7.260 7.234 7.216 7.014 6.994 6.975 6.956 6.907 6.887
0.86 1.78 3.70 1.01 0.91 1.84 0.88
0.86 1.78 3.70 1.01 0.91 1.84 0.88 0.91 0.95 0.88 1.00 0.97 1.02 1.99 0.94
f1 (ppm)
f1 (ppm)
Ph
CN
O
O
2m, major

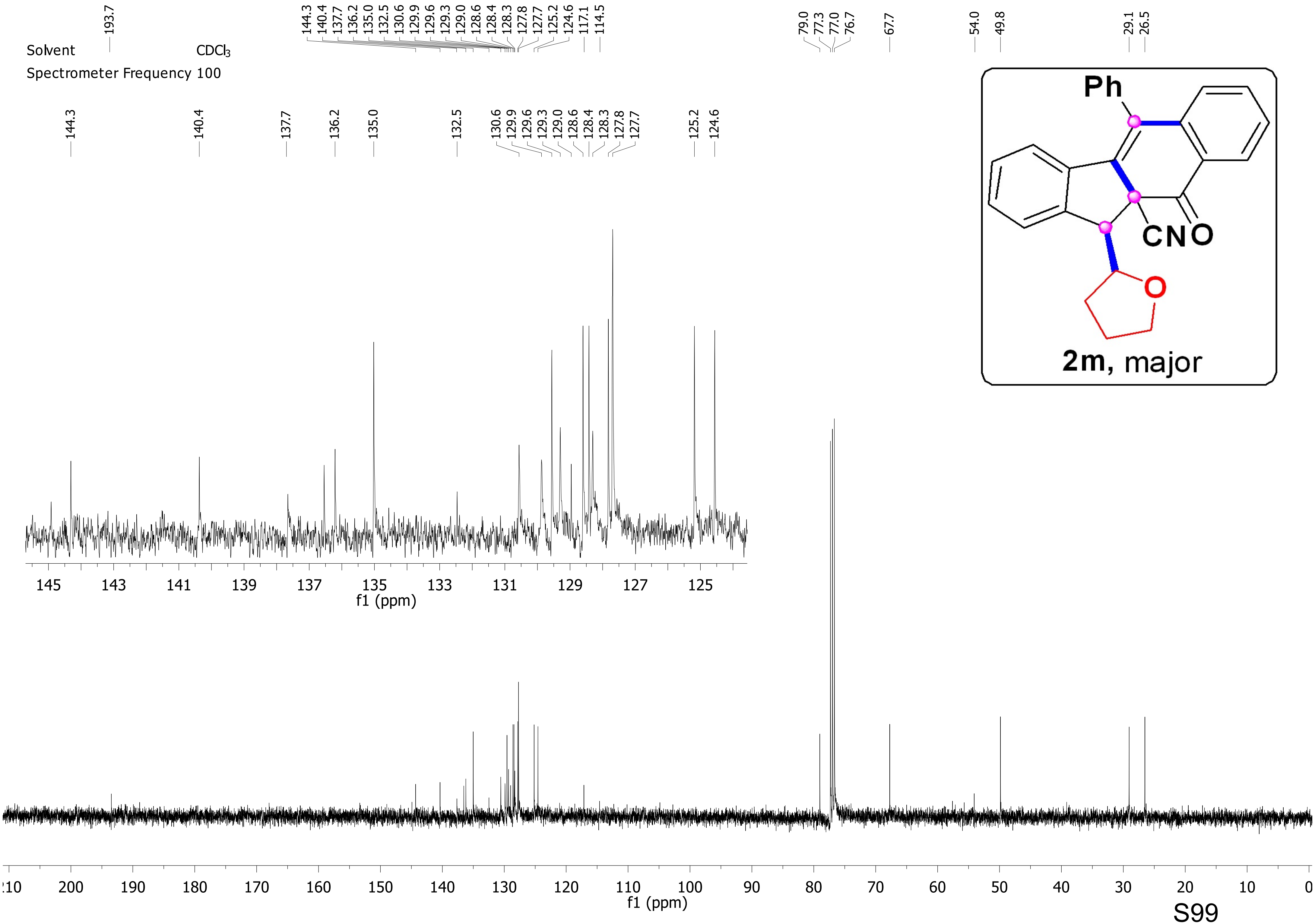

Solvent CDCl$_3$
Spectrometer Frequency 100
193.7
144.3 140.4 137.7 136.2 135.0 132.5 130.6 129.9 129.6 129.3 129.0 128.6 128.4 128.3 127.8 127.7 125.2 124.6 117.1 114.5
79.0 77.3 77.0 76.7
67.7
54.0 49.8
29.1 26.5
Ph
CN
O
O
2m, major
145 143 141 139 137 135 133 131 129 127 125
f1 (ppm)
210 200 190 180 170 160 150 140 130 120 110 100 90 80 70 60 50 40 30 20 10 0
f1 (ppm)

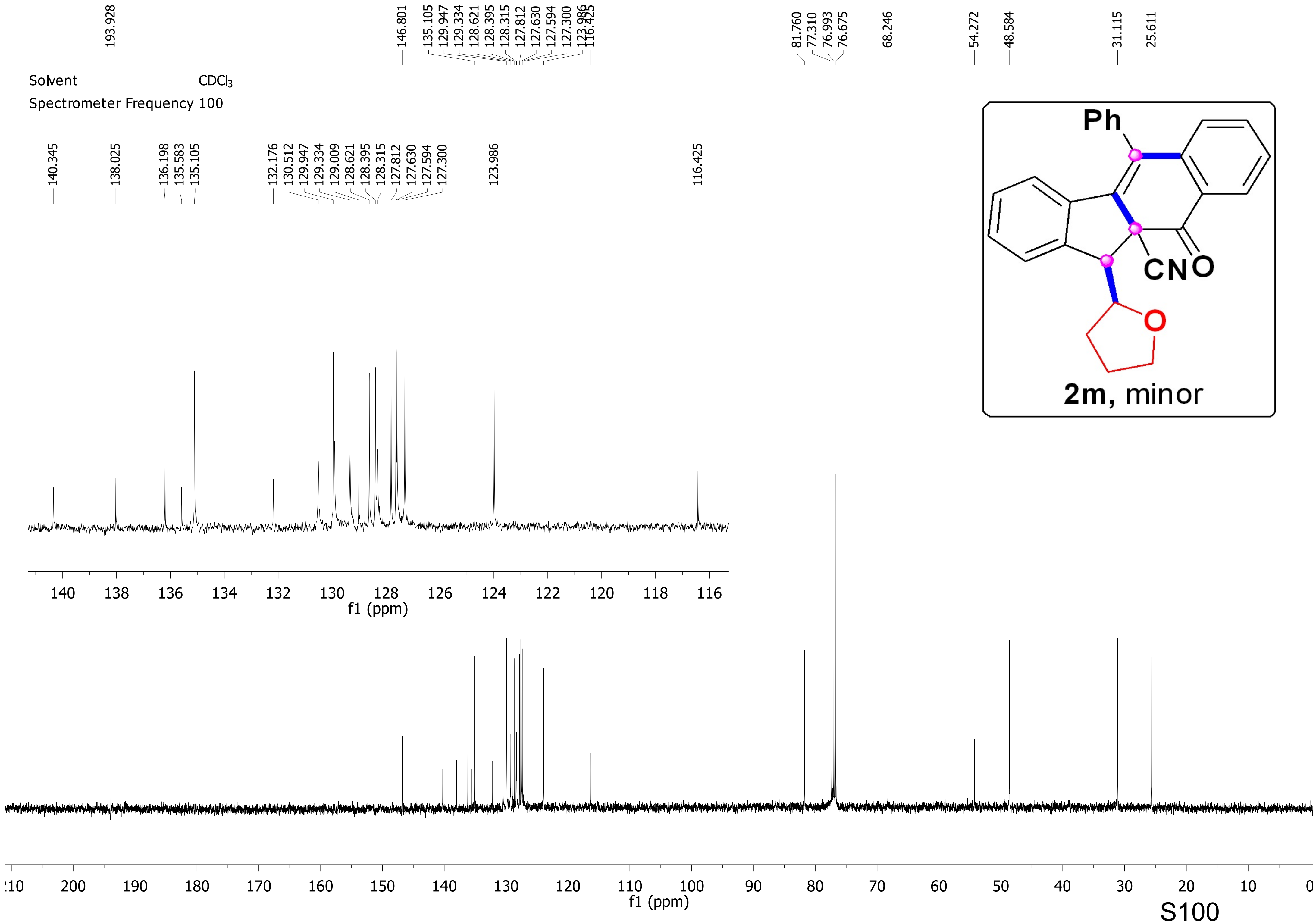
193.928
146.801
135.105
129.947
129.334
128.621
128.395
128.315
127.812
127.630
127.594
127.300
123.986
116.425
81.760
77.310
76.993
76.675
68.246
54.272
48.584
31.115
25.611
Solvent CDCl3
Spectrometer Frequency 100
140.345
138.025
136.198
135.583
135.105
132.176
130.512
129.947
129.334
129.009
128.621
128.395
128.315
127.812
127.630
127.594
127.300
123.986
116.425
Ph
CN
O
O
2m, minor
f1 (ppm)
f1 (ppm)

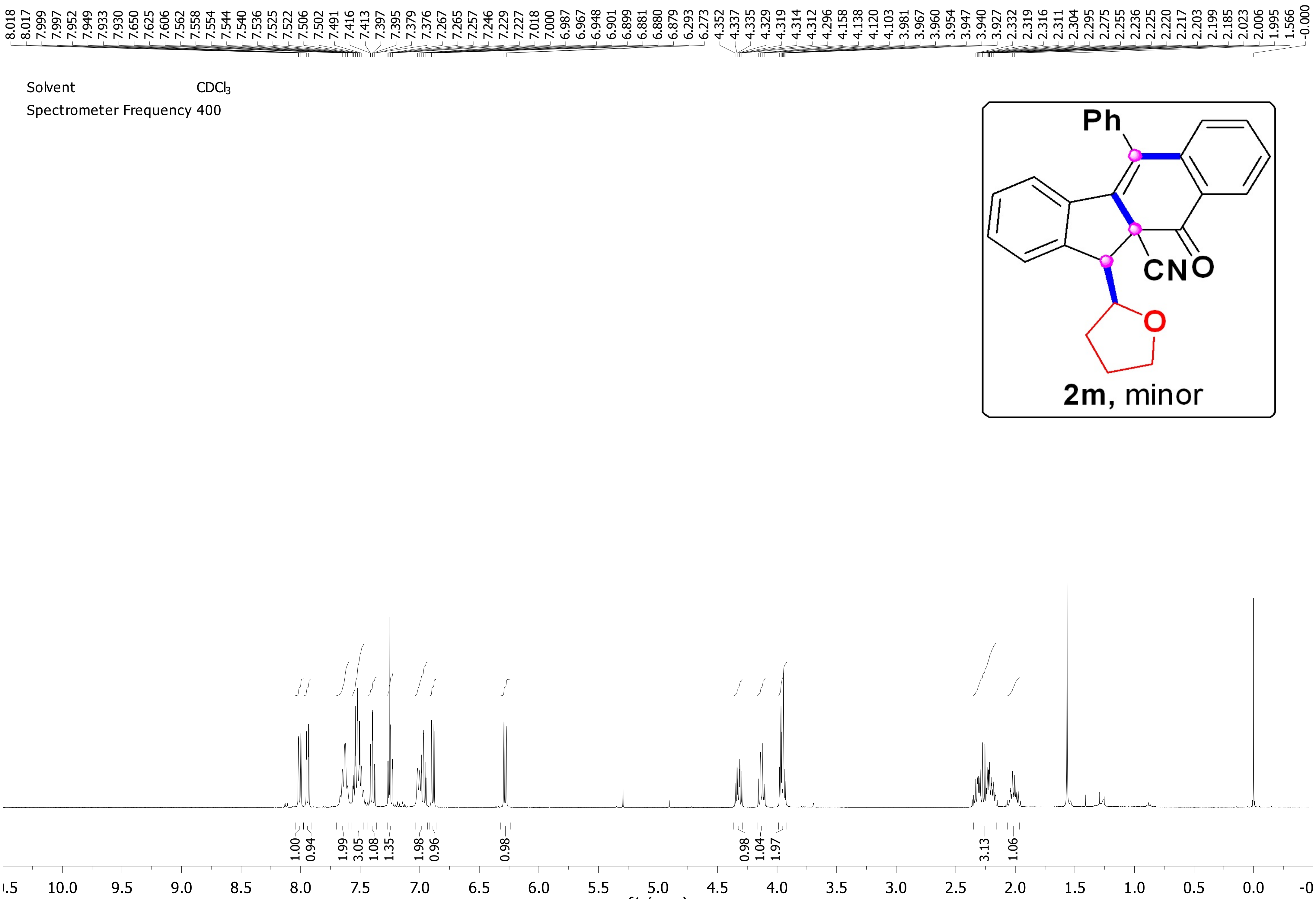
8.018 8.017 7.999 7.997 7.952 7.949 7.933 7.930 7.650 7.625 7.606 7.562 7.558 7.554 7.544 7.540 7.536 7.525 7.522 7.506 7.502 7.491 7.416 7.413 7.397 7.395 7.379 7.376 7.267 7.265 7.257 7.246 7.229 7.227 7.018 7.000 6.987 6.967 6.948 6.901 6.899 6.881 6.880 6.879 6.293 6.273 4.352 4.337 4.335 4.329 4.319 4.314 4.312 4.296 4.158 4.138 4.120 4.103 3.981 3.967 3.960 3.954 3.947 3.940 3.927 2.332 2.319 2.316 2.311 2.304 2.295 2.275 2.255 2.236 2.225 2.220 2.217 2.203 2.199 2.185 2.023 2.006 1.995 1.566 -0.000
Solvent CDCl3
Spectrometer Frequency 400
Ph
CN
O
O
2m, minor
1.00 0.94 1.99 3.05 1.08 1.35 1.98 0.96 0.98 0.98 1.04 1.97 3.13 1.06
10.0 9.5 9.0 8.5 8.0 7.5 7.0 6.5 6.0 5.5 5.0 4.5 4.0 3.5 3.0 2.5 2.0 1.5 1.0 0.5 0.0
f1 (ppm)

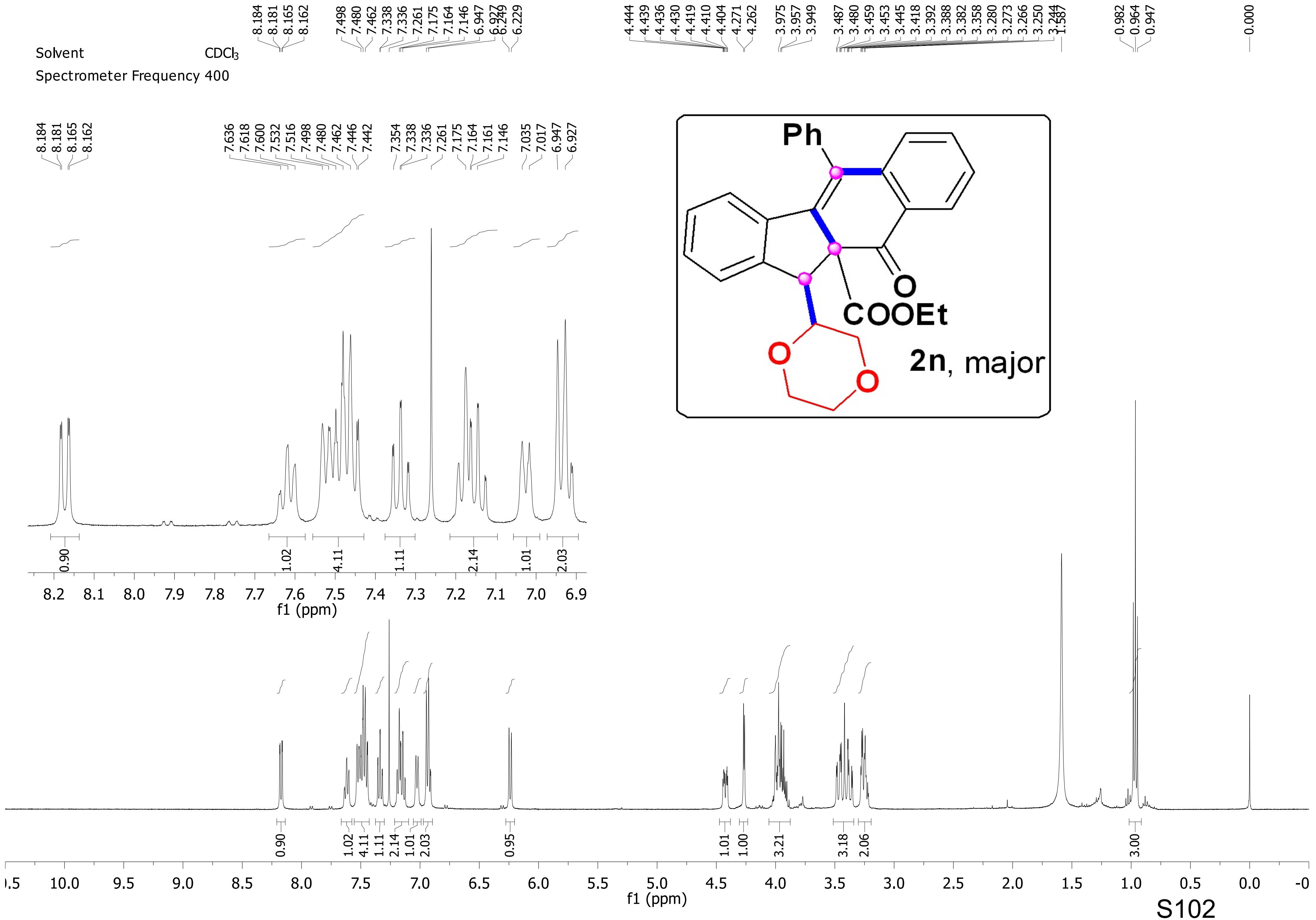
Solvent CDCl3
Spectrometer Frequency 400
8.184 8.181 8.165 8.162 7.498 7.480 7.462 7.338 7.336 7.261 7.175 7.164 7.146 6.947 6.927 6.249 6.229
4.444 4.439 4.436 4.430 4.419 4.410 4.404 4.271 4.262 3.975 3.957 3.949 3.487 3.480 3.459 3.453 3.445 3.418 3.392 3.388 3.382 3.358 3.280 3.273 3.266 3.250 3.244 1.587
0.982 0.964 0.947 0.000
8.184 8.181 8.165 8.162 7.636 7.618 7.600 7.532 7.516 7.498 7.480 7.462 7.446 7.442 7.354 7.338 7.336 7.261 7.175 7.164 7.161 7.146 7.035 7.017 6.947 6.927
0.90 1.02 4.11 1.11 2.14 1.01 2.03
8.2 8.1 8.0 7.9 7.8 7.7 7.6 7.5 7.4 7.3 7.2 7.1 7.0 6.9
f1 (ppm)
Ph
O
COOEt
O
O
2n, major
0.90 1.02 4.11 1.11 2.14 1.01 2.03 0.95 1.01 1.00 3.21 3.18 2.06 3.00
10.0 9.5 9.0 8.5 8.0 7.5 7.0 6.5 6.0 5.5 5.0 4.5 4.0 3.5 3.0 2.5 2.0 1.5 1.0 0.5 0.0
f1 (ppm)

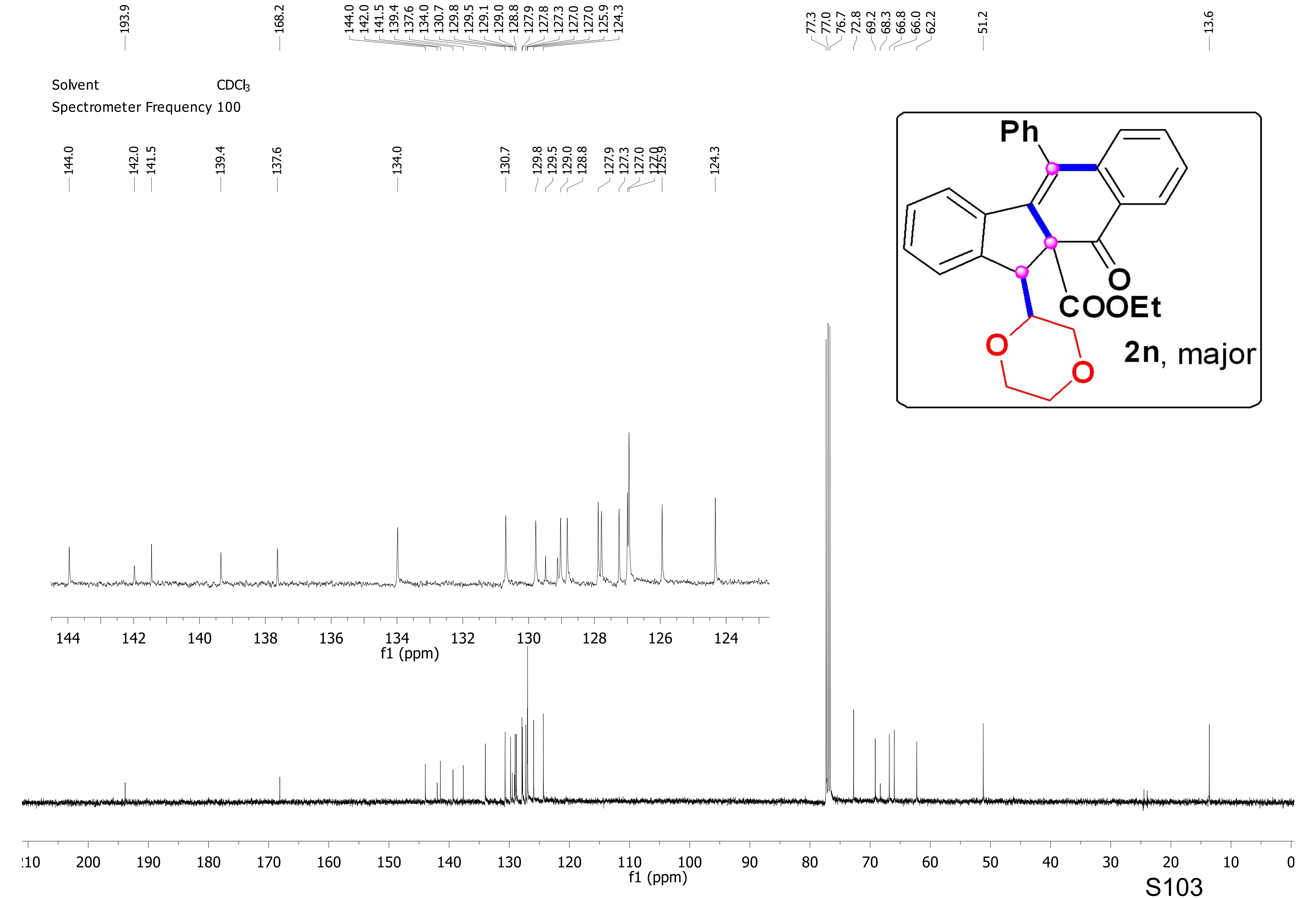
193.9
168.2
144.0 142.0 141.5 139.4 137.6 134.0 130.7 129.8 129.5 129.1 129.0 128.8 127.9 127.8 127.3 127.0 127.0 125.9 124.3
77.3 77.0 76.7 72.8 69.2 68.3 66.8 66.0 62.2
51.2
13.6
Solvent CDCl3
Spectrometer Frequency 100
144.0 142.0 141.5 139.4 137.6 134.0 130.7 129.8 129.5 129.0 128.8 127.9 127.3 127.0 127.0 125.9 124.3
Ph
O
COOEt
O
O
2n, major
144 142 140 138 136 134 132 130 128 126 124
f1 (ppm)
210 200 190 180 170 160 150 140 130 120 110 100 90 80 70 60 50 40 30 20 10 0
f1 (ppm)

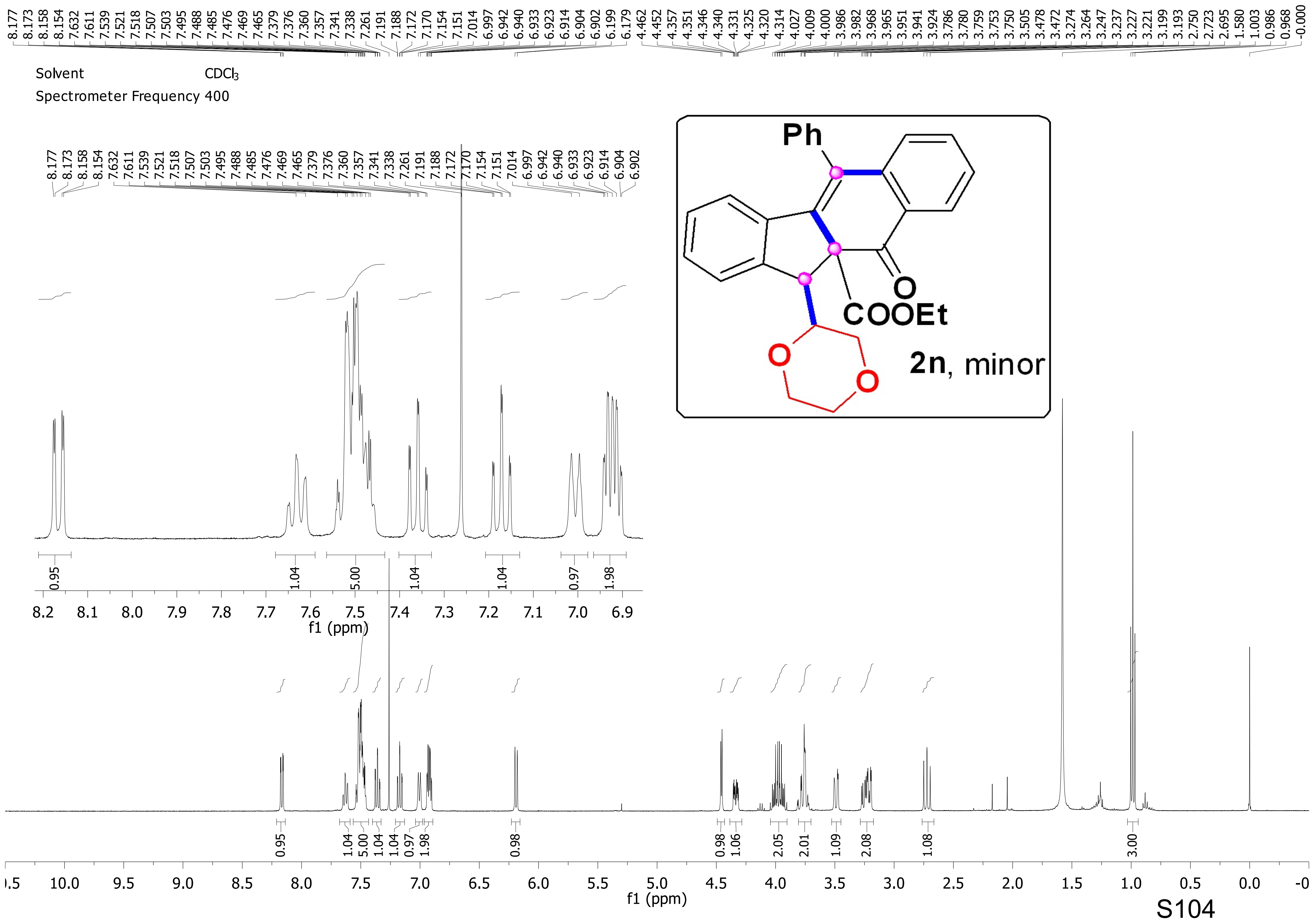
8.177 8.173 8.158 8.154 7.632 7.611 7.539 7.521 7.518 7.507 7.503 7.495 7.488 7.485 7.476 7.469 7.465 7.379 7.376 7.360 7.357 7.341 7.338 7.261 7.191 7.188 7.172 7.170 7.154 7.151 7.014 6.997 6.942 6.940 6.933 6.923 6.914 6.904 6.902 6.199 6.179 4.462 4.452 4.357 4.351 4.346 4.340 4.331 4.325 4.320 4.314 4.027 4.009 4.000 3.986 3.982 3.968 3.965 3.951 3.941 3.924 3.786 3.780 3.759 3.753 3.750 3.505 3.478 3.472 3.274 3.264 3.247 3.237 3.227 3.221 3.199 3.193 2.750 2.723 2.695 1.580 1.003 0.986 0.968 -0.000
Solvent CDCl3
Spectrometer Frequency 400
Ph
O
COOEt
O
O
2n, minor
0.95 1.04 5.00 1.04 1.04 0.97 1.98
f1 (ppm)
0.95 1.04 5.00 1.04 1.04 0.97 1.98 0.98 0.98 1.06 2.05 2.01 1.09 2.08 1.08 3.00
f1 (ppm)

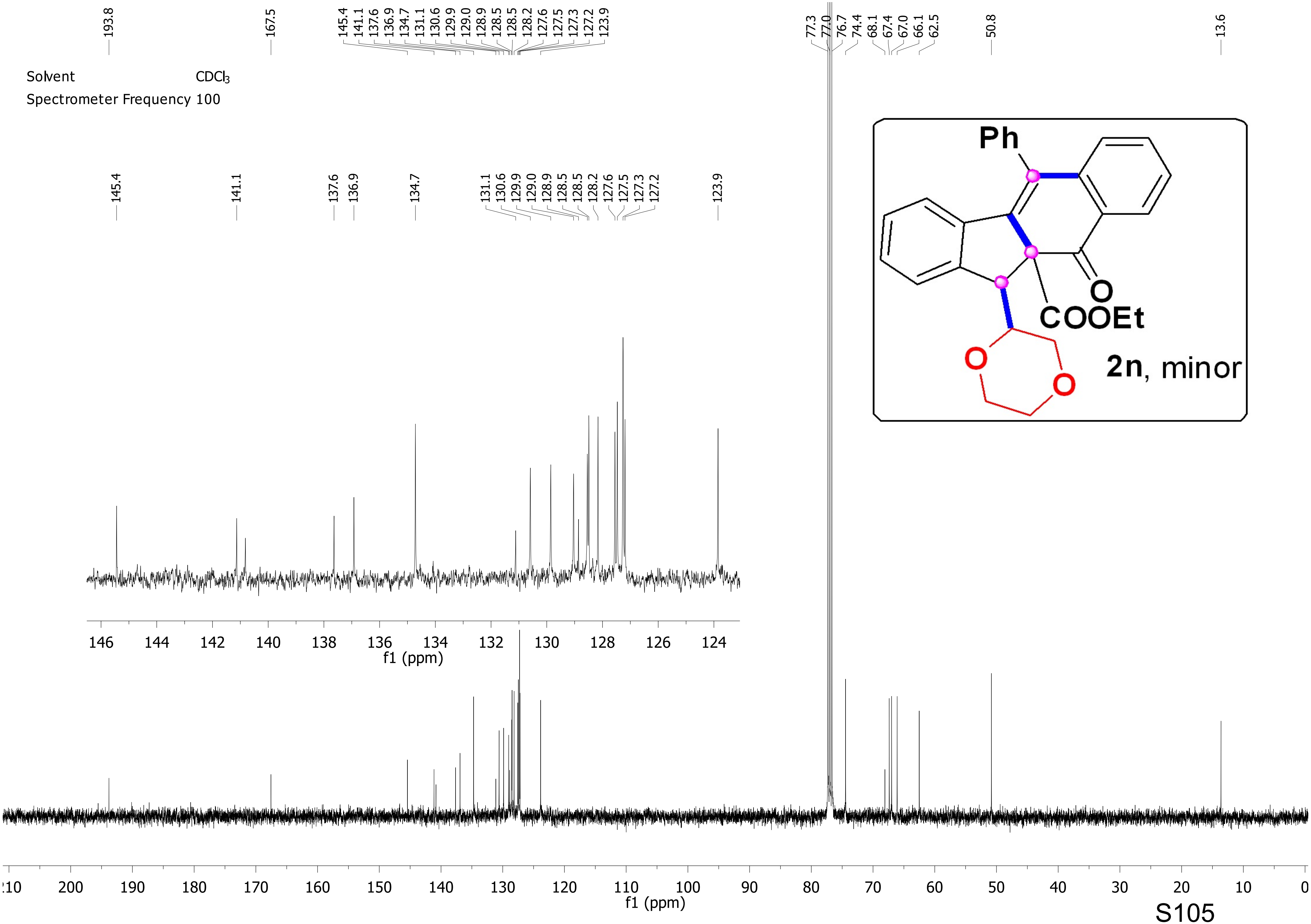

193.8
167.5
145.4
141.1
137.6
136.9
134.7
131.1
130.6
129.9
129.0
128.9
128.5
128.5
128.2
127.6
127.5
127.3
127.2
123.9
77.3
77.0
76.7
74.4
68.1
67.4
67.0
66.1
62.5
50.8
13.6
Solvent CDCl3
Spectrometer Frequency 100
Ph
O
COOEt
O
O
2n, minor
f1 (ppm)
f1 (ppm)

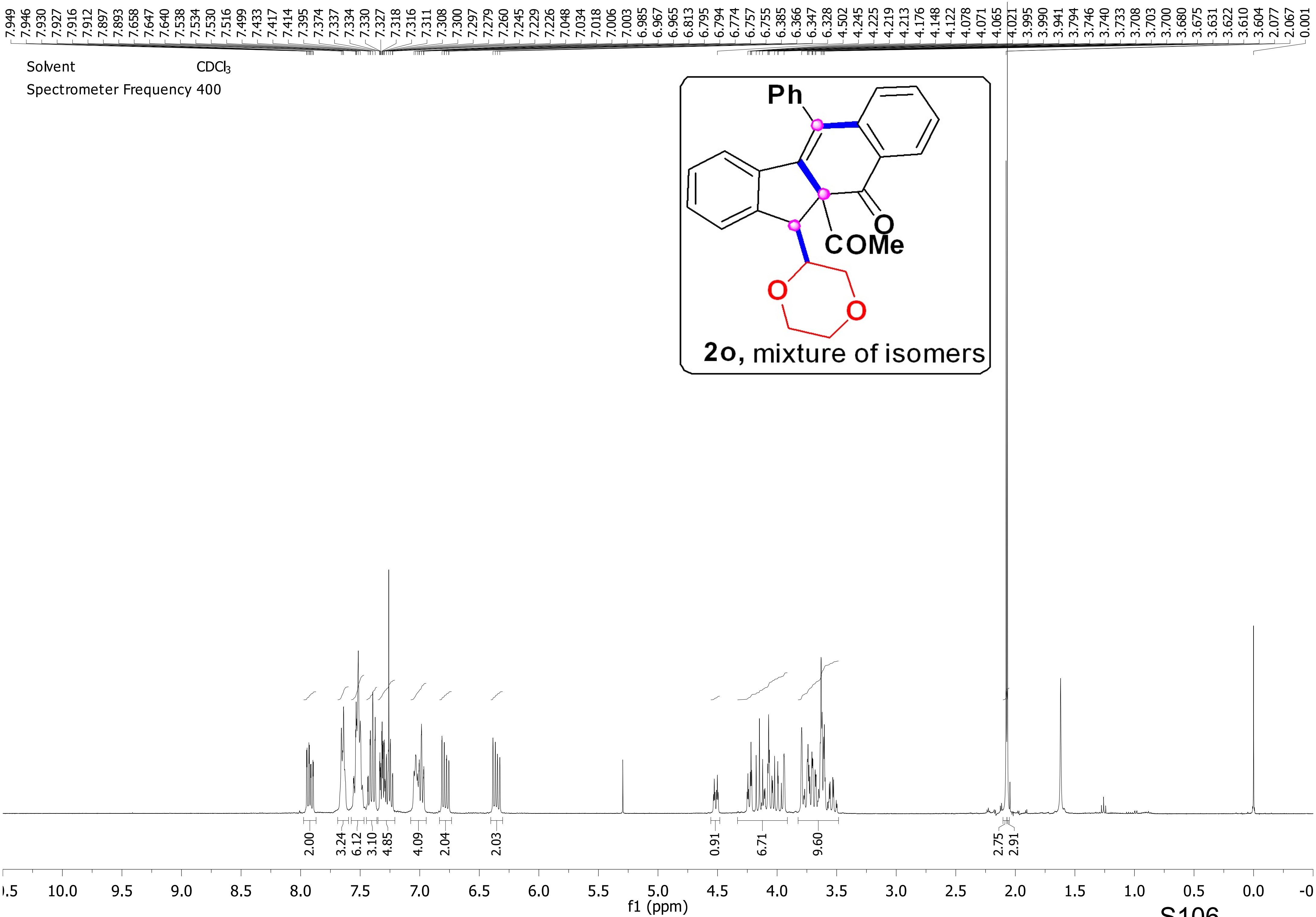
7.949 7.946 7.930 7.927 7.916 7.912 7.897 7.893 7.658 7.647 7.640 7.538 7.534 7.530 7.516 7.499 7.433 7.417 7.414 7.395 7.374 7.337 7.334 7.330 7.327 7.318 7.316 7.311 7.308 7.300 7.297 7.279 7.260 7.245 7.229 7.226 7.048 7.034 7.018 7.006 7.003 6.985 6.967 6.965 6.813 6.795 6.794 6.774 6.757 6.755 6.385 6.366 6.347 6.328 4.502 4.245 4.225 4.219 4.213 4.176 4.148 4.122 4.078 4.071 4.065 4.021 3.995 3.990 3.941 3.794 3.746 3.740 3.733 3.708 3.703 3.700 3.680 3.675 3.631 3.622 3.610 3.604 2.077 2.067 0.001
Solvent CDCl3
Spectrometer Frequency 400
Ph
O
COMe
O
O
2o, mixture of isomers
2.00 3.24 6.12 3.10 4.85 4.09 2.04 2.03 0.91 6.71 9.60 2.75 2.91
10.0 9.5 9.0 8.5 8.0 7.5 7.0 6.5 6.0 5.5 5.0 4.5 4.0 3.5 3.0 2.5 2.0 1.5 1.0 0.5 0.0
f1 (ppm)

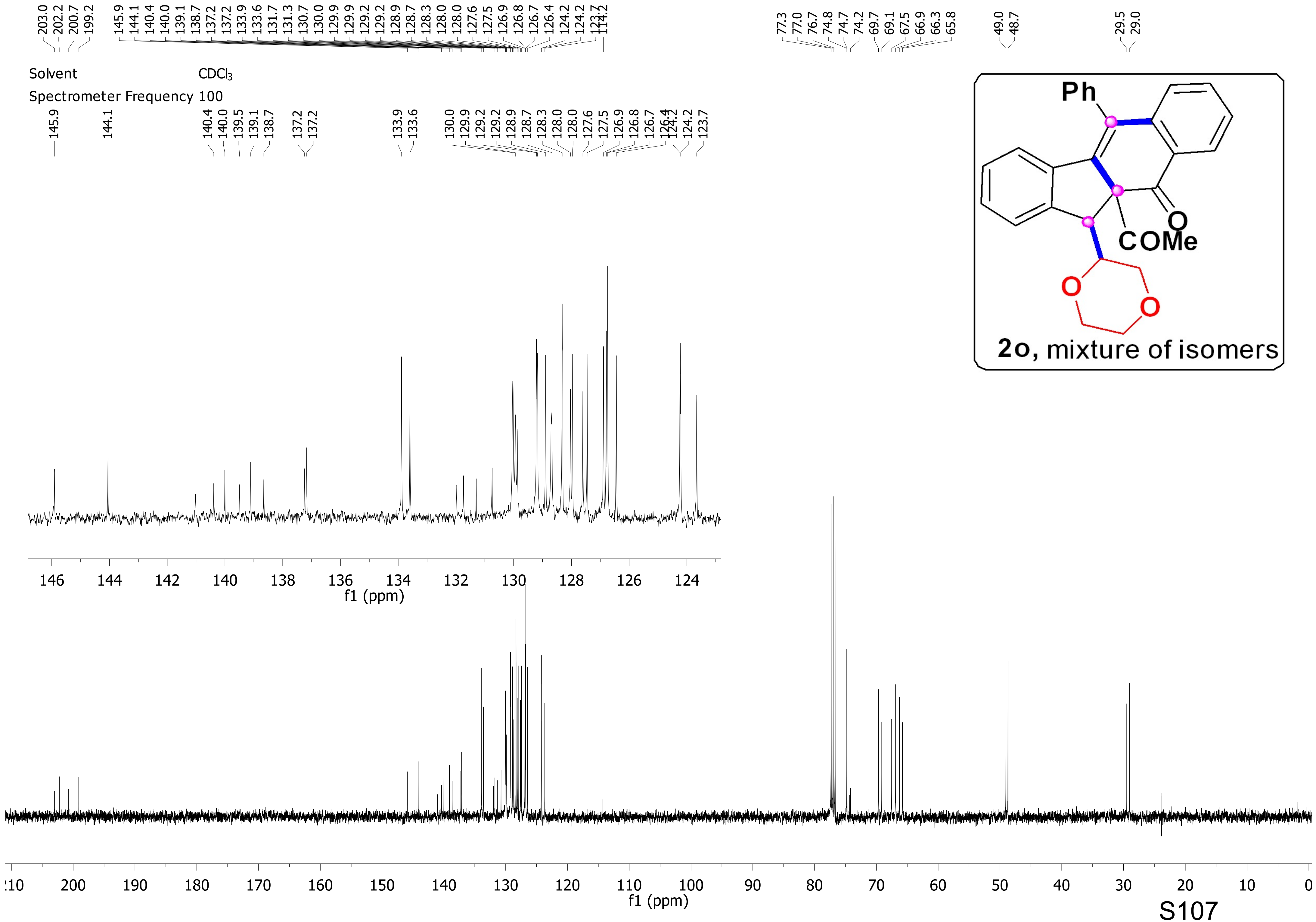
203.0 202.2 200.7 199.2 145.9 144.1 140.4 140.0 139.1 138.7 137.2 137.2 133.9 133.6 131.7 131.3 130.7 130.0 129.9 129.9 129.2 129.2 128.9 128.7 128.3 128.0 128.0 127.6 127.5 126.9 126.8 126.7 126.4 124.2 124.2 123.7 114.2
77.3 77.0 76.7 74.8 74.7 74.2 69.7 69.1 67.5 66.9 66.3 65.8
49.0 48.7
29.5 29.0
Solvent CDCl3
Spectrometer Frequency 100
145.9 144.1 140.4 140.0 139.5 139.1 138.7 137.2 137.2 133.9 133.6 130.0 129.9 129.2 129.2 128.9 128.7 128.3 128.0 128.0 127.6 127.5 126.9 126.8 126.7 126.4 124.2 124.2 123.7
f1 (ppm)
Ph
O
COMe
O
O
2o, mixture of isomers
f1 (ppm)

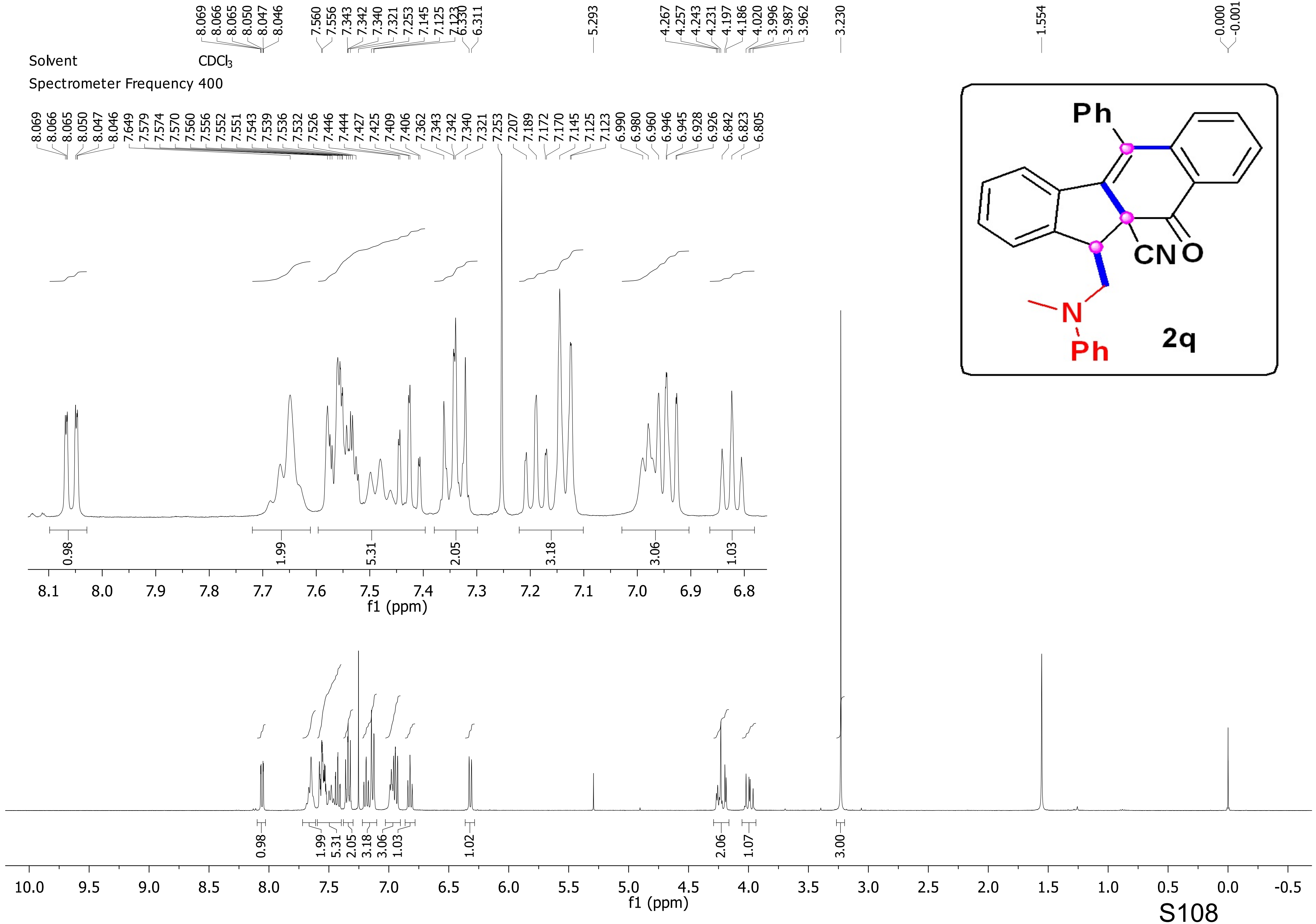
8.069 8.066 8.065 8.050 8.047 8.046 7.560 7.556 7.343 7.342 7.340 7.321 7.253 7.145 7.125 7.123 6.330 6.311 5.293 4.267 4.257 4.243 4.231 4.197 4.186 4.020 3.996 3.987 3.962 3.230 1.554 0.000 -0.001
Solvent CDCl3
Spectrometer Frequency 400
8.069 8.066 8.065 8.050 8.047 8.046 7.649 7.579 7.574 7.570 7.560 7.556 7.552 7.551 7.543 7.539 7.536 7.532 7.526 7.446 7.444 7.427 7.425 7.409 7.406 7.362 7.343 7.342 7.340 7.321 7.253 7.207 7.189 7.172 7.170 7.145 7.125 7.123 6.990 6.980 6.960 6.946 6.945 6.928 6.926 6.842 6.823 6.805
Ph
CN
O
N
Ph
2q
0.98 1.99 5.31 2.05 3.18 3.06 1.03
f1 (ppm)
0.98 1.99 5.31 2.05 3.18 3.06 1.03 1.02 2.06 1.07 3.00
f1 (ppm)

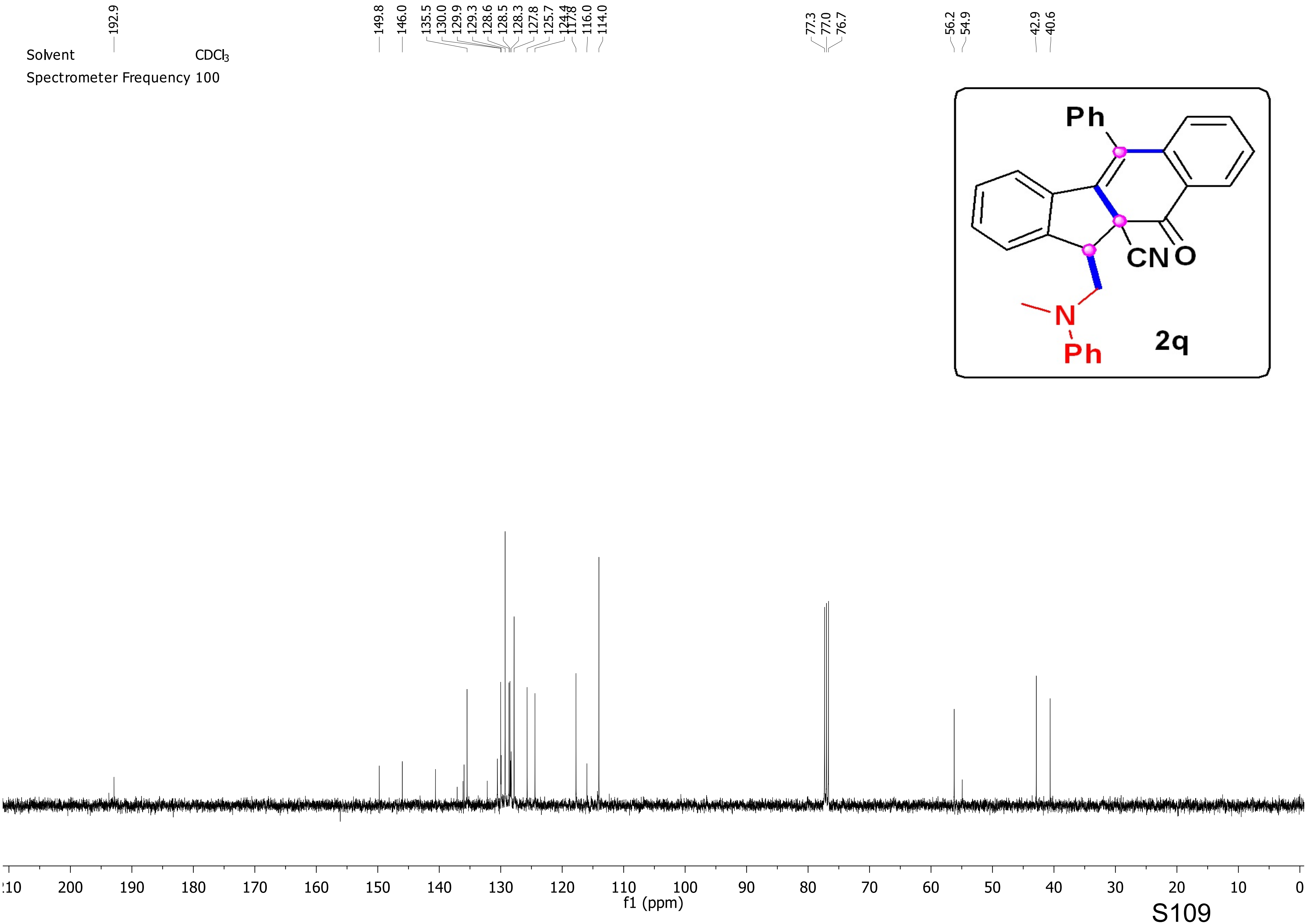
Solvent CDCl3
Spectrometer Frequency 100
192.9
149.8
146.0
135.5
130.0
129.9
129.3
128.6
128.5
128.3
127.8
125.7
124.4
117.8
116.0
114.0
77.3
77.0
76.7
56.2
54.9
42.9
40.6
210 200 190 180 170 160 150 140 130 120 110 100 90 80 70 60 50 40 30 20 10 0
f1 (ppm)
Ph
CN
O
N
Ph

2q

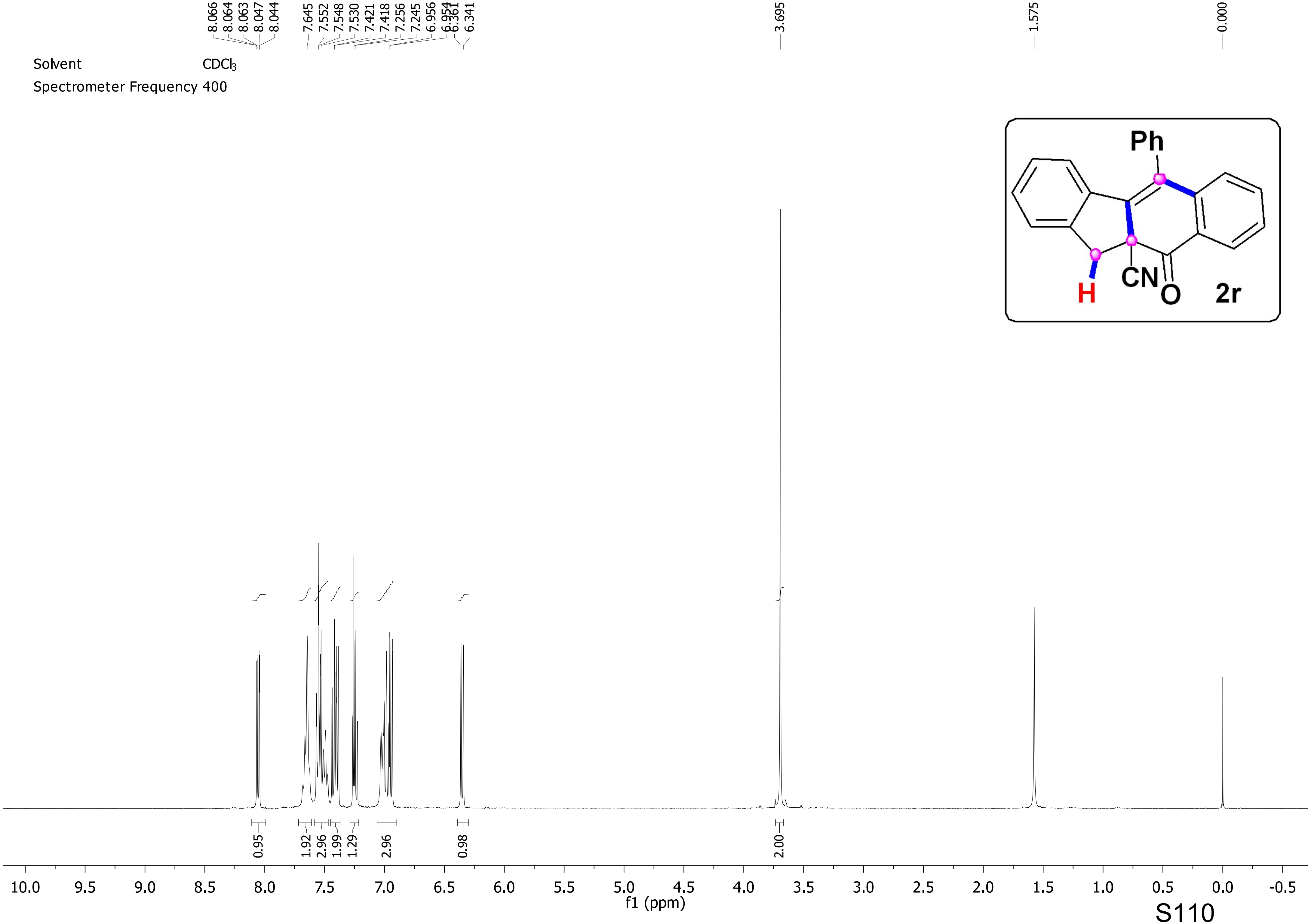
Solvent CDCl3
Spectrometer Frequency 400
8.066
8.064
8.063
8.047
8.044
7.645
7.552
7.548
7.530
7.421
7.418
7.256
7.245
6.956
6.954
6.361
6.341
3.695
1.575
0.000
Ph
CN
O
H
2r
0.95
1.92
2.96
1.99
1.29
2.96
0.98
2.00
f1 (ppm)

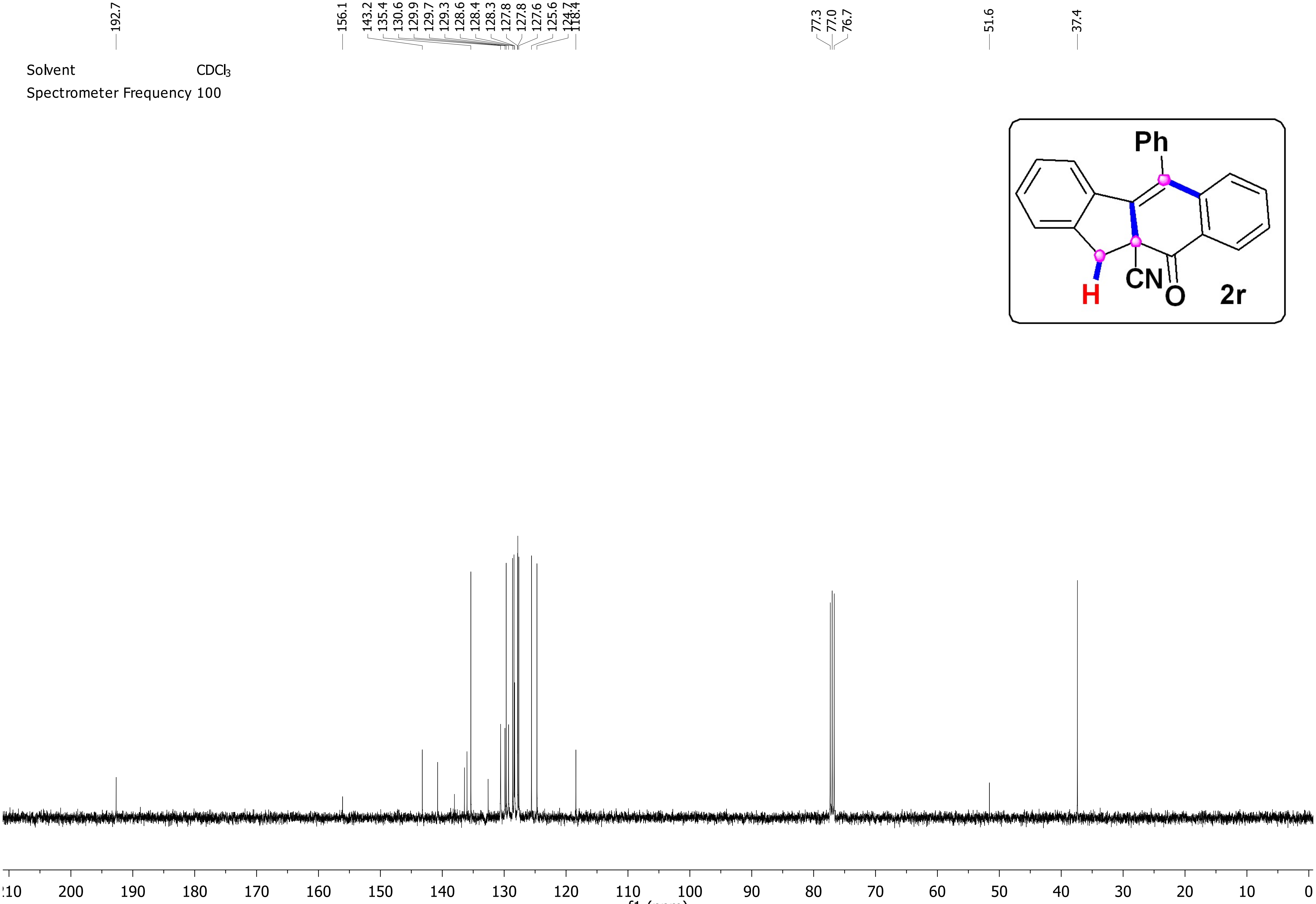
192.7
156.1
143.2
135.4
130.6
129.9
129.7
129.3
128.6
128.4
128.3
127.8
127.8
127.6
125.6
124.7
118.4
77.3
77.0
76.7
51.6
37.4
Solvent CDCl3
Spectrometer Frequency 100
Ph
H
CN
O
2r
210
200
190
180
170
160
150
140
130
120
110
100
90
80
70
60
50
40
30
20
10
0
f1 (ppm)

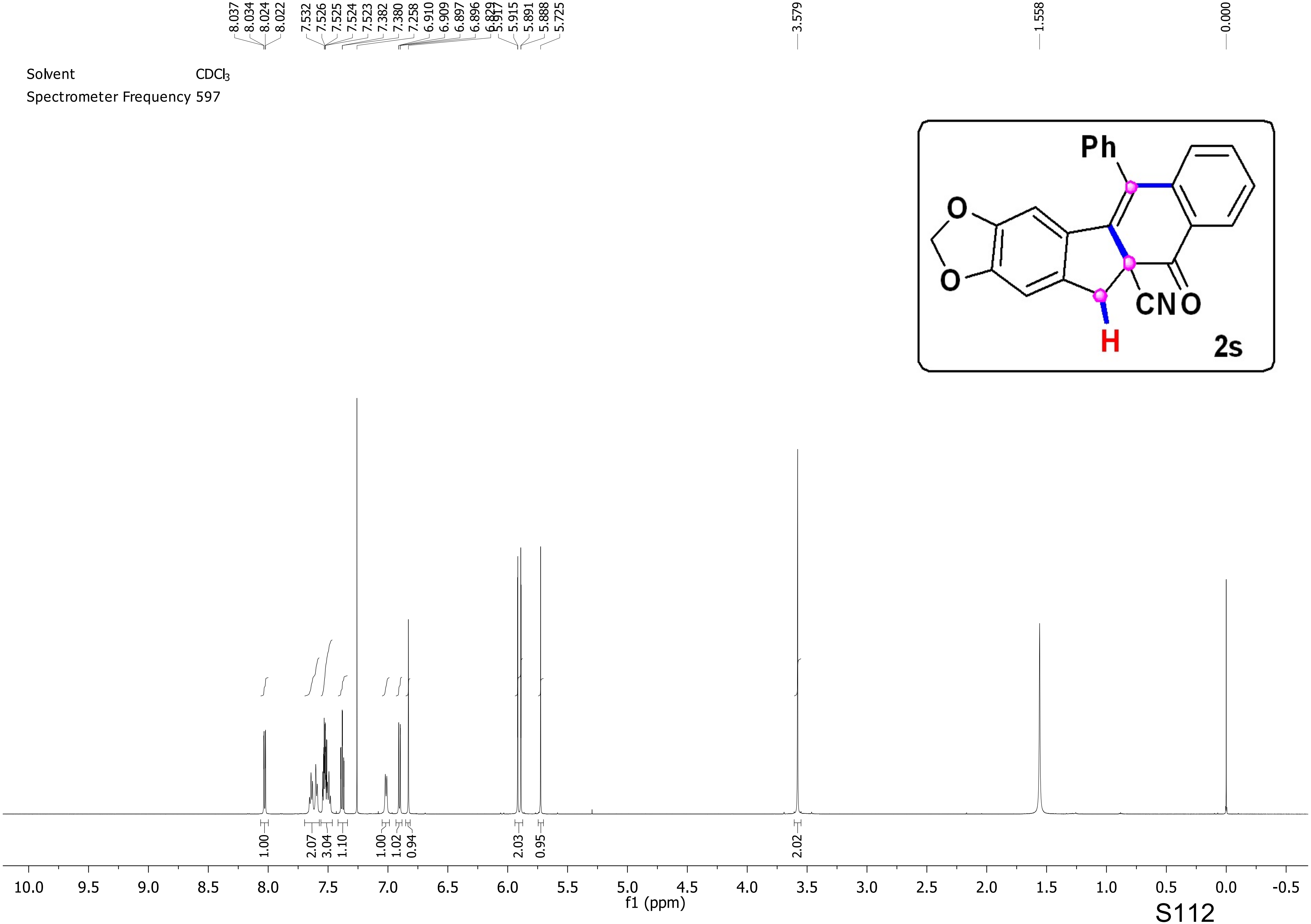
8.037
8.034
8.024
8.022
7.532
7.526
7.525
7.524
7.523
7.382
7.380
7.258
6.910
6.909
6.897
6.896
6.829
5.917
5.915
5.891
5.888
5.725
3.579
1.558
0.000
Solvent CDCl3
Spectrometer Frequency 597
Ph
O
O
CN
O
H
2s
1.00
2.07
3.04
1.10
1.00
1.02
0.94
2.03
0.95
2.02
10.0
9.5
9.0
8.5
8.0
7.5
7.0
6.5
6.0
5.5
5.0
4.5
4.0
3.5
3.0
2.5
2.0
1.5
1.0
0.5
0.0
-0.5
f1 (ppm)

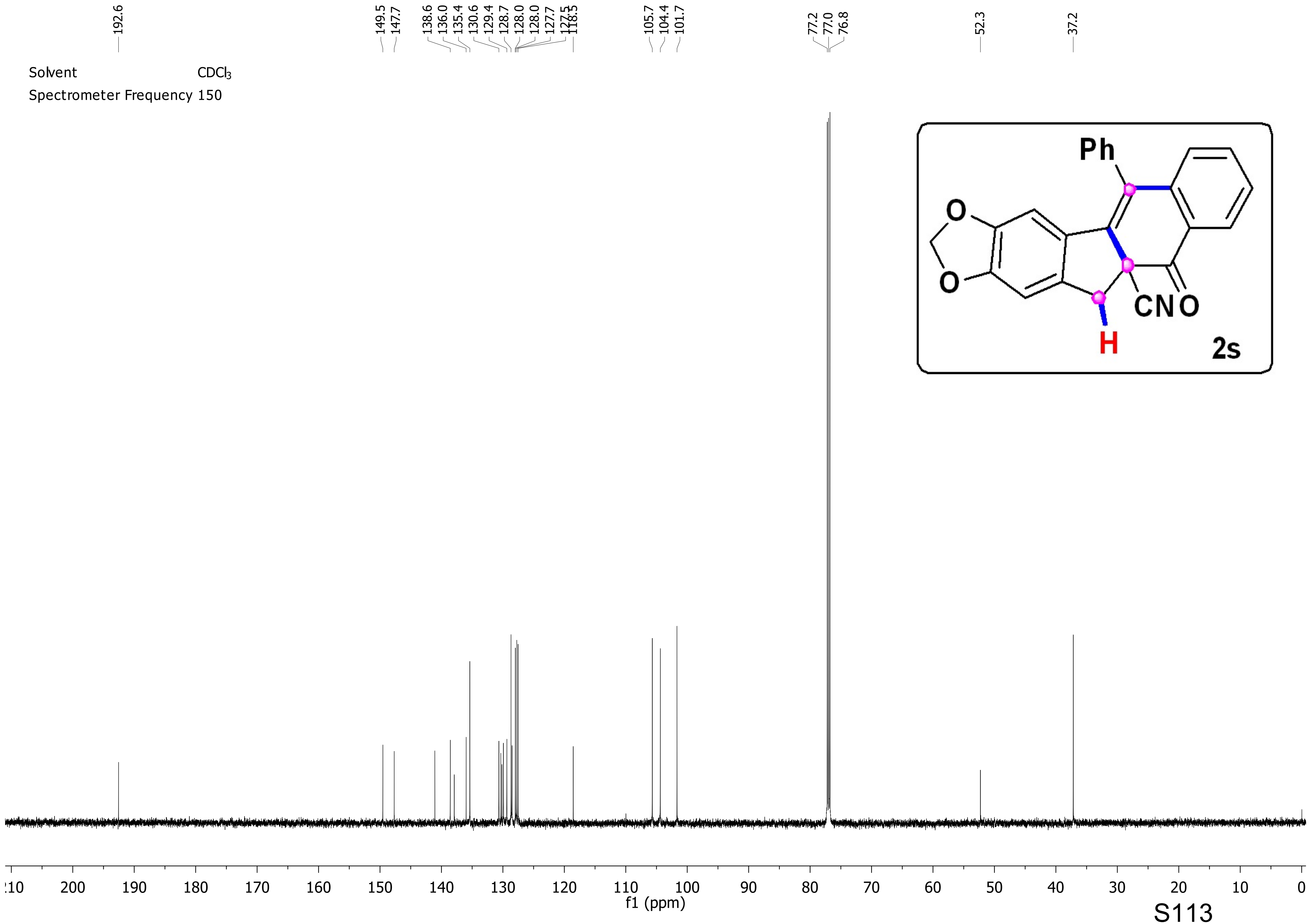
192.6
149.5
147.7
138.6
136.0
135.4
130.6
129.4
128.7
128.0
128.0
127.7
127.5
118.5
105.7
104.4
101.7
77.2
77.0
76.8
52.3
37.2
Solvent CDCl3
Spectrometer Frequency 150
Ph
O
O
CN
O
H
2s
210
200
190
180
170
160
150
140
130
120
110
100
90
80
70
60
50
40
30
20
10
0
f1 (ppm)

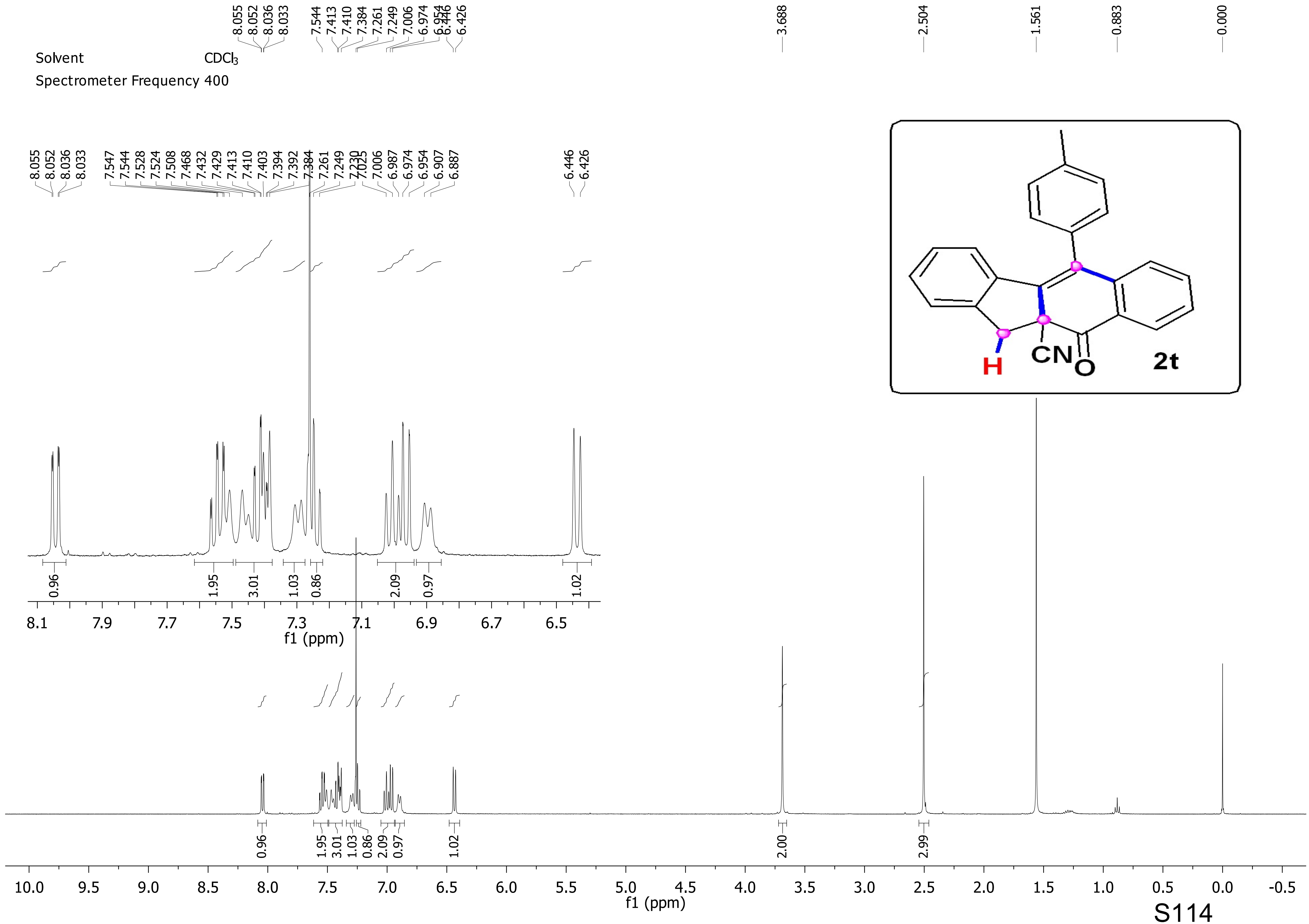
Solvent CDCl3
Spectrometer Frequency 400
8.055 8.052 8.036 8.033 7.544 7.413 7.410 7.384 7.261 7.249 7.006 6.974 6.954 6.446 6.426 3.688 2.504 1.561 0.883 0.000
8.055 8.052 8.036 8.033 7.547 7.544 7.528 7.524 7.508 7.468 7.432 7.429 7.413 7.410 7.403 7.394 7.392 7.384 7.261 7.249 7.230 7.025 7.006 6.987 6.974 6.954 6.907 6.887 6.446 6.426
H
CN
O
2t
0.96 1.95 3.01 1.03 0.86 2.09 0.97 1.02
f1 (ppm)
0.96 1.95 3.01 1.03 0.86 2.09 0.97 1.02 2.00 2.99
f1 (ppm)

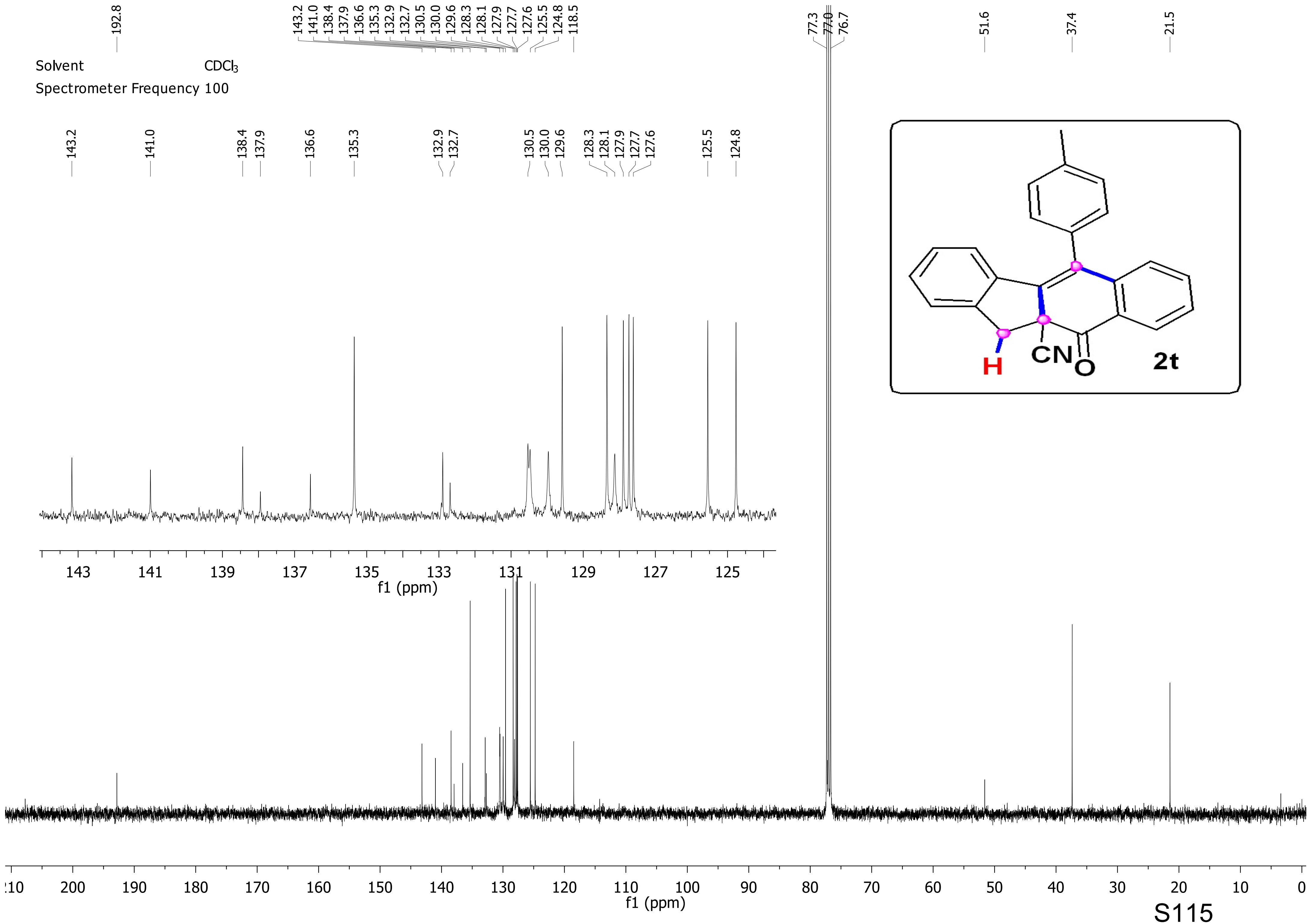
192.8
143.2 141.0 138.4 137.9 136.6 135.3 132.9 132.7 130.5 130.0 129.6 128.3 128.1 127.9 127.7 127.6 125.5 124.8 118.5
77.3 77.0 76.7
51.6
37.4
21.5
Solvent CDCl3
Spectrometer Frequency 100
143.2 141.0 138.4 137.9 136.6 135.3 132.9 132.7 130.5 130.0 129.6 128.3 128.1 127.9 127.7 127.6 125.5 124.8
143 141 139 137 135 133 131 129 127 125
f1 (ppm)
H
CN
O
2t
210 200 190 180 170 160 150 140 130 120 110 100 90 80 70 60 50 40 30 20 10 0
f1 (ppm)

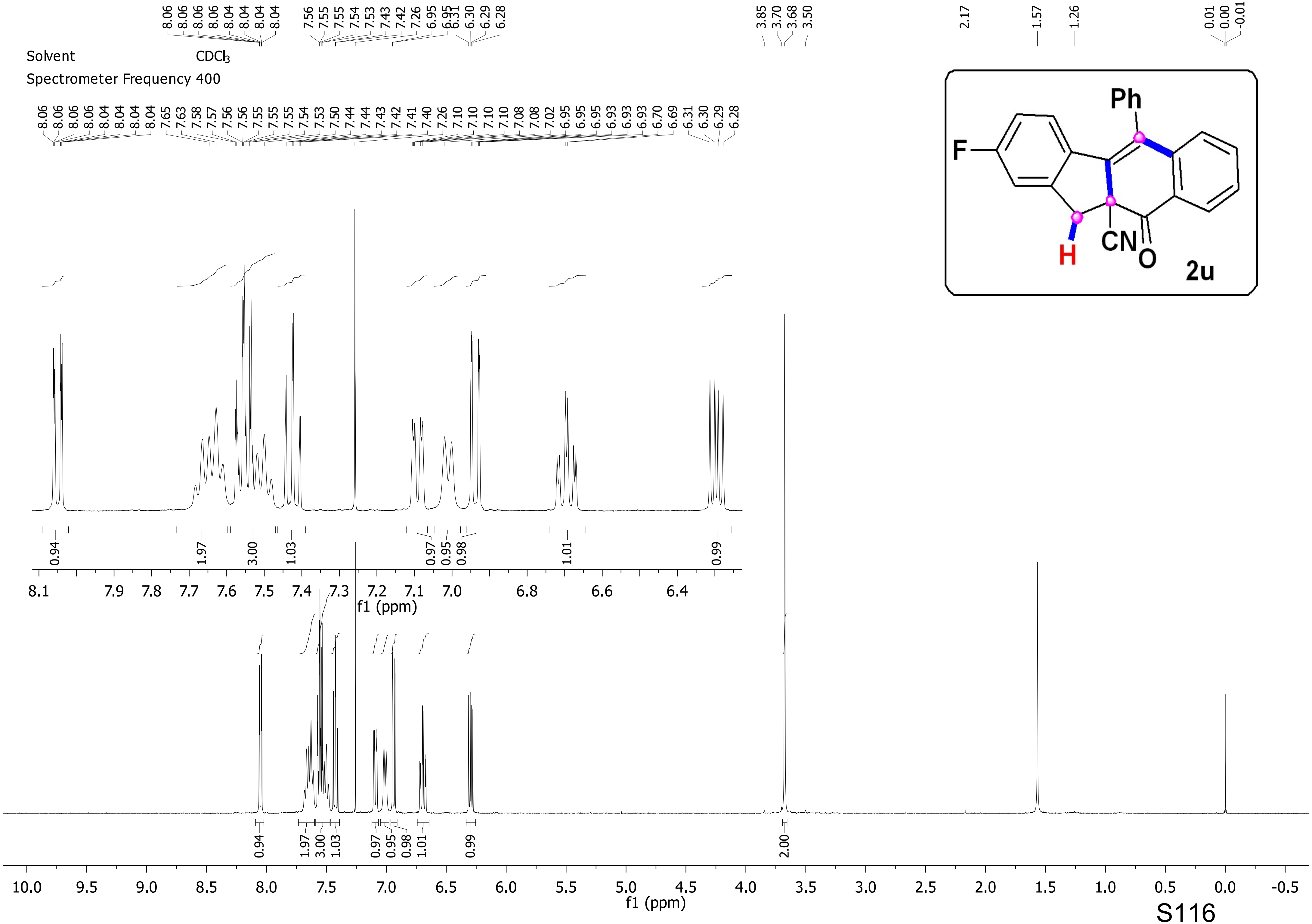
Solvent CDCl3
Spectrometer Frequency 400
F
Ph
H
CN
O
2u
f1 (ppm)

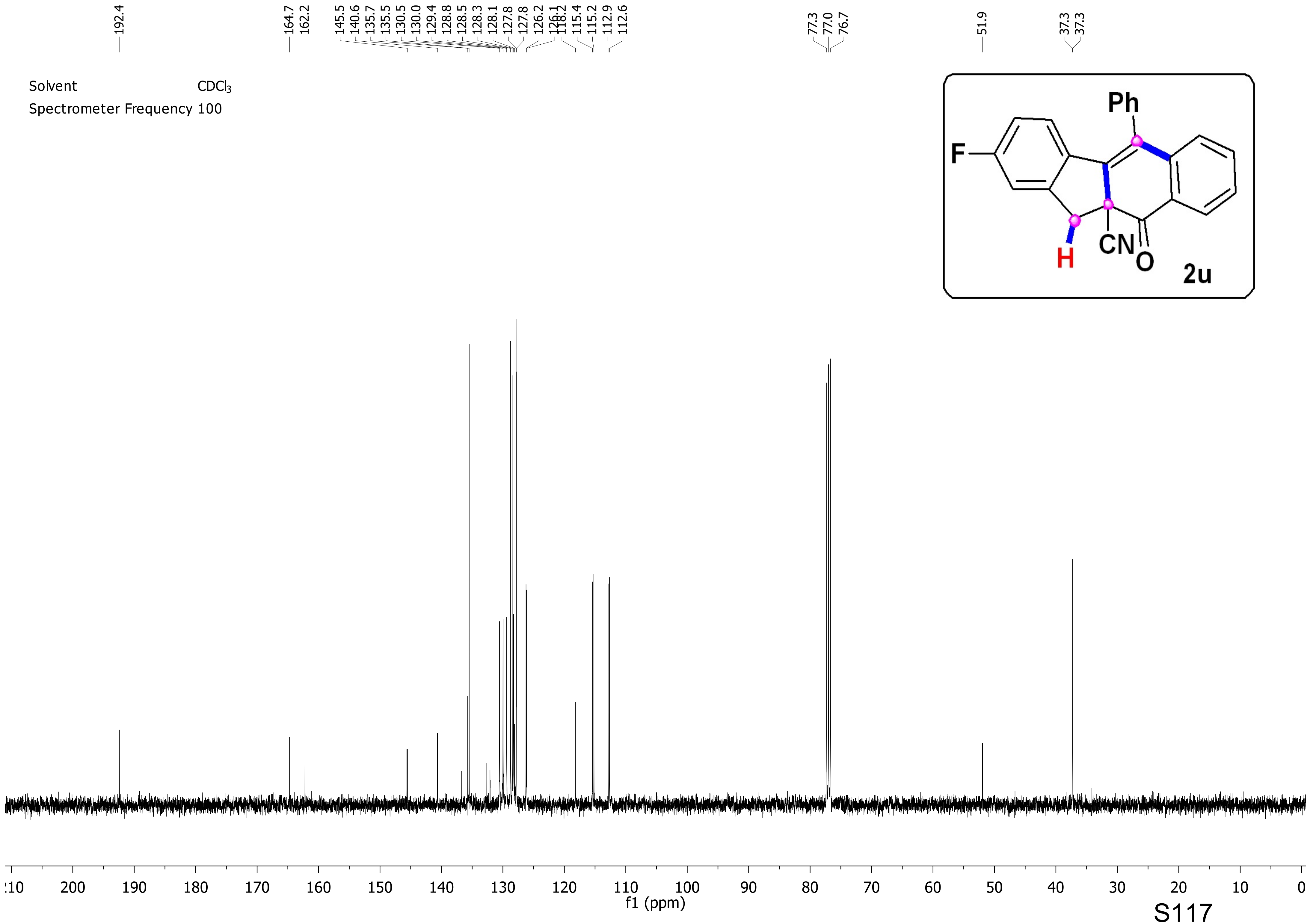
192.4
164.7
162.2
145.5
140.6
135.7
135.5
130.5
130.0
129.4
128.8
128.5
128.3
128.1
127.8
127.8
126.2
126.1
118.2
115.4
115.2
112.9
112.6
77.3
77.0
76.7
51.9
37.3
37.3
Solvent CDCl3
Spectrometer Frequency 100
Ph
F
H
CN
O
2u
f1 (ppm)

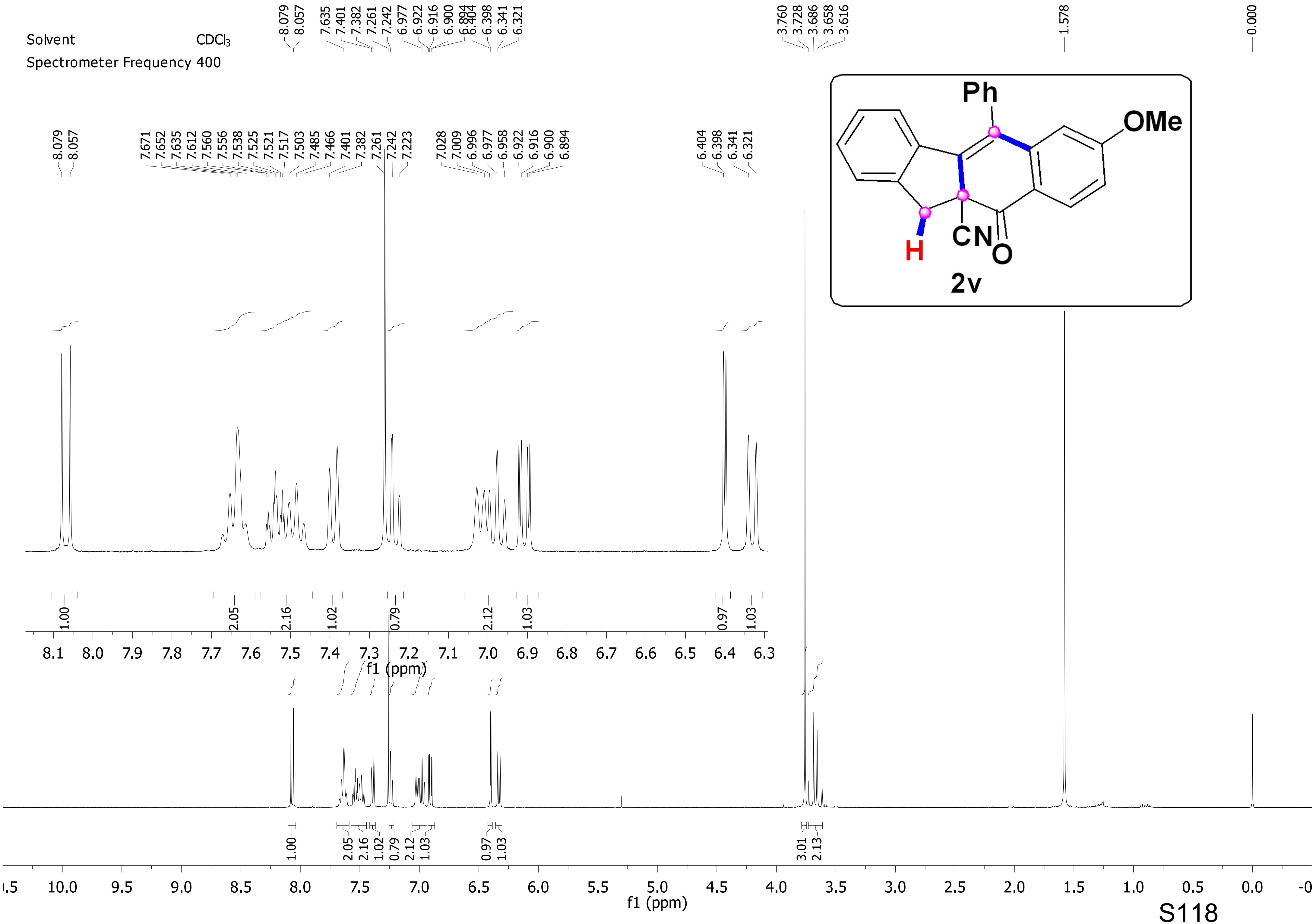
Solvent CDCl3
Spectrometer Frequency 400
8.079 8.057 7.635 7.401 7.382 7.261 7.242 6.977 6.922 6.916 6.900 6.894 6.404 6.398 6.341 6.321
3.760 3.728 3.686 3.658 3.616
1.578
0.000
8.079 8.057
7.671 7.652 7.635 7.612 7.560 7.556 7.538 7.525 7.521 7.517 7.503 7.485 7.466 7.401 7.382 7.261 7.242 7.223
7.028 7.009 6.996 6.977 6.958 6.922 6.916 6.900 6.894
6.404 6.398 6.341 6.321
Ph
OMe
H
CN
O
2v
1.00 2.05 2.16 1.02 0.79 2.12 1.03 0.97 1.03
f1 (ppm)
1.00 2.05 2.16 1.02 0.79 2.12 1.03 0.97 1.03 3.01 2.13
f1 (ppm)

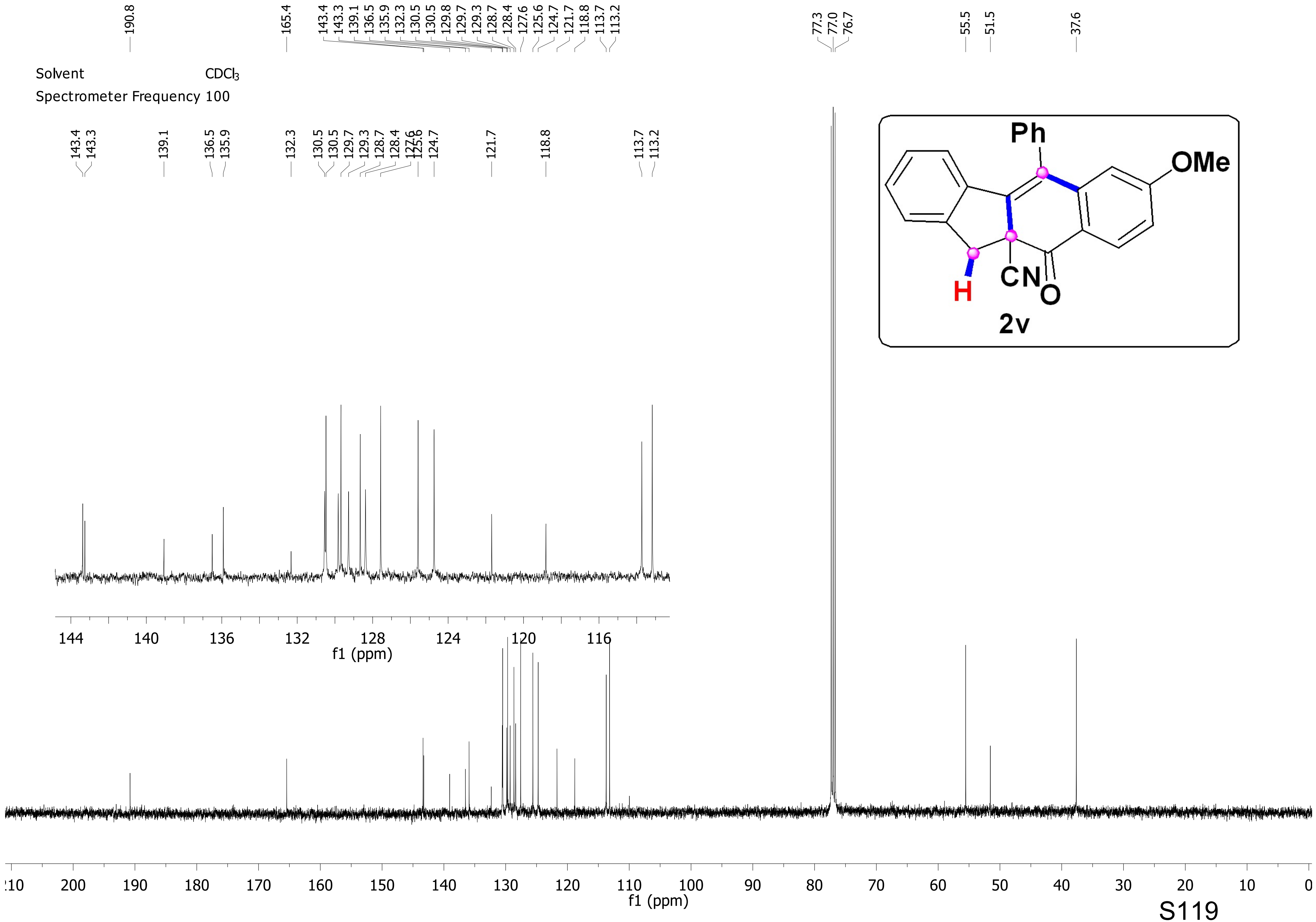
190.8
165.4
143.4 143.3 139.1 136.5 135.9 132.3 130.5 130.5 129.8 129.7 129.3 128.7 128.4 127.6 125.6 124.7 121.7 118.8 113.7 113.2
77.3 77.0 76.7
55.5 51.5
37.6
Solvent CDCl3
Spectrometer Frequency 100
143.4 143.3 139.1 136.5 135.9 132.3 130.5 130.5 129.7 129.3 128.7 128.4 127.6 125.6 124.7 121.7 118.8 113.7 113.2
Ph
OMe
H
CN
O
2v
f1 (ppm)
f1 (ppm)

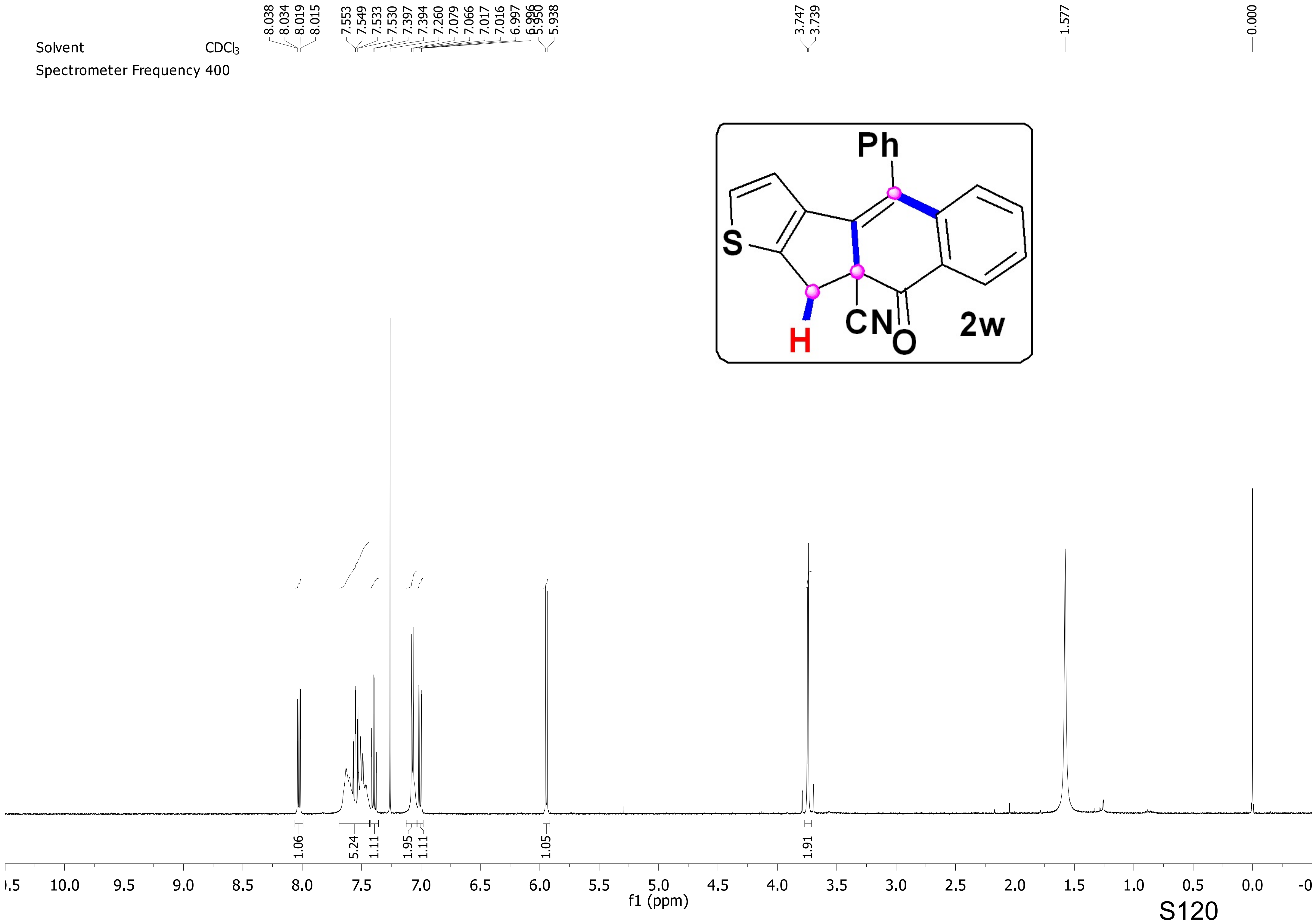
Solvent CDCl3
Spectrometer Frequency 400
8.038 8.034 8.019 8.015 7.553 7.549 7.533 7.530 7.397 7.394 7.260 7.079 7.066 7.017 7.016 6.997 6.996 5.950 5.938
3.747 3.739
1.577
0.000
Ph
S
H
CN
O
2w
1.06 5.24 1.11 1.95 1.11 1.05 1.91
10.0 9.5 9.0 8.5 8.0 7.5 7.0 6.5 6.0 5.5 5.0 4.5 4.0 3.5 3.0 2.5 2.0 1.5 1.0 0.5 0.0
f1 (ppm)

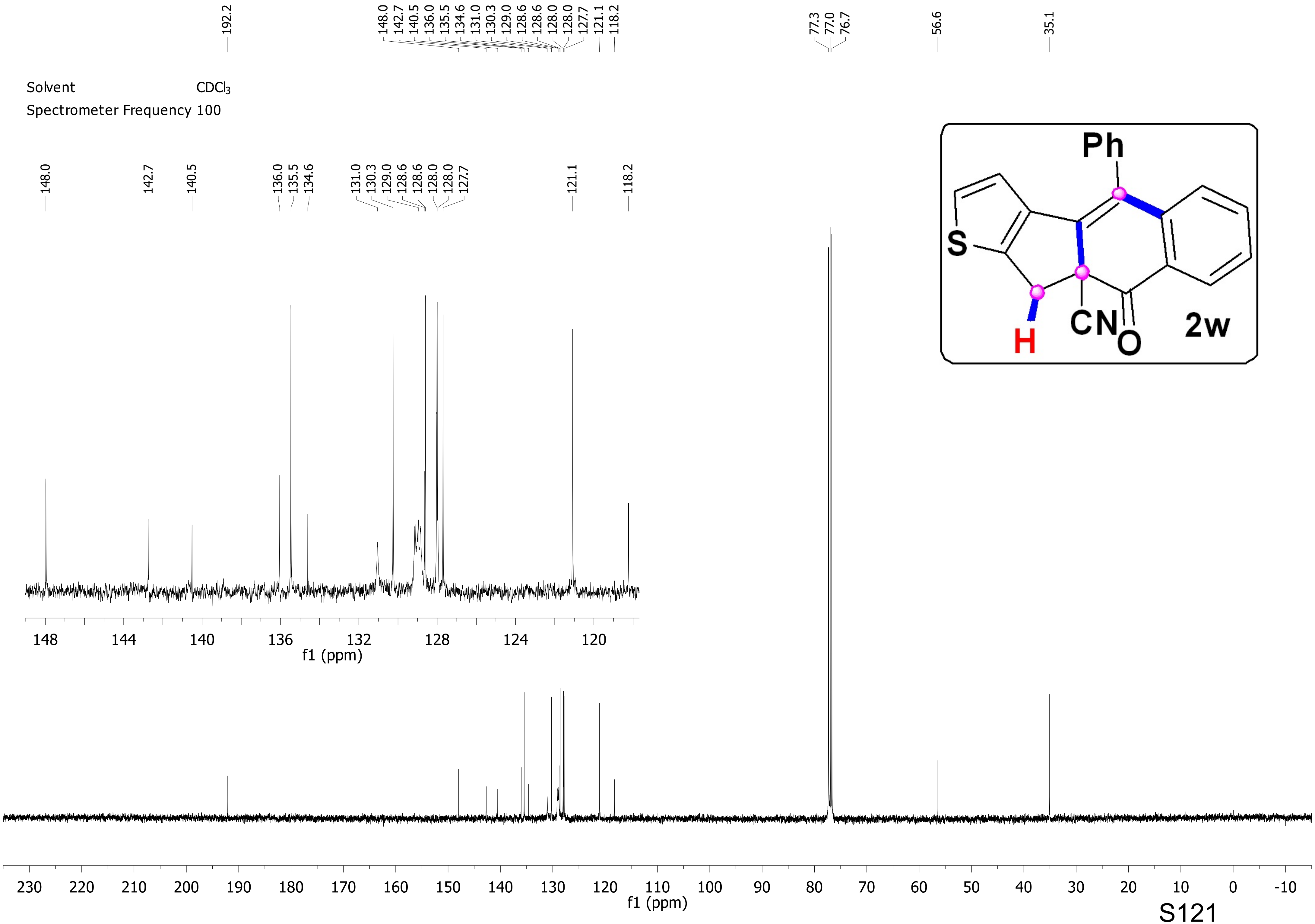

192.2
148.0 142.7 140.5 136.0 135.5 134.6 131.0 130.3 129.0 128.6 128.6 128.0 128.0 127.7 121.1 118.2
77.3 77.0 76.7
56.6
35.1
Solvent CDCl3
Spectrometer Frequency 100
Ph
S
H
CN
O
2w
148 144 140 136 132 128 124 120
f1 (ppm)
230 220 210 200 190 180 170 160 150 140 130 120 110 100 90 80 70 60 50 40 30 20 10 0 -10
f1 (ppm)

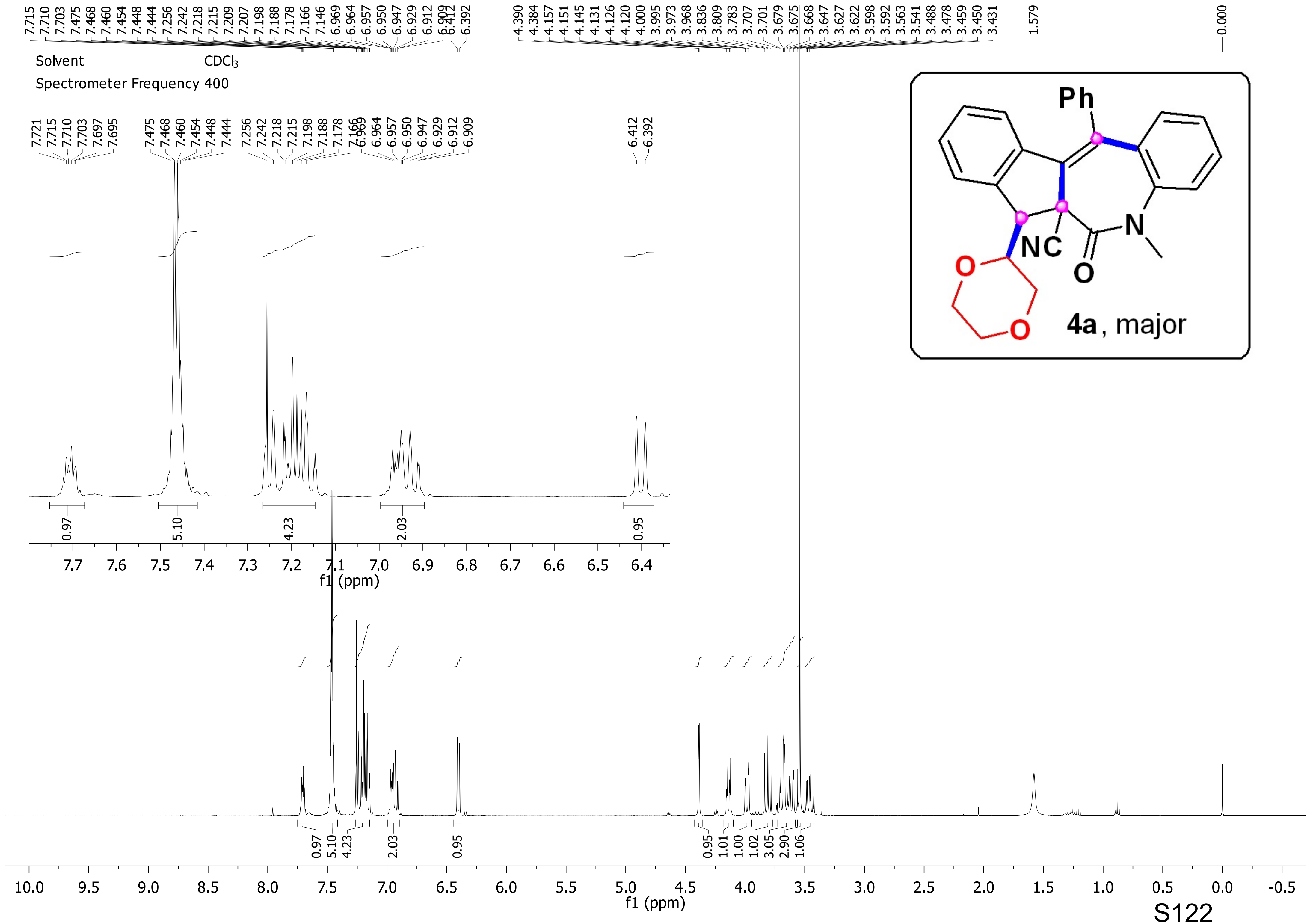

Solvent CDCl3
Spectrometer Frequency 400
Ph
NC
N
O
O
O
4a, major
f1 (ppm)

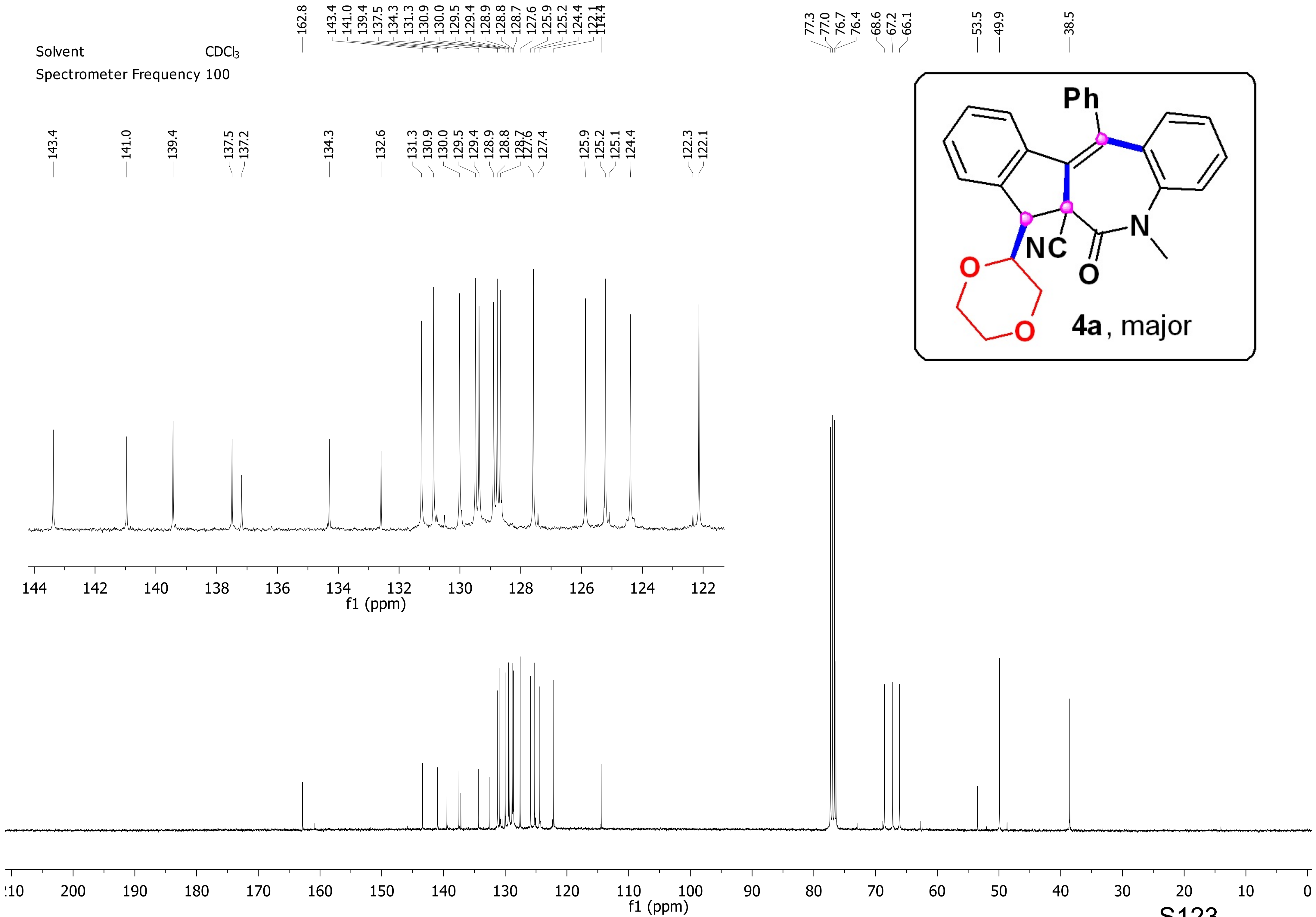
Solvent CDCl3
Spectrometer Frequency 100
162.8
143.4 141.0 139.4 137.5 134.3 131.3 130.9 130.0 129.5 129.4 128.9 128.8 128.7 127.6 125.9 125.2 124.4 122.1 114.4
77.3 77.0 76.7 76.4 68.6 67.2 66.1
53.5 49.9 38.5
143.4 141.0 139.4 137.5 137.2 134.3 132.6 131.3 130.9 130.0 129.5 129.4 128.9 128.8 128.7 127.6 127.4 125.9 125.2 125.1 124.4 122.3 122.1
Ph
NC
N
O
O
O
4a, major
f1 (ppm)
f1 (ppm)

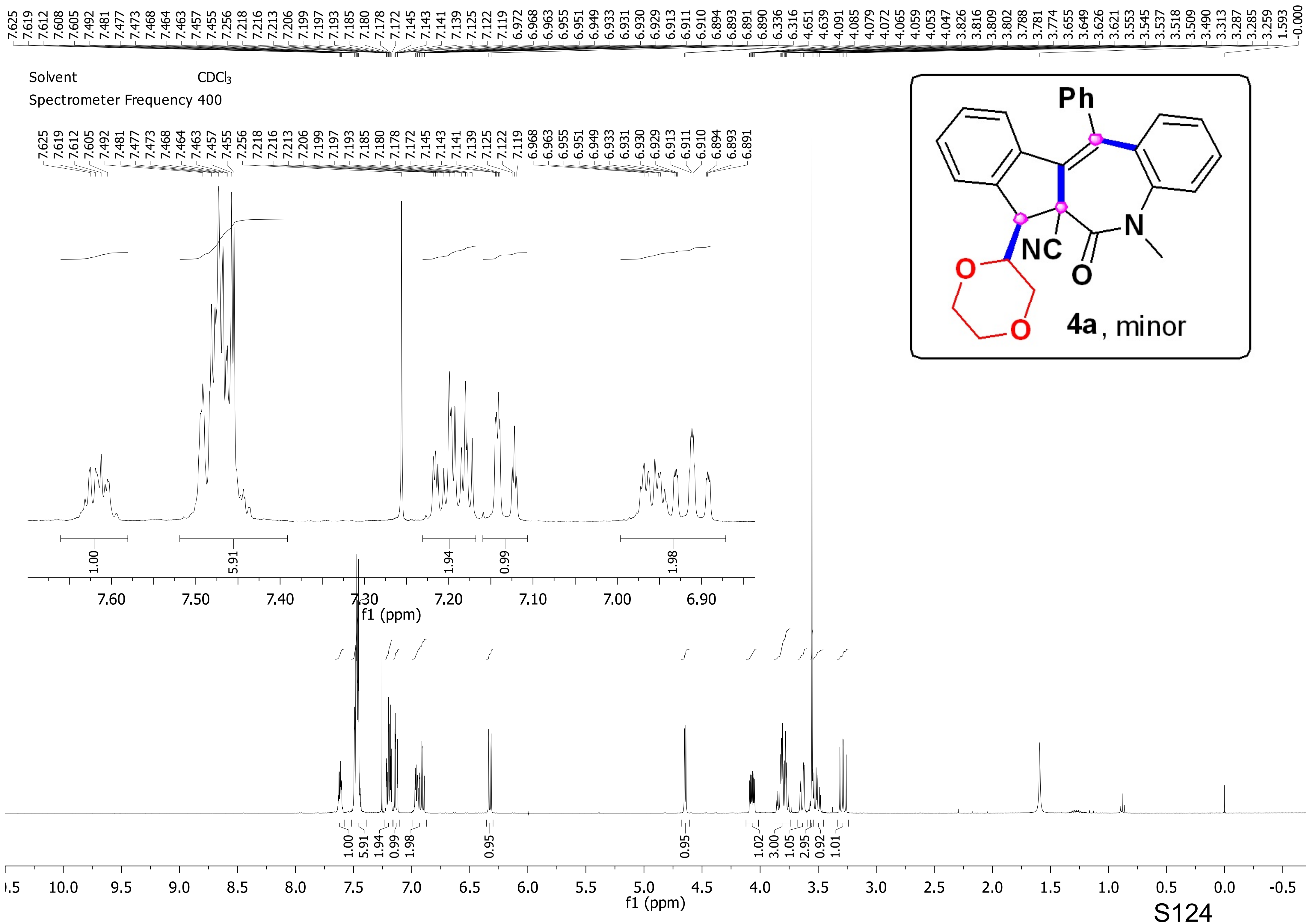
7.625 7.619 7.612 7.608 7.605 7.492 7.481 7.477 7.473 7.468 7.464 7.463 7.457 7.455 7.256 7.218 7.216 7.213 7.206 7.199 7.197 7.193 7.185 7.180 7.178 7.172 7.145 7.143 7.141 7.139 7.125 7.122 7.119 6.972 6.968 6.963 6.955 6.951 6.949 6.933 6.931 6.930 6.929 6.913 6.911 6.910 6.894 6.893 6.891 6.890 6.336 6.316 4.651 4.639 4.091 4.085 4.079 4.072 4.065 4.059 4.053 4.047 3.826 3.816 3.809 3.802 3.788 3.781 3.774 3.655 3.649 3.626 3.621 3.553 3.545 3.537 3.518 3.509 3.490 3.313 3.287 3.285 3.259 1.593 -0.000
Solvent CDCl3
Spectrometer Frequency 400
Ph
NC
N
O
4a, minor
f1 (ppm)

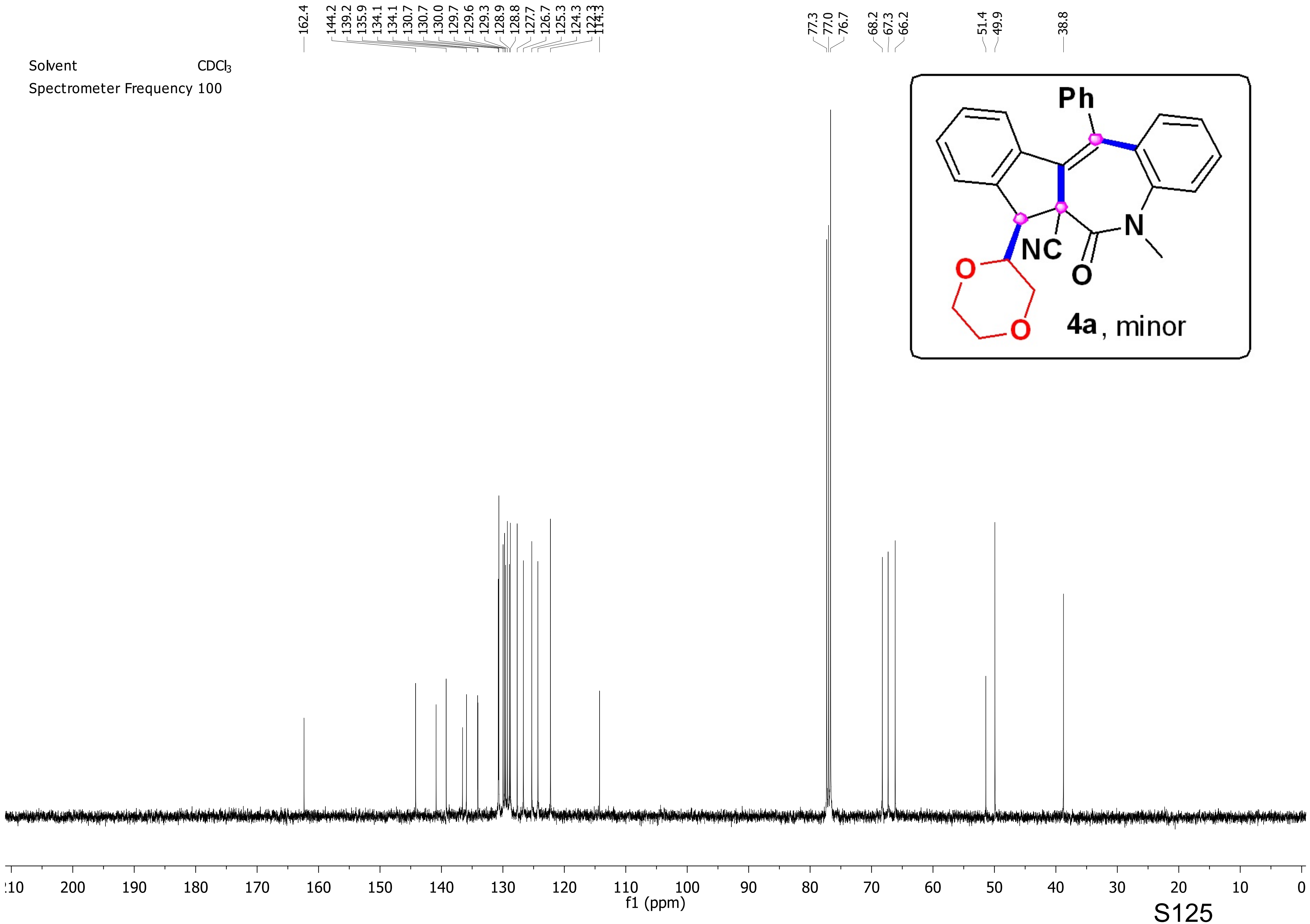
162.4
144.2
139.2
135.9
134.1
134.1
130.7
130.7
130.0
129.7
129.6
129.3
128.9
128.8
127.7
126.7
125.3
124.3
122.3
114.3
77.3
77.0
76.7
68.2
67.3
66.2
51.4
49.9
38.8
Solvent CDCl3
Spectrometer Frequency 100
Ph
N
NC
O
O
O
4a, minor
210
200
190
180
170
160
150
140
130
120
110
100
90
80
70
60
50
40
30
20
10
0
f1 (ppm)

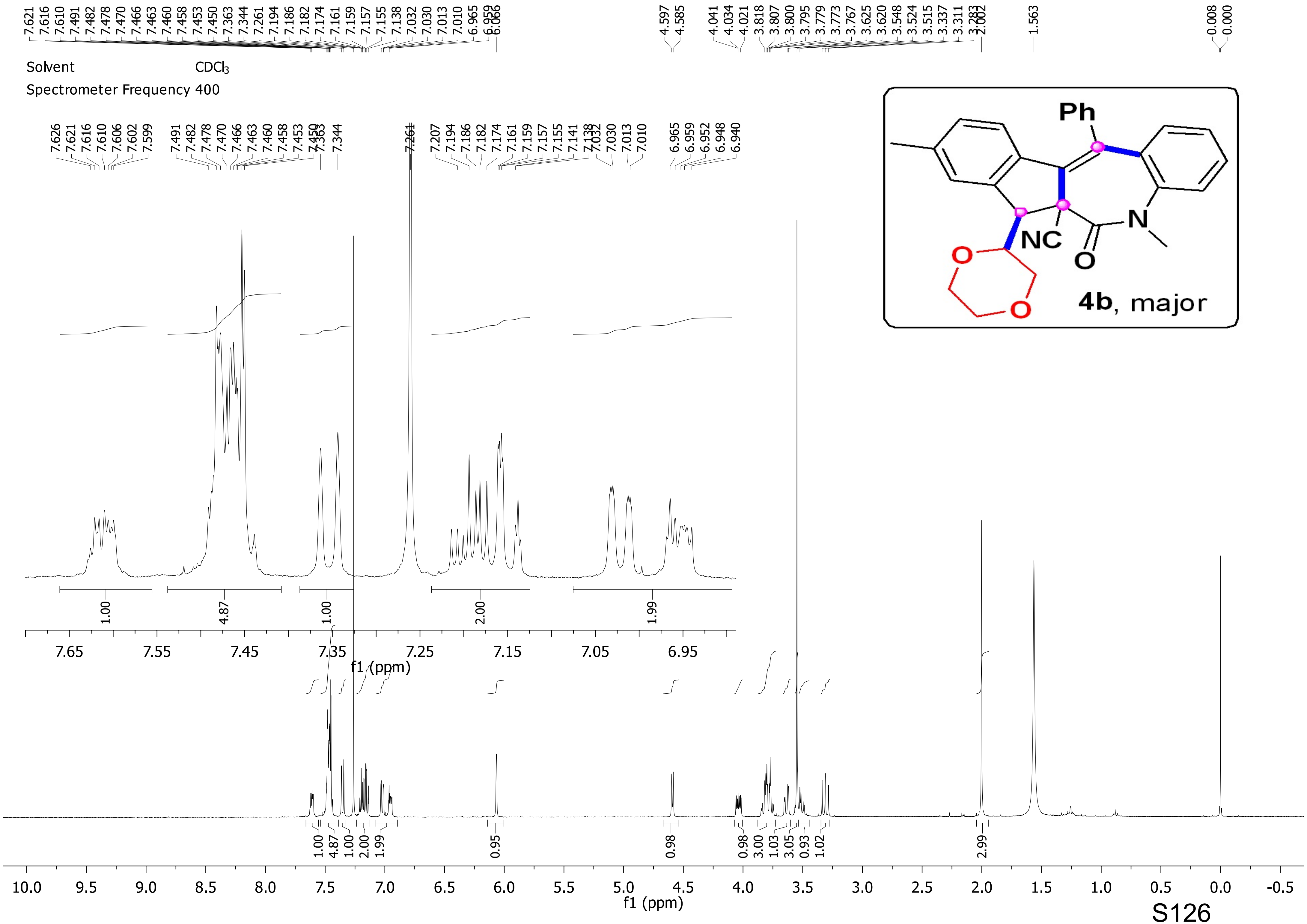
Solvent CDCl3
Spectrometer Frequency 400
Ph
NC
N
O
4b, major
f1 (ppm)

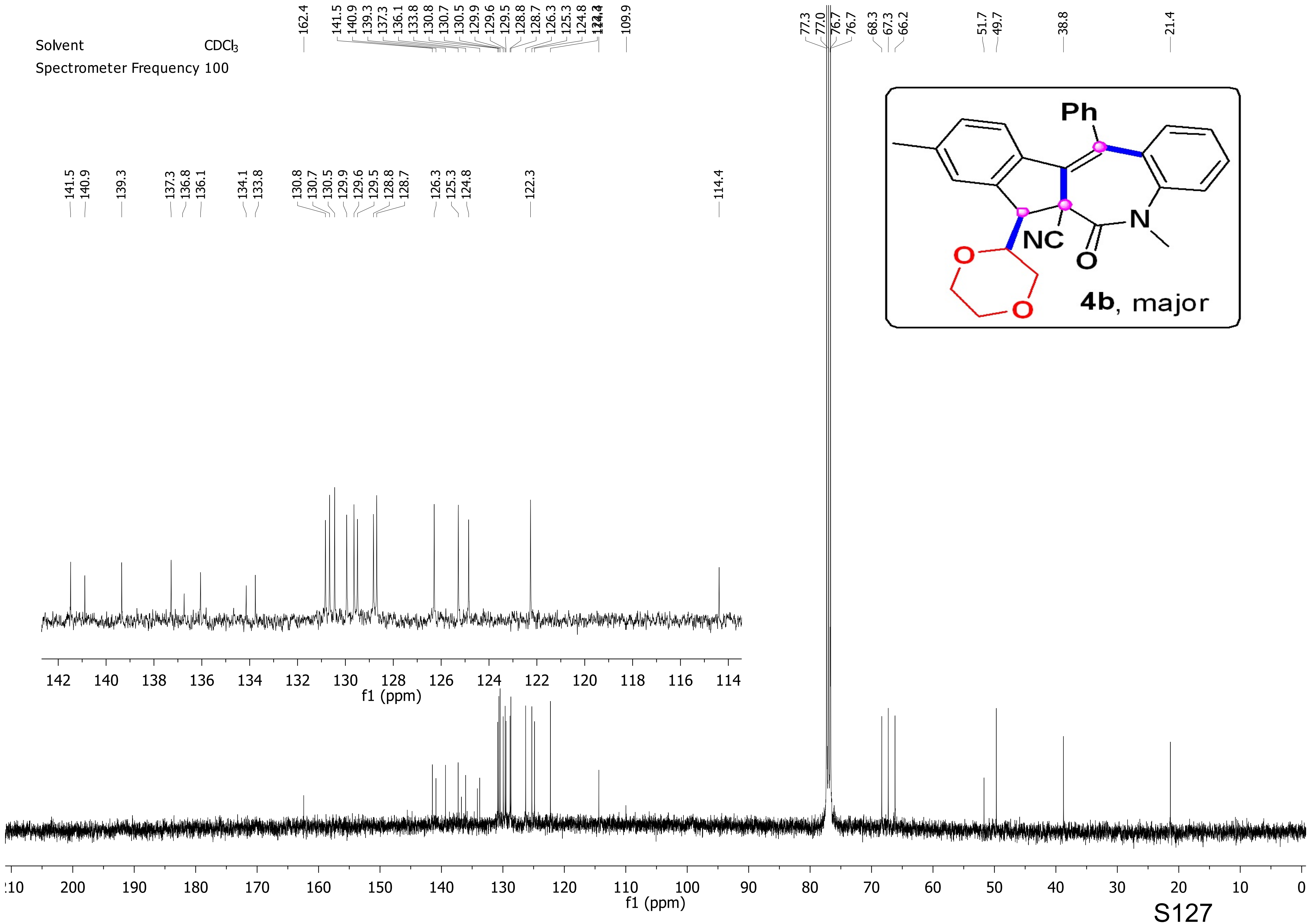
Solvent CDCl3
Spectrometer Frequency 100
162.4
141.5
140.9
139.3
137.3
136.1
133.8
130.8
130.7
130.5
129.9
129.6
129.5
128.8
128.7
126.3
125.3
124.8
109.9
77.3
77.0
76.7
76.7
68.3
67.3
66.2
51.7
49.7
38.8
21.4
141.5
140.9
139.3
137.3
136.8
136.1
134.1
133.8
130.8
130.7
130.5
129.9
129.6
129.5
128.8
128.7
126.3
125.3
124.8
122.3
114.4
Ph
NC
N
O
O
O
4b, major
f1 (ppm)
f1 (ppm)

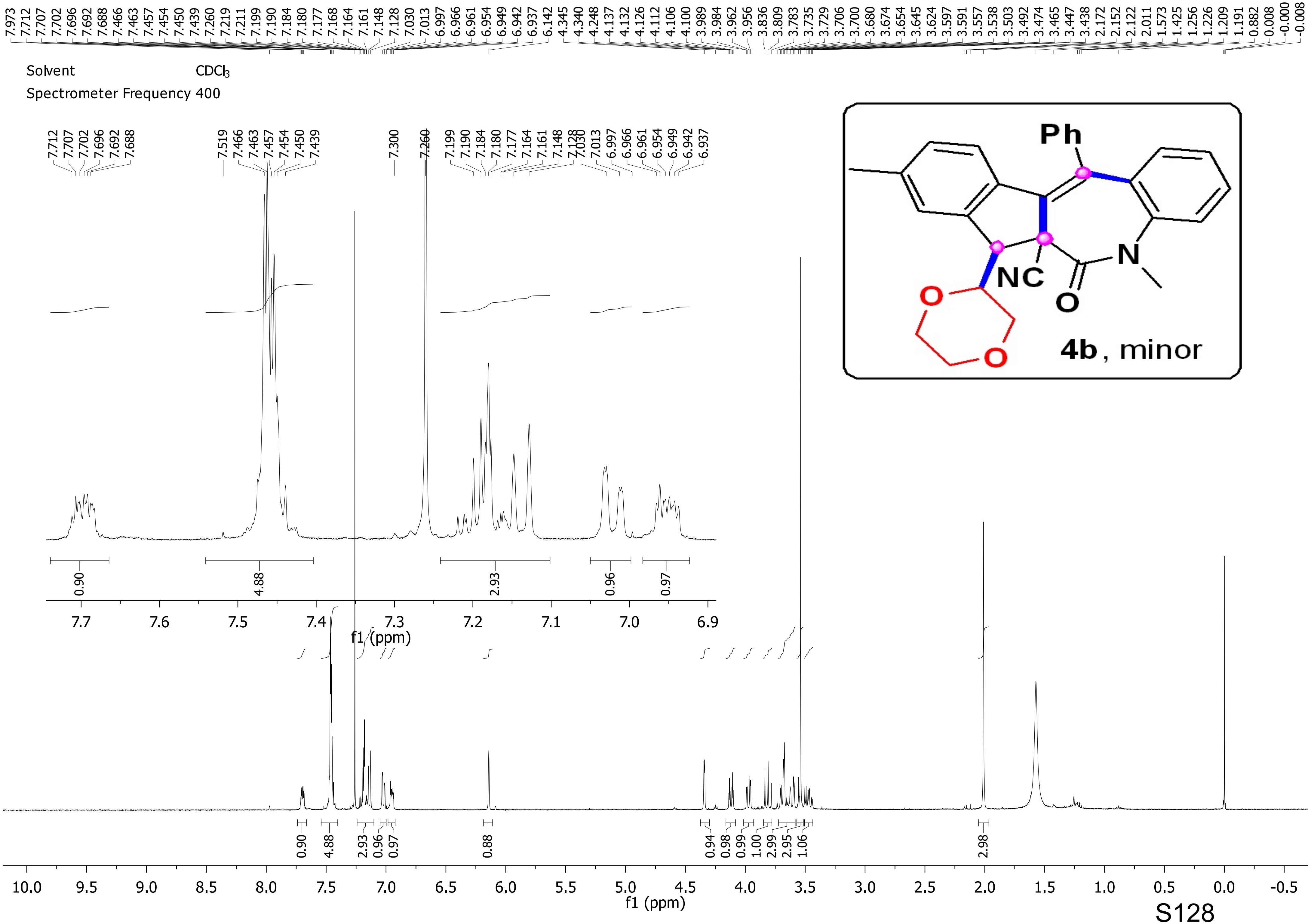
7.973 7.712 7.707 7.702 7.696 7.692 7.688 7.466 7.463 7.457 7.454 7.450 7.439 7.260 7.219 7.211 7.199 7.190 7.184 7.180 7.177 7.168 7.164 7.161 7.148 7.128 7.030 7.013 6.997 6.966 6.961 6.954 6.949 6.942 6.937 6.142 4.345 4.340 4.248 4.137 4.132 4.126 4.112 4.106 4.100 3.989 3.984 3.962 3.956 3.836 3.809 3.783 3.735 3.729 3.706 3.700 3.680 3.674 3.654 3.645 3.624 3.597 3.591 3.557 3.538 3.503 3.492 3.474 3.465 3.447 3.438 2.172 2.152 2.122 2.011 1.573 1.425 1.256 1.226 1.209 1.191 0.882 0.008 -0.000 -0.008
Solvent CDCl3
Spectrometer Frequency 400
Ph
NC
O
N
O
O
4b, minor
0.90 4.88 2.93 0.96 0.97
f1 (ppm)
0.90 4.88 2.93 0.96 0.97 0.88 0.94 0.98 0.99 1.00 2.99 2.95 1.06 2.98
f1 (ppm)

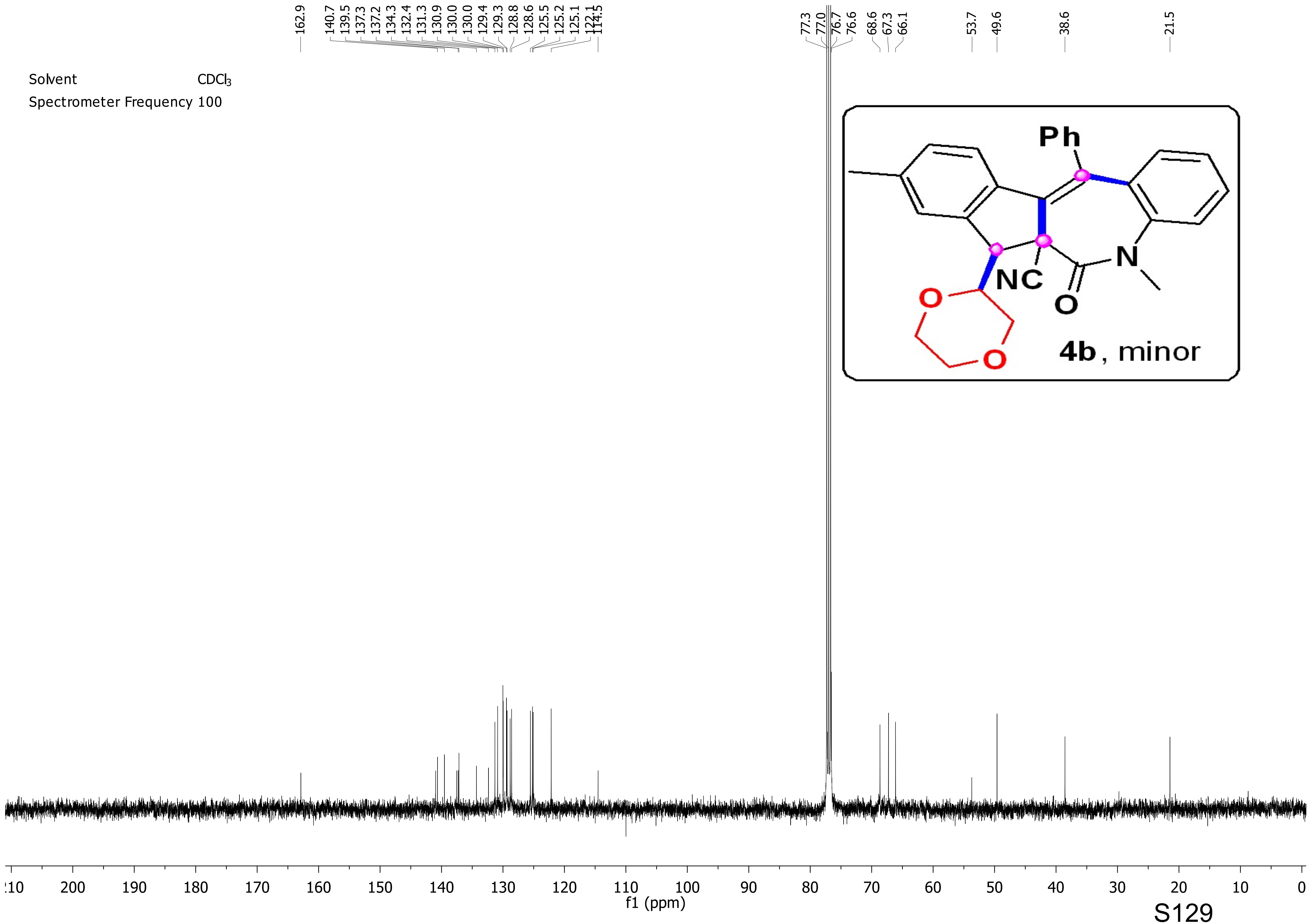
162.9
140.7
139.5
137.3
137.2
134.3
132.4
131.3
130.9
130.0
130.0
129.4
129.3
128.8
128.6
125.5
125.2
125.1
122.1
114.5
77.3
77.0
76.7
76.6
68.6
67.3
66.1
53.7
49.6
38.6
21.5
Solvent CDCl3
Spectrometer Frequency 100
Ph
N
NC
O
O
O
4b, minor
f1 (ppm)

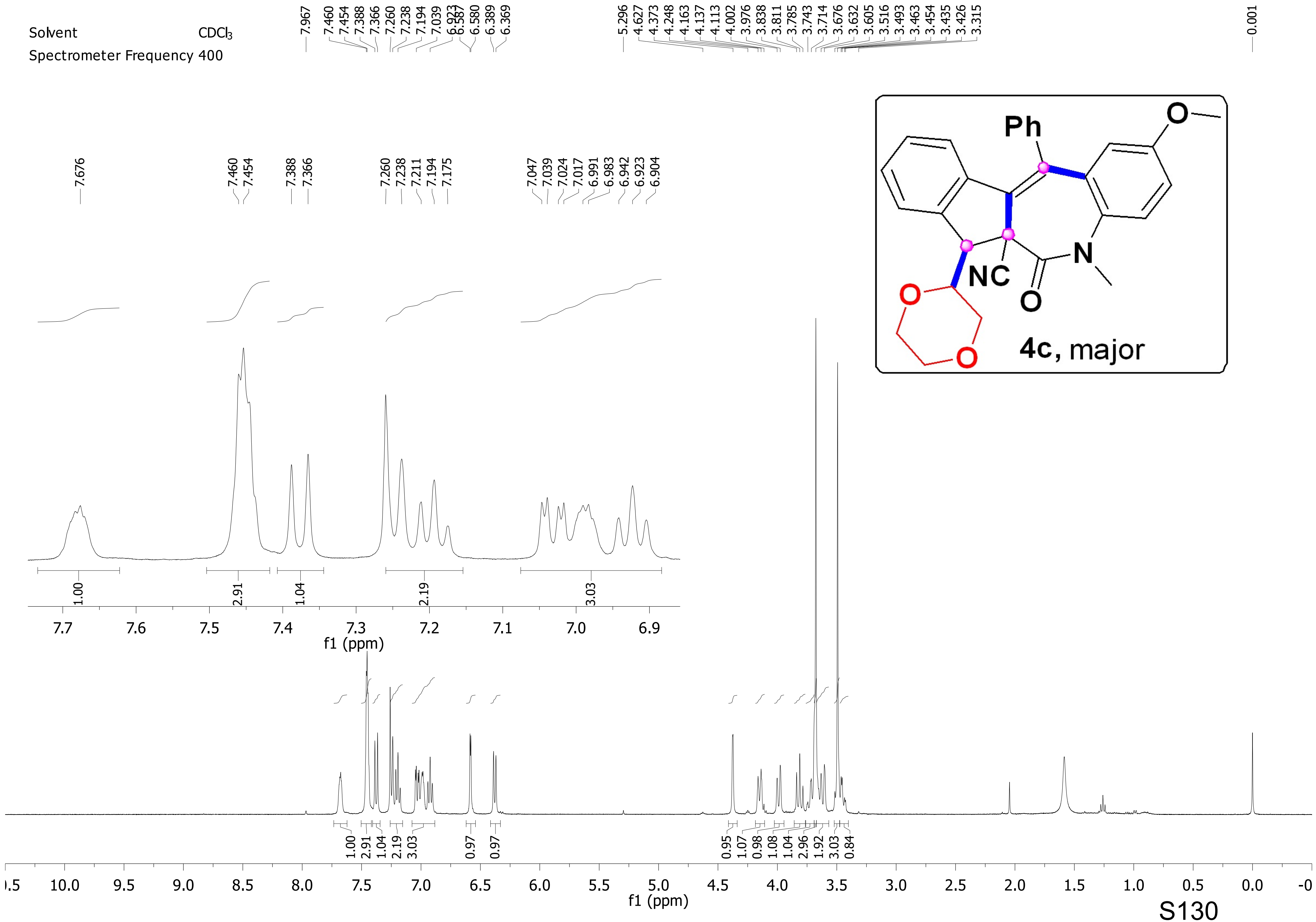
Solvent CDCl3
Spectrometer Frequency 400
7.967 7.460 7.454 7.388 7.366 7.260 7.238 7.194 7.039 6.923 6.587 6.580 6.389 6.369
5.296 4.627 4.373 4.248 4.163 4.137 4.113 4.002 3.976 3.838 3.811 3.785 3.743 3.714 3.676 3.632 3.605 3.516 3.493 3.463 3.454 3.435 3.426 3.315
0.001
7.676 7.460 7.454 7.388 7.366 7.260 7.238 7.211 7.194 7.175 7.047 7.039 7.024 7.017 6.991 6.983 6.942 6.923 6.904
1.00 2.91 1.04 2.19 3.03
f1 (ppm)
1.00 2.91 1.04 2.19 3.03 0.97 0.97 0.95 1.07 0.98 1.08 1.04 2.96 1.92 3.03 0.84
f1 (ppm)
Ph
O
N
NC
O
O
O
4c, major

Solvent CDCl$_3$

Spectrometer Frequency 100

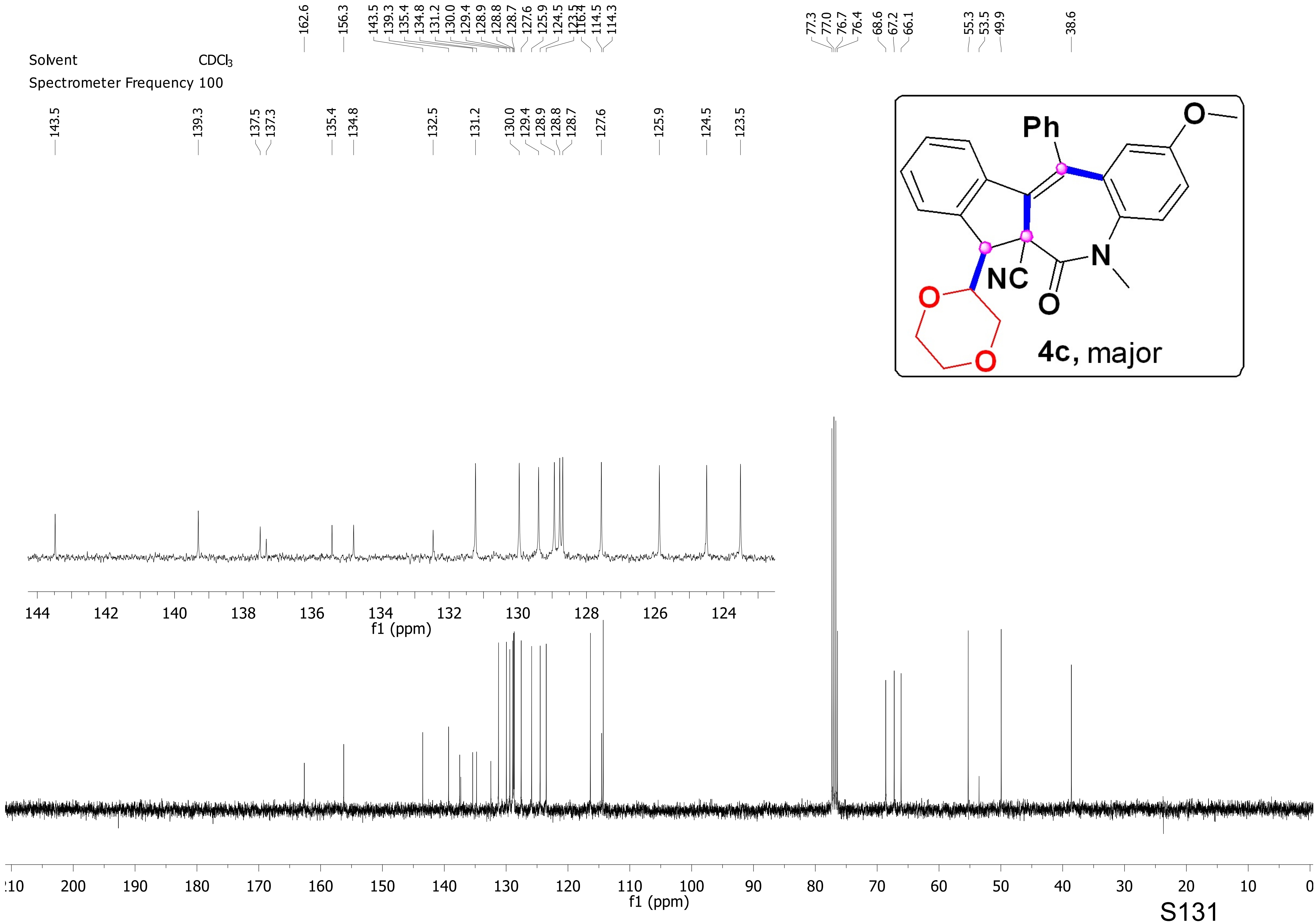

4c, major

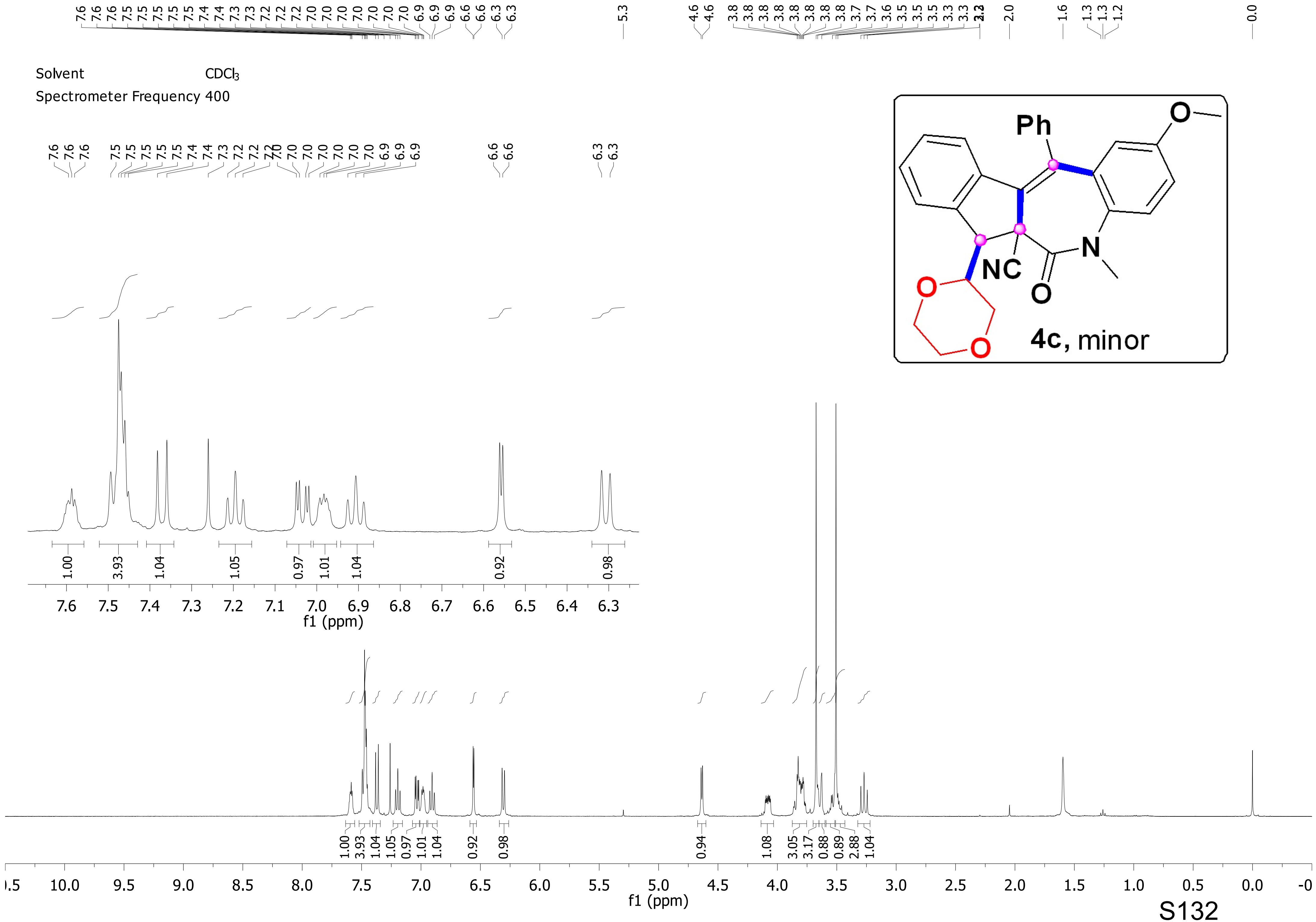
Solvent CDCl3
Spectrometer Frequency 400
f1 (ppm)
Ph
NC
N
O
4c, minor

Solvent CDCl$_3$
Spectrometer Frequency 100

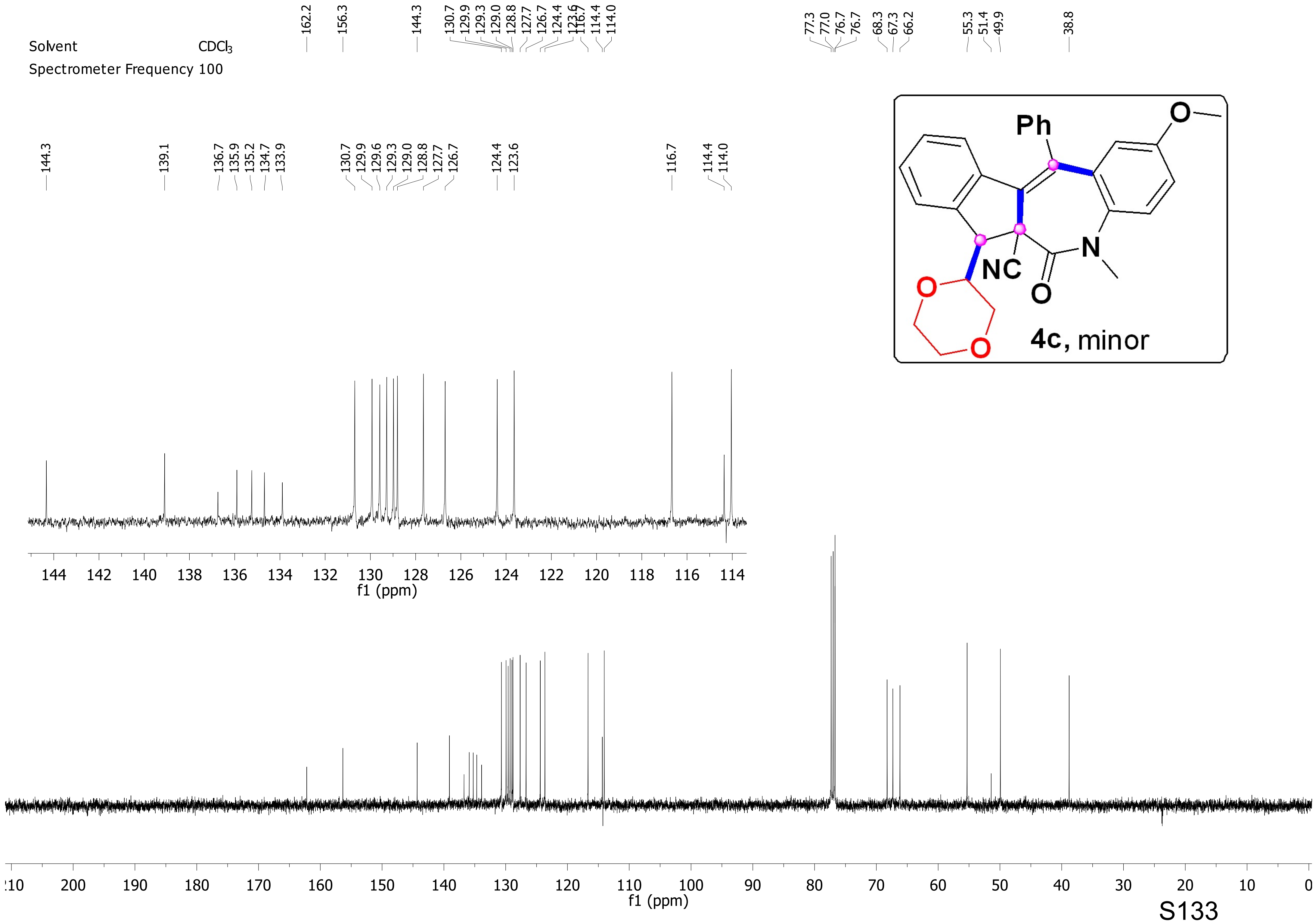

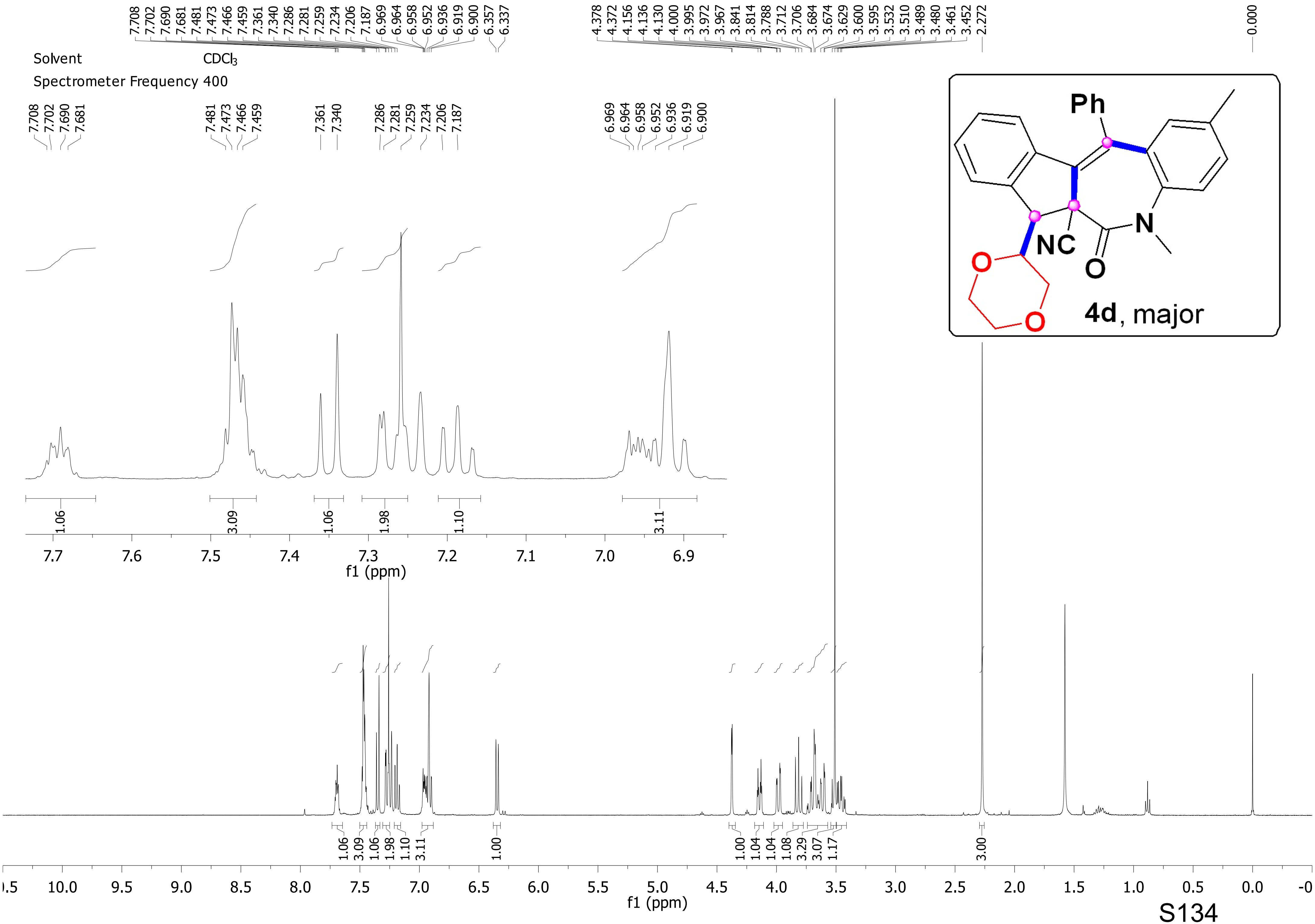
Solvent CDCl3
Spectrometer Frequency 400
4d, major
Ph
NC
N
O
f1 (ppm)

Solvent CDCl$_3$

Spectrometer Frequency 100

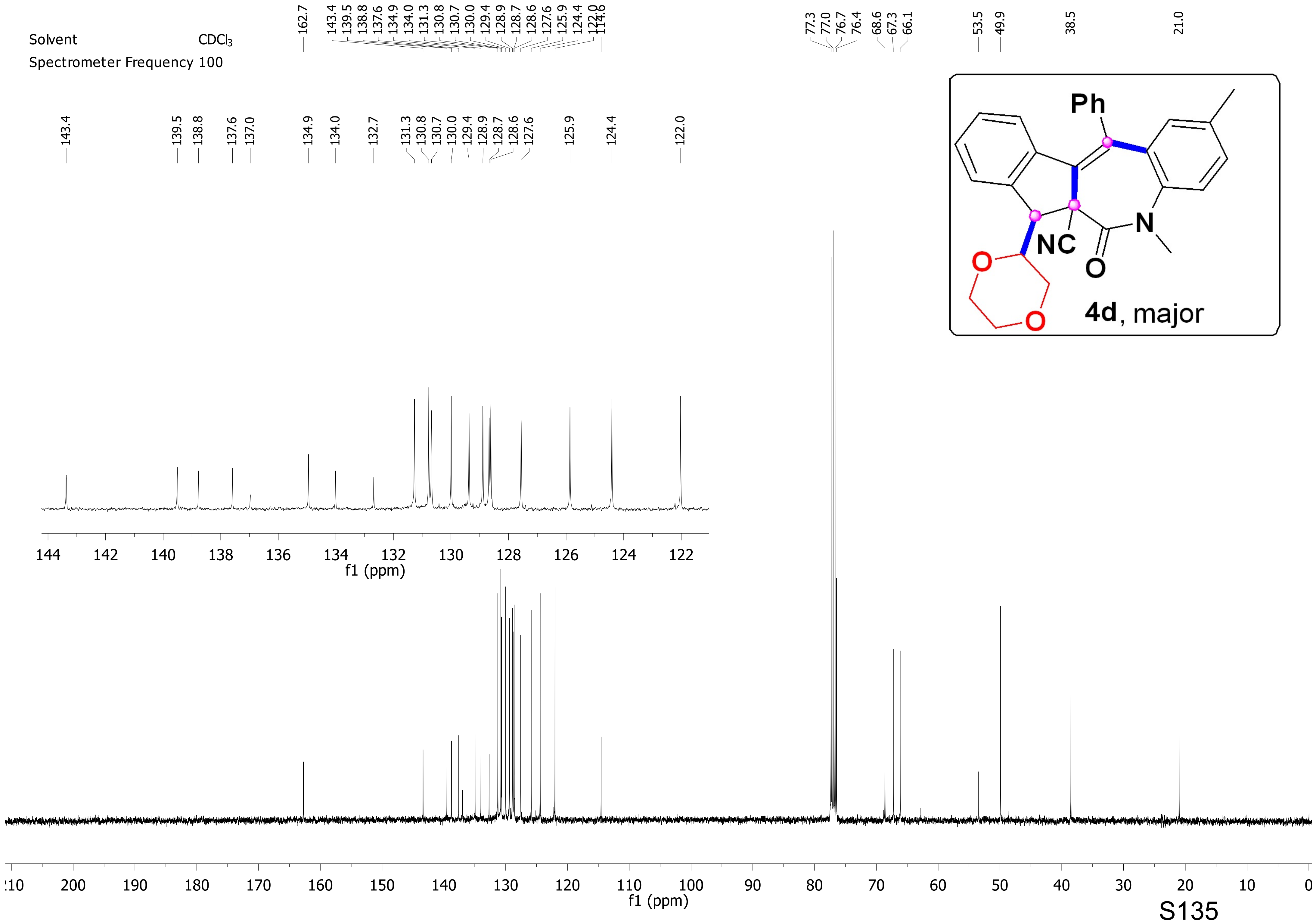

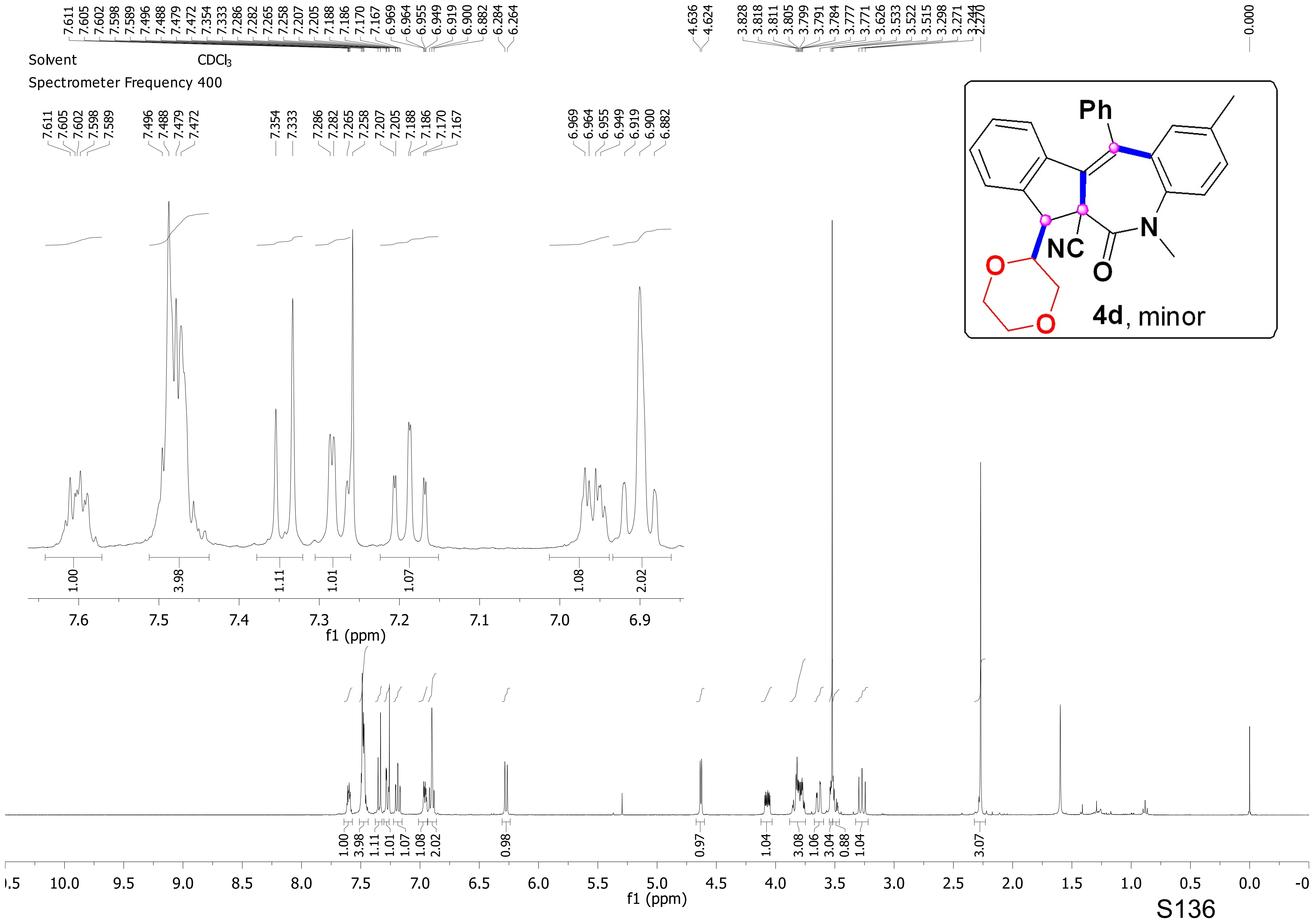

4d, minor

Solvent CDCl$_3$
Spectrometer Frequency 100

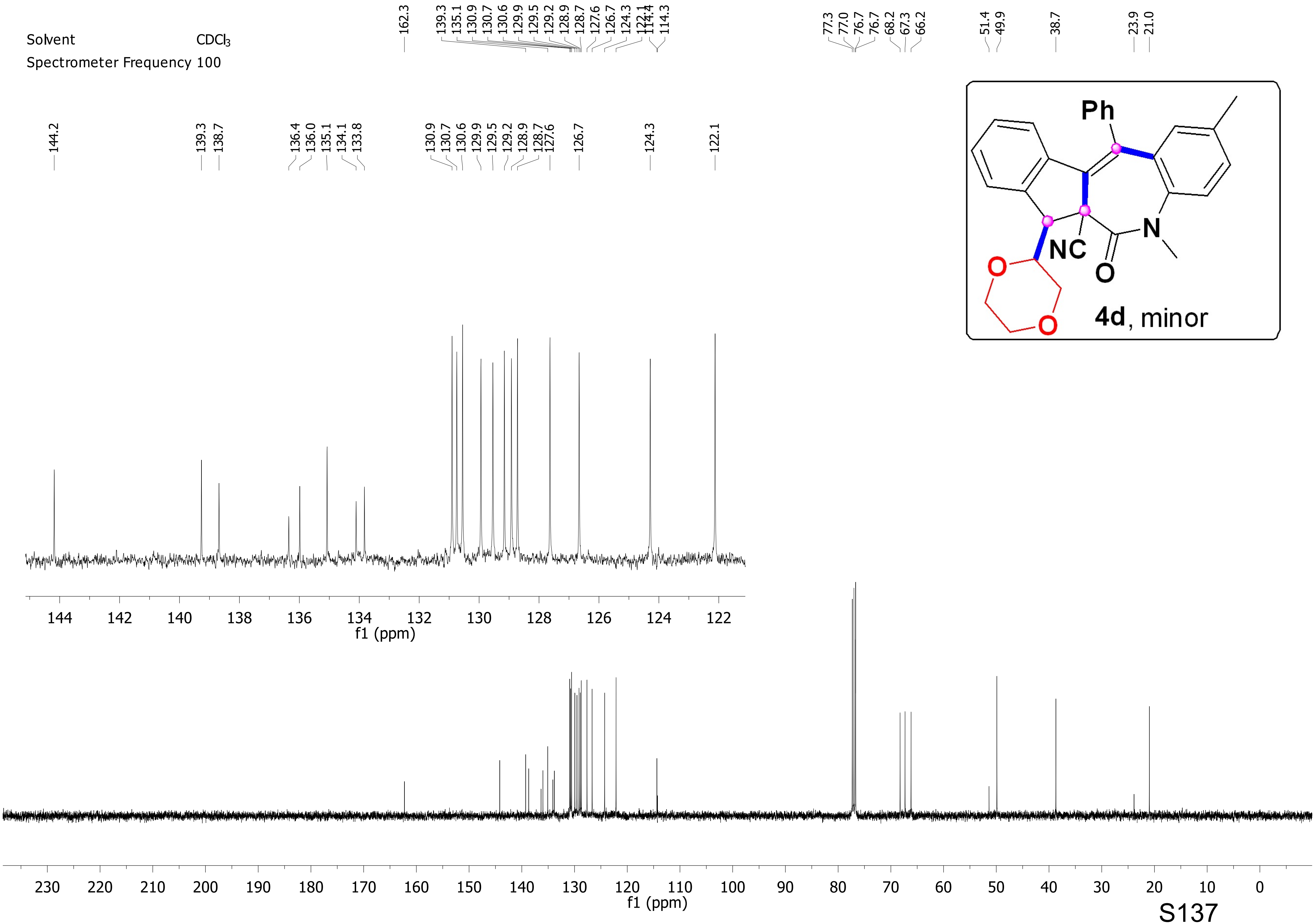

4d, minor

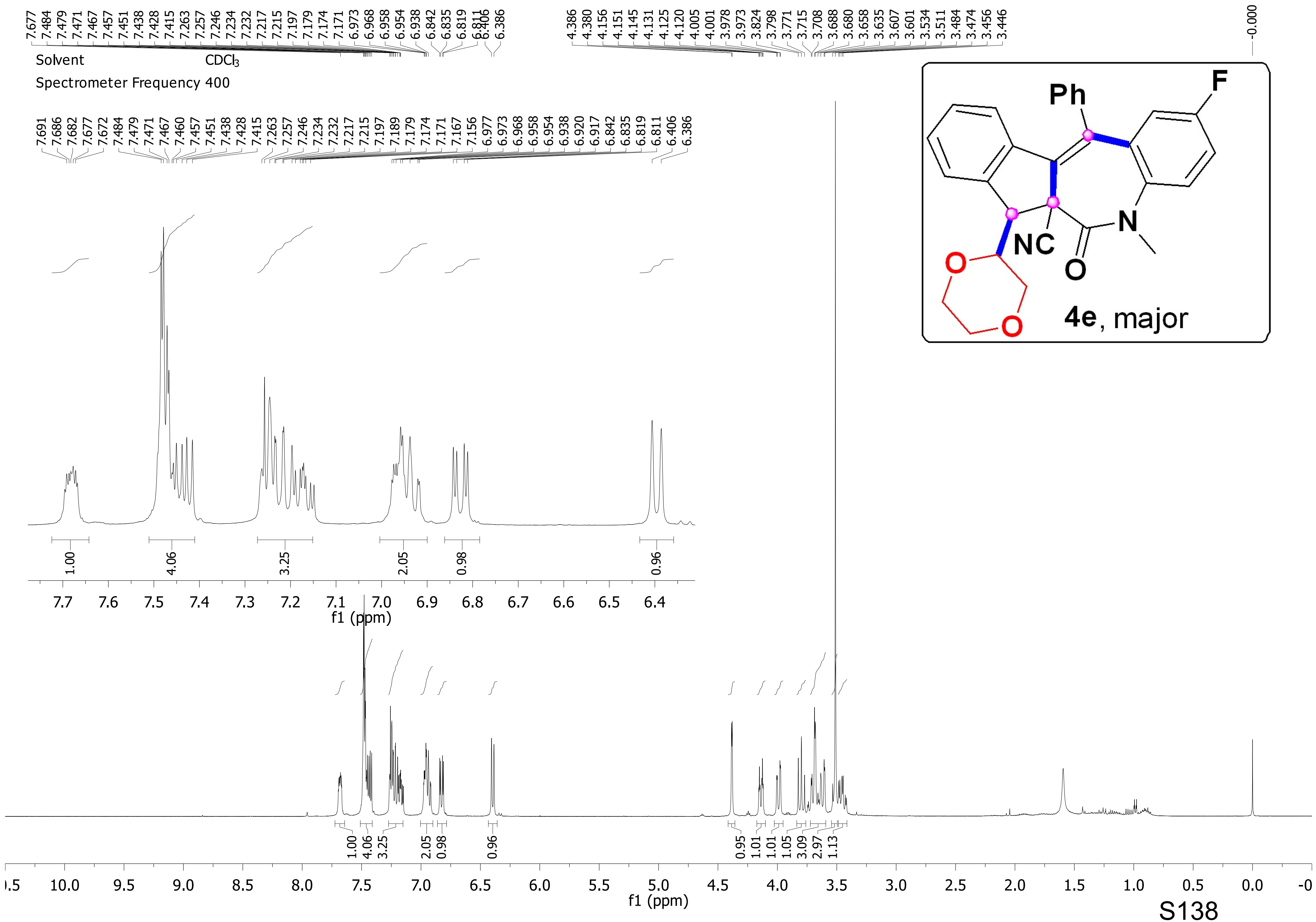
7.677 7.484 7.479 7.471 7.467 7.457 7.451 7.438 7.428 7.415 7.263 7.257 7.246 7.234 7.232 7.217 7.215 7.197 7.179 7.174 7.171 6.973 6.968 6.958 6.954 6.938 6.842 6.835 6.819 6.811 6.406 6.386
4.386 4.380 4.156 4.151 4.145 4.131 4.125 4.120 4.005 4.001 3.978 3.973 3.824 3.798 3.771 3.715 3.708 3.688 3.680 3.658 3.635 3.607 3.601 3.534 3.511 3.484 3.474 3.456 3.446
-0.000
Solvent CDCl3
Spectrometer Frequency 400
7.691 7.686 7.682 7.677 7.672 7.484 7.479 7.471 7.467 7.460 7.457 7.451 7.438 7.428 7.415 7.263 7.257 7.246 7.234 7.232 7.217 7.215 7.197 7.189 7.179 7.174 7.171 7.167 7.156 6.977 6.973 6.968 6.958 6.954 6.938 6.920 6.917 6.842 6.835 6.819 6.811 6.406 6.386
1.00 4.06 3.25 2.05 0.98 0.96
7.7 7.6 7.5 7.4 7.3 7.2 7.1 7.0 6.9 6.8 6.7 6.6 6.5 6.4
f1 (ppm)
Ph
F
N
NC
O
O
O
4e, major
1.00 4.06 3.25 2.05 0.98 0.96 0.95 1.01 1.01 1.05 3.09 2.97 1.13
10.0 9.5 9.0 8.5 8.0 7.5 7.0 6.5 6.0 5.5 5.0 4.5 4.0 3.5 3.0 2.5 2.0 1.5 1.0 0.5 0.0
f1 (ppm)

Solvent CDCl$_3$

Spectrometer Frequency 100

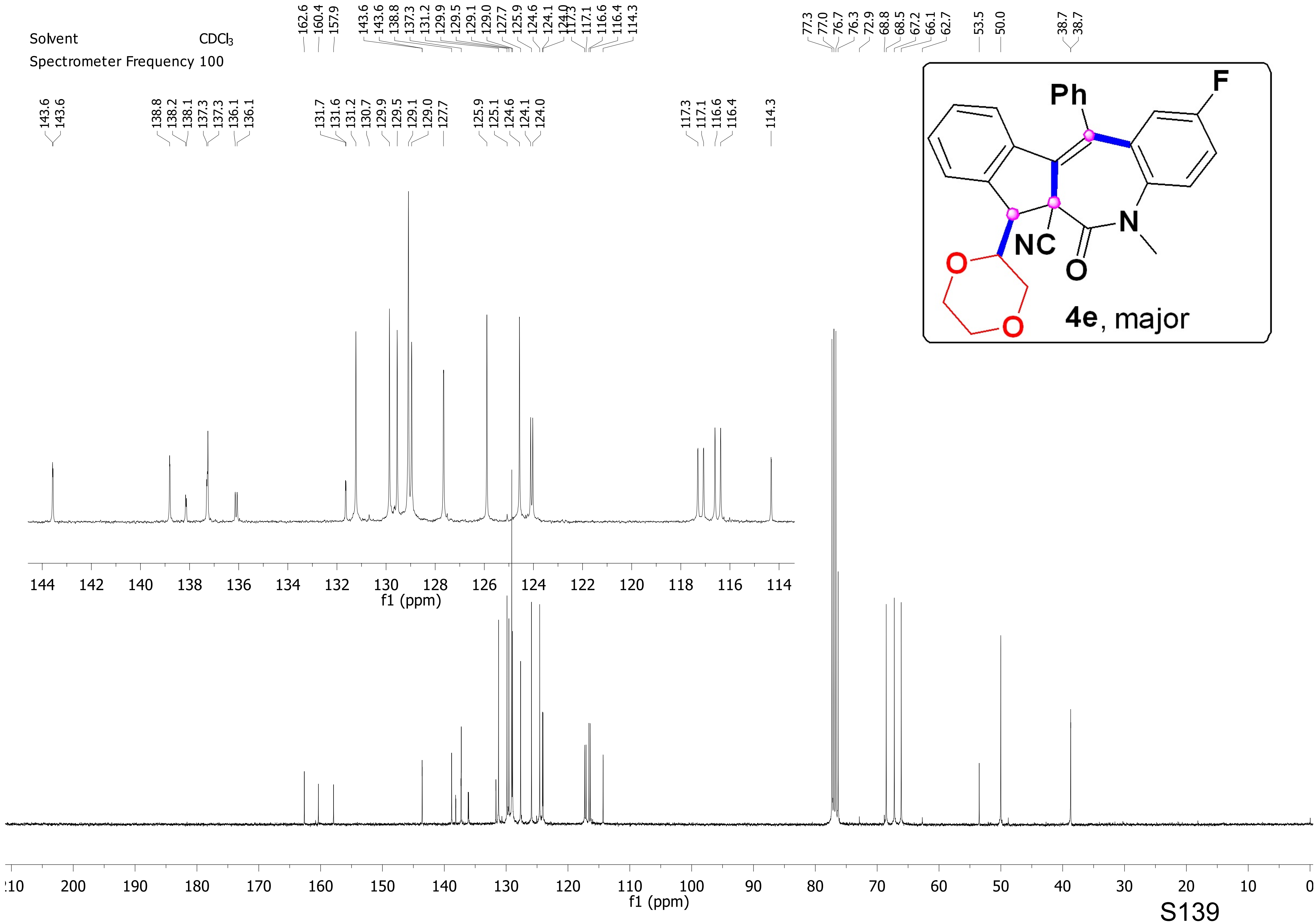

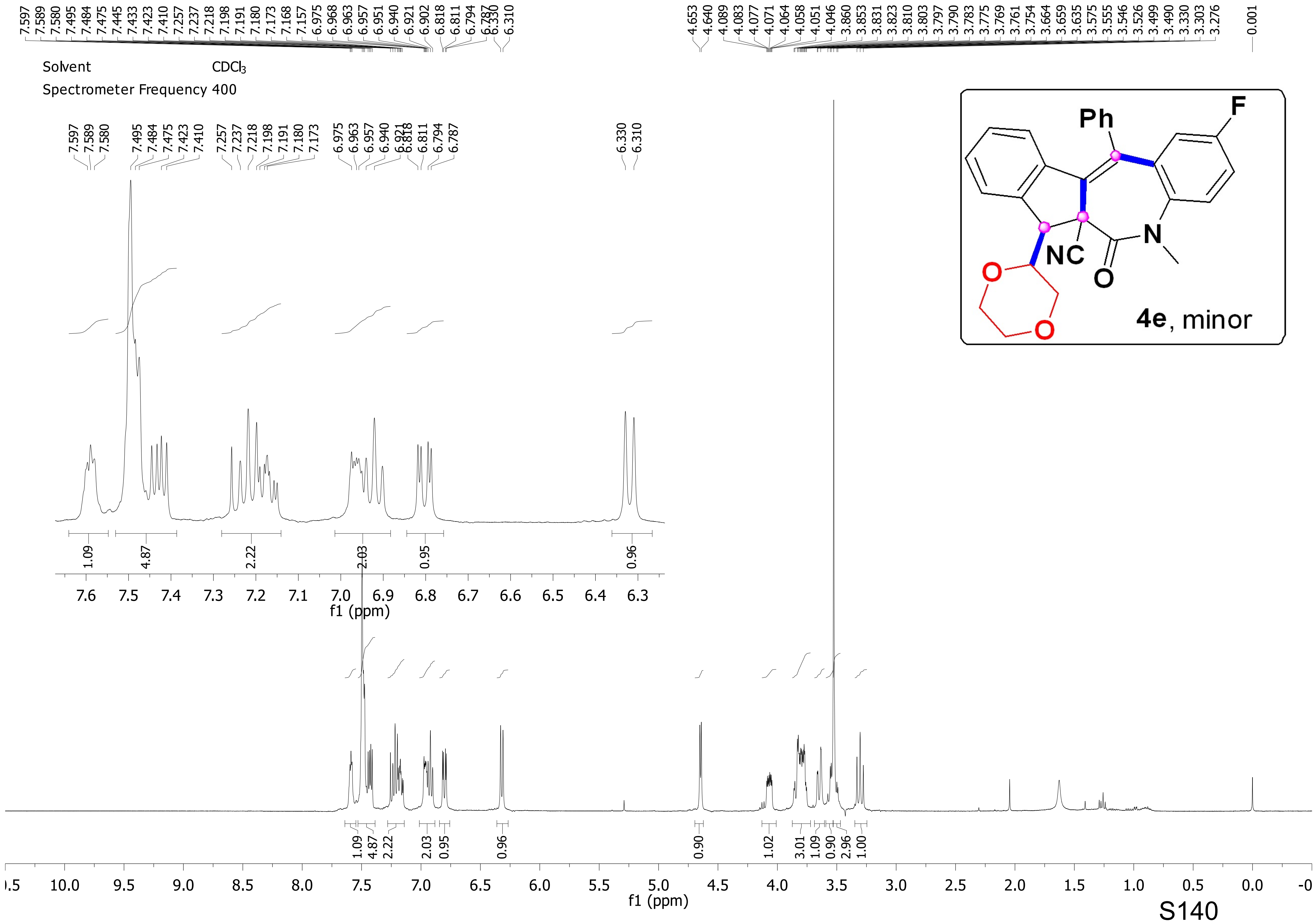
7.597 7.589 7.580 7.495 7.484 7.475 7.445 7.433 7.423 7.410 7.257 7.237 7.218 7.198 7.191 7.180 7.173 7.168 7.157 6.975 6.968 6.963 6.957 6.951 6.940 6.921 6.902 6.818 6.811 6.794 6.787 6.330 6.310
4.653 4.640 4.089 4.083 4.077 4.071 4.064 4.058 4.051 4.046 3.860 3.853 3.831 3.823 3.810 3.803 3.797 3.790 3.783 3.775 3.769 3.761 3.754 3.664 3.659 3.635 3.575 3.555 3.546 3.526 3.499 3.490 3.330 3.303 3.276
0.001
Solvent CDCl3
Spectrometer Frequency 400
Ph
F
N
NC
O
4e, minor
1.09 4.87 2.22 2.03 0.95 0.96
f1 (ppm)
1.09 4.87 2.22 2.03 0.95 0.96 0.90 1.02 3.01 1.09 0.90 2.96 1.00
f1 (ppm)

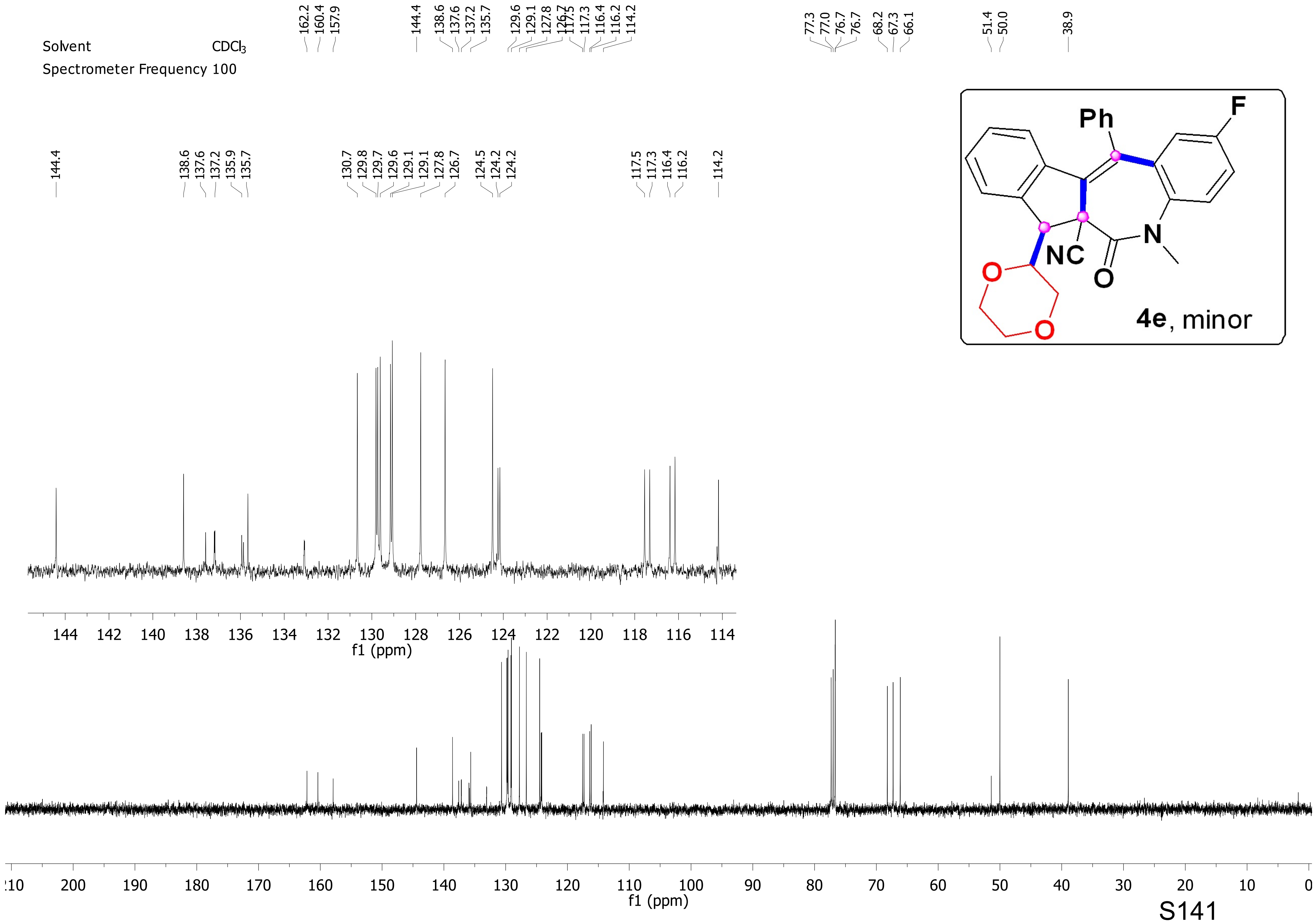
Solvent CDCl3
Spectrometer Frequency 100
162.2 160.4 157.9 144.4 138.6 137.6 137.2 135.7 129.6 129.1 127.8 126.7 117.5 117.3 116.4 116.2 114.2 77.3 77.0 76.7 76.7 68.2 67.3 66.1 51.4 50.0 38.9
144.4 138.6 137.6 137.2 135.9 135.7 130.7 129.8 129.7 129.6 129.1 129.1 127.8 126.7 124.5 124.2 124.2 117.5 117.3 116.4 116.2 114.2
Ph
F
NC
N
O
4e, minor
f1 (ppm)
f1 (ppm)

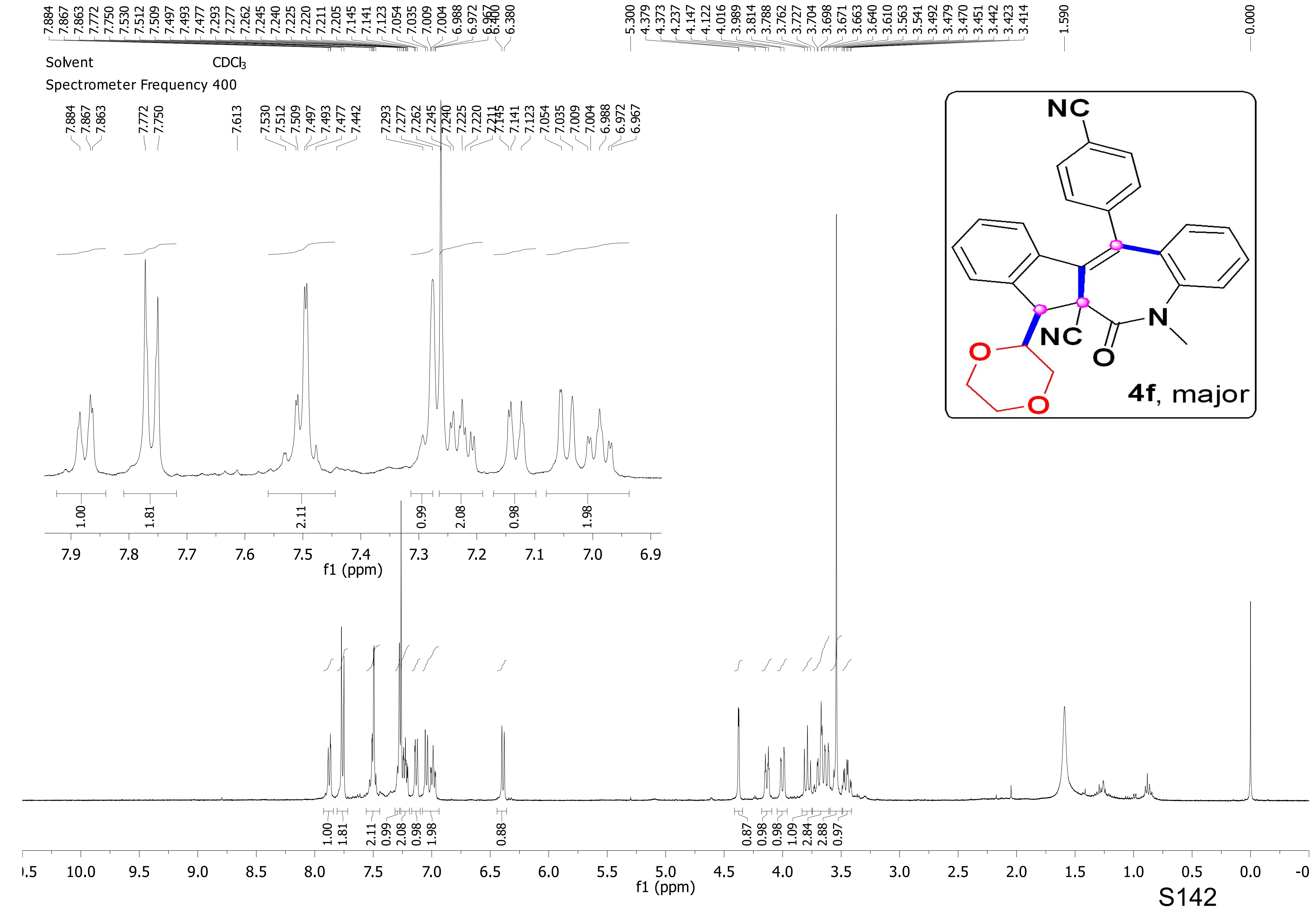
7.884 7.867 7.863 7.772 7.750 7.530 7.512 7.509 7.497 7.493 7.477 7.293 7.277 7.262 7.245 7.240 7.225 7.220 7.211 7.205 7.145 7.141 7.123 7.054 7.035 7.009 7.004 6.988 6.972 6.967 6.400 6.380
5.300 4.379 4.373 4.237 4.147 4.122 4.016 3.989 3.814 3.788 3.762 3.727 3.704 3.698 3.671 3.663 3.640 3.610 3.563 3.541 3.492 3.479 3.470 3.451 3.442 3.423 3.414
1.590
0.000
Solvent CDCl3
Spectrometer Frequency 400
7.884 7.867 7.863 7.772 7.750 7.613 7.530 7.512 7.509 7.497 7.493 7.477 7.442 7.293 7.277 7.262 7.245 7.240 7.225 7.220 7.211 7.145 7.141 7.123 7.054 7.035 7.009 7.004 6.988 6.972 6.967
1.00 1.81 2.11 0.99 2.08 0.98 1.98
7.9 7.8 7.7 7.6 7.5 7.4 7.3 7.2 7.1 7.0 6.9
f1 (ppm)
NC
NC
N
O
O
O
4f, major
1.00 1.81 2.11 0.99 2.08 0.98 1.98 0.88 0.87 0.98 0.98 1.09 2.84 2.88 0.97
10.0 9.5 9.0 8.5 8.0 7.5 7.0 6.5 6.0 5.5 5.0 4.5 4.0 3.5 3.0 2.5 2.0 1.5 1.0 0.5 0.0
f1 (ppm)

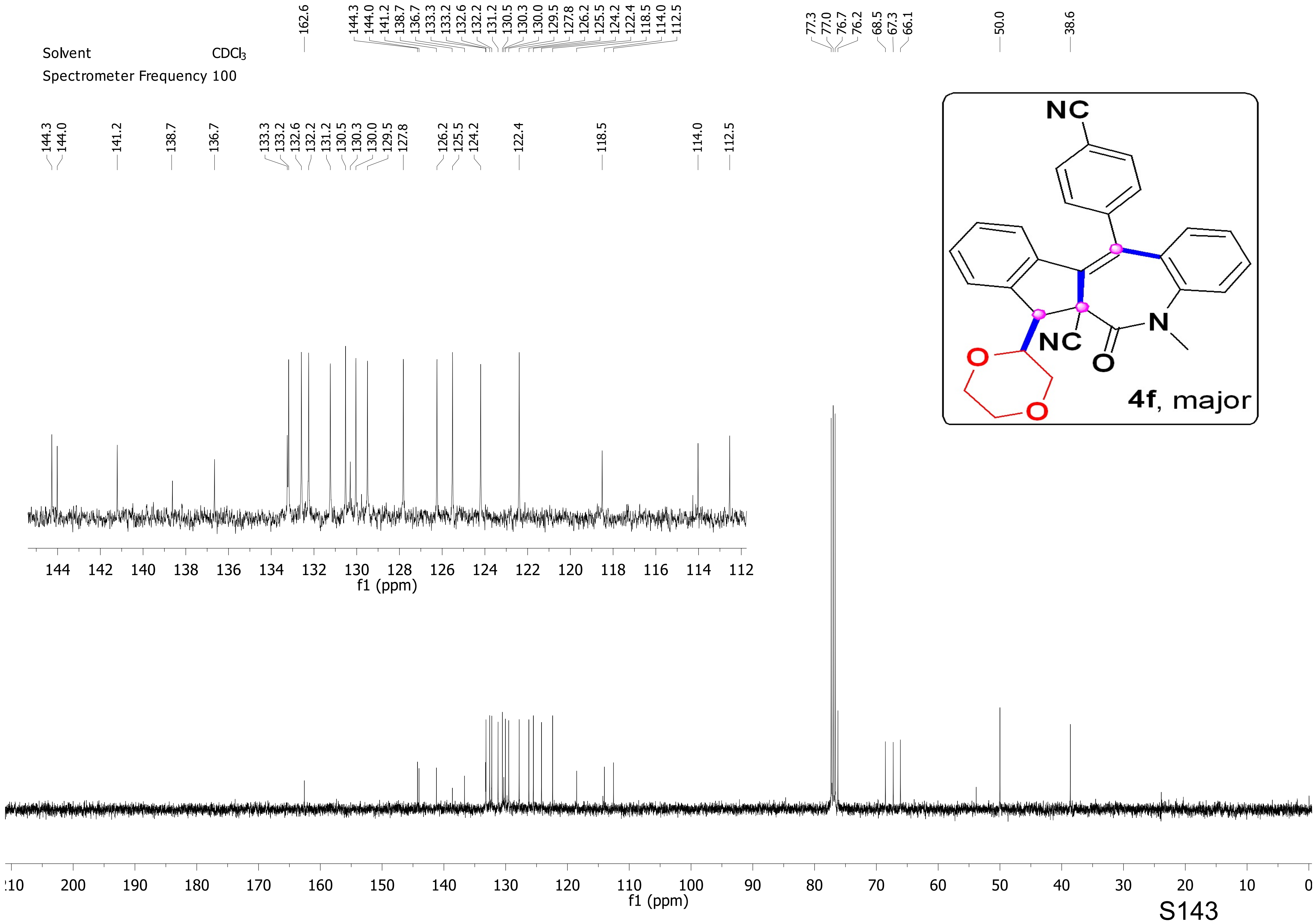
Solvent CDCl$_3$
Spectrometer Frequency 100
162.6
144.3 144.0 141.2 138.7 136.7 133.3 133.2 132.6 132.2 131.2 130.5 130.3 130.0 129.5 127.8 126.2 125.5 124.2 122.4 118.5 114.0 112.5
77.3 77.0 76.7 76.2 68.5 67.3 66.1
50.0
38.6
NC
N
NC
O
O
O
4f, major
f1 (ppm)
f1 (ppm)

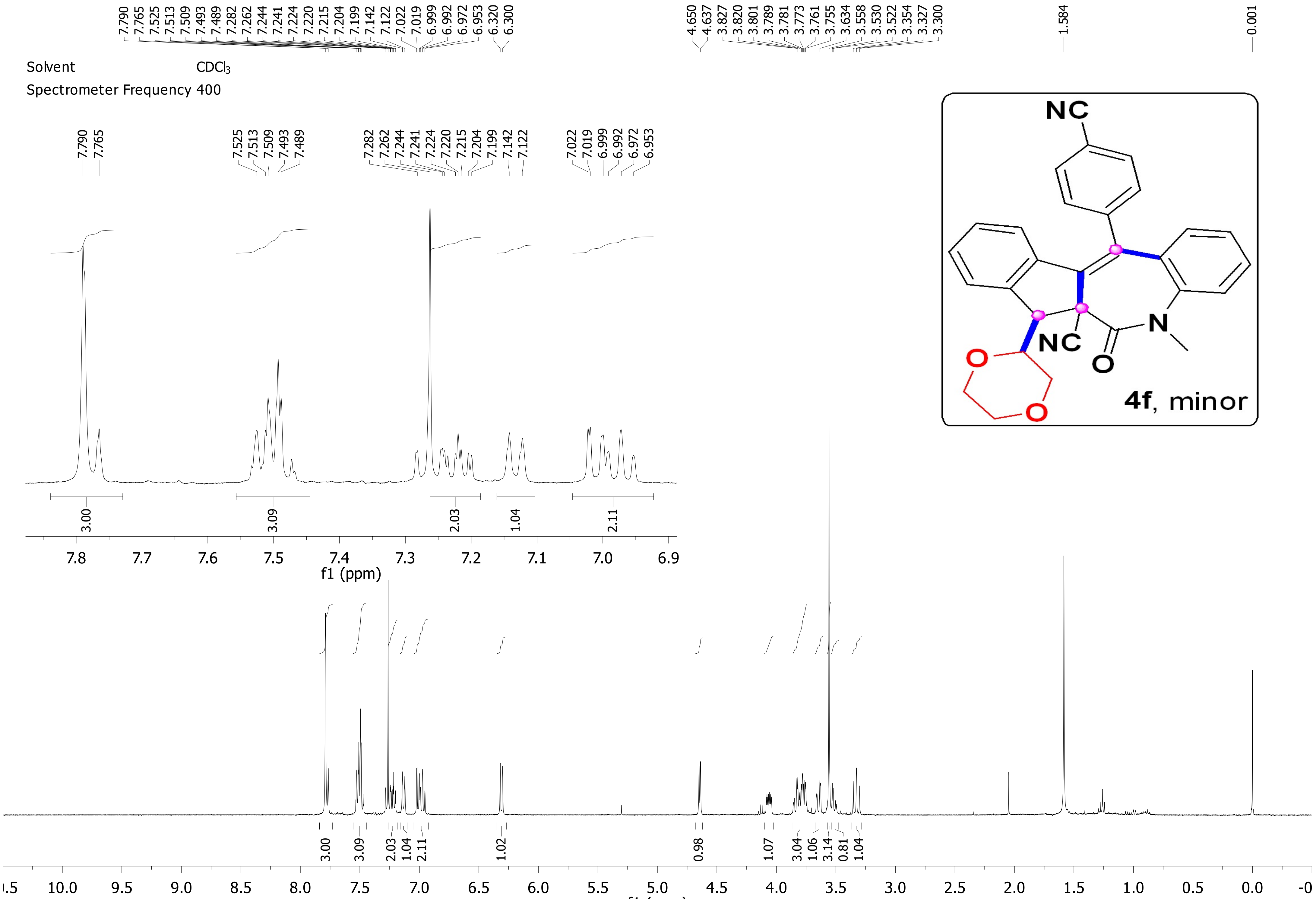
7.790 7.765 7.525 7.513 7.509 7.493 7.489 7.282 7.262 7.244 7.241 7.224 7.220 7.215 7.204 7.199 7.142 7.122 7.022 7.019 6.999 6.992 6.972 6.953 6.320 6.300
4.650 4.637 3.827 3.820 3.801 3.789 3.781 3.773 3.761 3.755 3.634 3.558 3.530 3.522 3.354 3.327 3.300
1.584
0.001
Solvent CDCl3
Spectrometer Frequency 400
NC
NC
N
O
O
O
4f, minor
3.00 3.09 2.03 1.04 2.11
f1 (ppm)
3.00 3.09 2.03 1.04 2.11 1.02 0.98 1.07 3.04 1.06 3.14 0.81 1.04
f1 (ppm)

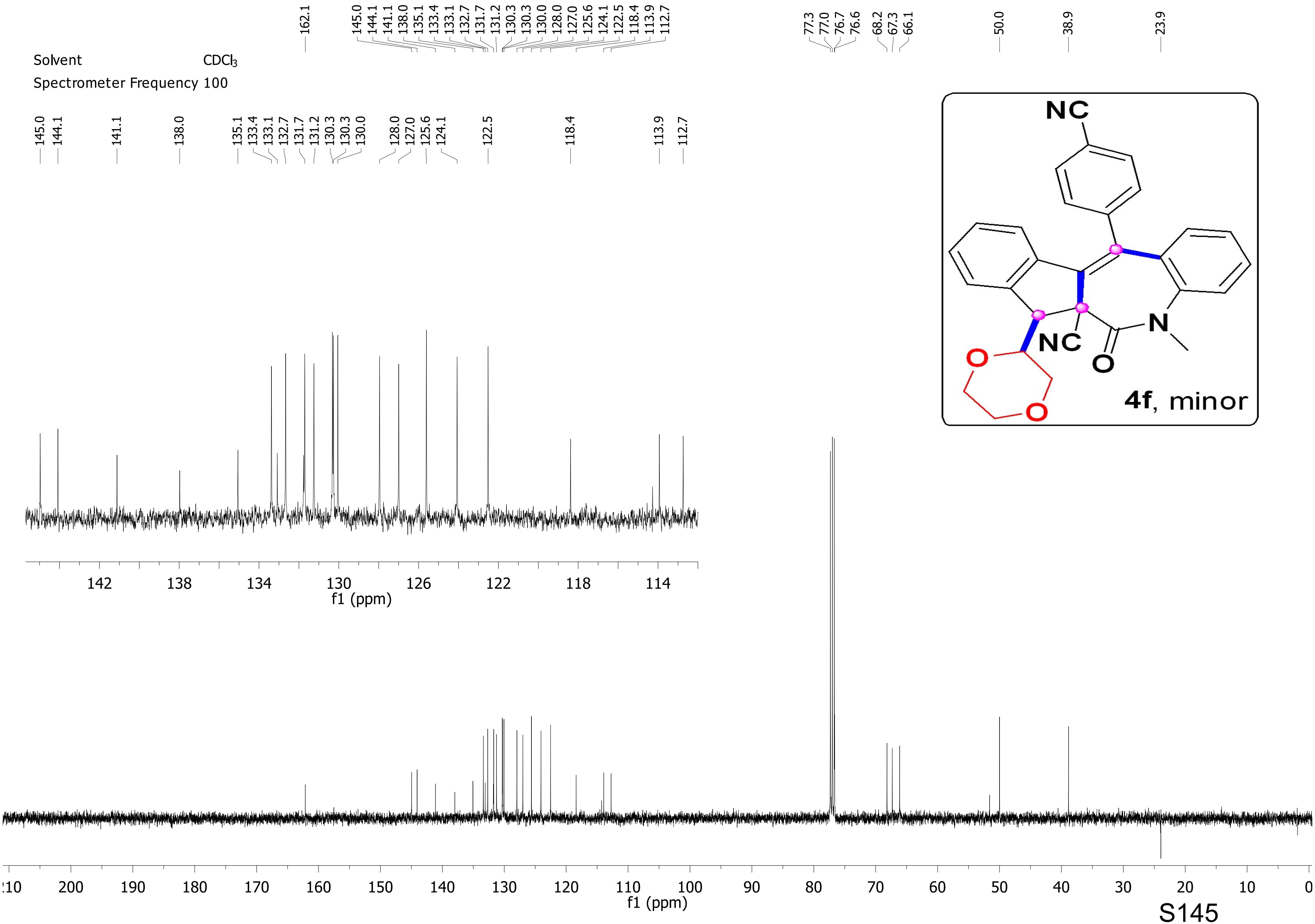
Solvent CDCl$_3$
Spectrometer Frequency 100
162.1
145.0 144.1 141.1 138.0 135.1 133.4 133.1 132.7 131.7 131.2 130.3 130.3 130.0 128.0 127.0 125.6 124.1 122.5 118.4 113.9 112.7
77.3 77.0 76.7 76.6 68.2 67.3 66.1
50.0
38.9
23.9
NC
NC
N
O
O
O
4f, minor
142 138 134 130 126 122 118 114
f1 (ppm)
210 200 190 180 170 160 150 140 130 120 110 100 90 80 70 60 50 40 30 20 10 0
f1 (ppm)

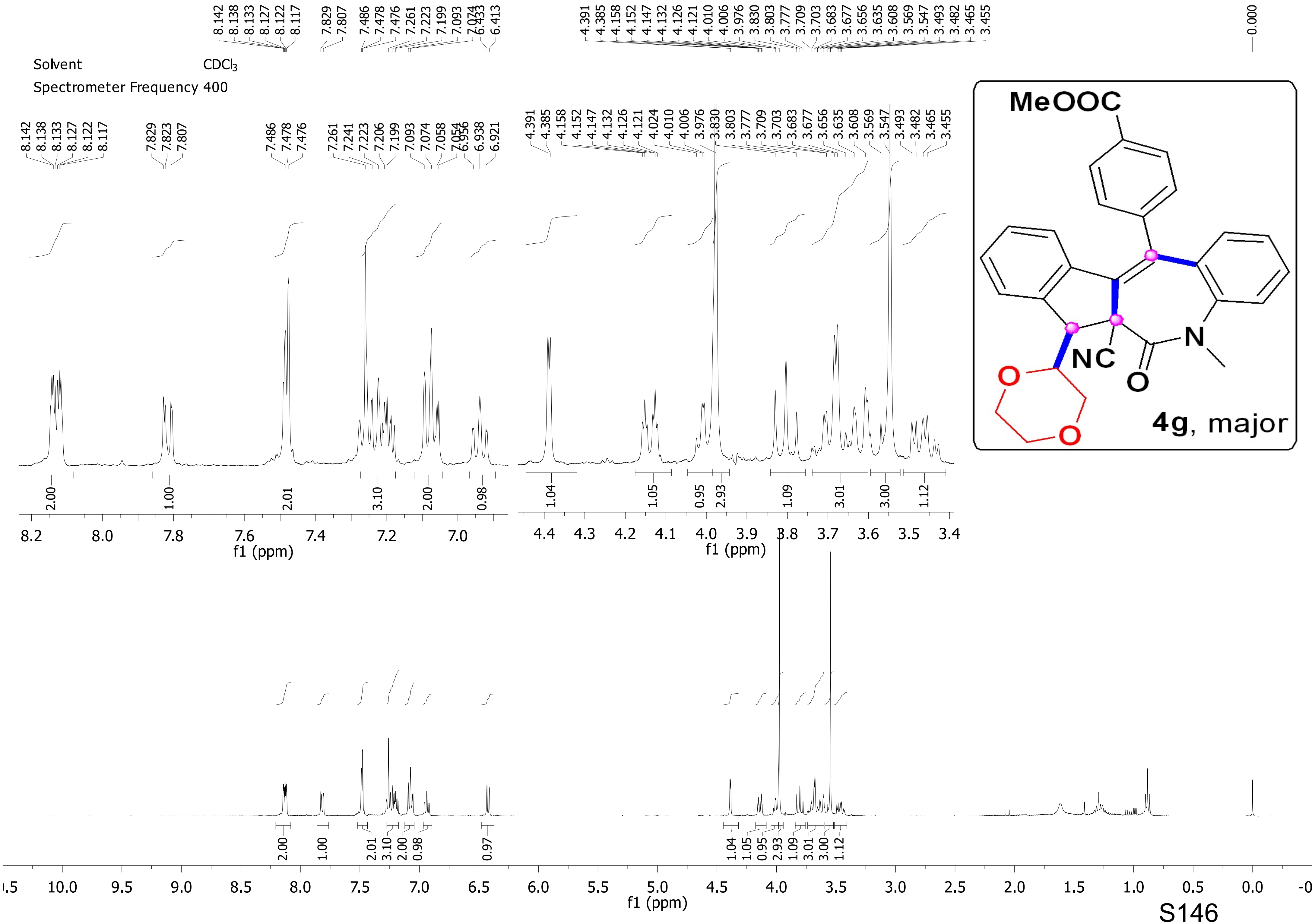
Solvent CDCl$_3$
Spectrometer Frequency 400
MeOOC
NC
N
O
O
O
4g, major
f1 (ppm)

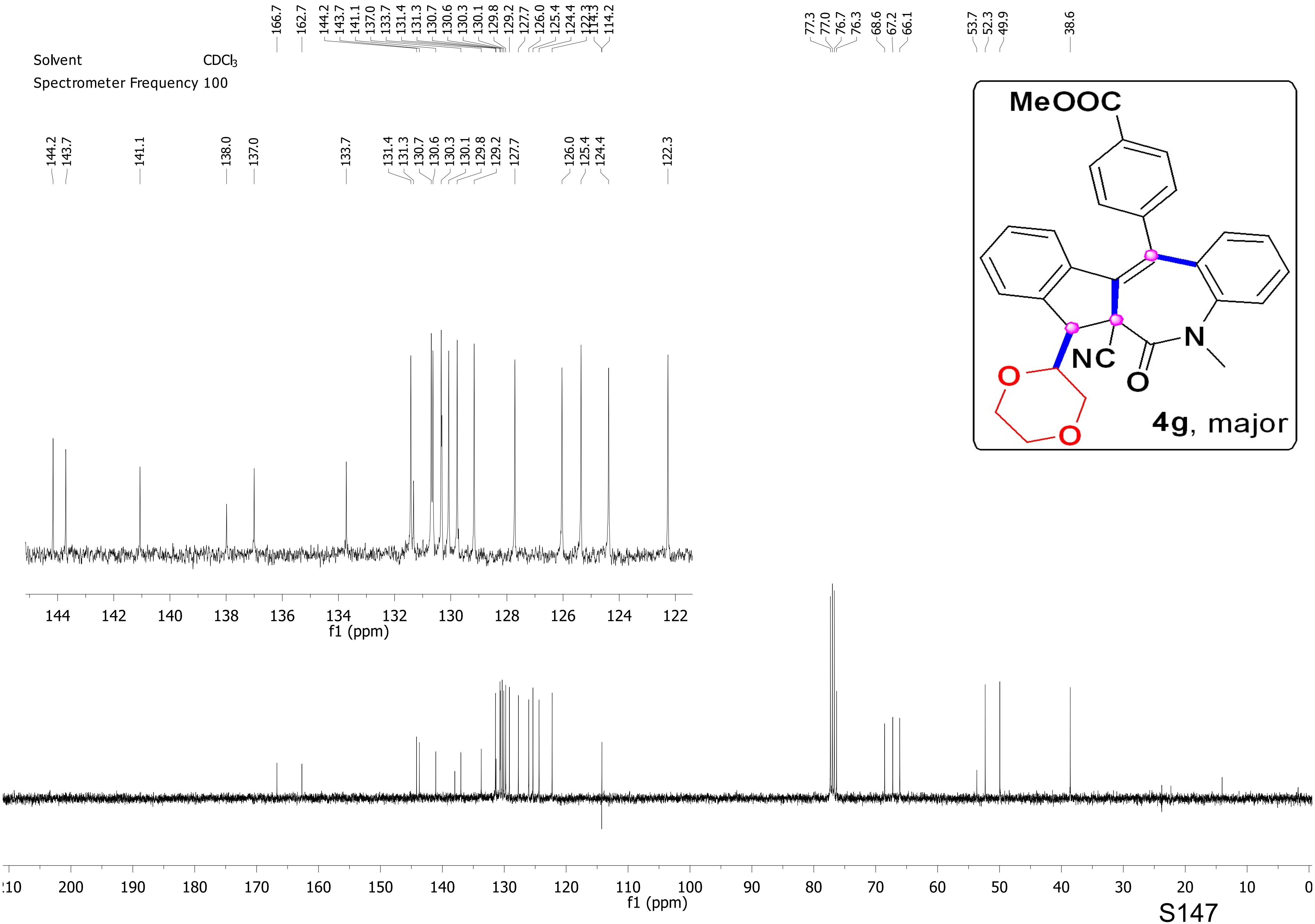
Solvent CDCl3
Spectrometer Frequency 100
166.7
162.7
144.2
143.7
141.1
137.0
133.7
131.4
131.3
130.7
130.6
130.3
130.1
129.8
129.2
127.7
126.0
125.4
124.4
122.3
114.3
114.2
77.3
77.0
76.7
76.3
68.6
67.2
66.1
53.7
52.3
49.9
38.6
144.2
143.7
141.1
138.0
137.0
133.7
131.4
131.3
130.7
130.6
130.3
130.1
129.8
129.2
127.7
126.0
125.4
124.4
122.3
f1 (ppm)
f1 (ppm)
MeOOC
NC
N
O
O
O
4g, major

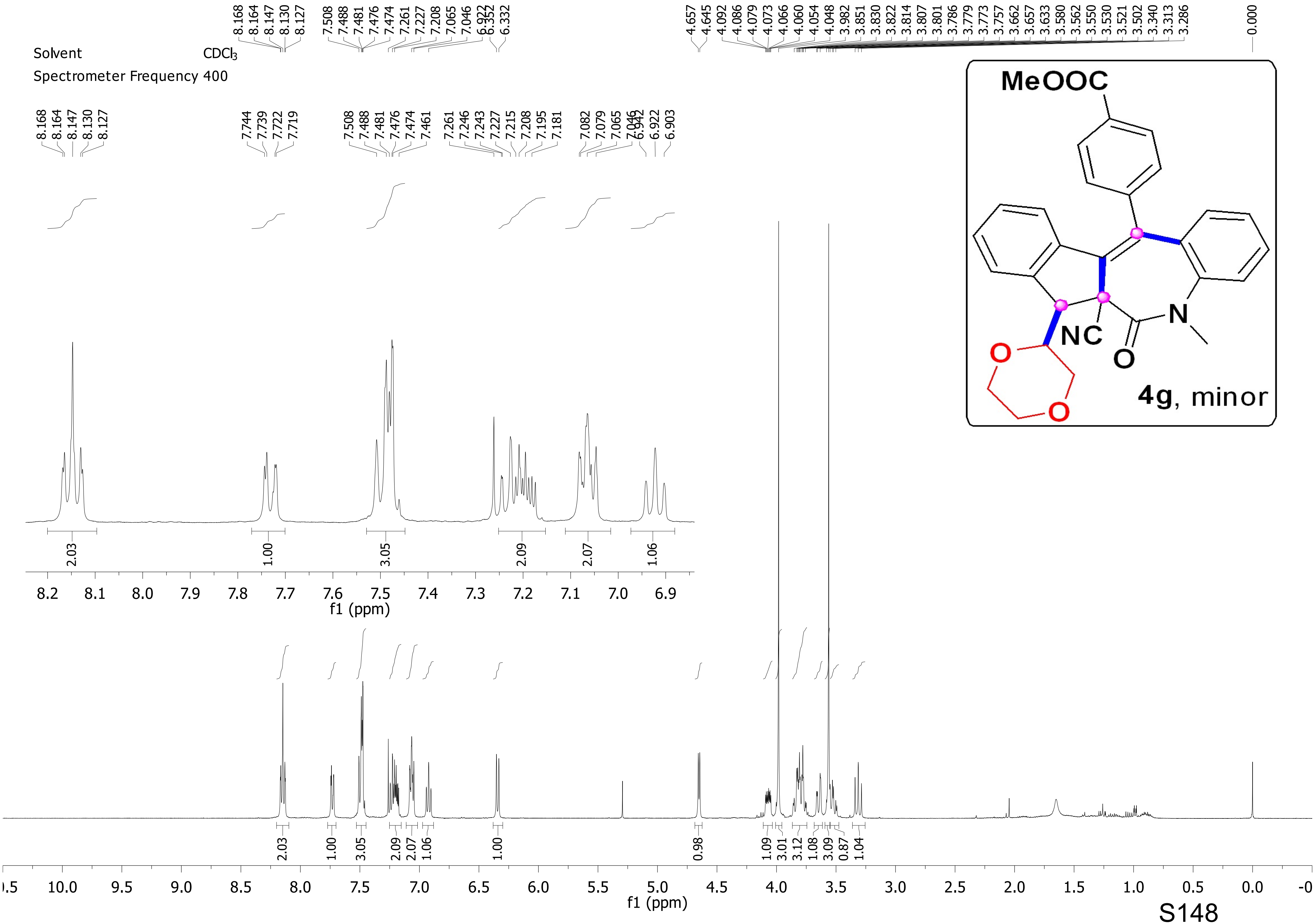
Solvent CDCl3
Spectrometer Frequency 400
f1 (ppm)
f1 (ppm)
MeOOC
NC
N
O
O
O

4g, minor

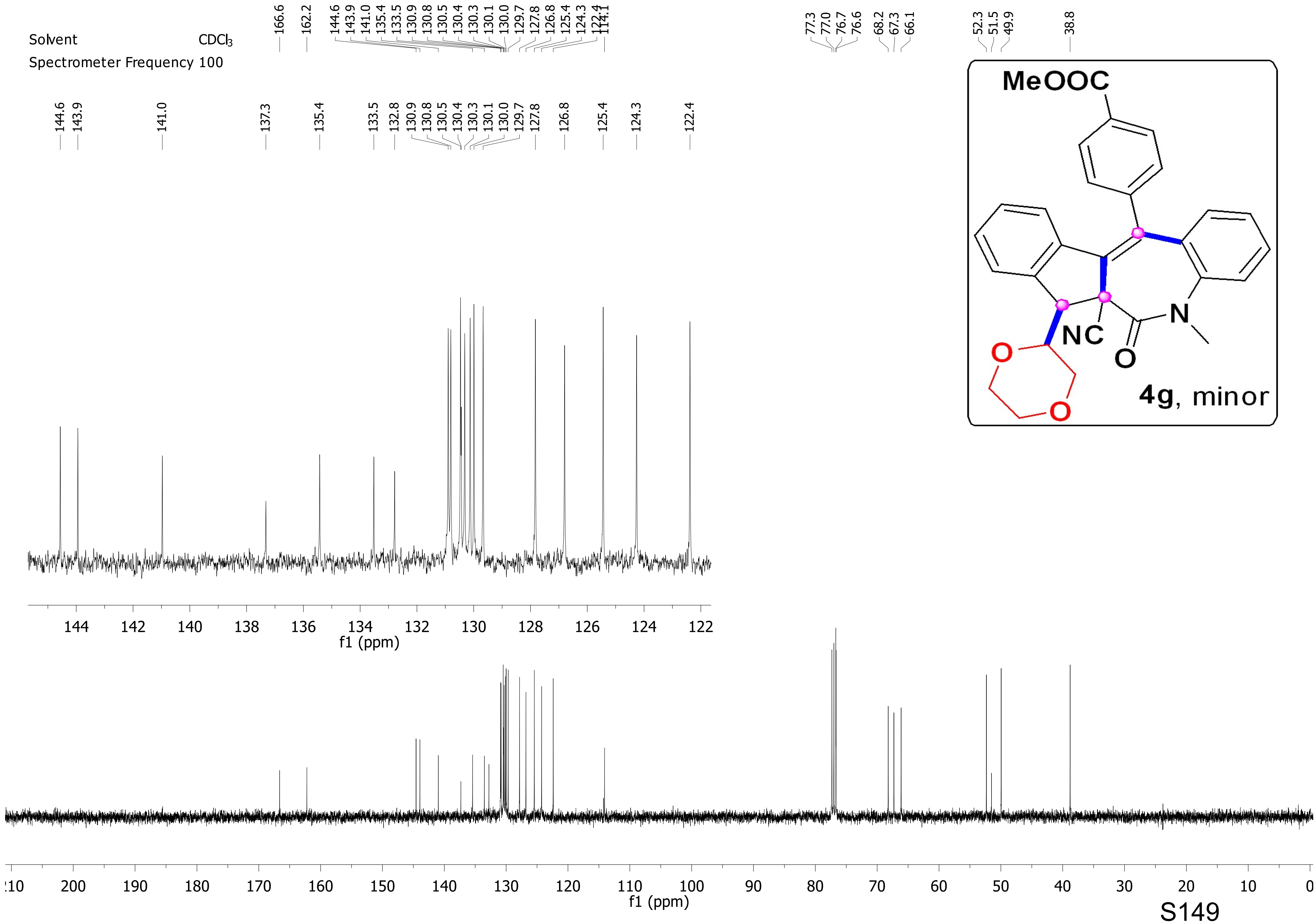
Solvent CDCl$_3$
Spectrometer Frequency 100
166.6 162.2 144.6 143.9 141.0 135.4 133.5 130.9 130.8 130.5 130.4 130.3 130.1 130.0 129.7 127.8 126.8 125.4 124.3 122.4 114.1
77.3 77.0 76.7 76.6 68.2 67.3 66.1 52.3 51.5 49.9 38.8
144.6 143.9 141.0 137.3 135.4 133.5 132.8 130.9 130.8 130.5 130.4 130.3 130.1 130.0 129.7 127.8 126.8 125.4 124.3 122.4
f1 (ppm)
f1 (ppm)
MeOOC
NC
N
O
O
O
4g, minor

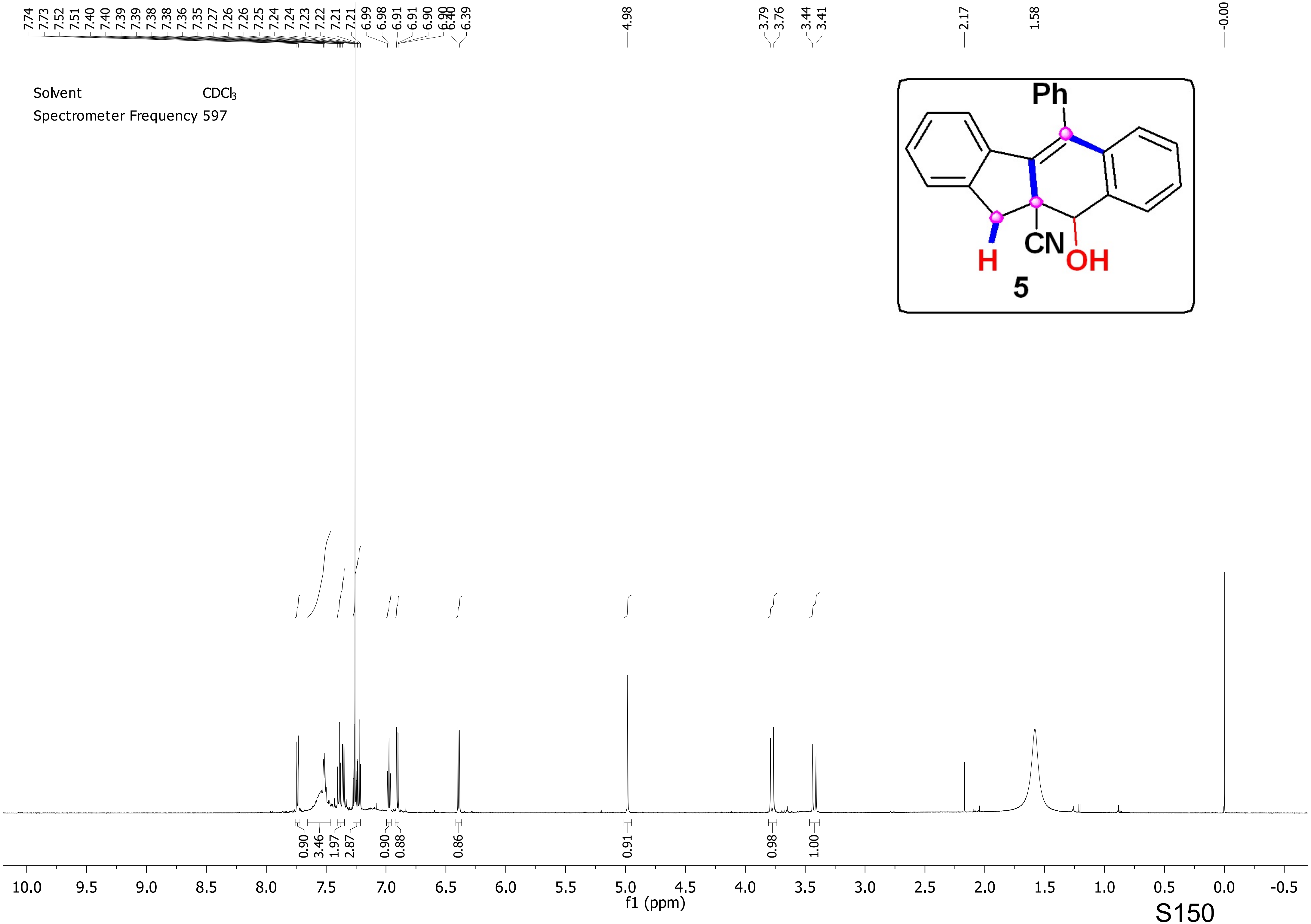
7.74
7.73
7.52
7.51
7.40
7.40
7.39
7.39
7.38
7.38
7.36
7.35
7.27
7.26
7.26
7.25
7.24
7.24
7.23
7.22
7.21
7.21
6.99
6.98
6.91
6.91
6.90
6.90
6.40
6.39
4.98
3.79
3.76
3.44
3.41
2.17
1.58
-0.00
Solvent CDCl3
Spectrometer Frequency 597
Ph
CN
H
OH
5
0.90
3.46
1.97
2.87
0.90
0.88
0.86
0.91
0.98
1.00
10.0
9.5
9.0
8.5
8.0
7.5
7.0
6.5
6.0
5.5
5.0
4.5
4.0
3.5
3.0
2.5
2.0
1.5
1.0
0.5
0.0
-0.5
f1 (ppm)

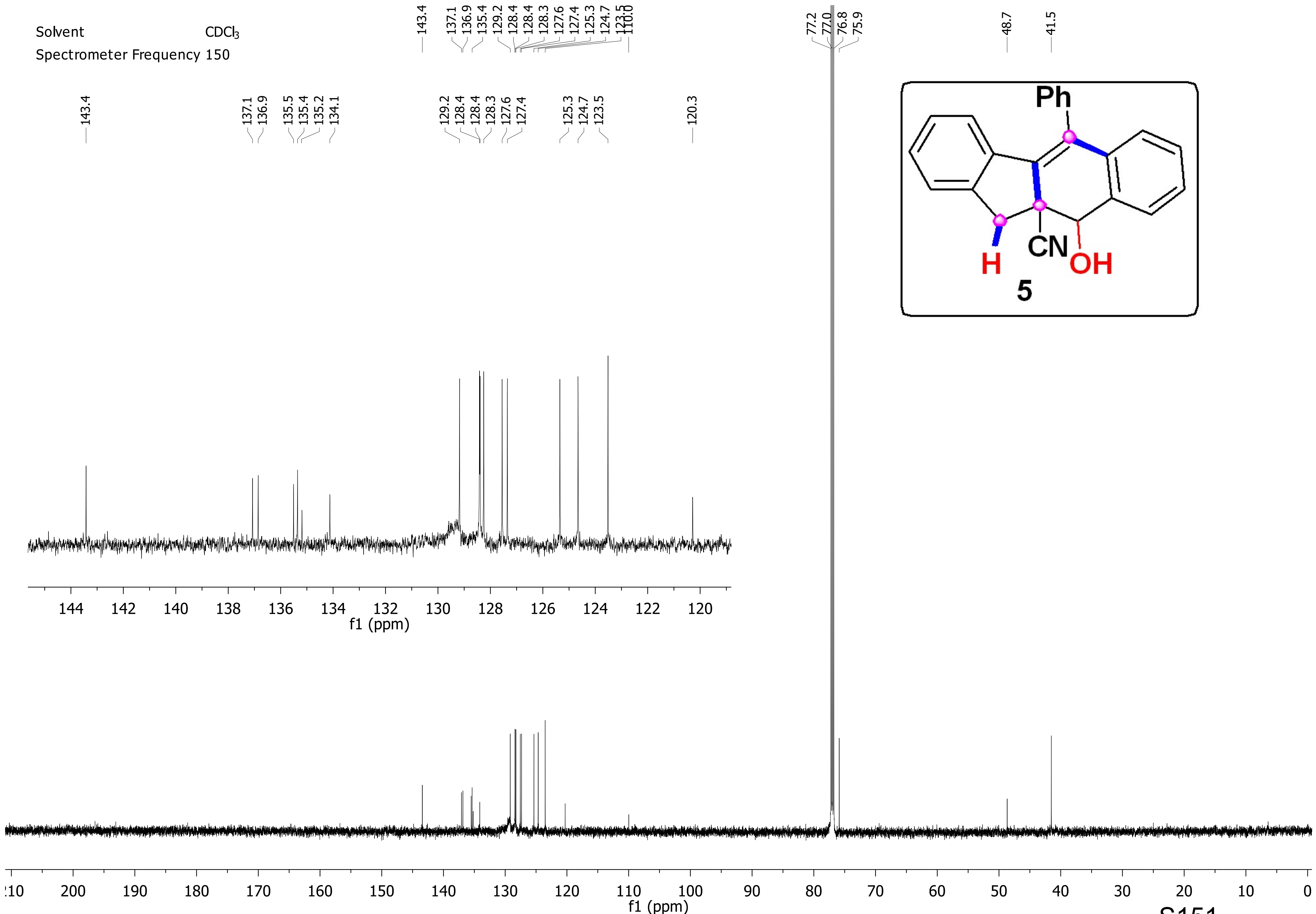
Solvent CDCl3
Spectrometer Frequency 150
143.4 137.1 136.9 135.4 129.2 128.4 128.4 128.3 127.6 127.4 125.3 124.7 123.5 110.0
77.2 77.0 76.8 75.9
48.7
41.5
143.4
137.1 136.9
135.5 135.4 135.2 134.1
129.2 128.4 128.4 128.3 127.6 127.4
125.3 124.7 123.5
120.3
Ph
CN
H
OH
5
f1 (ppm)
f1 (ppm)

TPG -620_20231206030400 #17 RT: 0.12 AV: 1 NL: 8.74E7
T: FTMS + p ESI sid=35.00 Full ms [100.0000-1000.0000]

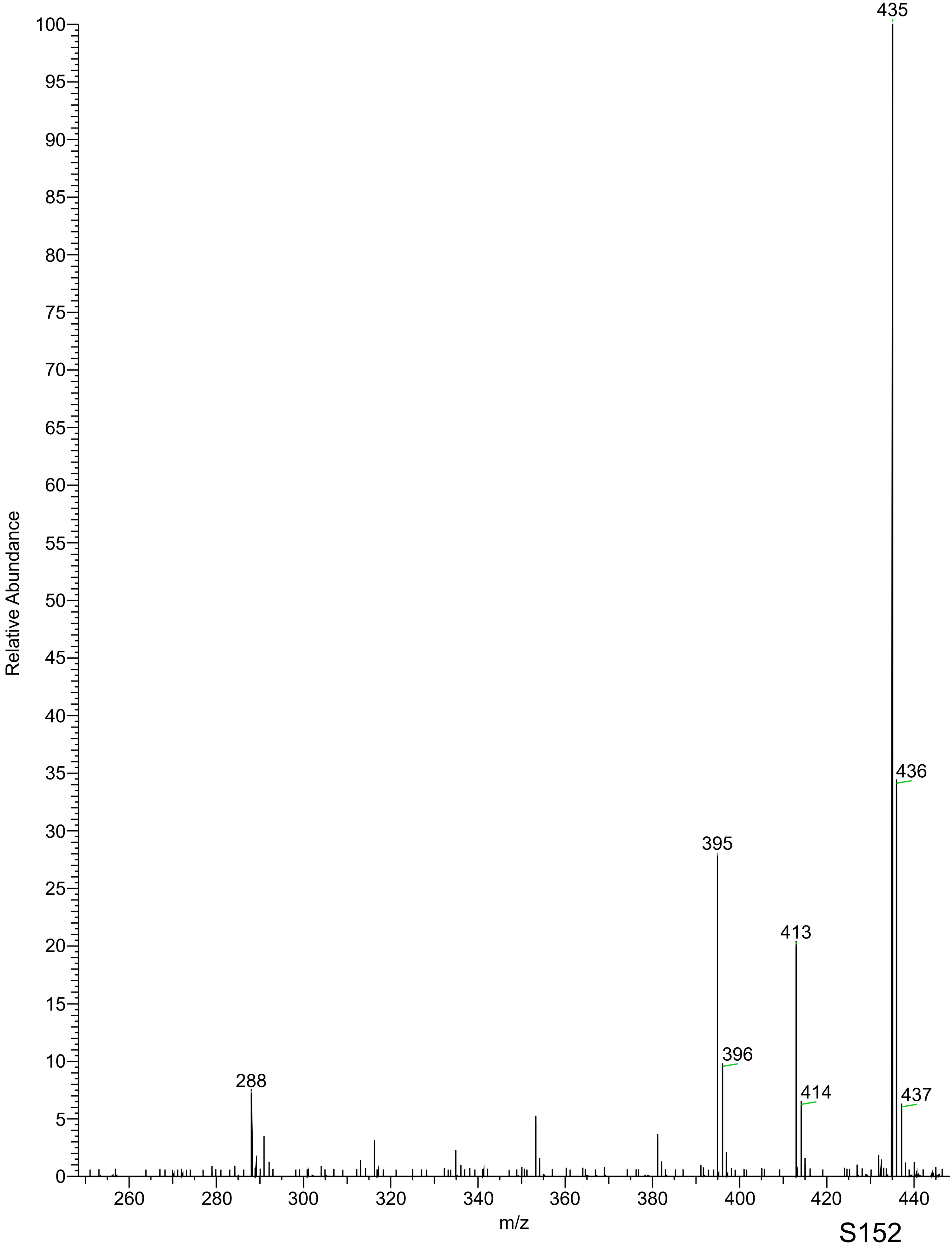

S152

TPG -621_20231206031517 #17 RT: 0.12 AV: 1 NL: 7.87E7
T: FTMS + p ESI Full ms [100.0000-1000.0000]

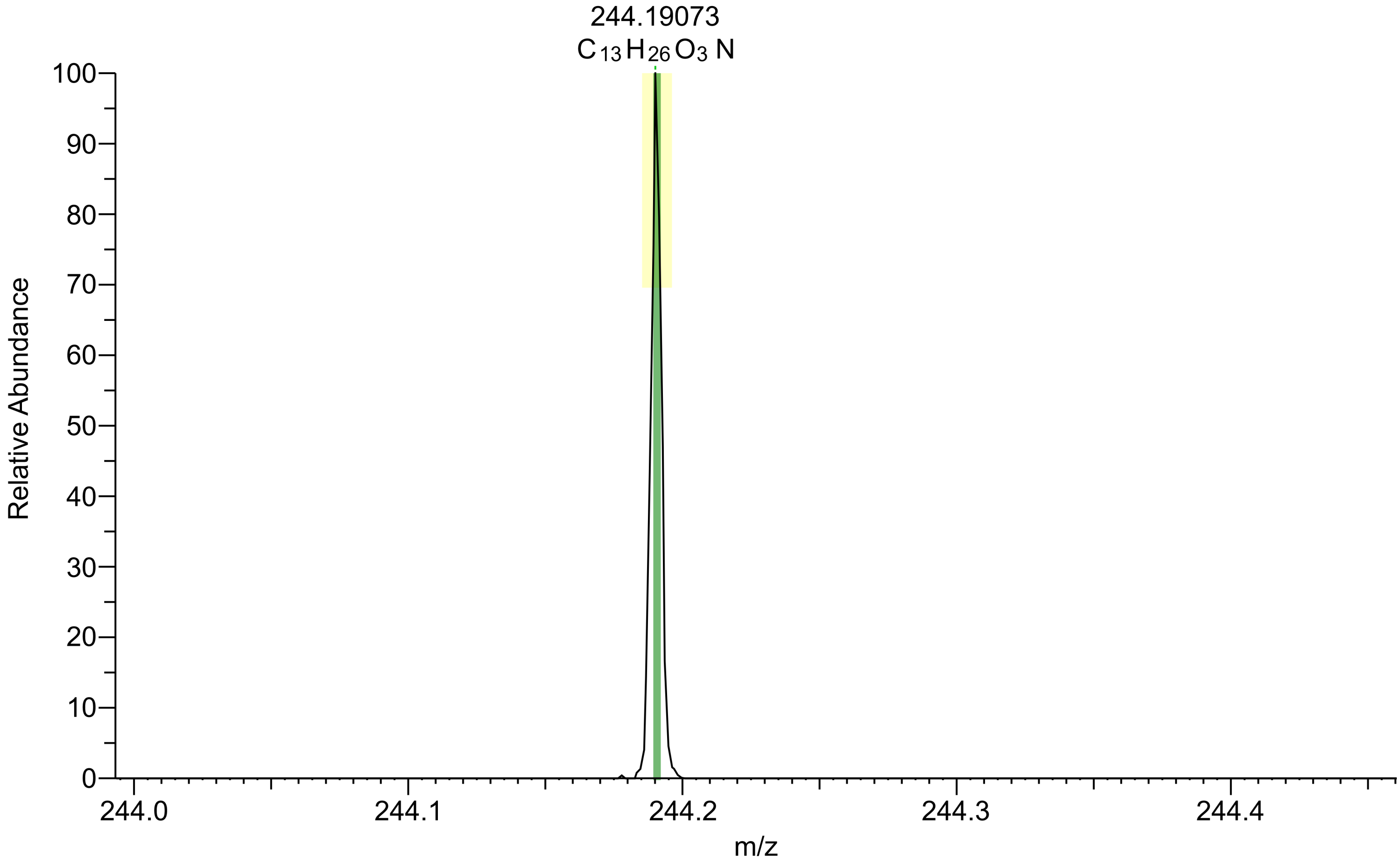

Peak Mass	Display Formula	S Fit	RDB	Delta [ppm]	Theo. mass	Pattern Cov. [%]	MSMS Matched...
244.19073	$C_{13}H_{26}O_3N$	47.2379119351581	1.50	0.06	244.19072	99.91	(Collection)

checkCIF/PLATON report

Structure factors have been supplied for datablock(s) tpg36

THIS REPORT IS FOR GUIDANCE ONLY. IF USED AS PART OF A REVIEW PROCEDURE FOR PUBLICATION, IT SHOULD NOT REPLACE THE EXPERTISE OF AN EXPERIENCED CRYSTALLOGRAPHIC REFEREE.

No syntax errors found. CIF dictionary Interpreting this report

Datablock: tpg36

Bond precision:	C-C = 0.0066 A		Wavelength=0.71073
Cell:	a=11.0983(10)	b=13.3820(11)	c=14.7589(11)
	alpha=90	beta=104.129(3)	gamma=90
Temperature:	292 K		

	Calculated	Reported
Volume	2125.6(3)	2125.6(3)
Space group	P 21/n	P 21/n
Hall group	-P 2yn	-P 2yn
Moiety formula	C28 H21 N O3	C28 H21 N O3
Sum formula	C28 H21 N O3	C28 H21 N O3
Mr	419.46	419.46
Dx,g cm-3	1.311	1.311
Z	4	4
Mu (mm-1)	0.085	0.085
F000	880.0	880.0
F000'	880.40	
h,k,lmax	13,16,18	13,16,18
Nref	4364	4348
Tmin,Tmax	0.961,0.975	0.819,0.928
Tmin'	0.950	

Correction method= # Reported T Limits: Tmin=0.819 Tmax=0.928
AbsCorr = MULTI-SCAN

Data completeness= 0.996 Theta(max)= 26.409

R(reflections)= 0.0933(3108) wR2(reflections)= 0.2495(4348)

S = 1.051 Npar= 289

The following ALERTS were generated. Each ALERT has the format
test-name_ALERT_alert-type_alert-level.
Click on the hyperlinks for more details of the test.

Alert level A

```
SYMMG01_ALERT_1_A          _symmetry_space_group_number does not match H-M symbol
           From the CIF: _space_group_IT_number
           From the CIF: _symmetry_space_group_name_H-M      P2/n
           Int. Tables space group number for P2/n is  13
SYMMG02_ALERT_1_A        The _symmetry_equiv_pos_as_xyz values are inconsistent
                     with the H-M symbol given
           From the CIF: _symmetry_equiv_pos_as_xyz
                                  x, y, z
                                  -x+1/2, y+1/2, -z+1/2
                                  -x, -y, -z
                                  x-1/2, -y-1/2, z-1/2
           These symops generate the Hall space group symbol   -p_2yn
           From the CIF: _symmetry_space_group_name_H-M     p2/n
           From the xyz: _symmetry_space_group_name_H-M     p_1_21/n_1
```

Alert level C

```
PLAT340_ALERT_3_C Low Bond Precision on  C-C Bonds ...............    0.00658 Ang.
PLAT906_ALERT_3_C Large K Value in the Analysis of Variance ......      9.144 Check
PLAT910_ALERT_3_C Missing # of FCF Reflection(s) Below Theta(Min).          5 Note
PLAT911_ALERT_3_C Missing FCF Refl Between Thmin & STh/L=    0.600          6 Report
```

Alert level G

```
PLAT083_ALERT_2_G SHELXL Second Parameter in WGHT  Unusually Large       7.33 Why ?
PLAT398_ALERT_2_G Deviating  C-O-C   Angle From 120 for O3        .     108.6 Degree
PLAT793_ALERT_4_G Model has Chirality at C1          (Centro SPGR)          S Verify
PLAT793_ALERT_4_G Model has Chirality at C17         (Centro SPGR)          R Verify
PLAT793_ALERT_4_G Model has Chirality at C18         (Centro SPGR)          R Verify
PLAT883_ALERT_1_G No Info/Value for _atom_sites_solution_primary .    Please Do !
PLAT898_ALERT_4_G Second Reported H-M Symbol in CIF Ignored ......          ! Check
PLAT912_ALERT_4_G Missing # of FCF Reflections Above STh/L=  0.600          6 Note
PLAT913_ALERT_3_G Missing # of Very Strong Reflections in FCF ....          1 Note
PLAT965_ALERT_2_G The SHELXL WEIGHT Optimisation has not Converged    Please Check
PLAT978_ALERT_2_G Number C-C Bonds with Positive Residual Density.          2 Info
```

2 **ALERT level A** = Most likely a serious problem - resolve or explain
0 **ALERT level B** = A potentially serious problem, consider carefully
4 **ALERT level C** = Check. Ensure it is not caused by an omission or oversight
11 **ALERT level G** = General information/check it is not something unexpected

3 ALERT type 1 CIF construction/syntax error, inconsistent or missing data
4 ALERT type 2 Indicator that the structure model may be wrong or deficient
5 ALERT type 3 Indicator that the structure quality may be low
5 ALERT type 4 Improvement, methodology, query or suggestion
0 ALERT type 5 Informative message, check

It is advisable to attempt to resolve as many as possible of the alerts in all categories. Often the minor alerts point to easily fixed oversights, errors and omissions in your CIF or refinement strategy, so attention to these fine details can be worthwhile. In order to resolve some of the more serious problems it may be necessary to carry out additional measurements or structure refinements. However, the purpose of your study may justify the reported deviations and the more serious of these should normally be commented upon in the discussion or experimental section of a paper or in the "special_details" fields of the CIF. checkCIF was carefully designed to identify outliers and unusual parameters, but every test has its limitations and alerts that are not important in a particular case may appear. Conversely, the absence of alerts does not guarantee there are no aspects of the results needing attention. It is up to the individual to critically assess their own results and, if necessary, seek expert advice.

Publication of your CIF in IUCr journals

A basic structural check has been run on your CIF. These basic checks will be run on all CIFs submitted for publication in IUCr journals (*Acta Crystallographica*, *Journal of Applied Crystallography*, *Journal of Synchrotron Radiation*); however, if you intend to submit to *Acta Crystallographica Section C* or *E* or *IUCrData*, you should make sure that full publication checks are run on the final version of your CIF prior to submission.

Publication of your CIF in other journals

Please refer to the *Notes for Authors* of the relevant journal for any special instructions relating to CIF submission.

Validation response form

Please find below a validation response form (VRF) that can be filled in and pasted into your CIF.

```
# start Validation Reply Form
_vrf_SYMMG01_tpg36
;
PROBLEM: _symmetry_space_group_number does not match H-M symbol
RESPONSE: ...
;
_vrf_SYMMG02_tpg36
;
PROBLEM: The _symmetry_equiv_pos_as_xyz values are inconsistent
RESPONSE: ...
;
_vrf_PLAT340_tpg36
;
PROBLEM: Low Bond Precision on  C-C Bonds ..............    0.00658 Ang.
RESPONSE: ...
;
_vrf_PLAT906_tpg36
;
PROBLEM: Large K Value in the Analysis of Variance ......      9.144 Check
RESPONSE: ...
;
_vrf_PLAT910_tpg36
```

```
;
PROBLEM: Missing # of FCF Reflection(s) Below Theta(Min).           5 Note
RESPONSE: ...
;
_vrf_PLAT911_tpg36
;
PROBLEM: Missing FCF Refl Between Thmin & STh/L=    0.600          6 Report
RESPONSE: ...
;
# end Validation Reply Form
```

PLATON version of 28/11/2022; check.def file version of 28/11/2022

Datablock tpg36 - ellipsoid plot

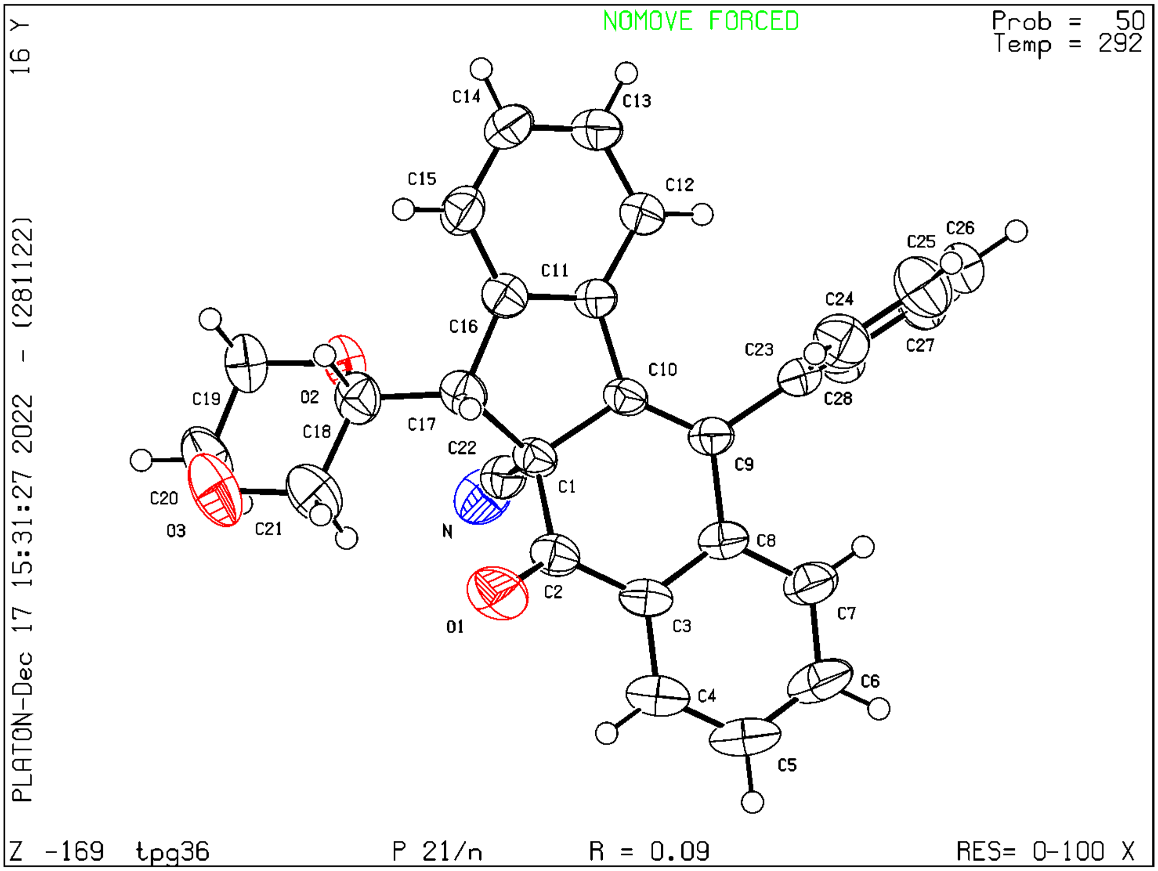

checkCIF/PLATON report

Structure factors have been supplied for datablock(s) I

THIS REPORT IS FOR GUIDANCE ONLY. IF USED AS PART OF A REVIEW PROCEDURE FOR PUBLICATION, IT SHOULD NOT REPLACE THE EXPERTISE OF AN EXPERIENCED CRYSTALLOGRAPHIC REFEREE.

No syntax errors found. CIF dictionary Interpreting this report

Datablock: I

```
Bond precision:  C-C = 0.0030 A          Wavelength=0.71073

Cell:           a=11.9063(5)    b=13.1181(5)       c=14.1751(6)
                alpha=90        beta=104.613(4)    gamma=90
Temperature:    113 K

                Calculated                Reported
Volume          2142.36(16)               2142.36(16)
Space group     P 21/n                    P 1 21/n 1
Hall group      -P 2yn                    -P 2yn
Moiety formula  C28 H21 N O3              C28 H21 N O3
Sum formula     C28 H21 N O3              C28 H21 N O3
Mr              419.46                    419.46
Dx,g cm-3       1.301                     1.300
Z               4                         4
Mu (mm-1)       0.084                     0.084
F000            880.0                     880.0
F000'           880.40
h,k,lmax        14,15,16                  14,15,16
Nref            3771                      3703
Tmin,Tmax       0.967,0.975               0.469,1.000
Tmin'           0.967

Correction method= # Reported T Limits: Tmin=0.469 Tmax=1.000
AbsCorr = MULTI-SCAN

Data completeness= 0.982          Theta(max)= 25.000

R(reflections)= 0.0515( 2990)     wR2(reflections)= 0.1213( 3703)

S = 1.039                  Npar= 289
```

The following ALERTS were generated. Each ALERT has the format
test-name_ALERT_alert-type_alert-level.
Click on the hyperlinks for more details of the test.

Alert level C

```
RINTA01_ALERT_3_C  The value of Rint is greater than 0.12
            Rint given   0.125
PLAT906_ALERT_3_C Large K Value in the Analysis of Variance ......      7.375 Check
PLAT911_ALERT_3_C Missing FCF Refl Between Thmin & STh/L=    0.595         67 Report
```

Alert level G

```
PLAT020_ALERT_3_G The Value of Rint is Greater Than 0.12 .........      0.125 Report
PLAT230_ALERT_2_G Hirshfeld Test Diff for    C1       --C18      .        6.2 s.u.
PLAT398_ALERT_2_G Deviating  C-O-C    Angle From 120 for O2             109.6 Degree
PLAT793_ALERT_4_G Model has Chirality at C1          (Centro SPGR)          S Verify
PLAT793_ALERT_4_G Model has Chirality at C17         (Centro SPGR)          R Verify
PLAT793_ALERT_4_G Model has Chirality at C25         (Centro SPGR)          S Verify
PLAT909_ALERT_3_G Percentage of I>2sig(I) Data at Theta(Max) Still        60% Note
PLAT910_ALERT_3_G Missing # of FCF Reflection(s) Below Theta(Min).          1 Note
PLAT933_ALERT_2_G Number of OMIT Records in Embedded .res File ...         68 Note
PLAT978_ALERT_2_G Number C-C Bonds with Positive Residual Density.          6 Info
```

```
   0 ALERT level A = Most likely a serious problem - resolve or explain
   0 ALERT level B = A potentially serious problem, consider carefully
   3 ALERT level C = Check. Ensure it is not caused by an omission or oversight
  10 ALERT level G = General information/check it is not something unexpected

   0 ALERT type 1 CIF construction/syntax error, inconsistent or missing data
   4 ALERT type 2 Indicator that the structure model may be wrong or deficient
   6 ALERT type 3 Indicator that the structure quality may be low
   3 ALERT type 4 Improvement, methodology, query or suggestion
   0 ALERT type 5 Informative message, check
```

checkCIF publication errors

Alert level A

```
PUBL004_ALERT_1_A  The contact author's name and address are missing,
            _publ_contact_author_name and _publ_contact_author_address.
PUBL005_ALERT_1_A  _publ_contact_author_email, _publ_contact_author_fax and
            _publ_contact_author_phone are all missing.
            At least one of these should be present.
PUBL006_ALERT_1_A  _publ_requested_journal is missing
            e.g. 'Acta Crystallographica Section C'
PUBL008_ALERT_1_A  _publ_section_title is missing. Title of paper.
PUBL009_ALERT_1_A  _publ_author_name is missing. List of author(s) name(s).
PUBL010_ALERT_1_A  _publ_author_address is missing. Author(s) address(es).
PUBL012_ALERT_1_A  _publ_section_abstract is missing.
            Abstract of paper in English.
```

```
   7 ALERT level A = Data missing that is essential or data in wrong format
   0 ALERT level G = General alerts. Data that may be required is missing
```

Publication of your CIF

You should attempt to resolve as many as possible of the alerts in all categories. Often the minor alerts point to easily fixed oversights, errors and omissions in your CIF or refinement strategy, so attention to these fine details can be worthwhile. In order to resolve some of the more serious problems it may be necessary to carry out additional measurements or structure refinements. However, the nature of your study may justify the reported deviations from journal submission requirements and the more serious of these should be commented upon in the discussion or experimental section of a paper or in the "special_details" fields of the CIF. *checkCIF* was carefully designed to identify outliers and unusual parameters, but every test has its limitations and alerts that are not important in a particular case may appear. Conversely, the absence of alerts does not guarantee there are no aspects of the results needing attention. It is up to the individual to critically assess their own results and, if necessary, seek expert advice.

If level A alerts remain, which you believe to be justified deviations, and you intend to submit this CIF for publication in a journal, you should additionally insert an explanation in your CIF using the Validation Reply Form (VRF) below. This will allow your explanation to be considered as part of the review process.

Validation response form

Please find below a validation response form (VRF) that can be filled in and pasted into your CIF.

```
# start Validation Reply Form
_vrf_PUBL004_GLOBAL
;
PROBLEM: The contact author’s name and address are missing,
RESPONSE: ...
;
_vrf_PUBL005_GLOBAL
;
PROBLEM: _publ_contact_author_email, _publ_contact_author_fax and
RESPONSE: ...
;
_vrf_PUBL006_GLOBAL
;
PROBLEM: _publ_requested_journal is missing
RESPONSE: ...
;
_vrf_PUBL008_GLOBAL
;
PROBLEM: _publ_section_title is missing. Title of paper.
RESPONSE: ...
;
_vrf_PUBL009_GLOBAL
;
PROBLEM: _publ_author_name is missing. List of author(s) name(s).
RESPONSE: ...
;
_vrf_PUBL010_GLOBAL
;
PROBLEM: _publ_author_address is missing. Author(s) address(es).
RESPONSE: ...
;
_vrf_PUBL012_GLOBAL
;
```

```
PROBLEM: _publ_section_abstract is missing.
RESPONSE: ...
;
# end Validation Reply Form
```

If you wish to submit your CIF for publication in Acta Crystallographica Section C or E, you should upload your CIF via the web. If you wish to submit your CIF for publication in IUCrData you should upload your CIF via the web. If your CIF is to form part of a submission to another IUCr journal, you will be asked, either during electronic submission or by the Co-editor handling your paper, to upload your CIF via our web site.

PLATON version of 18/09/2020; check.def file version of 20/08/2020

Datablock I - ellipsoid plot

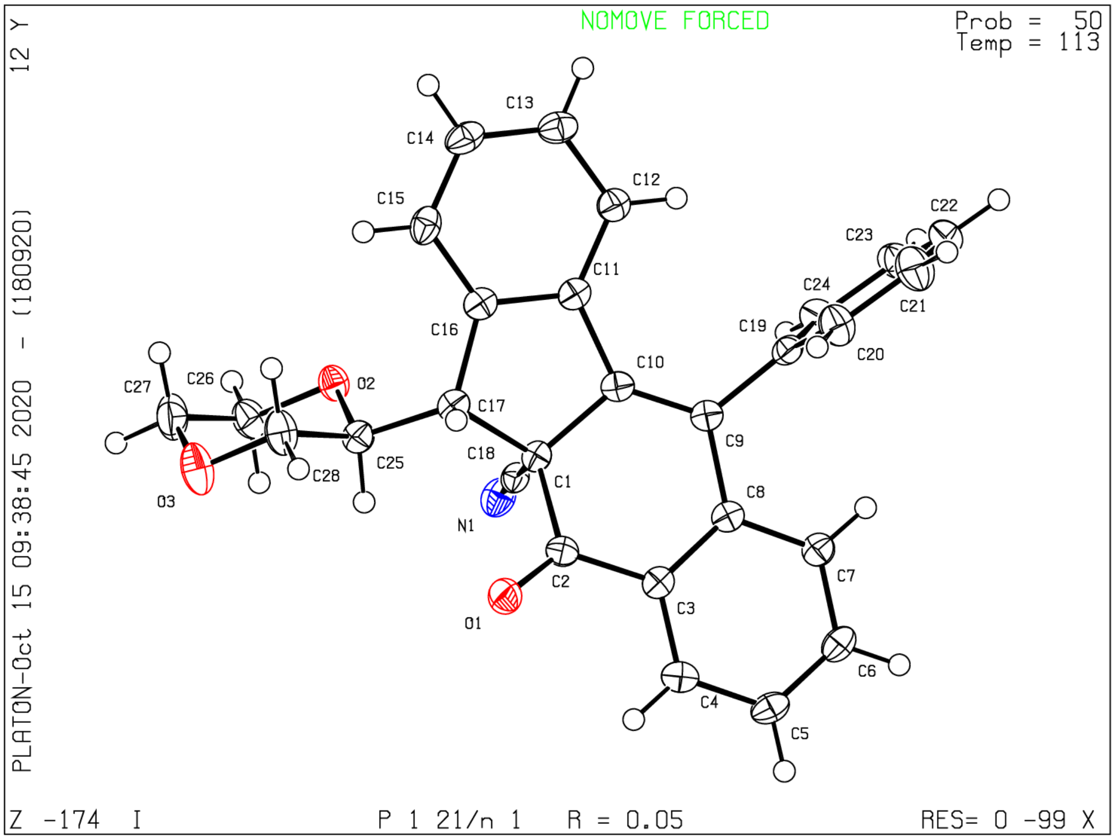

checkCIF/PLATON report

Structure factors have been supplied for datablock(s) eggpr3

THIS REPORT IS FOR GUIDANCE ONLY. IF USED AS PART OF A REVIEW PROCEDURE FOR PUBLICATION, IT SHOULD NOT REPLACE THE EXPERTISE OF AN EXPERIENCED CRYSTALLOGRAPHIC REFEREE.

No syntax errors found. CIF dictionary Interpreting this report

Datablock: eggpr3

```
Bond precision:   C-C = 0.0021 A          Wavelength=0.71073

Cell:          a=10.1865(5)      b=10.3420(6)       c=10.8011(6)
               alpha=84.945(2)   beta=64.5022(19)   gamma=63.1450(19)
Temperature:   150 K

                Calculated                   Reported
Volume          908.26(9)                    908.26(9)
Space group     P -1                         P -1
Hall group      -P 1                         -P 1
Moiety formula  C25 H15 N O3                 C25 H15 N O3
Sum formula     C25 H15 N O3                 C25 H15 N O3
Mr              377.38                       377.38
Dx,g cm-3       1.380                        1.380
Z               2                            2
Mu (mm-1)       0.091                        0.091
F000            392.0                        392.0
F000'           392.18
h,k,lmax        12,12,13                     12,12,13
Nref            3714                         3637
Tmin,Tmax       0.970,0.986                  0.852,0.928
Tmin'           0.962

Correction method= # Reported T Limits: Tmin=0.852 Tmax=0.928
AbsCorr = MULTI-SCAN

Data completeness= 0.979           Theta(max)= 26.387

R(reflections)= 0.0380( 3077)                        wR2(reflections)=
                                                     0.1129( 3637)
S = 0.989                  Npar= 262
```

The following ALERTS were generated. Each ALERT has the format
test-name_ALERT_alert-type_alert-level.
Click on the hyperlinks for more details of the test.

Alert level C

```
PLAT910_ALERT_3_C Missing # of FCF Reflection(s) Below Theta(Min).          7 Note
PLAT911_ALERT_3_C Missing FCF Refl Between Thmin & STh/L=    0.600         65 Report
PLAT913_ALERT_3_C Missing # of Very Strong Reflections in FCF ....         15 Note
PLAT934_ALERT_3_C Number of (Iobs-Icalc)/Sigma(W) > 10 Outliers ..          1 Check
```

Alert level G

```
PLAT230_ALERT_2_G Hirshfeld Test Diff for    C18      --C19      .        5.2 s.u.
PLAT398_ALERT_2_G Deviating  C-O-C   Angle From 120 for O2       .      106.0 Degree
PLAT398_ALERT_2_G Deviating  C-O-C   Angle From 120 for O3       .      105.8 Degree
PLAT793_ALERT_4_G Model has Chirality at C18         (Centro SPGR)          S Verify
PLAT883_ALERT_1_G No Info/Value for _atom_sites_solution_primary .     Please Do !
PLAT898_ALERT_4_G Second Reported H-M Symbol in CIF Ignored ......          ! Check
PLAT912_ALERT_4_G Missing # of FCF Reflections Above STh/L=  0.600          5 Note
PLAT933_ALERT_2_G Number of HKL-OMIT Records in Embedded .res File          2 Note
PLAT941_ALERT_3_G Average HKL Measurement Multiplicity ..........         2.9 Low
PLAT965_ALERT_2_G The SHELXL WEIGHT Optimisation has not Converged     Please Check
PLAT978_ALERT_2_G Number C-C Bonds with Positive Residual Density.         16 Info
```

```
   0 ALERT level A = Most likely a serious problem - resolve or explain
   0 ALERT level B = A potentially serious problem, consider carefully
   4 ALERT level C = Check. Ensure it is not caused by an omission or oversight
  11 ALERT level G = General information/check it is not something unexpected

   1 ALERT type 1 CIF construction/syntax error, inconsistent or missing data
   6 ALERT type 2 Indicator that the structure model may be wrong or deficient
   5 ALERT type 3 Indicator that the structure quality may be low
   3 ALERT type 4 Improvement, methodology, query or suggestion
   0 ALERT type 5 Informative message, check
```

Validation response form

Please find below a validation response form (VRF) that can be filled in and pasted into your CIF.

```
# start Validation Reply Form
_vrf_PLAT910_eggpr3
;
PROBLEM: Missing # of FCF Reflection(s) Below Theta(Min).          7 Note
RESPONSE: ...
;
_vrf_PLAT911_eggpr3
;
PROBLEM: Missing FCF Refl Between Thmin & STh/L=    0.600         65 Report
RESPONSE: ...
;
_vrf_PLAT913_eggpr3
;
```

```
PROBLEM: Missing # of Very Strong Reflections in FCF ....         15 Note
RESPONSE: ...
;
_vrf_PLAT934_eggpr3
;
PROBLEM: Number of (Iobs-Icalc)/Sigma(W) > 10 Outliers ..          1 Check
RESPONSE: ...
;
# end Validation Reply Form
```

It is advisable to attempt to resolve as many as possible of the alerts in all categories. Often the minor alerts point to easily fixed oversights, errors and omissions in your CIF or refinement strategy, so attention to these fine details can be worthwhile. In order to resolve some of the more serious problems it may be necessary to carry out additional measurements or structure refinements. However, the purpose of your study may justify the reported deviations and the more serious of these should normally be commented upon in the discussion or experimental section of a paper or in the "special_details" fields of the CIF. checkCIF was carefully designed to identify outliers and unusual parameters, but every test has its limitations and alerts that are not important in a particular case may appear. Conversely, the absence of alerts does not guarantee there are no aspects of the results needing attention. It is up to the individual to critically assess their own results and, if necessary, seek expert advice.

Publication of your CIF in IUCr journals

A basic structural check has been run on your CIF. These basic checks will be run on all CIFs submitted for publication in IUCr journals (*Acta Crystallographica*, *Journal of Applied Crystallography*, *Journal of Synchrotron Radiation*); however, if you intend to submit to *Acta Crystallographica Section C* or *E* or *IUCrData*, you should make sure that full publication checks are run on the final version of your CIF prior to submission.

Publication of your CIF in other journals

Please refer to the *Notes for Authors* of the relevant journal for any special instructions relating to CIF submission.

PLATON version of 28/11/2022; check.def file version of 28/11/2022

Datablock eggpr3 - ellipsoid plot

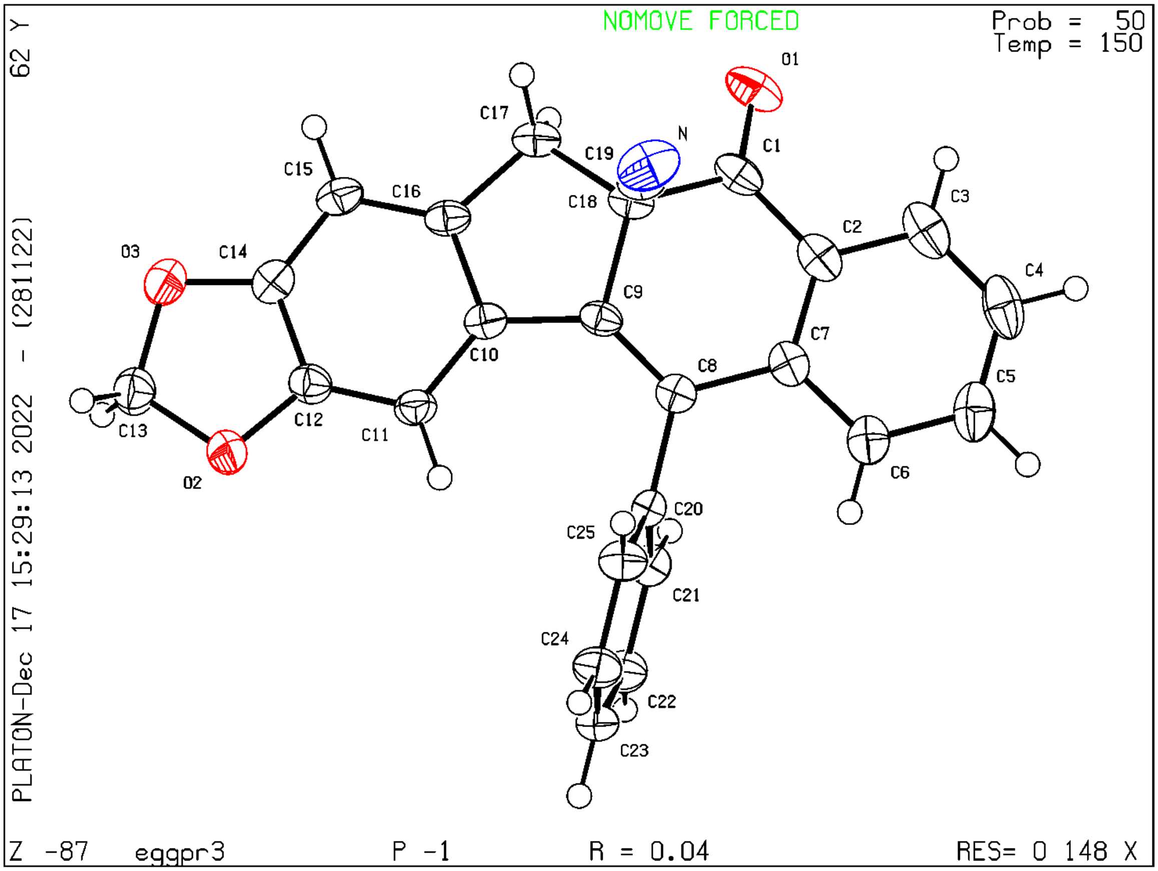

Supplement: Supplementary file 1 — ol3c04246_si_001.pdf [file ol3c04246_si_001.pdf]
